# Factors associated with isoniazid resistant tuberculosis among human immunodeficiency virus positive patients in Swaziland: a case-control study

**DOI:** 10.1186/s12879-019-4384-6

**Published:** 2019-08-20

**Authors:** Nonhlanhla Christinah Dlamini, Dar-Der Ji, Li-Yin Chien

**Affiliations:** 1 Graduate student, International Health Program, National Yang-Ming University, Taipei, Taiwan; 2 National drug resistant TB coordinator, Swaziland National TB Control Programme, Manzini, Swaziland; 3 Associate Professor, Division of Tropical Medicine, Department of Medicine, National Yang-Ming University, Taipei, Taiwan; 4 Professor, Institute of Community Health Care, National Yang-Ming University, 155, Section 2, Li-Nong Street, Beitou, Taipei, 11221 Taiwan

**Keywords:** Drug resistance, Human immunodeficiency virus, Isoniazid, Risk factors, Tuberculosis, Swaziland

## Abstract

**Background:**

Isoniazid resistant tuberculosis is the most prevalent type of resistance in Swaziland and over two-thirds of the isoniazid resistant tuberculosis patients are tuberculosis and human immunodeficiency virus co-infected. The study aimed to determine risk factors associated with isoniazid resistant tuberculosis among human immunodeficiency virus positive patients in Swaziland.

**Methods:**

This was a case-control study conducted in nine healthcare facilities across Swaziland. Cases were patients with isoniazid resistant tuberculosis (including 78 patients with isoniazid mono-resistant tuberculosis, 42 with polydrug-resistant tuberculosis, and 77 with multidrug-resistant tuberculosis). Controls were presumed drug-susceptible tuberculosis patients (*n* = 203). Multinomial logistic regression was used to determine related factors.

**Results:**

The median time lag from diagnosis to tuberculosis treatment initiation was 50 days for isoniazid mono or poly drug-resistant tuberculosis, 17 days for multidrug-resistant tuberculosis compared to 1 day for drug-susceptible tuberculosis patients. History of previous tuberculosis treatment was positively associated with either isoniazid mono or poly drug-resistant tuberculosis (OR = 7.91, 95% CI: 4.14–15.11) and multidrug-resistant tuberculosis (OR = 12.20, 95% CI: 6.07–24.54). Isoniazid mono or poly resistant tuberculosis patients were more likely to be from rural areas (OR = 2.05, 95% CI: 1.23–3.32) and current heavy alcohol drinkers compared to the drug-susceptible tuberculosis group. Multi drug-resistant tuberculosis patients were more likely to be non-adherent to tuberculosis treatment compared to drug-susceptible tuberculosis group (OR = 3.01, 95% CI: 1.56–5.82).

**Conclusion:**

To prevent and control isoniazid resistant tuberculosis among HIV-positive patients in Swaziland, the tuberculosis program should strengthen the use of rapid diagnostic tests, detect resistance early, promptly initiate supervised tuberculosis treatment and decentralize quality tuberculosis services to the rural areas. Adherence to tuberculosis treatment should be improved.

**Electronic supplementary material:**

The online version of this article (10.1186/s12879-019-4384-6) contains supplementary material, which is available to authorized users.

## Background

Drug-resistant tuberculosis (DR-TB) is a global challenge faced mostly by low- and middle-income countries. A survey in 2008 revealed that 10.3% of new cases and 27.7% of previously treated cases had TB strains resistant to isoniazid worldwide [1]. Globally, in 2015, 3.9% of new cases and 21% of previously treated cases had multidrug-resistant TB (MDR-TB) or rifampicin-resistant TB (RR-TB) [2] .

Swaziland is one of the countries affected by the scourge of DR-TB. A nationwide DR-TB survey conducted in 2009–2010 revealed that 13.5 and 45.2% of new and previously treated cases, respectively, had isoniazid resistant TB; 7.7% of the new cases and 33.8% of the previously treated cases had MDR-TB. Human immunodeficiency virus (HIV) has been identified as the main driver of DR-TB in Swaziland, as 80% of the TB patients were co-infected with HIV [3]. Isoniazid resistant TB is the most prevalent type of resistance in Swaziland, and is a major concern, because isoniazid is one of the most effective and specific first-line drugs for the prevention and treatment of TB [4, 5]. Additionally, the development of isoniazid resistant TB is usually the first step in the evolution of MDR-TB or even more complicated types of DR-TB [5–7].

Many international studies have presented risk factors for isoniazid resistant TB. These include previous history of TB treatment [3, 8, 9], poor adherence to TB treatment regimens [8, 10], HIV [8], younger age (15–40 years) [9, 11–13], alcohol use, smoking [11, 14], male sex [9, 11–13, 15], diabetes [16, 17], TB contact [11, 18, 19], and occupation [10]. In addition to these factors, some studies have associated isoniazid mono-resistant TB (HR-TB) with history of isoniazid prophylaxis therapy (IPT) [20], history of imprisonment, and unemployment [11].

Although DR-TB is a priority in Swaziland, there has been no study published on risk factors associated with isoniazid resistant TB among HIV positive patients. Therefore, there is a need for empirical evidence to guide the TB and HIV programs on the drivers of isoniazid resistant TB and to enable the programs to develop interventions to curb the situation. Furthermore, the government of Swaziland has implemented numerous interventions to reduce the burden of TB among HIV-positive people. These include intensified TB case findings, rapid diagnostic tests for TB (Xpert MTB/RIF), capacity to conduct both first- and second-line TB drug-susceptibility tests locally, IPT for HIV-positive patients who have been screened negative for TB, scaled-up antiretroviral treatment (ART), TB drugs procured from quality-assured companies, decentralized TB and HIV services, and integrated TB/HIV services.

Since the implementation of these interventions, there is a progressive decline in drug-susceptible TB (DS-TB) cases and a non-significant decrease in the number of DR-TB cases [3]. Thus, the objective of the present study was to explore the risk factors associated with isoniazid resistant TB (HR-TB plus poly drug-resistant TB (PDR-TB) or MDR-TB), including socio-demographic, clinical, and behavioral characteristics among HIV-positive patients in Swaziland.

## Methods

### Design and participants

This was a case-control study conducted from July through September 2016. The data were collected from nine healthcare facilities, including all the eight facilities that provide DR-TB care plus one TB/HIV facility that had the highest number of DS-TB/HIV positive patients. When a patient presents to the facility for any reason, he or she is screened for TB using the signs and symptoms screening tool. If TB presumptive positive by the screening tool, a sample for gene Xpert is taken. The gene Xpert has capacity to detect *Mycobacterium tuberculosis* (MTB) and rifampicin resistance. If MTB positive and rifampicin resistance not detected, the patient is assumed to be having drug susceptible TB (DS-TB), then the patient is initiated on DS-TB treatment. If MTB positive and rifampicin resistance detected, the patient is initiated on MDR-TB standardized regimen. If MTB not detected, the patient was considered TB negative.

In the study, cases included those with HR-TB, PDR-TB, and MDR-TB diagnosed by culture and drug susceptibility testing (C&DST) and line probe assay (LPA) plus co-infected with HIV. HR-TB patients were those with TB resistant to isoniazid only. PDR-TB was defined as TB resistant to isoniazid and one of the following first-line anti-TB drugs: ethambutol and streptomycin, but not resistant to rifampicin. MDR-TB was defined as TB resistant to rifampicin and isoniazid with or without resistance to the other first-line drugs.

Controls were patients diagnosed by Xpert MTB/RIF to be MTB positive and rifampin resistance not detected. The DS-TB patients were on treatment for at least 2 months, and had converted to smear-negative status. Smear conversion at 2 or 3 months was used as a proxy for drug susceptibility. C&DST is not yet universal in Swaziland. According to the Eswatini DR-TB guidelines, 2012, C&DST was supposed to be done to all patients who had history of previous TB treatment, failed to convert at 2–3 months of first-line TB treatment, failed the first-line TB treatment, are contacts of DR-TB patients, and health care workers (nurses, doctors, laboratory staff). But there are facilities that are supported by partners like medicins san frontiers (MSF), where C&DST is universal for all TB presumptive cases and 65% of our controls were from those facilities.

According to the TB manual, 2012 and the DR-TB guideline, 2012 (Additional files 1 and 2), all persons diagnosed with TB should be tested for HIV and HIV positive should be screened for TB. To diagnose HIV, parallel testing was implemented, *determine* test was the first test that was done, if positive, *unigold test* was then done*,* if positive, the patient was diagnosed as HIV positive. Based on the guidelines, all TB patients who are HIV positive should be initiated on antiretroviral treatment regardless of CD4 count.

HIV infected patients were recruited if they had a documented HIV positive status on the TB register. The cases were selected from the DR-TB register and the controls from the DS-TB register. The participants were enrolled in the consecutive order as they appeared on the register until the desired sample size was reached. We estimated the desired sample size of 197 for DR-TB cases and DS-TB group each, based on an estimation of OR = 1.8, probability in the control group = 0.3, two-sided alpha = 0.05, and power = 0.8.

In this study, patients younger than 18 years were excluded. For the case group, patients initiated on second-line TB treatment without C&DST and extensively DR-TB cases (resistance to any fluoroquinolone and at least of one of the 3 second-line injectables (kanamycin, amikacin, or capreomycin) in addition to MDR-TB) were excluded. For the control group, we excluded those who were TB negative at the beginning of TB treatment. A total of 400 TB/HIV patients participated in the study: 197 with DR-TB (78 with HR-TB, 42 PDR-TB, and 77 MDR-TB) and 203 with DS-TB. Of the 203 controls, 35% (*n* = 71) were TB patients who were diagnosed by Xpert MTB/RIF to be MTB positive and rifampin resistance not detected and were on treatment for at least 2 months, and had converted to smear-negative status and 65% (*n* = 132) were confirmed DS-TB by C&DST in addition to the above-mentioned criteria. Of the 400 participants, 212 had HIV diagnosed before TB (outside the TB department) and 188 were diagnosed with HIV within the TB department post TB diagnosis; 9 had not been initiated on antiretroviral treatment at 2 months after TB treatment initiation due to their refusal. The study protocol has been approved by the Swaziland Scientific and Ethics Committee. The study participants signed an informed consent form.

### Measurements

Data were collected through face-to-face interviews and review of medical records. The interviews were conducted using a structured questionnaire, and the medical record reviews were performed using a case form. The questionnaire was pre-tested on 10 patients at a TB center. Necessary amendments were made to improve the clarity of the questionnaire. The data collectors, nurses or TB data clerks, were trained on data collection tools onsite.

The dependent variable, drug-resistant status, was divided into 3 groups, HR-TB or PDR-TB, MDR-TB, and DS-TB. We combined HR-TB and PDR-TB as one group since isoniazid and rifampicin were the most powerful first-line TB drugs and HR-TB and PDR-TB in the study can be treated with rifampicin. In addition, the sample size for PDR-TB was not large enough to compose an individual group in the analysis. The independent variables included the socio-demographic characteristics (age, sex, marital status, level of education, occupation, residence, and region), clinical characteristics (history of previous TB treatment, length of time from diagnosis to treatment initiation (date from results authorization at laboratory level to treatment initiation), history of TB contact, history of IPT, whether patient started ART before current episode of TB, CD4 count at TB diagnosis, diabetes, TB treatment non-adherence (self-rated adherence to the current TB treatment being less than 100%) and HIV treatment non-adherence (self-rated adherence to the ART treatment being less than 100%), and behavioral characteristics (smoking status, alcohol drinking status, and history of imprisonment). For patients with previous TB treatment, we classified them as “recurrence,” “retreatment after failure,” and “retreatment after lost to follow-up.” Recurrence was defined when a patient previously treated for TB with the most recent treatment curing TB or the treatment being completed, and who is subsequently diagnosed with a recurrent episode of TB. Heavy alcohol drinking was determined based on whether the patient had ever drunk > 150 glasses of alcohol beverage per month during the past 12 months (current drinker), or during the lifetime but not in the past 12 months (previous drinker), or had never drunk > 150 glasses of alcohol beverage per month during his lifetime (never).

Data on socio-demographics, behavioral characteristics, IPT exposure, TB and HIV treatment adherence, and comorbid conditions (diabetes) of patients were collected through face-to-face interviews. Other clinical characteristics were collected through the review of medical records.

### Data analysis

The data analyses were carried out using IBM SPSS statistics 22. The characteristics of the three groups (HR-TB or PDR-TB, MDR-TB, and DS-TB) were compared using the chi-squared test, ANOVA, and Kruskal-Wallis test. Multinomial logistic regression was used to determine factors associated with isoniazid resistant tuberculosis. Isoniazid resistant tuberculosis was divided into HR-TB or PDR-TB, and MDR-TB, with DS-TB being the reference group. In the multinomial logistic regression, exponential ßs were the odds ratios (OR) for the predictors. The odds ratio of a coefficient indicates how the risk of the outcome falling in the comparison group compared to the risk of the outcome falling in the reference group changes with the variable in question. Backward selection with one least significant variable being dropped from the full model each time until all variables in the model having a 2-sided alpha of < 0.05 was used to select the final parsimonious model.

## Results

The drug susceptibility test results among the study participants are presented in Table 1. Comparisons of socio-demographic characteristics among the HR-TB or PDR-TB (*n* = 120), MDR-TB (*n* = 77), and DS-TB (*n* = 203) groups are presented in Table 2. Of the socio-demographic characteristics, only occupation and residence were significantly different among the three groups. Most of the cases were unemployed compared to controls, with 50% of HR-TB or PDR-TB patients and 44.2% of MDR-TB patients being unemployed compared to only 35.1% of DS-TB patients (*p* = 0.028). Moreover, higher percentage of the cases lived in rural areas compared to controls with 60.8% of HR-TB or PDR-TB patients and 51.9% of MDR-TB patients living in rural areas compared to 41.1% of DS-TB patients (*p* = 0.003).

**Table 1:** Drug susceptibility test results

	HR-TB *n* = 78	PDR-TB *n* = 42	MDR-TB *n* = 77	DS-TB *n* = 203
R(H)	78 (100%)	0	0	0
R (RH)	0	0	28 (36.4%)	0
R (HES)	0	38 (90.5%)	0	0
R (RHES)	0	0	20 (26.0%)	0
R (RHE)	0	0	15 (19.5%)	0
R (HE)	0	4 (9.5%)	0	0
R (RHS)	0	0	14 (18.2%)	0
S (RHES)	0	0	0	131 (64.5%)
S (R)	0	0	0	72 (35.5%)

R(H): Resistance to Isoniazid; R (RH): resistant to rifampicin and isoniazid; R (HES): resistance to isoniazid, ethambutol and streptomycin; R (RHES): resistance to rifampicin, isoniazid, ethambutol and streptomycin; R (RHE): resistance to rifampicin, isoniazid and ethambutol; R (HE): Resistance to isoniazid and ethambutol; S (RHES): susceptible to rifampicin, isoniazid, ethambutol and streptomycin; S(R): susceptibility to rifampicin by gene xpert

*HR-TB* Isoniazid mono-resistant tuberculosis, *PDR-TB* poly drug-resistant tuberculosis, *MDR-TB* multi drug-resistant tuberculosis, *DS-TB* drug-susceptible tuberculosis

**Table 2:** Socio-demographic characteristics of the study participants (*N* = 400)

Characteristics	HR-TB or PDR-TB (*n* = 120)	MDR-TB (*n* = 77)	DS-TB (*n* = 203)	
	n (%)	n (%)	n (%)	*p*-value
Mean age (SD) in years	36.5 (9.55)	35.9 (9.54)	36.0 (9.54)	0.878
Male	55 (45.8)	42 (54.5)	108 (53.2)	0.358
Marital status				0.848
Married	42 (35.0)	29 (37.7)	69 (34.0)	
Single	78 (65.0)	48 (62.3)	134 (66.0)	
Educational level				0.330
Secondary and above	77 (64.2)	57 (74.0)	141 (69.5)	
Primary and below	43 (35.8)	20 (26.0)	62 (30.5)	
Occupation				0.028*
Unemployed	60 (50.0)	34 (44.2)	71 (35.1)	
Employed	60 (50.0)	43 (55.8)	131 (64.9)	
Residence				0.003*
Rural	73 (60.8)	40 (51.9)	84 (41.1)	
Urban	47 (39.2)	37 (48.1)	119 (58.6)	
Region				0.802
Manzini	93 (77.5)	63 (80.5)	156 (76.8)	
Other regions	27 (22.5)	15 (19.5)	47 (23.2)	

*HR-TB* Isoniazid-mono resistant tuberculosis, *PDR-TB* poly drug-resistant tuberculosis, *MDR-TB* multi drug-resistant tuberculosis, *DS-TB* drug-susceptible tuberculosis. Other regions include Hhohho, Shiselweni and Lubombo

*p <  0.05. Occupation did not add-up because of 1 missing

Of the clinical and behavioral characteristics (Table 3), case definition, ART started before current TB diagnosis, median time from diagnosis to treatment initiation, TB treatment non-adherence, diabetes, and heavy alcohol drinking were significantly different among the three groups. About 17.5% of patients with HR-TB or PDR-TB and 33.8% of MDR-TB were recurrent patients compared to 7.9% of patients with DS-TB. Additionally, 21.7% of HR-TB or PDR-TB patients and 14.3% of MDR-TB patients were retreatment cases after first-line treatment failure compared to none among the DS-TB group (*p* <  0.0001). A higher percentage of HR-TB or PDR-TB (65.0%) patients and MDR-TB patients (58.4%) had started ART before the current episode of TB compared to DS-TB patients (43.8%). The median number of days (IQR) from diagnosis to treatment initiation was 50 (31) days for HR-TB or PDR-TB patients, 17 (57) days for MDR-TB patients, compared to 1 (4) day for DS-TB patients. Although there were a few patients with diabetes among the study participants, most of them had HR-TB or PDR-TB (5.0%) compared to MDR-TB (2.6%) and DSTB (0.5%). A higher percentage of patients with MDR-TB (30.6%) showed non-adherence to TB treatment compared to patients with HR-TB or PDR-TB (22.4%) and DSTB (13.3%). A higher percentage of HR-TB or PDR-TB patients (22.5%) appeared to be current heavy alcohol drinkers than MDR-TB patients (11.7%) and DS-TB patients (10.3%).

**Table 3:** Clinical and behavioral characteristics of the study participants (*N* = 400)

Characteristics	HR-TB or PDR-TB (*n* = 120)	MDR-TB (*n* = 77)	DS-TB (*n* = 203)	
	n (%)	n (%)	n (%)	*P*-value
Case definition				< 0.0001*
New	72 (60.0)	39 (50.6)	187 (92.1)	
Recurrence	21 (17.5)	26 (33.8)	16 (7.9)	
Retreatment after failure	26 (21.7)	11 (14.3)	0 (0)	
Retreatment after lost to follow-up	1 (0.8)	1 (1.3)	0 (0)	
History of TB contact	41 (34.2)	24 (31.2)	52 (25.6)	0.242
Median length of time (days) from diagnosis to treatment initiation (IQR)	50 (31)	17 (57)	1 (4)	< 0.0001*
IPT exposure	12 (10.0)	4 (5.2)	16 (7.9)	0.477
On ART	120 (100)	75 (97.4)	196 (96.6)	0.129
ART started before current TB episode	78 (65.0)	45 (58.4)	89 (43.8)	0.001*
CD4 count at TB diagnosis				0.680
0–200	74 (61.7)	55 (71.4)	125 (61.6)	
> 200–350	18 (15.0)	11 (14.3)	35 (17.2)	
> 350–500	16 (13.3)	5 (6.5)	21 (10.3)	
> 500	12 (10.0)	6 (7.8)	22 (10.8)	
Diabetes	6 (5.0)	2 (2.6)	1 (0.5)	0.030*
TB treatment non-adherence	11 (22.4)	19 (30.6)	27 (13.3)	0.005*
ART non-adherence	26 (21.7)	17 (22.4)	26 (13.3)	0.078
Smoking Status				0.051
Never	74 (61.7)	60 (77.9)	152 (74.9)	
Previous	29 (24.2)	13 (16.9)	35 (17.2)	
Current	17 (14.2)	4 (5.2)	16 (7.9)	
Heavy alcohol drinking				0.046*
Never	66 (55.0)	49 (63.6)	131 (64.5)	
Previous	27 (22.5)	19 (24.7)	51 (25.1)	
Current	27 (22.5)	9 (11.7)	21 (10.3)	
History of imprisonment	13 (10.8)	8 (10.4)	24 (11.8)	0.930

*HR-TB* Isoniazid mono-resistant tuberculosis, *PDR-TB* poly drug-resistant tuberculosis; *MDR-TB* multi drug-resistant tuberculosis, *DS-TB* drug-susceptible tuberculosis, *IPT* Isoniazid prophylaxis treatment, *ART* antiretroviral treatment, *TB* tuberculosis

*p < 0.05

The parsimonious multinomial logistic regression model (Table 4) showed that history of previous treatment was positively associated with either HR-TB or PDR-TB (OR = 7.91, 95% CI: 4.14–15.11) and MDR-TB (OR = 12.20, 95% CI: 6.07–24.54). Individuals who lived in rural areas (OR = 2.02, 95% CI: 1.23–3.32), and were current heavy alcohol drinkers were more likely to have HR-TB or PDR-TB compared to the DSTB group. Patients in the MDR-TB group were more likely to be non-adherent to TB treatment compared to the DSTB group (OR = 3.01, 95% CI: 1.56–5.82).

**Table 4:** Parsimonious multinomial logistic regression model for factors associated with HR-TB and PDR-TB or MDR-TB, in reference to DS-TB (*N* = 400)

Variable	HR-TB or PDR-TB	MDR-TB
	OR [95% CI]	OR [95% CI]
Residence: Rural	2.02 [1.23–3.32]*	1.27 [0.71–2.27]
History of previous TB treatment	7.91 [4.14–15.11]*	12.20 [6.07–24.54]*
Heavy alcohol drinking		
Current	1	1
Previous	0.36 [0.16–0.81]*	0.76 [0.27–2.13]
Never	0.46 [0.23–0.92]*	1.16 [0.46–2.93]
TB treatment non-adherence	1.52 [0.81–2.86]	3.01 [1.56–5.82]*

*HR-TB* Isoniazid mono-resistant tuberculosis, *PDR-TB* poly drug-resistant tuberculosis, *MDR-TB*: multi drug-resistant tuberculosis, *DS-TB* drug-susceptible tuberculosis

*p < 0.05

To address the concern that patients with a history of previous treatment may be more likely to be in the case group, we excluded patients with a history of previous treatment and re-ran the parsimonious model (Table 5). The adjusted OR decreased for living in rural area and lost its statistical significance, but the direction remained the same. The adjusted OR for TB treatment non-adherence increased and were significant for both case groups. The adjusted OR for heavy alcohol drinking appeared to be similar.

**Table 5:** Parsimonious multinomial logistic regression model for factors associated with HR-TB and PDR-TB or MDR-TB, in reference to DS-TB, excluding patients with a history of previous treatment (*N* = 298)

Variable	HR-TB or PDR-TB	MDR-TB
	OR [95% CI]	OR [95% CI]
Residence: Rural	1.55 [0.88–2.72]	1.02 [0.50–2.08]
Heavy alcohol drinking		
Current	1	1
Previous	0.31 [0.12–0.81]*	0.76 [0.30–3.96]
Never	0.45 [0.21–0.97]*	1.09 [0.29–3.09]
TB treatment non-adherence	2.09 [1.01–4.35]*	3.26 [1.43–7.45]*

*HR-TB* Isoniazid mono-resistant tuberculosis, *PDR-TB* poly drug-resistant tuberculosis; *MDR-TB* multi drug-resistant tuberculosis, *DS-TB* drug-susceptible tuberculosis

*p < 0.05

## Discussion

The long time lag between diagnosis and initiation of TB treatment in patients with HR-TB or PDR-TB, and with MDR-TB might be a significant factor for isoniazid resistant TB among HIV-positive patients in Swaziland. The time lag in the study meant the time taken to initiate a TB patient on treatment after diagnosis with either LPA or C&DST or gene Xpert testing regardless of the number of effective drugs. This time lag may be due to underutilization of LPA and delayed dispatch of results from national reference laboratory to facilities. The time lag between diagnosis and initiation of effective treatment means that the patient will continue to spread the infection. This has been supported by Nardell, et al. (2010) who stated that there is longstanding evidence suggesting that TB patients on effective treatment rapidly become non-infectious and that unsuspected or untreated TB cases account for transmission the most [21]. This might be true for Swaziland as well, given that, as more than 90% of the study participants were on ART, most of them had CD4 counts of 200 cells/microliter or even lower at TB diagnosis, which may be the reason for infection. Most of the isoniazid resistant TB patients started ART before the current episode of TB, which may suggest facility transmission of isoniazid resistant TB.

Recurrence and failure of previous treatment was found to be a predictor of resistance to all kind of isoniazid resistant TB in the study. This result is obvious because those patients were prioritized for DST in the study context. But a history of previous TB treatment was still included in the study to emphasize the importance of universal C&DST to allow for early initiation of effective treatment to patients thus cutting the spread of TB and preventing resistance amplification. This is supported by a study conducted by Ragonnet et al. (2017) and World Health Organization who found that failure to diagnose DR-TB at first presentation is the leading cause of the high proportion of DR-TB among re-treatment cases [10, 22]. This was further echoed by a systematic review study conducted Gegia et al. (2017) which analyzed 19 cohort studies and 33 trials and revealed that treatment of isoniazid resistant tuberculosis with the WHO standard regimen for new patients resulted in treatment failure, relapse, and acquired multi drug-resistance in 11% (6–17), 10% (5–15) and 8% (3–13) of patients, respectively [23].

Current heavy alcohol drinking was significantly associated with HR-TB or PDR-TB, consistent with the results of previous studies [11, 15]. According to a systematic review conducted by Rehm et al. (2009), many studies showed pathogenic impact of alcohol on the immune system causing susceptibility to TB among heavy drinkers. Heavy alcohol use influences both the incidence and the outcome of the disease and was found to be linked to altered pharmacokinetics of medicines used in treatment of TB, higher rate of re-infection, higher rate of treatment defaults, and development of drug-resistant forms of TB [24]. This may also be attributed to the fact that alcohol shops in Swaziland are poorly ventilated, thus promoting the spread of TB among the immunocompromised (HIV-positive) alcohol drinkers.

Moreover, HR-TB and PDR-TB was associated with rural residence. This might be attributed to the fact that living in rural areas may increase a patient’s risk of being exposed to poor living conditions or malnutrition, and having poor access to diagnosis and treatment because of financial constraints and scarcity of accessible healthcare services, consequently enhancing the spread of isoniazid resistant TB [10, 15, 25]. Rural residence lost its’ significance in the model when the analysis was restricted to patients without previous history of treatment. But TB treatment non-adherence increased its strength and significance in the association. This results suggest a positive association between rural residence and TB treatment non-adherence.

MDR-TB was associated with non-adherence to TB treatment. If the analysis was restricted to patients without previous history of treatment, non-adherence to TB treatment was positively associated with HR-TB and PDR-TB as well as MDR-TB. The results are consistent with the results of a study conducted by Naidoo et al. (2013) and a companion handbook on programmatic management of MDR-TB from the World Health Organization, which identified poor adherence to TB treatment as a risk factor for DR-TB [10, 26]. This might be explained by the association of TB with poverty, with most patients not adhering to treatment because of lack of food or lack of money for transport to visit healthcare facilities for treatment refill. Alternatively, TB is one of the most stigmatized diseases, and may also hinder adherence, or patients may not adhere due to side-effects [10].

IPT was not associated with isoniazid resistant TB in this study. Previous studies revealed mixed results on the effect of IPT on isoniazid resistant TB. A study by Cattamanchi et al. (2009) revealed that IPT is positively associated with HR-TB [20]. On the contrary, a meta-analysis showed that IPT was not significantly associated with HR-TB but findings did not exclude an increased risk for isoniazid resistant TB after IPT [27]. Results from the current study are inconclusive because less than 10% of the HIV-positive patients had received IPT; thus, to obtain valid results, future prospective study with a larger sample size is required.

This study has several limitations. As C&DST testing is not universal in Swaziland and the baseline TB diagnostic test (Xpert MTB/RIF) used has no capacity to detect isoniazid resistance, there was a chance of misclassifying cases as controls in the 35% (*n* = 71) of the DS-TB patients (controls) who did not receive C&DST to confirm their status. Hence, this might result in the underestimation of the association between the independent and the dependent variable. Diagnostic bias was possible because C&DST was done in patients not responding to treatment, being previously treated, and having DR-TB contacts. However, the significant risk factors were similar in the two models including or excluding patients with previous treatment. The sample size estimation was based on comparison between isoniazid resistant and control groups when the study was planned. But when we analyzed the data, we found that MDR-TB was quite different from HR- and PDR-TB. Dividing the drug resistance cases into sub-groups may decrease the statistical power of the study. Additionally, this study could not confer causality because temporality was not established. Current adherence to TB and HIV treatment was used to measure patients’ overall adherence status, and this may not accurately measure the overall adherence status or adherence status before development of drug resistance. Finally, the cases were on treatment for a maximum period of two years and controls were on treatment for a maximum of six months; this may compromise the comparability of cases and controls. Nonetheless, the generalizability of the results were enhanced by using a national sample from nine treatment facilities across Swaziland.

## Conclusions

For Swaziland to reduce the burden of isoniazid-resistant TB among HIV-positive patients, there is a need to strengthen the use of first-line LPA for prompt detection of isoniazid resistant TB and prompt initiation of treatment, conduct early HIV diagnosis and early ART initiation, implement interventions to reduce alcohol consumption, decentralize quality TB services to rural areas, and improve DOTs.

## Additional files

Additional file 1: National drug-resistant tuberculosis management guidelines. (DOCX 1345 kb)

Additional file 2: National tuberculosis programmemanual. (DOCX 1617 kb)

## Data Availability

The datasets used and/or analyzed during the current study are available from the corresponding author on reasonable request.

## Abbreviations

ART: Antiretroviral treatment
C&DST: Culture and drug susceptibility testing
DOT: Directly observed treatment
DR-TB: Drug-resistant tuberculosis
DS-TB: Drug-susceptible tuberculosis
HIV: Human immunodeficiency virus
HR-TB: Isoniazid mono-resistant tuberculosis
IPT: Isoniazid prophylaxis treatment
LPA: Line probe assay
MDR-TB: Multidrug-resistant tuberculosis
PDR-TB: Polydrug-resistant tuberculosis
RR-TB: Rifampicin-resistant tuberculosis
TB: Tuberculosis
