# Supplementary material for: Factors associated with isoniazid resistant tuberculosis among human immunodeficiency virus positive patients in Swaziland: a case-control study
Source: BMC Infect Dis. 2019 Aug 20;19:731. doi: 10.1186/s12879-019-4384-6 (PMC6701091; doi:10.1186/s12879-019-4384-6)

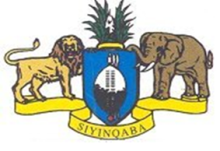


**Kingdom of Swaziland**

**MINISTRY OF HEALTH**

**NATIONAL DRUG-RESITANT TUBERCULOSIS**

**MANAGEMENT GUIDELINES**

**
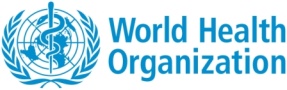
**

**
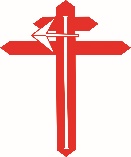
**

**National TB Control Programme**

***2^nd^ Edition 2012***

Publication Notice

Publication of the Swaziland National Tuberculosis Control Programme. All rights reserved.

Any enquiries regarding this publication or permission to reproduce or translate the material should be addressed to National Tuberculosis Control Programme, P.O Box 54 Manzini. Telephone +268-25057951, 2505-7454 or e-mail [dlaminip@tbprogram.org.sz](mailto:dlaminip@tbprogram.org.sz)

CONTENTS

[PREFACE 7](#_Toc335207485)

[FOREWORD 8](#_Toc335207486)

[ACKNOWLEDGEMENT 9](#_Toc335207487)

[EXECUTIVE SUMMARY 11](#_Toc335207488)

[1.1 Purpose of the guidelines 11](#_Toc335207489)

[1.2 Target audience 11](#_Toc335207490)

[1.3 Scope 11](#_Toc335207491)

[1.4 Justification for guideline update 11](#_Toc335207492)

[1.5 Highlights of the new revision 11](#_Toc335207493)

[CHAPTER 1 BACKGROUND INFORMATION 14](#_Toc335207494)

[1.1 Magnitude of the DR-TB problem 14](#_Toc335207495)

[1.2 DR-TB in Swaziland 14](#_Toc335207496)

[1.3 Causes of DR-TB 14](#_Toc335207497)

[CHAPTER 2 FRAMEWORK FOR DR-TB CONTROL IN SWAZILAND 17](#_Toc335207498)

[2.1 The strategic approach 17](#_Toc335207499)

[2.2 Coordination 18](#_Toc335207500)

[2.2.1 Coordination of stakeholder efforts: 18](#_Toc335207501)

[2.2.2 Coordination of case management between TB Diagnostic health Units and the National TB Hospital 18](#_Toc335207502)

[2.2.3 Coordination at Community level care: 20](#_Toc335207503)

[2.2.4 Coordination with prisons and other congregate and Institutional organizations 20](#_Toc335207504)

[2.2.5 Coordination of all health-care providers (both public and private) 21](#_Toc335207505)

[2.2.6 Coordination with the International partners 21](#_Toc335207506)

[CHAPTER 3 DEFINITIONS, CASE REGISTRATION, BACTERIOLOGY AND TREATMENT OUTCOMES 23](#_Toc335207507)

[3.1 Definitions of drug resistance 23](#_Toc335207508)

[3.2 DR-TB case registration groups 23](#_Toc335207509)

[3.2.1 Registration based on site of drug-resistant TB disease (pulmonary and extra-pulmonary) 23](#_Toc335207510)

[3.2.2 Registration based on bacteriological examinations and Sputum conversion 24](#_Toc335207511)

[3.2.3 DR-TB patient registration group based on history of previous anti-tuberculosis treatment 24](#_Toc335207512)

[3.3 Treatment outcome definitions for DR-TB treatment 25](#_Toc335207513)

[3.4 Analysis of Interim results and final treatment outcomes 26](#_Toc335207514)

[CHAPTER 4 DR-TB CASE FINDING STRATEGIES AND DIAGNOSIS 27](#_Toc335207515)

[4.1 DR-TB risk groups 27](#_Toc335207516)

[4.2 Rapid DST methods 28](#_Toc335207517)

[4.2.1 Xpert MTB/Rif (Gene Xpert) test 28](#_Toc335207518)

[4.2.1.1 Use of Xpert MTB/Rif in new TB cases 28](#_Toc335207519)

[4.2.1.2 Use of Xpert MTB/Rif in previously treated TB cases 28](#_Toc335207520)

[4.2.1.3 Practical considerations for use of Xpert MTB/Rif 29](#_Toc335207521)

[4.2.1.4 Use of Xpert MTB/Rif results 29](#_Toc335207522)

[4.2.1.5 Repeat of Gene Xpert test 29](#_Toc335207523)

[4.2.2 Line Probe Assay (LPA) 30](#_Toc335207524)

[4.3 Conventional *M. TB* culture 30](#_Toc335207525)

[4.4 DST for first line anti-TB drugs 30](#_Toc335207526)

[4.5 DST for Second line drugs and XDR-TB diagnosis 30](#_Toc335207527)

[4.6 DST specimen collection 31](#_Toc335207528)

[4.7 The DR-TB diagnostic algorithm 31](#_Toc335207529)

[4.7.1 Diagnostic Algorithm for settings without Xpert MTB/Rif 32](#_Toc335207530)

[4.7.2 Diagnostic Algorithm for settings with Xpert MTB/Rif 33](#_Toc335207531)

[CHAPTER 5 TREATMENT OF DRUG RESISTANT TUBERCULOSIS 34](#_Toc335207532)

[5.1 Definitions of DR-TB treatment strategies 34](#_Toc335207533)

[5.2 Classes of anti-tuberculosis drugs 34](#_Toc335207534)

[5.3 Swaziland Standardized DR-TB Regimen design 36](#_Toc335207535)

[5.4 Duration of second-line anti-TB regimens 36](#_Toc335207536)

[5.5 Role of drug susceptibility testing 36](#_Toc335207537)

[5.6 Recommended Standardized treatment regimen 37](#_Toc335207538)

[5.7 Dosage of second line drugs 37](#_Toc335207539)

[5.7.1 Adult and Adolescent dosages of second line drugs 37](#_Toc335207540)

[5.8 Completion of the injectable agent (intensive phase) 38](#_Toc335207541)

[5.9 Treatment phases 38](#_Toc335207542)

[5.10 Treatment of XDR-TB 38](#_Toc335207543)

[5.11 Treatment of extra-pulmonary DR-TB 39](#_Toc335207544)

[5.12 Treatment of mono- and poly resistant TB 39](#_Toc335207545)

[5.12.1 Consequences for reporting 39](#_Toc335207546)

[5.12.2 Initiation of treatment for mono or poly resistant cases: 39](#_Toc335207547)

[5.12.2.1 Treatment options for mono or poly resistant cases: 40](#_Toc335207548)

[5.13 Role of Surgery in DR-TB treatment 40](#_Toc335207549)

[5.13.1 Timing of surgery 41](#_Toc335207550)

[5.13.2 Length of treatment after surgery 41](#_Toc335207551)

[5.14 Adjuvant therapy 41](#_Toc335207552)

[5.14.1 Nutritional support 41](#_Toc335207553)

[5.14.2 Corticosteroids 41](#_Toc335207554)

[5.15 Compassionate use of drugs in DR-TB Management 42](#_Toc335207555)

[5.15.1 General guide for compassionate use 42](#_Toc335207556)

[CHAPTER 6 TREATMENT OF DR-TB IN SPECIAL SITUATIONS 44](#_Toc335207557)

[6.1 Pregnancy 44](#_Toc335207558)

[6.2 Breastfeeding 44](#_Toc335207559)

[6.3 Contraception 45](#_Toc335207560)

[6.4 Diabetes mellitus 45](#_Toc335207561)

[6.5 Renal insufficiency 46](#_Toc335207562)

[6.5.1 Indications for Renal dialysis and how to access 47](#_Toc335207563)

[6.6 Liver disorders 48](#_Toc335207564)

[6.7 Seizure disorders 48](#_Toc335207565)

[6.8 Psychiatric disorders 49](#_Toc335207566)

[6.9 Substance dependence 49](#_Toc335207567)

[6.10 HIV-infected patients 50](#_Toc335207568)

[CHAPTER 7 MONITORING DR-TB TREATMENT AND MANAGEMENT OF ADVERSE EFFECTS 51](#_Toc335207569)

[7.1 Pre-treatment screening and evaluation 51](#_Toc335207570)

[7.2 Monitoring progress of treatment 52](#_Toc335207571)

[7.3 Follow-up bacteriological examinations (Microscopy and Culture) 52](#_Toc335207572)

[7.3.1 Culture conversion 52](#_Toc335207573)

[7.3.2 Sputum transportation for culture and DST 53](#_Toc335207574)

[7.4 Monitoring XDR-TB treatment 53](#_Toc335207575)

[7.5 Monitoring for adverse effects during treatment 53](#_Toc335207576)

[7.6 Management of adverse effects 55](#_Toc335207577)

[7.6.1 General approach 55](#_Toc335207578)

[7.6.2 Management of specific conditions 56](#_Toc335207579)

[7.6.2.1 Management of Nausea and Vomiting 56](#_Toc335207580)

[7.6.3 Management of Diarrhea 58](#_Toc335207581)

[7.6.3.1 Management of Gastritis 58](#_Toc335207582)

[7.6.3.2 Management of Hepatitis 59](#_Toc335207583)

[7.6.3.3 Management of Peripheral Neuropathy 59](#_Toc335207584)

[7.6.3.4 Management of Psychosis 60](#_Toc335207585)

[7.6.3.5 Management of seizures 61](#_Toc335207586)

[7.6.3.6 Hypothyroidism 62](#_Toc335207587)

[7.6.3.7 Management of myalgia and arthralgias 63](#_Toc335207588)

[7.6.3.8 Management of electrolyte abnormalities 64](#_Toc335207589)

[7.6.3.9 Management of nephrotoxicity 65](#_Toc335207590)

[7.6.3.10 Management of ototoxicity 65](#_Toc335207591)

[7.7 Management of irregular and noncompliant patient 69](#_Toc335207592)

[7.7.1 Abandoning or interrupting DR-TB treatment 69](#_Toc335207593)

[7.7.2 Re-initiation of treatment 69](#_Toc335207594)

[CHAPTER 8 MANAGEMENT OF CHILDHOOD MDR-TB 71](#_Toc335207595)

[8.1 MDR-TB in children 71](#_Toc335207596)

[8.1.1 MDR-TB case finding in children 71](#_Toc335207597)

[8.2 Diagnosis of MDR-TB in Children 72](#_Toc335207598)

[8.2.1 When to suspect DR TB in children 72](#_Toc335207599)

[8.3 Treatment of Paediatric MDR-TB 73](#_Toc335207600)

[8.3.1 Second line drugs for MDR-TB treatment in Children 74](#_Toc335207601)

[8.3.2 Adherence support for MDR-TB children 74](#_Toc335207602)

[8.3.3 Chemoprophylaxis 75](#_Toc335207603)

[8.3.4 Side effects 75](#_Toc335207604)

[8.3.5 Managing HIV/DR-TB co-infection in Children 75](#_Toc335207605)

[8.3.5.1 Provision of ART 75](#_Toc335207606)

[8.3.5.2 Reasons to postpone ART 75](#_Toc335207607)

[8.3.6 Recommended ART regimens for children on DR TB treatment: 76](#_Toc335207608)

[8.3.7 Alternative TB/HIV co-treatment ART regimen options for special situations: 76](#_Toc335207609)

[8.3.8 Provision of Cotrimoxazole 77](#_Toc335207610)

[CHAPTER 9 ORGANIZATION OF DR-TB TREATMENT DELIVERY AND COMMUNITY-BASED CARE 78](#_Toc335207611)

[9.1 Treatment delivery models 78](#_Toc335207612)

[9.1.1 Hospitalization: The National TB Hospital 78](#_Toc335207613)

[9.1.1.1 Referral of DR-TB patients 78](#_Toc335207614)

[9.1.1.2 Hospitalization 78](#_Toc335207615)

[9.1.1.3 Discharging hospitalized patients 79](#_Toc335207616)

[9.1.2 Ambulatory Care 80](#_Toc335207617)

[9.1.2.1 Clinic-based treatment: 80](#_Toc335207618)

[9.1.2.2 Community-based care 80](#_Toc335207619)

[9.2 Ensuring adherence to therapy 81](#_Toc335207620)

[9.2.1 Health education 81](#_Toc335207621)

[9.2.2 Directly observed therapy (DOT) 81](#_Toc335207622)

[9.2.2.1 Who should deliver DOT? 81](#_Toc335207623)

[9.2.2.2 Maintaining confidentiality: 81](#_Toc335207624)

[9.2.3 Socioeconomic interventions 81](#_Toc335207625)

[9.2.4 Psychosocial and emotional support 82](#_Toc335207626)

[9.2.5 Early and effective management of adverse drug effects 82](#_Toc335207627)

[9.2.6 Monitoring and follow-up of the non-adherent patient 82](#_Toc335207628)

[9.2.7 Community-based care and support 82](#_Toc335207629)

[9.2.8 Post-treatment follow-up of DR-TB cases 83](#_Toc335207630)

[CHAPTER 10 DRUG RESISTANT TB AND HIV 84](#_Toc335207631)

[10.1 General considerations 84](#_Toc335207632)

[10.2 Recommended collaborative activities 84](#_Toc335207633)

[10.3 Clinical features and diagnosis of DR-TB in HIV-infected patients 84](#_Toc335207634)

[10.4 Concomitant treatment of DR-TB and HIV 85](#_Toc335207635)

[10.4.1 Initiating ART treatment in patients with DR-TB 85](#_Toc335207636)

[10.4.2 DR-TB in patients already receiving ART 85](#_Toc335207637)

[10.4.3 Important drug–drug interactions in the treatment of HIV and DR-TB 86](#_Toc335207638)

[10.4.4 Potential drug toxicity in the treatment of HIV and DR-TB 86](#_Toc335207639)

[10.4.5 Monitoring of DR-TB and HIV therapy in co-infected patients 89](#_Toc335207640)

[10.4.6 Immune reconstitution inflammatory syndrome (IRIS) 90](#_Toc335207641)

[CHAPTER 11 MANAGEMENT OF DR-TB CONTACTS 91](#_Toc335207642)

[11.1 General considerations 91](#_Toc335207643)

[11.2 Contact investigation procedure 91](#_Toc335207644)

[11.3 Management of symptomatic adult contacts of a patient with MDR-TB 92](#_Toc335207645)

[11.4 Management of symptomatic Paediatric contacts of patients with MDR-TB 93](#_Toc335207646)

[11.5 Chemoprophylaxis of contacts of MDR-TB index cases 93](#_Toc335207647)

[CHAPTER 12 DR-TB AND INFECTION CONTROL 94](#_Toc335207648)

[12.1 When is TB infectious? 94](#_Toc335207649)

[12.2 The priorities of infection control 94](#_Toc335207650)

[12.3 Infection Control measures 94](#_Toc335207651)

[12.3.1 Administrative controls 94](#_Toc335207652)

[12.3.1.1 Infection Control plan 95](#_Toc335207653)

[12.3.2 Environmental controls 95](#_Toc335207654)

[12.3.3 Personal respiratory protection (special masks) 96](#_Toc335207655)

[12.4 High Risk areas 97](#_Toc335207656)

[12.5 Measures to reduce infection transmission in community settings where there is congregation 97](#_Toc335207657)

[CHAPTER 13 DR-TB MONITORING AND EVALUATION SYSTEM 98](#_Toc335207658)

[13.1 Aims of the information system and performance indicators 98](#_Toc335207659)

[13.2 Scope of the information system 101](#_Toc335207660)

[13.3 Main forms/registers and flow of information 101](#_Toc335207661)

[13.3.1 MDR-TB Treatment Card 101](#_Toc335207662)

[13.3.2 MDR-TB Register 102](#_Toc335207663)

[13.3.3 Request for sputum examination 103](#_Toc335207664)

[13.3.4 Laboratory Register for culture and DST 103](#_Toc335207665)

[13.3.5 Quarterly report on MDR-TB detection and DR-TB treatment 103](#_Toc335207666)

[13.3.6 Six-month interim outcome assessment of confirmed MDR-TB case (Form 11) 103](#_Toc335207667)

[13.3.7 Annual report of treatment result of confirmed MDR-TB patients starting DR-TB treatment 104](#_Toc335207668)

[ANNEXES 105](#_Toc335207669)

[13.4 Algorithm for management of depression: 105](#_Toc335207670)

[13.5 Algorithm for management of Nausea and vomiting: 106](#_Toc335207671)

[13.6 Algorithm for management of Hepatitis: 107](#_Toc335207672)

[13.7 Algorithm for management of Hypothyroidism: 108](#_Toc335207673)

[13.8 Algorithm for management of Nephrotoxicity: 109](#_Toc335207674)

[13.9 Algorithm for management of Psychosis: 110](#_Toc335207675)

[13.10 Algorithm for management of Hypolalaemia: 111](#_Toc335207676)

[13.11 Algorithm for management of Haemoptysis: 112](#_Toc335207677)

[13.12 Algorithm for management of Haemoptysis -2: 113](#_Toc335207678)

[13.13 Algorithm for management of respiratory insufficiency: 114](#_Toc335207679)

[REFERENCES 117](#_Toc335207680)

**List of tables:**

[Table 1.2: Resistance to first line and second anti-tuberculosis drugs in Swaziland from the Swaziland XDR-TB rapid survey, July-August 2007 15](#_Toc335059977)

[Table 1.3: Pattern of resistance to first line anti-TB drugs by previous treatment history in Swaziland 2009 16](#_Toc335059978)

# PREFACE

The emergence of resistance to anti-tuberculosis drugs, and particularly of multidrug-resistant TB (MDR-TB) in Swaziland brought to our realization the fact that we are not only confronted with a major public health threat, but also a formidable an obstacle to bringing the TB epidemic under control. The unacceptably high HIV prevalence further threatens to make an already dire situation even worse. Most importantly, the situation if not timely and adequately responded to could jeopardize the chances of the country meeting the Millennium Development Goals for TB as well as the Stop TB Partnership targets for 2015. In addition, the country remains focused on realizing the goal of universal access to high quality TB, TB/HIV and MDR-TB interventions in line with the 2006 AU declarations and the 2009 Beijing Call for action on DR-TB.

Recognizing that the main reasons behind the emergence of anti-TB drug resistance are linked inadequate basic TB control systems, poor management of the supply and quality of anti-tuberculosis drugs, improper TB case management; and transmission of the disease in congregate settings, the ministry of health decided to take a bold step to strengthen our National TB response through full implementation of the WHO recommended Programmatic Management of Drug-resistant TB (PMDT).

The rapidly changing terrain in the management of drug resistant TB since WHO published the first global guidelines in 2006 requires our national TB control programme to respond with novel and effective responses grounded in the most recent evidence-based recommendations, and hence the need for this updated guideline. This guideline therefore represents one of the essential tools in response to the 62^nd^World Health Assembly’s (WHA) call for Member States to develop a comprehensive framework for the management and care of multidrug-resistant tuberculosis (MDR-TB) and extensively drug-resistant tuberculosis (XDR-TB). The guidelines takes into consideration to need ensure access to culture and drug susceptibility testing to all patients in whom DR-TB is considered likely; the globally collated experience in treating DR-TB as well as DR-TB management in the context of high HIV prevalence.

It is also gratifying to note that the guidelines have been structured around a flexible framework, combining a consistent core of principles based on current WHO recommendations; but also directly addressing our local health systems context including the community and household levels.

The ministry of health will continue to ensure that beyond the development of such DR-TB management tools, essential requirements like supply of quality-assured second line anti-TB drugs will continue to be pursued. To this end, the ministry will continue to optimize the opportunity to access concessionally-priced, quality-assured second-line anti-tuberculosis drugs through the WHO Green Light Committee (GLC).

May I congratulate the National TB Programme Programme and its partners for the quality of work in the revision of this guideline, which we expect will be used by all concerned in the management of DR-TB in the country.

**Minister of Health**

# FOREWORD

According to the recent World Health Organization (WHO) Global TB Report of 2011, the incidence rate of TB in Swaziland remains the highest in the world. The TB programme faces problems of poor diagnosis of cases, poor case holding and high rates of loss to follow up.

In recent times, WHO has expressed concern over the emergence of TB strains that are resistant to first line anti TB drugs (MDR TB) and even second line drugs (XDR-TB) and is calling for measures to be strengthened and implemented to prevent the global spread of these deadly TB strains. This follows research showing the extent of XDR-TB, a newly identified TB threat which leaves patients (including many people living with HIV) virtually untreatable using currently available anti-TB drugs. XDR-TB poses a grave public health threat, especially in populations with high rates of HIV and where there are few health care resources.

Swaziland started implementing an ambitious 5 year plan to combat TB, a major public health problem that was declared an emergency in the SADC region in 2006. As Swaziland makes frantic efforts to identify additional financial resources for TB, there are efforts to strengthen health systems in the country.

The country has focused on improving the quantity and quality of staff involved in TB control; increasing TB case detection and treatment success rates with expanded DOTS coverage at national and lower levels; reducing the combined TB patient loss to follow up and transfer out rates; scaling up access to counseling and testing for HIV among TB patient; and scaling up interventions to manage TB and HIV together, including increased access to anti-retroviral therapy for TB patients who are co-infected with HIV. Efforts will also be directed towards strengthening collaboration with partners through expanding national TB partnerships, public-private collaboration and community participation in TB control activities.

At the centre of the strategy will be strengthening basic TB care to prevent the emergence of drug-resistance; ensuring prompt diagnosis and treatment of drug resistant cases to cure existing cases and prevent further transmission; and increasing investment in laboratory infrastructures to enable better detection and management of resistant cases.

The outbreak of XDR-TB in the KwaZulu-Natal province of South Africa made it imperative for the Swaziland National Tuberculosis Control Programme to devise plans to respond to the increasing threat of MDR and XDR-TB because Swaziland shares its borders with KwaZulu- Natal. Subsequently a short term plan was developed to address the immediate priorities in order to limit the negative impact of drug-resistant TB. In developing the plan, the NTCP did analyze the issues affecting MDR identification and management through a process of problem identification and analysis.

**Dr. Simon Zwane**

**Director of Health Services**

# ACKNOWLEDGEMENT

The Ministry of Health appreciates the efforts of the Writing Group particularly the team responsible for developing this document. We congratulate the National Tuberculosis Control Programme and the HIV and AIDS Program for taking a leading role in the strategic development process, which will hopefully guarantee sustainability of our programmes and interventions.

The contributions of the following editing team members are specially acknowledged:

**National TB Control Programme :** Mr. Themba Dlamini, Dr. Mohammed Kamal, Dr. Charity Newton, Dr. Fikao Woreta, Mr. Africa Mnisi, Mrs. Nonhlanhla Dlamini, Mrs. Gugu Mchunu, Mrs. Philile Mzebele, Mrs. Siphiwe Khumalo, Mrs. Lindiwe Mduli; Mr. Thabo Kunene, Mr. Ernest, Mr. Sandile Ginindza, Dr Velephi Okello, Ms. Prudence Gwebu, Dr Marianne Calnan, Doreen Dlamini, Mr. Blessing Marondera, Ms. Sindisiwe Dlamini, Mrs. Gugu Maphalala and Mr. Derrick Khumalo.

**World Health Organization:** Dr. Kefas Samson, Dr. Augustine Ntilivamunda; Dr. Benjamin Gama, Dr. Hind Satti (WHO Consultant).

**University Research Co., LLC:** Dr. Samson Haumba, Dr. Yohannes Ghebreyesus, Ms Cindy Dladla,

**PEPFAR:** Dr. Peter Ehrenkranz

**Medicins Sans Frontieres (MSF):** Dr Hyak Korakozoin, Mr. Kees Keus; Dr. Benjamin Mwangombe

**Baylor College of Medicine:** Dr. Piluca Pustero

**Acknowledgement of funding:** Printed by WHO through the WHO/Italian Initiative.

**Technical Assistance coordinated through:** The World Health Organization Country office and Afro-Regional office.

**Dr. Steven V. Shongwe**

**Principal Secretary, MOH****GLOSSARY AND ABBREVIATIONS**

| AFB | Acid-fast bacilli |
| --- | --- |
| Am | Amikacin |
| Amx/Clv | Amoxicillin/ClavulanateLzd Linezolid |
| ART | Anti-retroviral therapy |
| CDC | United States Centers for Disease Control and Prevention |
| Cfx | Ciprofloxacin |
| Cfz | Clofazimine |
| Clr | Clarithromycin |
| Cm | Capreomycin |
| CPT | Cotrimoxazole Preventive Therapy |
| Cs | Cycloserine |
| DOT | Directly Observed Therapy |
| DOTS | Directly Observed Treatment Short Course |
| DRS | Drug Resistance Survey |
| DST | Drug Susceptibility Testing |
| E | Ethambutol |
| Eto | Ethionamide |
| GFATM | Global Fund to Fight AIDS, Tuberculosis and Malaria |
| Gfx | Gatifloxacin |
| GLC | Green Light Committee |
| H | Isoniazid |
| HIV | Human Immuno-deficiency Virus |
| HPF | High-power field |
| Km | Kanamycin |
| LFT | Liver Function Test |
| Lfx | Levofloxacin |
| MDR-TB | Multidrug-resistant Tuberculosis |
| Mfx | Moxifloxacin |
| NTM | Non-tuberculous mycobacterium |
| Ofx | Ofloxacin |
| PAS | Para-amino salicylic acid |
| PPD | Purified Protein Derivative |
| Pto | Protionamide |
| R | Rifampicin |
| S | Streptomycin |
| SCC | Short-course Chemotherapy |
| TB | Tuberculosis |
| Th | Thioacetazone |
| Trd | Terizidone |
| TSH | Thyroid-stimulating Hormone |
| URC | University Research Corporation |
| UVGI | Ultra-violet Germicidal Irradiation |
| WHO | World Health Organization |
| Z | Pyrazinamide |

EXECUTIVE SUMMARY

## Purpose of the guidelines

Effective management of drug-resistant tuberculosis requires a set of coordinated activities by different components or units of the health care delivery system under the national TB control programme. The components of a sound DR-TB management system include case detection, treatment, prevention, surveillance, and monitoring and evaluation of the programme’s performance, which are collectively referred to as the “programmatic management of drug-resistant tuberculosis” (PMDT).

These guidelines offer updated recommendations regarding all these components to ensure successful implementation of PMDT in the country. It is therefore designed for TB control programme staff, health care workers who are faced with the responsibilities of clinical management MDR-TB and other drug-resistant forms of TB or programmatic coordination to ensure universal access to quality services. This second edition replaces the 2009 first edition, and takes into account important developments in TB diagnostics and recent evidence on results of DR-TB therapy.

## Target audience

The target audience of the guidelines is staff and medical practitioners working in treatment and control of TB, partners implementing programmatic management of drug-resistant TB, and organizations providing technical and financial support for care of drug-resistant TB.

## Scope

The guidelines cover topics relating to DR-TB case finding and treatment strategies for both adults and Children; laboratory aspects; surveillance, management of DR-TB/HIV co-morbidity; and infection prevention and control. It does not however address the details of basic DOTS, laboratory activities and infection control as specific guidelines and Standard Operating Procedures for these already exists, but ensures adequate linkage between them.

## Justification for guideline update

The first MDR-TB management guidelines developed by the National TB Control Programme provided guidance based on the results of a rapid assessment of the DR-TB situation in the country conducted in 2007 and the best available evidence from DOTS-Plus projects reflected in the WHO guidelines for Programmatic Management of Drug resistant TB – 2008 Emergency update. Since then, the NTCP conducted a country-wide Drug Resistance Survey (DRS), which enhance the understanding of the resistance pattern and implemented a GLC project in collaboration with partner e.g MSF. The experiences of this project, and the 2011 WHO review of current evidence reflected in the 2011 update provided new insights into MDR-TB management.

This necessitated the review of the Swaziland first edition of the MDR-TB guideline in line with current realities and international recommendations.

## Highlights of the new revision

This guideline consists of and Executive summary and twelve chapters divided into two main sections namely: Section 1 which includes Chapters 1 addressing the general introduction to the programme context and Chapter 2 which dealt with the strategic approaches for DR-TB management and coordination in the country. The Section 2 consists of chapters 3 to 13 i.e 11 Chapters addressing mainly different components of DR-TB clinical management as well as surveillance, monitoring and evaluation system. The guideline also has annexes where additional guidance through tables and algorithms are provided for specific issues.

The various coordination mechanisms established under the National TB Control Programme for effective DR-TB management, which covers coordination between health facilities, referrals, implementation partners, private care providers, institutions with congregate settings and with the community levels described in Chapter 2.

Chapter 3 and 4 outlines the DR-TB Case definitions; registration, bacteriology and treatment outcomes based on the recommendations in the WHO 2011 update to the Guidelines on Programmatic Management of DR-TB. Key among the new updates is the revision of DR-TB case definitions based on the newly approved WHO recommended diagnostics (WRDs). The duration of MDR-TB treatment is also revised to total of 20 months consisting of 8 months of intensive and 12 months of continuation phases.

DR-TB Case finding Strategies, diagnosis and treatment strategies have been updated in chapters 4 to 8 including the use of rapid DST methods to guide initial treatment regimen with particular reference to molecular techniques (Xpert MTB/Rif and Line Probe Assay) as well later adjustments based on full conventional DST results. The nationally agreed diagnostic algorithms for DR-TB detection and treatment based on availability of rapid diagnostic tests to guide health care facilities are also included.

This version also has an updated section on Management of Childhood MDR-TB covered in chapter 8, while chapter 9 dealt with the current agreed models for DR-TB treatment delivery from health facility to community and household levels.

Chapter 10 provides updated guide to co-management of Drug resistant TB and HIV which includes ART regimen selection, timing of ART initiation and addressing possible overlapping toxicities including the Immune Reconstitution syndrome (IRIS).

Systematic investigation and management of DR-TB contacts is dealt with in Chapter 11 for both adult and adults and child contacts, while Chapter 12 addressed DR-TB and Infection Control in the context of interventions and measures already elaborated in the National TB Infection Control guidelines.

Chapter 13 outlines dealt with the DR-TB monitoring and evaluation system, which is also updated based on the revised DR-TB case definitions, definitions of treatment outcomes and the WHO minimum set of monitoring indicators.

Finally, this edition includes annexes that contain key essential information in the management of DR-TB, which includes specific algorithms for clinical management of some of the most critical adverse effects often experienced by the patients.

**PART 1:**

**MANAGEMENT OF DRUG RESISTANT TUBERCULOSIS:**

**MANAGEMENT AND COORDINATION**

1. BACKGROUND INFORMATION

## Magnitude of the DR-TB problem

The incidence of drug resistance has increased since the first drug treatment for TB was introduced in 1943. The emergence of MDR-TB following the widespread use of Rifampicin beginning in the 1970s led to the use of second-line drugs. Improper use of these drugs has fuelled the generation and subsequent transmission of highly resistant strains of TB termed extensively DR-TB, or XDR-TB. These strains are resistant to at least one of the fluoroquinolone drugs as well as a second line injectable agent, in addition to isoniazid and rifampicin.

The most recent global estimates indicate about 489,000 cases of MDR-TB in 2006 with 116,000 deaths, unevenly spread among 27 countries (of which 15 are in Eastern Europe) accounting for 86% of the total. By 2007, the WHO collects data from 117 countries covering areas that contain more than 50% of global smear-positive TB cases. Overall about 4.8% of all TB cases are said to have drug resistant strains. With an estimated 66,711 MDR-TB cases in 2006, Africa accounts for 13.6% of the global case load; and about 2.2% of all TB cases are multi-drug resistant (WHO 4^th^ Global Report n DR-TB, 2008). WHO also estimates about 27,000 cases of Extensively Drug Resistant tuberculosis globally with about 16,000 deaths.

## DR-TB in Swaziland

Tuberculosis remains a major public health challenge in the kingdom of Swaziland with an estimated 20% of institutional deaths attributable to the disease. With an estimated TB incidence of 1,287 per every 100,000 of its population, Swaziland has the highest TB incidence in the world. Compared to a 1990 level of 267 new cases (all forms) per 100,000 population per year, TB incidence had increased six-fold, by 2010, while incidence of the infectious cases (sputum smear positive pulmonary TB cases) tripled in the same period.

Similarly, the TB prevalence has doubled from a 1990 level of 350 per 100,000 population to 704 per 100,000 population in 2010, while the TB mortality (excluding HIV based on revised ICD-10 classification) remains rather stable at 32 per 100,000 population compared to 31 in 1990. Swaziland also has a very high HIV prevalence of 26% among the 15-49 years age group; thereby resulting in a significantly high HIV-TB co-infection rate. Currently, the prevalence of HIV among incident tuberculosis patients was 82% (2010 data).

Drug resistant tuberculosis also constitutes a major threat to the national TB Control efforts. A country-wide drug resistance survey among TB patients conducted in 2009/2010revealed 7.7% of MDR among new cases of TB, and 33.9% among previously treated cases, which are significantly high relative to the neighbouring countries. Cumulatively 503 MDR-TB cases have been reported from 2006 to 2009.

The laboratory capacity to diagnose and manage drug resistant tuberculosis has been improved with the increased MGIT capacity at the NRL and recent introduction of Gene Xpert and Line Probe Assay genotypic/molecular methods.

## Causes of DR-TB

TB drug resistance is said to be present if growth of *M. tuberculosis* isolates is observed in spite of the presence of anti-TB drugs. Although its causes could be microbial, clinical or programmatic, DR-TB is essentially a man-made phenomenon. From a microbiological perspective, resistance is caused by a genetic mutation that makes a drug ineffective against the mutant bacilli. From a clinical and programmatic perspective, it is an inadequate or poorly administered treatment regimen that allows a drug-resistant strain to become the dominant strain in a patient infected with TB. **Table 1.1** below summarizes the common causes of inadequate treatment.

Short-course chemotherapy (SCC) for patients infected with drug-resistant strains may create even more resistance to the drugs in use. This has been termed the “amplifier effect” of SCC. Ongoing transmission of established drug-resistant strains in a population is also a significant source of new DR cases.

Table 1.1: Causes of inadequate anti-tuberculosis treatment*

| **Health-care providers: inadequate regimens**   - Inappropriate guidelines or non-compliance with guidelines - Absence of guidelines - Poor training - No monitoring of treatment - Poorly organized or funded TB control programmes | **Drugs: inadequate supply or quality**   - Poor quality - Unavailability of certain drugs (stock-outs or delivery disruptions) - Poor storage conditions - Wrong dose or combination of drugs | **Patients: inadequate drug intake**   - Poor adherence (or poor DOT) - Lack of information on treatment , - Adverse effects of treatment; - Social barriers (stigma, restrictions) - Malabsorption due to other causes - Substance dependency disorders - Mental disorder; - Non-cooperative |
| --- | --- | --- |

* *Source: Adapted from WHO guidelines for programmatic management of drug resistant tuberculosis. Emergency update 2008. WHO/HTM/TB/2008.402*

A rapid survey of MDR/XDR among high risk groups in Swaziland conducted in July-Aug 2007 revealed significant levels of resistance to first line and second line drugs in the country as shown in **Table 1.2** below:

Table 1.2: Resistance to first line and second anti-tuberculosis drugs in Swaziland from the Swaziland XDR-TB rapid survey, July-August 2007

| **Drug** | **Number Isolates tested (Denominator)** | **Resistant Strains** | | **Sensitive Strains** | |
| --- | --- | --- | --- | --- | --- |
|  |  | **Frequency** | **Percentage** | **Frequency** | Percentage |
| **Streptomycin** | 116 | 87 | 75.0 | 29 | 25.0 |
| **Isoniazid** | 115 | 96 | 83.5 | 19 | 16.5 |
| **Ethambutol** | 115 | 77 | 67.0 | 38 | 33.0 |
| **Pyrazinamide** | 89 | 63 | 70.8 | 26 | 29.2 |
| **Rifampicin** | 115 | 55 | 47.8 | 60 | 52.2 |
| **Amikacin** | 111 | 12 | 10.8 | 99 | 89.2 |
| **Kanamycin** | 111 | 9 | 3.5 | 102 | 91.9 |
| **Capreomycin**  **(lower dose)** | 111 | 23 | 20.7 | 88 | 79.3 |
| **Capreomycin**  **(higher dose)** | 111 | 15 | 13.5 | 96 | 86.5 |
| **Ofloxacin**  **(lower dose)** | 111 | 14 | 12.6 | 97 | 87.4 |
| **Ofloxacin**  **(higher dose)** | 111 | 9 | 3.5 | 102 | 91.9 |
| Ethionamide | 111 | 43 | 38.7 | 68 | 61.3 |

*Source: Report of Rapid assessment of MDR-TB in Swaziland July to August 2007*

An MDR-TB prevalence of 18.5% among TB patients receiving Category II anti-TB treatment was was also seen. However, there were not accurate recent national figures on the extent of DR-TB among general TB patients. Moreover several factors as the proximity to the province of Kwazulu Natal in South Africa, where an outbreak of XDR-TB was reported in 2005 among HIV co-infected cases with very high fatality rate (7), and the difficulties faced by the National TB Program to ensure adequate TB case management (58% success rate for new smear positive cases and 37% for retreatment cases in 2007) suggested a high prevalence of DR-TB.

In 2009-2010, a country-wide DR survey was conducted with collaborative efforts of MOH, MSF, WHO and URC to measure the prevalence of MDR-TB in pulmonary tuberculosis smear positive patients, both new cases (NC) and previously treated cases (PTC) in Swaziland. This survey revealed a much higher levels of MDR-TB in the country. According to the survey results, 7.7% of new cases and 33.8% of previously treated cases have MDR-TB as summarized in table 1.3 below:

Table 1.3: Pattern of resistance to first line anti-TB drugs by previous treatment history in Swaziland 2009

| **Resistance pattern** | **New Cases** | | **Previously treated** | | **TOTAL** | |
| --- | --- | --- | --- | --- | --- | --- |
|  | **Number** | **%** | **Number** | **%** | **Number** | **%** |
|  |  |  |  |  |  |  |
| Susceptible to all | 298 | 84.7 | 142 | 50.5 | 440 | 69.5 |
|  |  |  |  |  |  |  |
| Any Resistance | 54 | 15.3 | 139 | 49.5 | 193 | 30.5 |
| INH | 47 | 13.4 | 127 | 45.2 | 174 | 27.5 |
| Rmp | 28 | 8 | 102 | 36.3 | 130 | 20.5 |
| Emb | 29 | 8.2 | 94 | 33.5 | 123 | 19.4 |
| Sm | 47 | 13.4 | 115 | 40.9 | 162 | 25.6 |
| Mon-resistance | 12 | 3.4 | 24 | 8.6 | 36 | 5.7 |
| INH | 5 | 1.4 | 12 | 4.3 | 17 | 2.7 |
| Rmp | 1 | 0.3 | 7 | 2.5 | 8 | 1.3 |
| Emb | 0 | 0 | 0 | 0 | 0 | 0 |
| Sm | 6 | 1.7 | 5 | 1.8 | 11 | 1.7 |
| MDR | 27 | 7.7 | 95 | 33.8 | 122 | 19.3 |
| INH + Rmp | 1 | 0.3 | 3 | 1.1 | 4 | 0.6 |
| INH + Rmp + Emb | 0 | 0 | 2 | 0.7 | 2 | 0.3 |
| INH + Rmp + Sm | 6 | 1.7 | 12 | 4.3 | 18 | 2.8 |
| INH + Rmp + Sm + Emb | 20 | 5.7 | 78 | 27.8 | 98 | 15.5 |
| Other FLD resistance pattern other than MDR | 15 | 4.3 | 20 | 7.1 | 35 | 5.5 |
| INH + Sm | 6 | 1.7 | 6 | 2.1 | 12 | 1.9 |
| INH + Sm + Emb | 9 | 2.6 | 14 | 5 | 23 | 3.6 |

*Source: Report of National DR-TB Survey in Swaziland June 2009 to January 2010*

The second line drug resistance pattern indicate 42% overall resistance to Etionamide, 8.2% to Ofloxacin, 2.5% to Capreomycin and 1.6% to Amikacin.

1. FRAMEWORK FOR DR-TB CONTROL IN SWAZILAND

## The strategic approach

Management of DR-TB in Swaziland will be an integral part of the NTP in such a way that ensures universal free access to MDR/XDR-TB treatment to all patients in need, including those diagnosed in the private sector through PPM approaches.

The primary objective of the National TB Control Programme is to pursue the expansion and enhancement of high quality DOTS and deliver basic TB treatment under proper case management conditions to prevent the emergence of resistance to second-line drugs.

The framework for ensuring effective management of drug-resistant TB in the country is based on the 6 components of the Stop TB Strategy as follows:

1. **Pursue high quality DOTS expansion and enhancement**

This aims to ensure quality basic DOTS implementation to prevent the emergence of MDR-TB through:

1. **Sustained political commitment:** Demonstrated through increased and sustained funding for TB control including Programmatic Management of Drug Resistant Tuberculosis (PMDT). This should cover human resources, adequate supply of first and second line anti-TB drugs, laboratory equipment and supplies, training, legal and regulatory documents, infrastructure and coordination of all stakeholders involved in all aspects of the framework for control of DR-TB.
2. **Appropriate case finding and diagnosis through quality-assured bacteriology:** The program aims to rapid diagnosis of DR-TB to enable provision of timely and effective treatment. This includes the adoption of appropriate diagnostic algorithm based on rapid molecular methods including the Xpert MTB/Rif for rifampicin resistance; and Line Probe Assay (LPA) for both rifampicin and isoniazid resistance; as well as increased capacity for conventional DST using the Mycobacterium Growth Indicator Tube (MGIT). Internal quality control (QC) and external quality assurance (EQA) would be maintained through collaboration with a WHO-recognized Supra-national Reference Laboratory (SNRL).
3. **Appropriate strategies that ensure treatment with second line drugs under proper case management conditions:** This refers to the provision of timely and effective treatment to all DR-TB cases based on bacteriological confirmation. It includes provision of appropriate DR-TB treatment regimens, development of adequate capacity and trained staff at the health facilities for DOT, adequate patient support measures, as well as timely identification and management adverse effects. It also includes establishment of a council of experts or steering committee to review treatment decisions and specific clinical situations. In addition, measures to improve patient adherence such as support group, psychological counseling, transportation subsidy, food packages, health education and stigma reduction.
4. **Uninterrupted supply of quality-assured second-line anti-tuberculosis drugs:** A sound Procurement and supply management system for quality-assured second line anti-TB drugs to obtained from WHO prequalified sources, or sources meeting WHO Good Manufacturing Practice (GMP) standards.
5. **Recording and reporting system for effective monitoring of DR-TB control:** This is based on the WHO minimum set of indicators for monitoring and evaluation of DR-TB programmes (2010). The program will ensure establishment of a sound and sensitive DR-TB surveillance system using standardized recording and reporting materials including appropriate analysis of interim and final treatment outcomes as recommended by WHO. This also includes periodic Drug Resistance Surveys (DRS) and development of electronic medical records (EMR) for MDR-TB management.
6. **Address TB/HIV and vulnerable populations:**

Implementing collaborative TB/HIV activities, preventing and controlling DR-TB, including XDR-TB, and addressing prisoners, miners, ex-miners, and other high-risk groups and situations.

1. **Contributing to Health Systems Strengthening**

By collaborating with other health-care programmes and departments e.g by mobilizing the necessary human and financial resources for strengthening laboratory, pharmaceutical and radiological services, strengthening infection control in health care facilities and correctional facilities. Also contributing to strengthening information management system.

1. **Engage all care providers in MDR-TB management**

By scaling up public–private mix (PPM) approaches for MDR-TB to ensure adherence to international standards of TB care and national DR-TB management guideline with special focus on universal access to quality DR-TB diagnosis, treatment and care.

1. **Empower patients and communities on TB including TB/HIV and MDR-TB**

By scaling up community TB care and creating demand through culturally appropriate advocacy, communication and social mobilization.

1. **Enable and promote research**

This includes programme-based operational research aimed at generating evidence that will improve DR-TB management.

## Coordination

Successful management of Drug-resistant TB requires adequate coordination of the efforts and contributions of all the key stakeholders, organizations and external partners. The responsibility for overall coordination of the DR-TB control lies with the National TB Programme. The coordination function is discharged through a designated MDR/XDR-TB focal point in NTCP. The various elements of the coordination are elaborated below.

### Coordination of stakeholder efforts:

All key stakeholders should operate within the framework of the National guidelines for programmatic Management of Drug-resistant TB in Swaziland. The underlying principle is ensuring standardization of DR-TB management throughout the country in terms of:

- standardization of diagnostic, treatment and care delivery procedures;
- ensuring coordinated procurement and management of second line drugs through the Green Light (GLC) Mechanism;
- ensuring one MDR/XDR-TB surveillance, monitoring and evaluation system into which every partner buys in.

### Coordination of case management between TB Diagnostic health Units and the National TB Hospital

Detection and treatment of all forms of TB, including drug-resistant forms, should integrated within the basic DOTS activities of all TB basic management units that manage drug-susceptible TB.

Untreated or improperly treated patients with DR-TB are a source of ongoing transmission of resistant strains increased morbidity and mortality.

The framework for the management of DR-TB should ensure adequate coordination of patient’s transfer system from hospitals to other out-patient settings and vice versa. Transfers should be carefully planned with advance planning and adequate communication between referral units.

Given the type of care required during the treatment of DR-TB patients, a team of health workers including physicians, nurses and social workers should be constituted to provide support and supervision of case management in peripheral units managing DR-TB cases.

The roles and responsibilities of the various levels are as follows:

**National TB Hospital**

- Admission (Criteria is on page 46)
- Centralized registration of MDR/XDR-TB patients (now we have register in every HC. The registration is not more centralized)
- Initiation of treatment
- Management of critically ill and patients with severe side effects
- Follow up
- Support supervision of TB diagnostic units and clinics
- Review of complicated and difficult cases
- Setting standards for MDR clinical management
- Assist in facilitating transportation of patients to lower levels of care when required, and subject to availability of vehicle;
- Ensuring community activities for MDR-TB
- SLD management and drugs for side effects

**The Central MDR/XDR-TB Clinical team**

The Central pool of experts will constitute the MDR/XDR-TB clinical team under the National TB Hospital. The main functions of this team shall be as follows:

- Provide technical support to the MDR-TB management teams at the various TB diagnostic sites;
- Advise on management of mono- and poly-drug resistant TB cases;
- Assist monthly reviews of patients at the TB diagnostic sites;
- Assist TB diagnostic sites in decisions regarding management of difficult to treat cases, side effects, and adverse drug reactions;
- Assist in maintaining up to date Register of all DR-TB cases country-wide.

**National Reference laboratory (NRL)**

The national Reference Laboratory has a critical role in the management of DR-TB cases with respect to diagnosis and follow-up through quality-assured bacteriological examinations. The NRL main functions are as follows:

- Assist the National TB Programe in general organization of TB laboratory services and network;
- Provide and coordinate Quality Assurance system for sputum smear microscopy for laboratories in all TB diagnostic sites;;
- Perform mycobacterial culture and Drug Susceptibility testing (DST)
- Training of microscopists or phlebotomists;
- Assist NTP in quantification of laboratory inputs (equipment, reagents and consumables);
- Assist in maintaining an effective TB surveillance system;
- Liaise with a WHO-accredited Supra-national reference laboratory for second line DST and External Quality Assurance;
- Assist the NTCP in organizing and conducting operational research;

**TB Diagnostic Units**

- Identification of DR-TB suspects
- Request for culture and DST for DR-TB suspects
- Refer confirmed MDR-TB patients for treatment initiation and MDR-TB suspects with multiple co-morbidity for management
- Initiate MDR-TB treatment where there is capacity (MO trained in MDR-TB management, follow guidelines for MDR-TB management, availability of an isolation facility to admit patients for at least 1 month
- Provide transport for MDR-TB patients and difficult suspects to TB hospital on prior arrangement with TB Hospital
- Manage Mono and Poly DR-TB cases
- Provision of DOTS plus to all patients including injections
- Proper management of reporting and recording system and notifying the central unit for registration
- Manage and dispense SLD and receive monthly supply per patient from the central level
- Collaboration with community HCWs and NGOs involved in MDR activities e.g. Contact investigation; tracing of patients who are lost to follow up; adherence support; and food supplement provision.

**Primary Health Centers/ TB Treatment Units**

- Identify and DR suspects
- Collect sputum samples for culture/DST or refer to the TB diagnostic Units or TB Hospital for investigation;
- Provide DOT including administration of injectable medications;
- Refer MDR-TB patients to TB diagnostic units for review
- Facilitate transportation of DR-TB patients for treatment follow up checks at MDR-TB management centres;
- Facilitation of DR-TB patient referrals for serious conditions and complications.

### Coordination at Community level care:

The NTCP should establish collaboration with communities to help address the interim needs of patients, including the provision of DOT, food and/or housing.

Community health workers play a crucial role in the provision of ambulatory care to DR-TB patients.

NTCP should ensure CHWs are properly selected, trained and screened for HIV and TB. Community leaders usually serve as entry points to enlist support of communities for TB care including those with drug resistance. This can be achieved through:

- Community education, involvement and organization around TB issues;
- Activities to foster community ownership of control programmes and stigma reduction.

### Coordination with prisons and other congregate and Institutional organizations

Transmission in prisons is an important source of spread of DR-TB, thereby requiring adequate infection control measures to be put in place to reduce incidence.

A strong collaboration between NTP and prison authorities will ensure that correctional service institutions are oriented in TB, and ensure that arrangements are in place to:

- screen new prisoners;
- conduct regular evaluation of inmates;
- ensure implementation of infection; and
- put in place mechanisms to ensure treatment adherence.

An arrangement should be made for inmates receiving treatment who are due to be released from prison to ensure that they complete their treatment. This can be achieved through:

- using specific procedures for transferring care after release from prison;
- effective communication between Prison authorities and NTP on discharge inmates;
- advance planning,
- targeted social and psychological support

Similar collaboration should be established and maintained with mental health and orphanage institutions with the view to ensure regular screening of persons at risk, provision treatment where indicated, and ensuring infection control.

### Coordination of all health-care providers (both public and private)

Private practitioners should be considered partners in managing tuberculosis including drug-resistant cases. Therefore collaboration with the private providers will be strengthened to ensure their involvement in the design and technical aspects of the programme for an effective and mutually beneficial cooperation. Private providers should follow the standard diagnostic and treatment protocols of the programme and report according to the national guidelines. The programme on the hand should support the private sector with quality-assured second line anti-TB drugs, technical support and training. Regardless of where patients are treated, access to treatment should be free.

### Coordination with the International partners

International technical support through WHO, the GLC secretariat - Geneva, SRLs, and other partners e.g. MSF, the University Research co., LLC, and Italian Cooperation will be made use of in the implementation of Drug Resistant TB management. The NTP will set up an MDR-TB Clinical Expert Committee that ensures clear division of tasks and responsibilities.

**PART 2: DRUG RESISTANCE TB MANAGEMENT**

1. DEFINITIONS, CASE REGISTRATION, BACTERIOLOGY AND TREATMENT OUTCOMES

## Definitions of drug resistance

Drug-resistant tuberculosis is **confirmed** through laboratory tests that demonstrates growth in-vitro of infecting isolates of *Mycobacterium tuberculosis* in the presence of one or more anti-tuberculosis drugs.

By definition, there are four different categories of drug resistance namely:

- Mono-resistance: resistance to one anti-tuberculosis drug.
- Poly-resistance: resistance to more than one anti-tuberculosis drug, other than both isoniazid and rifampicin.
- Rifampicin Resistance: resistance to Rifampicin only without established resistance against INH.
- Multi-drug-resistance: Mycobacterium tuberculosis complex isolates with in vitro resistance against isoniazid and rifampicin, with or without resistance to additional first and second-line anti-TB drugs.
- Extensive drug-resistance: resistance to any fluoroquinolone, and at least one of three injectable second-line drugs (capreomycin, kanamycin and amikacin), in addition to multidrug-resistance.

## DR-TB case registration groups

For the purpose of case registration, the following groups will apply:

- Suspected MDR-TB: This group includes any person suspected to be harbouring multi-drug resistant tuberculosis. These includes all previously treated ( failed treatment, return after treatment interruption or relapses) and any new or previously treated on anti-tuberculosis treatment who fail to convert after 2 months of treatment;. Contacts of MDR-TB patients should also be suspected of having MDR-TB. Such cases should be recorded in the MDR-TB suspect register for rapid investigation.
- ***Confirmed MDR-TB.*** In this group there is a bacteriological confirmation, through a DST result, of resistance to Rifampicin (R) and Isoniazid (INH) with or without resistance to additional first and second line anti-TB drugs.
- Rifampicin Resistant TB: This group refers to Patient with a phenotypic drug susceptibility test or line probe assay or Gene Xpert showing resistance to Rifampicin, and where INH is susceptible, or INH resistance is unknown) (See Section 4.2.1).
- Poly-resistant TB. Patients in whom there is bacteriological evidence of resistance to more than one of the first line anti-TB drugs, but not including rifampicin and isoniazid together.
- Mono-resistant TB. Patients in whom there is bacteriological evidence of resistance to any of the first line anti-TB drugs.

### Registration based on site of drug-resistant TB disease (pulmonary and extra-pulmonary)

The recommended treatment regimens for drug-resistant forms of TB are similar, irrespective of site of disease. However, defining site remains important for recording and reporting purposes.

Pulmonary TB. Tuberculosis involving only the lung parenchyma.

Extrapulmonary TB. Tuberculosis of organs other than the lungs, e.g. pleura, lymph nodes, abdomen, genitourinary tract, skin, joints and bones, meninges. Tuberculous intrathoracic lymphadenopathy (mediastinal and/or hilar) or tuberculous pleural effusion, without radiographic abnormalities in the lungs.

The definition of an extrapulmonary case with several sites affected depends on the site representing the most severe form of disease.

Patients with both pulmonary and extrapulmonary TB should be classified as a case of pulmonary TB.

### Registration based on bacteriological examinations and Sputum conversion

The diagnostic strategy for MDR-TB is to perform DST in all patients before treatment using rapid molecular tests.

The Line Probe Assay test (LPA) detects Rifampicin and Isoniazid resistance, while the Xpert MTB/Rif only detects rifampicin resistance.

LPA can only be performed on DR-TB suspects with positive sputum microscopy result, while smear examination is not required for Xpert MTB/Rif test as it is an initial diagnostic test for TB in place of microscopy.

Results of such examinations should be properly recorded in the patient records and DR-TB register.

At least one sputum sample for smear and culture should always be taken at the time of DR-TB treatment start.

Smear microscopy **and** culture should be used to monitor patients throughout therapy on monthly basis.

### DR-TB patient registration group based on history of previous anti-tuberculosis treatment

DR-TB patients should be assigned a registration group based on their treatment history, which is useful in assessing the risk for MDR-TB.

The registration group will reflect the history of previous treatment and do not purport to explain the reason(s) for drug resistance. Registration groups are determined by treatment history at the time of collection of the sputum sample that was used to confirm MDR-TB. The groups are as follows:

Table 3.1: Definitions of DR-TB patient registration categories

**Classification according to history of previous drug use**, mainly to assign the appropriate treatment regimen.

| Type of Category | Definition |
| --- | --- |
| New Case | Patients who have never received anti-tuberculosis treatment, or who have received treatment for less than one month. This includes patients who had DST at the start of treatment for drug-susceptible tuberculosis, and then switch to a DR-TB regimen on account of resistance. |
| Previously treated with first-line drugs only: | Patients treated for one month or more for TB with first line anti-TB drugs only. |
| Previously treated with second-line drugs only | Patients treated for one month or more for TB with second line anti-TB drugs only with or without first line drugs. |

In addition to determining the treatment registration category of the DR-TB patients, all patients should have their HIV status recorded at the start of treatment.

Rapid HIV testing should be performed according to national protocol if there is any doubt about the patient's HIV status, or if the patient has not been tested recently.

## Treatment outcome definitions for DR-TB treatment

The treatment outcome definitions for MDR-TB patients are based on the use of laboratory smear and mycobacterial culture as monitoring tools. There are mutually exclusive DR-TB outcomes corresponding to the DOTS outcome categories for drug-susceptible TB. All patients should be assigned the first outcome they experience for the treatment being evaluated for recording and reporting purposes. The outcome definitions are as follows:

Table 3.2: Definitions of DR-TB treatment outcomes

| **TREATMENT OUTCOME** | **DEFINITION** |
| --- | --- |
| Cured | A DR-TB patients who completed the course of DR-TB treatment according to the national guideline without evidence of failure **AND** three or more consecutive cultures taken at least 30 days apart are negative after the intensive phase. |
| Treatment Completed | A DR-TB patients who completed the course of DR-TB treatment according to the national guideline without evidence of failure **BUT** no record that three or more consecutive cultures taken at least 30 days apart are negative after the intensive phase |
| Died | A DR-TB patient who dies for any reason during the course of MDR-TB treatment. |
| Treatment failure | A DR-TB patient whose treatment is terminated or needs a permanent regimen change of greater than or equal to 2 anti-TB drugs because of any of the following:   - lack of conversion in the continuation phase, *or* - bacteriological reversion* in the continuation phase after conversion** to negative, *or* - evidence of additional acquired resistance, *or* - adverse drug reactions |
| Lost to follow up | A DR-TB patient whose treatment was interrupted for two or more consecutive months for any reason. |
| Not evaluated | Patient with RR or MDR-TB for whom no treatment outcome is assigned. |

*reversion = two positive cultures taken at least 30 days apart

**conversion = two negative cultures taken at least 30 days apart

Patients who have **transferred in** should have their outcome reported back to the treatment centre from which they originally were registered. The responsibility of reporting their final outcomes belongs to the original treatment centre.

## Analysis of Interim results and final treatment outcomes

**Interim results:**

This provides an indication of how patients are faring well before final outcomes can be assessed, typically two to three years after the start of enrolment.

It includes assessment of culture conversion (for confirmed pulmonary cases) and death by six months as a proxy for final outcomes. The information should include loss to follow up, and the number of patients started on second-line drugs for MDR turned out not to be MDR.

**Final outcomes:**

The final outcome is the most important direct measurement of the effectiveness of the MDR-TB control programme in terms of patient care.

All confirmed MDR-TB patients entered on the treatment register should be assigned one of six mutually exclusive outcomes at the end of their therapy. These include Cured, completed, failed, lost to follow up, died and not evaluated (no outcomes assigned including transfer out).

1. DR-TB CASE FINDING STRATEGIES AND DIAGNOSIS

The fundamental principle underlying the case finding strategy in Swaziland is the need to ensure timely diagnosis of DR-TB in persons suspected for drug resistance with the view to significantly minimize diagnostic delays. This aims to increase the chances of cure, prevent the spread of the disease as well as development of further resistance and progression to permanent lung damage.

## DR-TB risk groups

The case finding is based on performing rapid DST in all persons suspected of DR-TB before treatment using rapid molecular tests that detects resistance to both Rifampicin and Isoniazid; or to rifampicin alone.

The priority risk categories will include the following:

1. **NEW PATIENT:**

Patients who have never received anti-tuberculosis treatment, or who have received treatment for less than one month. This includes patients who had DST at the start of treatment for drug-susceptible tuberculosis, and then switch to a DR-TB regimen on account of resistance.

1. **PREVIOUSLY TREATED WITH FIRST LINE DRUGS:**
   1. **New cases:**
      1. **Failure to convert at 2 months**: Patients receiving TB treatment as new case whose smear result is positive at the end of 2 months of treatment,
      2. **Treatment Failures:** Patients receiving treatment regimen for new drug-susceptible TB whose smear result remains positive at 5^th^ month of treatment, or has positive culture at any time after the intensive phase of treatment;
      3. **Return after loss to follow up:** Patients who return to treatment with confirmed TB after interruption of treatment for two (2) months or more;
   2. **Previously treated TB cases:**
      1. **Re-treatment Failures:** Patients receiving TB treatment regimen for previously treated cases, whose smear result remains positive at 5^th^ month of treatment;
      2. **Relapse cases:** Patients previously treated for drug-susceptible tuberculosis who have been declared cured or treatment completed, and again diagnosed with TB.
2. **PREVIOUSLY TREATED WITH SECOND LINE DRUGS:**

Patients who have received previous treatment with First Line and Second Line Drugs for more than one month;

1. **MDR/XDR-TB CONTACTS:**

Household or other close contacts of MDR/XDR-TB patients with symptoms suggestive of TB.

1. **HEALTH CARE WORKERS:**

Presenting with symptoms suggestive of TB.

1. **OTHER:**

All other persons suspected of having DR-TB who do not fit the above definition. This group includes TB patients who received treatment in facilities not accredited to the National TB Programme, and for whom the outcome of the latest treatment is unknown.

## Rapid DST methods

Rapid resistance testing is at the core of the DR-TB case-finding strategy to enhance early identification of cases for timely treatment initiation. Rifampicin being the most potent anti-tuberculosis drug of the first-line regimen is the prime target for rapid resistance testing.

Since rifampicin resistance most commonly occurs concomitantly with isoniazid (INH) resistance; a positive rapid test for rifampicin resistance is considered a strong indicator MDR-TB while a negative test makes a final diagnosis of MDR-TB highly unlikely.

Therefore a patient with positive result of rapid resistance testing for rifampicin should be considered an MDR-TB case and initiated on the standardized MDR-TB regimen. Xpert MTB/Rif and Line Probe Assay (LPA) described below are rapid rifampicin, and Rifampicin/INH rapid resistance testing techniques currently available in the country.

### Xpert MTB/Rif (Gene Xpert) test

This is an automated cartridge-based rapid molecular test for mycobacterium tuberculosis as well as detection of rifampicin resistance-conferring mutations directly from sputum providing both results within 2 hours.

The test which was endorsed by WHO in 2010 as an initial diagnostic test for pulmonary tuberculosis has about 40% sensitivity gain over light microscopy, and is useful in diagnosing TB in PLHIV who often have smear negative microscopy tests. A typical 4-module Gene Xpert can perform about 16 tests per a working day.

#### Use of Xpert MTB/Rif in new TB cases

Given the high HIV prevalence among incident TB cases in Swaziland, Gene Xpert should be performed as an initial diagnostic test for all persons suspected of having tuberculosis where the equipment is available or access to an off-site laboratory with the equipment is possible through efficient sample transportation.

- If the result is ***positive for MTB but negative for rifampicin resistance,*** the patient should be started on New TB case treatment regimen;
- If the result is ***positive for MTB and also positive for rifampicin resistance,*** the patient should registered as ***Rifampicin Resistant tuberculosis (RR-TB)*** in the DR-TB register.
- Full DST should be requested for all patients diagnosed and started on treated as RR-TB cases;
- Reference should be made to the algorithm on pages 32-33 for further management decisions.

#### Use of Xpert MTB/Rif in previously treated TB cases

Gene Xpert test should be performed for all previously treated TB cases who fail to convert at the end of three months intensive phase, or having positive smear at 5^th^ month of treatment where the equipment is available or access to an off-site laboratory with the equipment is possible through efficient sample transportation.

- If the result is ***positive for rifampicin resistance***, the case should be registered as ***Rifampicin Resistant tuberculosis (RR-TB)*** in the DR-TB register.
- If the result is positive for ***MTB but negative for rifampicin resistance***, the patient should be continued on the treatment for Previously treated cases;
- Full DST should be requested and treatment adjusted when the results are available.
- Reference should be made to the algorithm on page 32-33 for further management decisions.

The detection of rifampicin resistance using the Xpert MTB/Rif test is considered sufficient to start the patient on the standardized MDR-TB regimen.

Recognizing that detection of resistance to both rifampicin and Isoniazid would have better outcomes than rifampicin alone, full DST for first line drugs should be requested immediately following initiation of second line treatment on the basis of a positive Xpert MTB/Rif test.

#### Practical considerations for use of Xpert MTB/Rif

The following should be observed for any site for placement of an Xpert MTB/Rif equipment:

- Gene Xpert should be used at the peripheral level of the laboratory network as it has similar biosafety requirements to microscopy.
- Xpert should be used for testing of all persons suspected of having tuberculosis
- Ensure stable uninterrupted power supply, and use of UPS for each unit while in operation;
- Ensure adequate and secure storage space for Xpert Cartridges;
- There should be adedicated staff to perform the Xpert tests;
- Ensure calibration of the Xpert Module after every 2000 tests or one year, whichever comes first;
- The use of GeneXpert on other samples other than respiratory is not validated yet.

**Gene Xpert should be performed for all patients with symptoms suspected of PTB**

**Gene Xpert should not be used as follow-up tests for monitoring response to TB treatment**

#### Use of Xpert MTB/Rif results

On receiving the Xpert results, treatment decisions should be made as follows:

- All patients whose diagnosis of TB has been confirmed by Xpert MTB/Rif but negative for Rifampicin resistance (i.e MTB +ve and Rif –ve) should be registered as bacteriologically confirmed tuberculosis ***(MTB positive)***, and started on first line anti-TB treatment;
- No additional microscopy is required for establishing baseline smear result in persons diagnosed using the Xpert;
- Xpert MTB +ve patients should be monitored while on treatment using smear microscopy at the recommended intervals until completion of treatment;
- Patients with TB and rifampicin resistance confirmed by Xpert should be registered as ***Rifampicin-resistant tuberculosis (RR-TB).***
- RR-TB diagnosis is considered a proxy for MDR-TB; and should therefore be started on the standardized MDR-TB treatment regimen;
- Xpert diagnosed MDR-TB patients should be monitored by sputum microscopy and culture as per the National MDR-TB management guidelines.

***Note: registration of diagnosed TB cases using conventional TB diagnostics remains unchanged if the results of Xpert MTB/Rif is not available*.**

#### Repeat of Gene Xpert test

A repeat of the Gene Expert test should be requested only in the following circumstances:

- When the test is negative but patient’s symptoms highly suggestive of TB;
- Patient does not show improvement on broad spectrum antibiotics;
- If the repeat Xpert MTB/Rif test is negative, culture and DST should be requested, while patient is started on New case TB treatment regimen.

### Line Probe Assay (LPA)

The Line Probe Assay (LPA) is molecular method for rapid detection of resistance to both Rifampicin and Isoniazid (MDR-TB) in a sputum sample. LPA indirectly detects presence of *Mycobacterium tuberculosis* by amplifying DNA present in the sputum by polymerase chain reaction (PCR), which can be subsequently visualized on a strip by the presence or absence of bands. Results of LPA tests are obtainable within a 48 to 72 hours period, which will enable timely initiation of MDR-TB treatment.

The indications to send sputum samples for Line Probe Assay are:

- Smear positive diagnostic samples from facilities that do not have access to Gene Xpert yet.
- Sputum from patients with a Xpert MTB +ve and Rif –ve

## Conventional *M. TB* culture

Mycobacterial culture method is considered the gold standard. Culture significantly increase the number of TB cases found (often by 30–50%) and allow earlier detection of cases (often before they become infectious). Culture also provides the necessary isolates for conventional drug susceptibility testing (DST). The disadvantage of this method lies in the relatively high turn-around-time (TAT) for obtaining the result.

Two culture methods have been adopted in Swaziland namely:

- The conventional Solid Culture method (L-J techniques)
- Liquid culture using the Mycobactria Growth Indicator Tube (MGIT), an automated system, which has about 10% sensitivity gain over solid media culture.

The MGIT method therefore reduces the TAT for culture results from about 60 days in the case of L-J to about 15-30 days. The results can be reported as early as 10 days (if the culture is positive), or up to 42 days to report a final culture-negative result. However, the system is more prone to contamination, and the manipulation of large volumes of infectious material.

Good laboratory practice should be ensured to maintain the delicate balance between the yield of mycobacteria and contamination by other microorganisms.

Mycobacterial cultures should always be performed in containment laboratories with biosafety level BSL III.

Positive cultures must be speciated to differentiate M. tuberculosis from non-tuberculous mycobacteria (MOTT), which are more common in HIV-infected patients.

## DST for first line anti-TB drugs

The National TB Reference Laboratory (NRL) performs susceptibility testing for first line drugs using the MGIT system, which gives DST results to all the four major anti-TB drugs (Rifampicin, Isoniazid, Ethambutol and Streptomycin) within an estimated four weeks period.

DST for first line drugs should be requested for:

- Gene Xpert MTB+ve / Rif+ve patients
- Patients failing to convert or failing at any stage of treatment;
- Household or close contacts of confirmed MDR-TB patients.

## DST for Second line drugs and XDR-TB diagnosis

DST for Second Line anti-TB Drugs enables case-finding for XDR-TB and to assure proper treatment.

The two strongest risk factors for XDR-TB are:

- Failure of an anti-TB regimen that contains second-line drugs including an injectable agent and a fluoroquinolone;
- Close contact with an individual with documented XDR-TB or with an individual for whom treatment with a regimen including second-line drugs is failing or has failed.

Second line DST is recommended for the following categories of patients:

- All confirmed MDR-TB cases;
- Non-converting MDR-TB patients after 6-months of treatment;
- MDR-TB patients who previously converted and become culture positive on 2 consecutive samples collected at least 30 days apart;
- MDR-TB patients showing clinical deterioration.

The NRL should make SLDST results of all confirmed MDR-TB cases available to DR-TB treatment sites, and to the NTCP DR-TB Focal Point. However, if DST results are not routinely made available by the NRL, physicians should contact the laboratory to obtain such results to enable timely patient management decisions.

## DST specimen collection

- For diagnosis of suspected MDR-TB, one sputum specimen is required. DST does not routinely need to be carried out in duplicate;
- Collection of sputum sample should always follow the recommended NTCP standard procedure.

## The DR-TB diagnostic algorithm

The National programme has adopted the Xpert MTB/Rif as the initial diagnostic test for all persons suspected of having tuberculosis in view of the high HIV and DR rates among incident cases, while LED microscopy will be phased in as a replacement of ordinary light microscopy in the bacteriological monitoring of response to treatment.

The diagnostic pathway is based on whether or not the centre has access to at least rapid rifampicin resistance testing using the Xpert MTB/Rif.

Two algorithms are therefore currently recommended for use in Swaziland:

1. Algorithm 1: for health facilities without the GeneXpert
2. Algorithm 2: for health facilities with GeneXpert

The main provisions of the diagnostic algorithm are as follows:

- For all persons suspected of having tuberculosis regardless of HIV status before treatment initiation;
- Health care workers suspected of having tuberculosis.
- LPA should be requested for all persons diagnosed with smear positive tuberculosis, in case Xpert is not available.
- Conventional DST should be performed for all cases with any molecular method showing any resistance.

### Diagnostic Algorithm for settings without Xpert MTB/Rif

1. Includes MDR TB suspects

2. For discrepant results between genotypic and phenotypic tests refer to guidelines

Sputum microscopy / LED microscopy (2 smears)

Negative or smear not done

Positive

Further Clinical assessment

Course of antibiotics

Suggestive of TB

R+/H+

R+/H-

R-/H+

LPA

Treat with FLD.

R-/H-

DST for FLD & SLD^2^ and adjust SLD regimen.

Follow up 2, 5 months with Sputum microscopy

Positive

Negative

Continue treatment

Culture/DST for FLD

INH and RIF Resistance

Susceptible or other resistance

Treat with SLD

and send for SLD DST

Initiate appropriate treatment. Assess adherence

Follow up monthly with sputum microscopy and culture

Treat as Clinically diagnosed TB and treat with FLD.

Modify treatment.

Do FLD & SLD DST. Adjust treatment accordingly

Not suggestive of TB or improved on antibiotics

Consider other diagnosis

### Diagnostic Algorithm for settings with Xpert MTB/Rif

HIV test and Xpert MTB/Rif^2^

Xpert MTB+/Rif+

Xpert MTB+/Rif-

Xpert MTB-/Rif-

Treat with Standard MDR-TB regimen

Treat for TB with FLD

Send for LPA. IF INH Resistant modify R and send for FLD DST

Further clinical assessment including Chest X-Ray, Antibiotics

DST for FLD&SL and adjust SLD regimen according to results

lts

Suggestive of TB

Not suggestive longer considered TB suspect

Investigate for EPTB or other disease;

Initiate TB treatment on clinical grounds and

Send for MGIT Culture /DST for FLD

Exit algorithm.

Retest with Xpert

MTB+

MTB-

Follow up 2, 5 months with Sputum microscopy

Culture / DST for FLD^2^

Positive

Negative

Continue with FLD treatment

1. Includes MDR TB suspects

2. One sputum specimen

3. **Note**

- Infection control measures should commence immediately
- In the absence of Rapid resistance testing, the algorithm can be followed using liquid culture

Initiate appropriate treatment. Assess adherence

No Rifampicin and INH Resistance

Rifampicin and INH Resistance

Treat with SLD and send for SLD DST

Follow up monthly with sputum microscopy and culture

lts

1. TREATMENT OF DRUG RESISTANT TUBERCULOSIS

The current treatment strategy for DR-TB is also based on the recent WHO 2011 update on Programmatic Management of Drug-resistant tuberculosis. It takes into account the increased capacity of the laboratory network to perform rapid DST for rifampicin through the Xpert MTB/Rif); or for both rifampicin and isoniazid using the LPA method.

Decision for initiation of a patient on DR-TB treatment must therefore be informed by the patients DST results.

## Definitions of DR-TB treatment strategies

For the purpose of MDR-TB treatment in Swaziland, the following definitions apply:

1. **Standardized treatment:** The regimen that has been designed based on the country’s representative anti-TB drugs resistance survey (DRS 2009) data. This regimen is prescribed for all patients at diagnosis who do not yet have their full DST profile. For example, this is applicable in patients diagnosed as RR-TB cases using Gene Xpert, or MDR-TB cases diagnosed using LPA. In this case, all patients in this category receive the same standard combination of second line anti-TB medicines.
2. **Standardized treatment followed by individualized treatment:** This is the situation when patients are started on the standardized MDR-TB regimen; followed by adjustment of the treatment when the patient’s full DST results become available;
3. **Empirical treatment:** Where the MDR-TB regimen is specifically designed for the treatment of an individual on the basis of his/her history, and without any DST result showing the resistance pattern. For example, in instances where rapid diagnosis is not available but the patient has a good documented treatment record.
4. **Empirical treatment followed by individualized treatment:** This is the situation where a patient is started on an Empiric MDR-TB regimen, and later adjusted in the course of treatment when DST results become available (often the DST is done of both first- and second-line drugs).
5. **Individualized treatment:** This refers to an MDR-TB regimen designed based on the patient’s individual DST results and previous history of anti-tuberculosis treatment. For example, in patients that present to the clinic already having their result of a recent DST.

A combination of these strategies could be employed depending on the prevailing situation as follows:

## Classes of anti-tuberculosis drugs

The classes of anti-tuberculosis drugs have traditionally been classified into first- and second-line drugs, with isoniazid, rifampicin, pyrazinamide, ethambutol and streptomycin being the primary first-line drugs.

Second-line anti-TB drugs are also categorized according to the group system based on efficacy, experience of use and drug class.

These groups are referred to in the following sections and are very useful for the design of treatment regimens. The different groups are shown in **Table 5.1**. Not all drugs in the same group have the same efficacy or safety profile.

Table 5.1: Classification (groups) of second line anti-tuberculosis drugs

| **Grouping** | **Drugs** | **Remarks** |
| --- | --- | --- |
| **Group 1  First-line oral agents** | isoniazid (H); rifampicin (R); ethambutol (E); pyrazinamide (Z); rifabutin (Rfb)^a^ | - These are the most potent and best tolerated, and should be used if there is good laboratory evidence and clinical history to suggest that a drug from this group is effective. - Pyrazinamide should be part of the standardized MDR-TB regimen. - If strains resistant to low concentrations of INH but susceptible to higher concentrations, then use high-dose INH especially in the treatment of mono or poly-resistance. |
| **Group 2 Second-line parenteral agents**  **(injectable second- line anti-tuberculosis drugs)** | kanamycin (Km); amikacin (Am); capreomycin (Cm); streptomycin (S) | - All patients should receive at least one of these injectable agents so long as there is documented or suspected susceptibility. - Kanamycin or amikacin should be the first choice given the less otoxicity than streptomycin and high rates of streptomycin resistance. However, Amikacin and kanamycin are considered to be very similar and have a high frequency of cross-resistance. - If an isolate is resistant to both streptomycin and kanamycin, or if DRS data show high rates of resistance to amikacin and kanamycin, then capreomycin should be used. - For patients with renal insufficiency, hearing loss, or peripheral neuropathy, capreomycin should be considered. Although the side effect profile of capreomycin is similar to the aminoglycosides, adverse events are reported to be less frequent. - If creatinine clearance is significantly reduced, the dose of the injectable agent should be lowered. |
| **Group 3 Fluoroquinolones** | moxifloxacin (Mfx); levofloxacin (Lfx); ofloxacin (Ofx) | - All patients should receive one of the fluoroquinolones if the strain is susceptible or if the agent is thought to have efficacy. - Levofloxacin is the fluoroquinolone of choice. - However, Oflaxacin can be prescribed for mono or PDR, while moxifloxacin is recommended for management of XDR-TB. |
| **Group 4  Oral bacteriostatic second-line agents** | ethionamide (Eto); protionamide (Pto); cycloserine (Cs); terizidone (Trd); *p*-aminosalicylic acid (PAS) | - Two drugs from this group are added based on estimated susceptibility, drug history, efficacy, side-effect profile and cost. - Ethionamide or protiomamide should be chosen for inclusion in the MDR-TB regimen from this group. - Terizidone or Cycloserine should also be chosen from this group for inclusion in the regimen. If either Terizidone or Cycloserine cannot be used, PAS (preferably the PASER® granules, which are more tolerable) should be included instead. - The drugs in Group 4 may be started at a low dose and escalated over two weeks (Drug ramping). |
| **Group 5  Agents with unclear role in DR-TB treatment (not recommended by WHO for routine use in DR-TB patients)** | clofazimine (Cfz); linezolid (Lzd);  amoxicillin/clavulanate (Amx/Clv); thioacetazone (Thz); imipenem/cilastatin (Ipm/Cln); high-dose isoniazid (high-dose H);^b^ clarithromycin (Clr) | - These drugs are not recommended by WHO for routine use in DR-TB treatment because their contribution to the efficacy of multidrug regimens is unclear. However, they can be used in cases where adequate regimens are impossible to design with the medicines from Groups 1–4. They should be used in consultation with an expert in the treatment of DR-TB. - If a situation requires the use of Group 5 drugs, it is recommended to use at least two drugs from the group, given the limited knowledge of efficacy. |

*Source: Guidelines for Programmatic Management of Drug-resistant tuberculosis. Emergency update 2008. WHO/HTM/TB/200.402.*

^a^Rifabutin is not on the WHO List of Essential Medicines. It has been added here as it is used routinely in patients on protease inhibitors in many settings.

^b^ High-dose H is defined as 16–20 mg/kg/day.

## Swaziland Standardized DR-TB Regimen design

The design of the standardized MDR-TB treatment regimen in Swaziland takes into consideration the resistance pattern revealed by the national anti-TB drug resistance survey conducted in 2009-2010.

In addition, the following basic principles were also considered:

- The standardized regimens to be composed of at least five (5) drugs with either certain, or almost certain, effectiveness.
- The initial (intensive) phase to include an injectable agent (an aminoglycoside or capreomycin) included for a minimum of eight (8) months;
- The continuation phase should be for at least 12 months, thus giving a total duration of at least 20 months;
- A later-generation fluoroquinolone (Levofloxacin as drug of choice) to be included;
- Ethionamide or Prothionamide to form part of the regimen;
- Pyrazinamide to be included throughout the regimen;
- Cycloserine or PAS (where cycloserine cannot be used) should be included
- Drugs are administered seven days a week;
- Each dose is given as directly observed therapy (DOT) throughout the treatment. A treatment supporter card is marked for each observed dose.
- When possible, pyrazinamide, ethambutol and fluoroquinolones should be given once per day as the high peaks attained in once-a-day dosing may be more efficacious.
- Once-a-day dosing is permitted for other second-line drugs depending on patient tolerance; however ethionamide/prothionamide, Terizidone/cycloserine and PAS have traditionally been given in split doses during the day to reduce adverse effects.

The drug dosage should be determined by body weight. A suggested weight-based dosing scheme is shown in **Table *5.1*** and **Table 8.2** for adults and children respectively.

## Duration of second-line anti-TB regimens

MDR-TB treatment should be commenced timely following confirmation of diagnosis to increase the chances of treatment success.

The standard MDR-TB treatment regimen for patient without any previous MDR-TB treatment should be 20 months duration consisting of:

- At least 8 months intensive phase using 5 drugs, which includes an injectable agent;
- At least12 months continuation phase without the injectable agent.

## Role of drug susceptibility testing

Although the National TB Programme has adopted the rapid molecular DST methods, conventional DST (MGIT) test for a full resistance profile of the patient should be requested immediately. Depending on the results of the conventional DST, the DR-TB treatment regimen could be modified as appropriate.

DST for second line anti-TB drugs should be requested after commencement of MDR-TB treatment regimen. Based on the results of DST for second line drugs, diagnosis of XDR-TB can be made, and appropriate treatment should be initiated.

In the absence of XDR-TB, MDR-TB treatment regimen can also be adjusted if indicated by the results of DST for second line drugs.

***Note:*** *Awaiting results of DST should not delay treatment of DR-TB. And Second-line DST should only be performed on specimens after resistance to first-line drugs is confirmed or where special request is made by the clinician.*

This guideline does not recommend using DST for ethambutol, pyrazinamide and the drugs in Groups 4 and 5 to base individualized regimen design.

## Recommended Standardized treatment regimen

The recommended standardized regimen for MDR-TB inn Swaziland is:

**8 {Km (Am)-Lfx-Eto-Trd (Cs) /PAS-Z} / 12 {Lfx-Eto-Trd (Cs)/PAS-Z}**

*Kanamycin can be substituted with Amikacin, while Cycloserine can be used when Terizidone is not available.*

Patients who require second line treatment, but do not have DST results or a significant delay in obtaining the DST results is foreseen, should be started on this regimen until the results are available, after which treatment may be individualized depending on the DST pattern.

## Dosage of second line drugs

Dosing of anti-tuberculosis drugs is based on the weight of the patient. Monthly monitoring of body weight is therefore especially important in paediatric cases, with adjustment of doses as children gain weight.

### Adult and Adolescent dosages of second line drugs

The recommended adult dosage regimes for the second line anti-TB drugs is presented in the table 5.2 below.

Table 5.2: Adults and Adolescents dosage for second line anti-TB drugs.

| Drug | Average daily dosage | 33-50 KG | 51–70 KG | >70 KG (max dose) |
| --- | --- | --- | --- | --- |
| Pyrazinamide (Z) (500 mg) | 30–40 mg/kg daily | 1000-1750 mg | 1750 mg | 2000-2500 mg |
| Streptomycin (S) (1 g vial) | 15–20 mg/kg daily | 500-750 mg | 1000 mg | 1000 mg |
| Amikacin (Am) (1 g vial) | 15–20 mg/kg daily | 500-750 mg | 1000 mg | 1000 mg |
| Kanamycin (Km) (1 g vial) | 15–20 mg/kg daily | 500-750 mg | 1000 mg | 1000 mg |
| Capreomycin (Cm) (1 g vial) | 15–20 mg/kg daily | 500-750 mg | 1000 mg | 1000 mg |
| Ofloxacin (Ofx) (200 mg) | Usual adult dose 800 mg | 800 mg | 800 mg | 800-1000 mg |
| Levofloxacin (Lfx) (250 mg, 500 mg) | Usual adult dose 1000 mg | 750 mg | 750-1000 mg | 750-1000mg |
| Moxifloxacin (Mfx) (400 mg) | Usual adult dose 400 mg | 400 mg | 400 mg | 400 mg |
| Ethionamide (Eto) (250 mg) | 15–20 mg/kg daily | 500mg | 750 mg | 750–1000 mg |
| Protionamide (Pto) (250 mg) | 15–20 mg/kg daily | 500mg | 750 mg | 750–1000 mg |
| Cycloserine (Cs) (250 mg) | 15–20 mg/kg daily | 500mg | 750 mg | 750–1000 mg |
| Terizidone (Trd) (250 mg) | 15–20 mg/kg daily | 500mg | 750 mg | 750–1000 mg |
| PAS (4 g sachets) | 150 mg/kg daily | 8 g | 8 g | 8 -12 g |
| Group 5 drugs |  |  |  |  |
| Clofazinmine (Cfz) | Usual adult dose 300mg | 100mg | 300mg | 300mg |
| Amoxicillin/Clavulanate (Amx/Clv) | Normal dose 875/125mg x 2 or 500/125 x 3 | 875/125mg x 2 or 500/125 x 3 | 875/125mg x 2 or 500/125 x 3 | 875/125mg x 2 or 500/125 x 3 |
| Clarothromycin (Clr) | Normal dose 500 mg x 2 | Normal dose 500 mg x 2 | Normal dose 500 mg x 2 | Normal dose 500 mg x 2 |
| High-dose INH | 16-20mg/kg daily |  |  |  |

*Source: Guidelines for Programmatic Management of Drug-resistant tuberculosis. Emergency update 2008. WHO/HTM/TB/200.402.*

All patients receiving terizidone or cycoserine should receive pyridoxine. The recommended daily dose is 50 mg for every 250 mg of terizidone/cycoserine.

Once daily dosing for all drugs is preferred. However, most patients cannot tolerate once-daily dosing of ethionamide, protionamide, Terizidone (or cycloserine) and PAS, and these drugs may be given in divided doses twice-daily.

## Completion of the injectable agent (intensive phase)

The recommended duration of administration of the injectable agent, or the intensive phase, should be guided by culture conversion. The injectable agent should be continued for at least eight (8) months AND at least four (4) months after the patient first becomes and remains culture-negative.

In patients with extensive lung damage, unknown DST pattern, or those in whom the effectiveness of any of the agents is questionable, injectables should be continued for at least 6 months after culture conversion. The patient’s smear, culture, X-rays and other test results should be properly assessed to determine improvement or worsening of the clinical picture to help in deciding whether to continue an injectable agent longer than the above recommendation.

## Treatment phases

The treatment of DR-TB is administered in two phases namely the initial intensive phase and the continuation phase, with duration of at least 8 and at least 12 months respectively depending on culture conversion.

Table 5.3: Description of the phases of DR-TB treatment

|  | **Duration** | **Characteristics** |
| --- | --- | --- |
| Initial Phase | At least 8 months AND 4 months after 2 consecutive negative cultures (SEPARATE AT LEAST FOR 30 DAYS) | - Close monitoring for side effects - At least 5-6 drugs - Includes injectable drugs daily. - DOT for every dose taken |
| Continuation Phase | At least 12 months from time of culture conversion. | - Close monitoring for side effects - Usually all oral drugs preferably given once daily. - DOT for every dose taken |

## Treatment of XDR-TB

Swaziland reported its first XDR-TB cases in May 2008. XDR-TB has proven to be much more difficult to treat than MDR-TB and is extremely difficult to treat in HIV-positive patients. Patients whose second line DST results show resistance to both a fluoroquinolone (e.g Levofloxacin, Ofloxacin); and second line injectable agent (e.g Kanamycin, Amikacin or Cpareomycin) should be diagnosed as having XDR-TB.

The recommended approach in designing XDR-TB treatment is as follows:

- Use any Group 1 agents that may be effective.
- Use an injectable agent to which the strain is susceptible and consider an extended duration of use (12 months or possibly the whole treatment). If resistant to all injectable agents, it is recommended to use one the patient has never used before.
- Use a later-generation fluoroquinolone such as moxifloxacin.
- Use all Group 4 agents that have not been used extensively (more than 6 months) in a previous regimen or any that are likely to be effective.
- Use two or more agents from Group 5.
- Consider high-dose isoniazid treatment if low-level resistance is documented (used LPA reported mutations –KatG and InhA- for Isoniazid)
- Consider adjuvant surgery if there is localized disease.
- Ensure strong infection control measures.
- Treat HIV as recommended in this guideline
- Provide comprehensive monitoring and full adherence support.

The recommended regimen is as follows:

**12 {Cm/Mfx /Eto /Trd (Cs)/PAS/Z/Amx/Clv/Clf/INH_High dose_} - Intensive**

**24 {Mfx/Eto/Trd (Cs)/PAS/Z/Amx/Clv/Clf/INH_High dose_} - Continuation**

## Treatment of extra-pulmonary DR-TB

Extrapulmonary DR-TB is treated with the same strategy and duration as pulmonary DR-TB. If the patient has symptoms suggestive of central nervous system involvement and is infected with DR-TB, the regimen should use drugs that have adequate penetration into the central nervous system.

Note that: Pyrazinamide, protionamide/ethionamide and cycloserine have good penetration into the cerebrospinal fluid (CSF); kanamycin, amikacin and capreomycin do so only in the presence of meningeal inflammation; PAS and ethambutol have poor or no penetration. The fluoroquinolones have variable CSF penetration, with better penetration seen in the later generations.

## Treatment of mono- and poly resistant TB

Patients with mono or poly resistance will be identified either through rapid resistance testing or routine DST testing. These patients should be managed based on the pattern of resistance reflected in the DST results. Patients with mono and poly resistant TB should be managed at the isolation units of TB diagnostic units or facility in collaboration with the National clinical team.

### Consequences for reporting

The regimens for treating mono or poly resistant cases are essentially “modifications” or rather strengthening of the treatment regimens for new or previously treated cases. Treatment records of mono or poly resistant cases should be recorded in the DR-TB Register.

### Initiation of treatment for mono or poly resistant cases:

Treatment of mono and poly resistant cases should be initiated by a medical officer either at the general hospital or the national TB Hospital in collaboration with the Central clinical team. Prior to initiation of treatment, all baseline investigations should be carried as outlined in chapter 7 of this guideline.

Treatment follow-up and monitoring for side effects and adverse reactions should follow the same protocol as for MDR-TB.

#### Treatment options for mono or poly resistant cases:

The following table provides a guide to suggested regimens depending on the resistance pattern.

Table 5.4: Recommended treatment regimens for use in mono- and poly resistant cases

| **Patter of resistance** | **Suggested regimen** | **Minimum duration of treatment (Months)** | **Comments** |
| --- | --- | --- | --- |
| **H (± S)** | R, Z, E + Lfx  (RHZE)+ Lfx | 6-9 | A fluoroquinolone may strengthen the regimen for patients with extensive disease. |
|  |  |  |  |
|  |  |  |  |
| **H, E, Z ± (S)** | 18 [R, Lfx, Eto] + Am (Km) for 3 months | 18 | A longer course (6 months) may strengthen the regimen for patients with extensive disease. Ensure clinical monitoring and Culture. |

H = isoniazid; R = rifampicin; E = ethambutol; Z = pyrazinamide; S = streptomycin

aAdapted from *Drug-resistant tuberculosis: a survival guide for clinicians* (*3*)

In view of the use of Gene Xpert and LPA as first strategy, the complete pattern of resistance (S and E) will take 1-2 months, and with result for Z currently unreliable, it is important to prescribe 4 strong drugs to which the patient is sensitive.

## Role of Surgery in DR-TB treatment

Some DR-TB patients may benefit from surgical interventions if there is significant lung damage. The most common operative procedure in patients with pulmonary DR-TB is resection surgery (taking out part or all of a lung). It is therefore considered an adjunct to chemotherapy and appears to be beneficial for patients when skilled thoracic surgeons and excellent postoperative care are available. It is not indicated in patients with extensive bilateral disease.

Surgery as an adjunct to chemotherapy for patients with localized disease can significantly improve outcomes where skilled thoracic surgeons and excellent postoperative care are available.

When resectable disease is present, surgery should be considered for the following cases:

- Failure to demonstrate clinical or bacteriologic response to chemotherapy after three to six months of treatment.
- High likelihood of failure or relapse, due to high degree of resistance or extensive parenchymal involvement, regardless of smear and culture status.
- Morbid complications of parenchymal disease, e.g., hemoptysis, bronchiectasis, bronchopleural fistula, or empyema.
- Recurrence of positive culture status during MDR-TB treatment.
- Relapse after completion of DOTS-Plus therapy and under consideration for further individualized chemotherapy.

The following lists the priority of cases that should be considered:

1. Persistent or severe haemoptysis.

2. Patients who have high levels of resistance.

- Patients with strains of bacteria demonstrating high levels of resistance should undergo an evaluation to determine the possibility of resection of their diseased lung.
- Resection should occur as quickly as possible in these patients.
- They should be continued on their regimens for their full course of treatment.
- Even if the patient converts and becomes bacteriologically negative, given the high resistance they are at high risk for eventual failure and therefore should be considered for surgery.

3. Localized surgical disease (i.e., cavities or destroyed lung tissue) in patients who remain culture-positive.

- All patients with what appears to be localized cavities or damaged lung on chest X-ray should undergo an evaluation for surgical resection of the damaged lung or cavity.

4. Localized surgical disease (i.e., cavities or destroyed lung tissue) in culture-negative patients.

### Timing of surgery

Although smear conversion prior to intervention is ideal, the timing of surgery should occur early in therapy and is normally undertaken in the first two to six months of therapy. If it is not possible to achieve conversion prior to surgery, then at least three months of chemotherapy is recommended.

Evaluating patients for surgery

Patients should receive anti-tuberculosis drugs based on documented or presumptive drug sensitivities, both prior to and after surgery. The work-up of the surgical candidate is described below.

- Evaluation should begin with a computed tomography scan of the chest to evaluate the extent of disease. If there is localized disease or a question as to whether the disease is sufficiently localized to be resected, the patient’s history and tomography should be evaluated by a thoracic surgeon experienced in operating on patients with tuberculosis.
- When a patient is thought to be an acceptable surgical candidate, the patient should have pulmonary function tests and, in some cases, ventilation perfusion scans to evaluate his predicted postoperative forced expiratory volume in one second (FEV1). Patients should have a predicted postoperative FEV1> 0.8 to be considered candidates for surgery.
- If a patient’s predicted postoperative FEV1 is acceptable, analysis of blood for HCT, ABG, performed preoperatively. A preoperative EKG should be performed on patients older than 50 and on patients with diabetes.

### Length of treatment after surgery

In all patients, therapy should continue for 18 to 24 months of consecutive negative cultures. In patients receiving adjunctive surgery, therapy may be prolonged in the postoperative period.

- In patients who are smear- or culture-positive at the time of surgery, treatment is continued for a minimum of 18 months of documented culture negativity.
- In patients who are smear- and culture-negative at the time of surgery, treatment should be continued for a minimum of 18 months after culture conversion and no less than 6 months after surgery.
- If pathology reveals viable bacilli on culture, it may be reasonable to continue therapy for 18 to 24 months after the surgery rather than 18 months after the culture conversion of sputum.

## Adjuvant therapy

### Nutritional support

In addition to causing malnutrition, DR-TB can be exacerbated by poor nutritional status. Without nutritional support, patients, especially those already suffering from baseline hunger, can become enmeshed in a vicious cycle of malnutrition and disease.

The second-line anti-tuberculosis medications can further decrease appetite, making adequate nutrition a greater challenge, and therefore nutritional support to be provided. Provision of free staple foods, and whenever possible should include a source of protein.

### Corticosteroids

The adjuvant use of corticosteroids in DR-TB patients has been shown to reduce mortality and can be beneficial in conditions such as severe respiratory insufficiency, and central nervous system, pericardial involvement and laryngeal TB.

Prednisone is commonly used, starting at approximately 1 mg/kg and gradually decreasing the dose to a total week dose 10 mg when a long course is indicated. Corticosteroids may also alleviate symptoms in patients with an exacerbation of obstructive pulmonary disease. In these cases, prednisone may be given in a short taper over 1–2 weeks, starting at approximately 1 mg/kg and decreasing the dose by 5–10 mg per day. Injectable corticosteroids are often used initially when a more immediate response is needed.

## Compassionate use of drugs in DR-TB Management

Compassionate use refers to the use of potentially life-saving experimental treatments to patients suffering from a disease for which no satisfactory authorized or effective therapy exists.

The addition of drugs that are still under clinical trials, not officially registered for use in the treatment regimen, but could potentially provide the last resort and a life-saving opportunity for some patients with complicated MDR-TB or XDR TB cases facing life-threatening situations.

Compassionate use of “Investigational New Drugs” relates to patients’ treatment, and is not medical research. For many patients, these treatments represent their last hope.

Both MDR-TB and XDR-TB can be life-threatening diseases for which approved drugs alone may be ineffective. In some cases, experimental TB drugs, used in combination with approved drugs, could potentially be effective or life-saving.

Compassionate Use of IND may be considered for patients presenting with a life-threatening condition (e.g. deteriorating clinical condition due to TB and/or severe immune depression) when available treatments have failed or are very likely to be ineffective (clinical or bacteriological failure), or no medical or surgical options are appropriate. However, compassionate use should only be considered if conditions for an adequate management of DRTB patients are in place, which should include:

- optimal treatment regimen;
- clinical, biological and bacteriological monitoring;
- adherence support and follow-up,
- drug susceptibility testing (DST) by a validated laboratory.

The National Tuberculosis Control Programme, upon advise of the National TB Technical working Group (TWG), the National DR-TB Clinical Expert Committee; and ethical clearance from the Swaziland National Ethics Committee, shall consider initiatives for compassionate use to be implemented in the country on any candidate substance that is perceived to be potentially beneficial.

### General guide for compassionate use

The following criteria will serve as a general guide for compassionate use in the country:

- Informed consent of the patient or relations is obtained;
- Confirmed case of MDR or XDR TB;
- Available treatments with second line anti-TB drugs have failed or are very likely to be ineffective (e.g. regimen comprises less than 3 highly likely effective drugs and/or clinical evolution shows that the treatment is not effective)
- No medical or surgical options are appropriate and/or enough
- Drug susceptibility to at least one effective drug is confirmed.
- The experimental drug should never be used as a mono-therapy. It should always be used in conjunction with other drug(s) with proven or probable efficacy in order to prevent emergence of resistance to the experimental drug.
- The patient is not pregnant and not breastfeeding.
- The use of the experimental drug does not result in the discontinuation of an essential effective drug. Special attention will be paid if the use of the drug imposes the replacement of a TB drug by a less effective one.

1. TREATMENT OF DR-TB IN SPECIAL SITUATIONS

In the treatment of DR-TB consideration should be made for certain physiological or medical conditions that may either be affected or may potentially interfere with the treatment regimen. Special precautions are required in these conditions, which include pregnancy, breast feeding, use of contraception, diabetes mellitus, renal insufficiency, liver disorders, seizure disorders, psychiatric disorders, and substance dependence. The recommended management approaches are elaborated below.

## Pregnancy

Pregnancy is not a contraindication for treatment of active DR-TB, given the greater risks posed by the disease to the lives of both mother and fetus.

However, pregnancy test should be performed on all female patients of child bearing age as part of initial assessment before starting second line treatment, and non-pregnant ones should be offered advice on contraception.

Pregnant patients should be carefully evaluated, taking into consideration gestational age and severity of the DR-TB. The risks and benefits of treatment should be carefully considered, with the primary goal of smear conversion to protect the health of the mother and child, both before and after birth.

For a decision regarding starting a pregnant female on treatment, this guideline recommends that ***if the condition of patient is not life-threatening, DR-TB treatment should delayed until second trimester****.* This is to avoid the teratogenic effects which are more likely to occur in the first trimester.

The decision to postpone the start of treatment should be agreed by both patient and doctor after analysis of the risks and benefits. It should be based on a thorough clinical judgment resulting from the analysis of life-threatening signs/symptoms and severity/aggressiveness of the disease (usually reflected in extent of weight loss and lung affection during the previous weeks). When therapy is started, three or four oral drugs with demonstrated efficacy against the infecting strain should be used and then reinforced with an injectable agent and possibly other drugs immediately postpartum.

**Avoid injectable agents:** For the most part, aminoglycosides should not be used in the regimens of pregnant patients due to the risk of toxicity to the developing fetal ear. Capreomycin may also carry a risk of ototoxicity but is the injectable drug of choice if an injectable agent cannot be avoided.

**Avoid ethionamide**: Ethionamide can increase the risk of nausea and vomiting associated with pregnancy, and the possibility of teratogenic effects. If possible, ethionamide should be avoided in pregnant patients.

## Breastfeeding

A breastfeeding mother with active DR-TB should receive a full course of anti-tuberculosis treatment, as timely and effective chemotherapy is the best way to prevent transmission of tubercle bacilli to her baby. It is recommended to give Pyridoxine 1-2mg/kg body weight to babies of breastfeeding mothers.

Although most anti-tuberculosis drugs will be found in the breast milk in concentrations that would equal only a small fraction of the therapeutic dose used in an infant, any effects on infants of such exposure during the full course of DR-TB treatment have not been established. The mother and her baby should not be completely separated.

However, if the mother is sputum smear-positive, the cooperation of a family member should be sought to care of the infant until the mother becomes sputum smear-negative. As whenever the mother and infant spend time together, they should do so in a well-ventilated area or outdoor.

If considered necessary and depending on the setting, the mother could be offered the option of using a surgical mask or an N-95 respirator until she becomes sputum smear-negative.

In the event of a perceived effect of second line drugs in the infant, infant formula options should be considered as an alternative to breastfeeding.

## Contraception

For patients on oral contraceptives, there is no contraindication to its use with the non-rifamycin containing regimens. However, patients who experience severe nausea or vomit directly after taking an oral contraceptive can be at risk of decreased absorption of the drug and therefore of decreased efficacy.

The patients should be advised either to change the timing of taking their contraceptives or consider other non-drug contraceptive methods e.g. barrier methods or injectables and preferably both depending on patient preferences and suitability.

For patients not using any contraceptives, the dual contraceptive method is highly recommended.

## Diabetes mellitus

Diabetic patients with MDR-TB are at risk for poor outcomes. In addition, the presence of diabetes mellitus may potentiate the adverse effects of anti-tuberculosis drugs, especially renal dysfunction and peripheral neuropathy. Diabetes must be managed closely throughout the treatment of DR-TB. The health-care provider should be in close communication with the physician who manages the patient’s diabetes.

Oral hypoglycaemic agents are not contraindicated during the treatment of DR-TB but may require the patient to increase the dosage. Use of ethionamide or protionamide may make it more difficult to control insulin levels. Creatinine and potassium levels should be monitored more frequently, often weekly for the first month and then at least monthly thereafter.

**1. Medical follow-up**

- Diabetes must be managed closely throughout DOTS-Plus treatment. The TB physician should be in close communication with a physician who manages the patient’s diabetes.

**2. Education for the patients**

- Diabetic diet—all nurses and promoters with diabetic patients should be familiar with the basics of the diabetic diet.
- Weight control
- Exercise
- Foot care
- Symptoms of hypo- and hyperglycemia

**3. Glucose monitoring**

- Goals for capillary blood testing: 80–120 mg/ dl before meals; 100–140 mg/dl before bedtime; the range should be higher if patient has a history of hypoglycemia.
- Patients may need a period of intensive glucose monitoring until these goals are met.
- Once a patient is on a stable dose of insulin, his or her blood sugar may be monitored four times weekly to ensure that targets are being maintained.
- If a patient is on oral anti-diabetic agents, his or her blood sugars may be monitored twice weekly.

**4. Regular monitoring**

- Creatinine and potassium should be monitored weekly for the first month and then at least monthly thereafter.
- If the creatinine rises, a creatinine clearance should be checked and antituberculosis medications should be adjusted accordingly. Once the dose is adjusted, the creatinine should be checked weekly until it has stabilized.
- HbA1C every three months if treatment changes or patient is not meeting goals; every 6 months if stable.
- Goal for HbA1C < 7.
- Retinal examination annually.

**5. Screening and treatment for hypertension**

- Blood pressure checks every month.
- Hypertensive patients with diabetes should be started on an ACE-inhibitor.

**6. Prevention of diabetic nephropathy**

- Injectable dosing according to the renal protocol.
- Consider using an ACE-inhibitor for patients with albuminuria (>300 mg/24 h).

## Renal insufficiency

Renal insufficiency caused by longstanding TB infection itself or previous use of aminoglycosides is not uncommon. Great care should be taken in the administration of second-line drugs in patients with renal insufficiency, and the dose and/or the interval between dosing should be adjusted according to Table 6.1 below.

The formula to calculate the creatinine clearance (CrCl) or the glomerular filtration rate (GFR) is:

GFR=(140 -age)(ideal body weight in kg) for men (x 0.85 for women)

(72)(serumcreatinine, mg/dl)

Normal values for the creatinine clearance are:

Men: 97 to 137 ml/min

Women: 88 to 128 ml/min

**Practical Example:** On adjusting the dose of a medication in renal insufficiency:

Calculate the dose of capreomycin (CM) to be prescribed for a 59 year old male patient having a serum creatinine of 2.4 mg/dl, and an ideal bodyweight of 53 kg.

**Step 1:** Calculate the Glomerular Filtration Rate (GFR)

GFR =(140 - Age) (ideal body weight in kg)

(72)(serumcreatinine, mg/dl)

= (140 -59)(53)= 24.8 ml/min

(72)(2.4)

**Step 2:** Refer to table 6.1 and make the appropriate dosage adjustment.

In this example, the 24.8 ml/min falls below 30 ml/min. the dose of CM given in the table is 12-15 mg/kg.

Therefore the dose to be prescribed should be between 12 x 53 = 636 mg and 15 x 53 = 795 mg. it is reasonable to choose a dose between these two values that can be easily drawn from the injection vial. In this case 750 mg would be the logical choice.

**Step 3:** Check Creatinine periodically (weekly or more frequently in patients with severe renal insufficiency and adjust medications for any change.

Table 6.1: Adjustment of anti-tuberculosis medication in renal insufficiency.

| **Drug** | **Change in frequency?** | **Recommended dose^c^ and frequency for patients with creatinine clearance <30 ml/min or for patients receiving haemodialysis** |
| --- | --- | --- |
| isoniazid | No change | 300 mg once daily, or 900 mg three times per week |
| rifampicin | No change | 600 mg once daily, or 600 mg three times per week |
| pyrazinamide | Yes | 25–35 mg/kg per dose three times per week (not daily) |
| ethambutol | Yes | 15–25 mg/kg per dose three times per week (not daily) |
| ofloxacin | Yes | 600–800 mg per dose three times per week (not daily) |
| levofloxacin | Yes | 750–1000 mg per dose three times per week (not daily) |
| moxifloxacin | No change | 400 mg once daily |
| cycloserine | Yes | 250 mg once daily, or 500 mg/dose three times per week^d^ |
| terizidone | – | Recommendations not available |
| protionamide | No change | 250–500 mg per dose daily |
| ethionamide | No change | 250–500 mg per dose daily |
| *p*-aminosalicylicacid^e^ | No change | 4 g/dose, twice daily |
| streptomycin | Yes | 12–15 mg/kg per dose two or three times per week (not daily)^f^ |
| capreomycin | Yes | 12–15 mg/kg per dose two or three times per week (not daily)^f^ |
| kanamycin | Yes | 12–15 mg/kg per dose two or three times per week (not daily)^f^ |
| amikacin | Yes | 12–15 mg/kg per dose two or three times per week (not daily)^f^ |

^a^ Source: *Adapted from WHO guidelines for programmatic management of drug resistant tuberculosis. Emergency update 2008. WHO/HTM/TB/2008.402*

^b^ For Group 5 drugs see manufacturers’ recommendations on adjustment in renal insufficiency.

^c^ To take advantage of the concentration-dependent bactericidal effect of many anti-tuberculosis drugs, standard doses are given unless there is intolerance.

^d^ The appropriateness of 250 mg daily doses has not been established. There should be careful monitoring for evidence of neurotoxicity (if possible measure serum concentrations and adjust accordingly).

e Sodium salt formulations of PAS may result in an excessive sodium load and should be avoided in patients with renal insufficiency. Formulations of PAS that do not use the sodium salt can be used without the hazard of sodium retention.

^f^ Caution should be used with the injectable agents in patients with renal function impairment because of the increased risk of both ototoxicity and nephrotoxicity.

### Indications for Renal dialysis and how to access

Renal dialysis may be required to provide an artificial replacement for lost kidney function (renal replacement therapy) due to renal failure. Dialysis may be used for very sick patients who have suddenly but temporarily, lost their kidney function (acute renal failure); or for quite stable patients who have permanently lost their kidney function (chronic kidney disease).

The decision to initiate dialysis or hemofiltration in patients with renal failure depends on several factors. These can be divided into acute or chronic indications:

The indications for dialysis in the patient with acute kidney insufficiency are:

- Metabolic acidosis where correction with sodium bicarbonate is impractical or may result in fluid overload;
- Electrolyte abnormality, such as severe hyperkalemia;
- Intoxication, that is, acute poisoning with a dialysable drug, such as lithium, or aspirin;
- Fluid overload not expected to respond to treatment with diuretics;
- Complications of uremia, such as pericarditis, encephalopathy, or gastrointestinal bleeding;

The indications for a patient with chronic renal failure are:

- Symptomatic renal failure
- Low glomerular filtration rate (GFR) of less than 10-15 mls/min/1.73m^2^). In diabetics dialysis should be started earlier.
- Difficulty in medically controlling fluid overload, serum potassium, and/or serum phosphorus when the GFR is very low.

## Liver disorders

The first-line drugs isoniazid, rifampicin and pyrazinamide are all associated with hepatotoxicity. Of the three, rifampicin is least likely to cause hepatocellular damage, although it is associated with cholestatic jaundice. Pyrazinamide is the most hepatotoxic of the three first-line drugs. Among the second-line drugs, ethionamide, protionamide and PAS can also be hepatotoxic, although less so than any of the first-line drugs. Hepatitis occurs rarely with the fluoroquinolones.

Patients with a history of liver disease can receive the usual DR-TB chemotherapy regimens provided there is no clinical evidence of severe chronic liver disease, hepatitis virus carriage, and recent history of acute hepatitis or excessive alcohol consumption. However, hepatotoxic reactions to anti-tuberculosis drugs may be more common in these patients and should be anticipated.

In general, patients with chronic liver disease should not receive pyrazinamide. All other drugs can be used, but close monitoring of liver enzymes is advised. If significant aggravation of liver inflammation occurs, the drugs responsible may have to be stopped.

Uncommonly, a patient with TB may have concurrent acute hepatitis that is unrelated to TB or anti-tuberculosis treatment. In this case, clinical judgment is necessary. In some cases, it is possible to defer anti-tuberculosis treatment until the acute hepatitis has been resolved. In other cases when it is necessary to treat DR-TB during acute hepatitis, the combination of four non-hepatotoxic drugs is the safest option.

## Seizure disorders

Some patients requiring treatment for DR-TB will have a previous or current medical history of a seizure disorder. The first step in evaluating such patients is to determine whether the seizure disorder is under control and whether the patient is taking anti-seizure medication.

If the seizures are not under control, initiation or adjustment of anti-seizure medication will be needed before the start of DR-TB therapy. In addition, any other underlying conditions or causes of seizures should be corrected.

Cycloserine should be avoided in patients with active seizure disorders that are not well controlled with medication. However, in cases where cycloserine is a crucial component of the treatment regimen, it can be given and the anti-seizure medication adjusted as needed to control the seizure disorder. The risks and benefits of using cycloserine should be discussed with the patient and the decision on whether to use cycloserine made together with the patient.

In mono- and poly-resistant cases, the use of isoniazid and rifampicin may interfere with many of the anti-seizure medications. Drug interactions should be checked before their use **(see Table 10.1** *for drug interactions).*

Seizures that present for the first time during anti-tuberculosis therapy are likely to be the result of an adverse effect of one of the anti-tuberculosis drugs. *More information on the specific strategies and protocols to address adverse effects is provided in* **Table 6.2.**

## Psychiatric disorders

Patients with psychiatric disorders should be managed as much as possible at the DR-TB Clinical Management Centres in close collaboration with the National Psychiatric Hospital.

It is advisable for psychiatric patients to be evaluated by a health-care worker with psychiatric training before the start of treatment for DR-TB. The initial evaluation documents any existing psychiatric condition and establishes a baseline for comparison if new psychiatric symptoms develop while the patient is on treatment.

Any psychiatric illness identified at the start of or during treatment should be fully addressed. There is a high baseline incidence of depression and anxiety in patients with MDR-TB, often connected with the chronicity and socioeconomic stress factors related to the disease.

Treatment with psychiatric medication, individual counseling and/or group therapy may be necessary to manage the patient suffering from a psychiatric condition or an adverse psychiatric effect caused by medication.

Group therapy has been very successful in providing a supportive environment for MDR-TB patients and may be helpful for patients with or without psychiatric conditions. (Adequate measures to prevent infection risk should be in place for the group therapy.)

The use of cycloserine is not absolutely contraindicated for the psychiatric patient. Adverse effects from cycloserine may be more prevalent in the psychiatric patient, but the benefits of using this drug may outweigh the potentially higher risk of adverse effects. Close monitoring is recommended if cycloserine is used in patients with psychiatric disorders.

All health-care workers treating DR-TB should work closely with a mental health specialist and have an organized system for psychiatric emergencies. Psychiatric emergencies include psychosis, suicidal tendencies and any situation involving the patient’s being a danger to him or herself or others. Additional information on psychiatric adverse effects is provided in ***Table 7.3****.*

## Substance dependence

Patients with substance dependence disorders should be offered treatment for their addiction. Complete abstinence from alcohol or other substances should be strongly encouraged, although active consumption is not a contraindication for anti-tuberculosis treatment. If the treatment is repeatedly interrupted because of the patient’s dependence, therapy should be suspended until successful treatment or measures to ensure adherence have been established. Good DOT gives the patient contact with and support from health-care providers, which often allows complete treatment even in patients with substance dependence.

Cycloserine will have a higher incidence of adverse effects (as in the psychiatric patient) in patients dependent on alcohol or other substances, including a higher incidence of seizures. However, if cycloserine is considered important to the regimen, it should be used and the patient closely observed for adverse effects, which are then adequately treated.

It is prudent to have a psychiatric evaluation before the start of treatment for all patients with MDR TB. The initial evaluation serves to document any preexisting psychiatric condition and establishes a baseline for comparison if new psychiatric symptoms develop while on treatment. Any psychiatric illness identified at the start of or during treatment should be addressed fully. There is a high baseline incidence of depression and anxiety in patients with MDR TB, often connected to the chronicity and socioeconomic stressors related to the disease.

Treatment should not be delayed while waiting for a psychiatric evaluation. If a psychiatrist is not available, the treating physician should do an initial psychiatric evaluation. It is also acceptable to wait until the patient is smear-negative prior to a full psychiatric evaluation, in order to decrease MDR TB exposure to the psychiatrist, other health care workers, and patients at the psychiatric clinic.

Treatment with psychiatric medications, individual counseling, and/or group therapy may be needed to manage the patient suffering from a psychiatric condition or adverse reaction.

The physician treating tuberculosis should be involved in all management modalities. Group therapy has been very successful in providing a supportive environment for the MDR TB patient and may be helpful for patients with or without psychiatric conditions.

The patient with a substance dependency poses a difficult challenge. Treatment for addiction should be offered in such cases. Complete abstinence from alcohol or drugs should be strongly encouraged. However, active alcohol or drug use is not an absolute contraindication to treatment. If treatment is repeatedly interrupted due to the patient’s addiction, MDR TB therapy should be suspended until treatment for the addiction is successful.

## HIV-infected patients

Given the important interaction between HIV infection and drug-susceptible and DR-TB, a full chapter ***(Chapter 10)*** is devoted to this subject.

1. MONITORING DR-TB TREATMENT AND MANAGEMENT OF ADVERSE EFFECTS

## Pre-treatment screening and evaluation

Pre-treatment assessment should be systematically conducted on all patients in order to identify those patients at greater risk of adverse effects, poor outcomes and also to establish a baseline.

The evaluation should include a thorough medical history, physical examination and baseline laboratory evaluations. Certain pre-existing conditions, which may affect the progress should be diagnosed early through more intensive baseline investigations and follow up. These should include:

**History including past medical history:**

- HIV infection
- diabetes mellitus
- Hypertension
- History of Chronic liver disease ( or jaundice)
- renal insufficiency
- acute or chronic liver disease
- thyroid disease
- Allergies
- Prior history of psychiatric illness
- Drug, alcohol or tobacco use
- Last menstrual period, contraception, pregnancy or breast feeding

Note: Contraception during treatment for women of childbearing age should be discussed, and strongly recommended.

**Physical Examination**

- Vital signs
- Heart rate
- Blood pressure
- Respiratory rate
- Height and weight

Review of systems:

This should include assessment of cough, sputum production, fever, night sweats, weight loss (include previous weight when healthy, with date), dyspnea, appetite, abdominal pain, nausea, vomiting, diarrhea, constipation, headache, peripheral leg pain, hearing loss, depression and anxiety.

**Laboratory tests:**

All patients starting MDR-TB treatment should have the following tests:

- TB cultures and DST
- Hematology: FBC, HB and blood film for malaria parasites;
- Chemistry: such as Glucose, K, Urea, Creatinine), LFT
- Serology: HIV rapid testing
- Flow-cytometry: CD4 cell count and CD4% in children
- ZN or Fluorescent microscopy of sputum smears
- Syphilis serology
- Pregnancy test
- Baseline Psychiatric evaluation
- Baseline Audiometry

## Monitoring progress of treatment

Patients should be monitored closely for signs of both treatment failure and side-effects. As laboratory tests evidence of improvement often lag behind clinical response, the most important way to monitor response to treatment is through *regular history-taking and physical examination.*

The classic symptoms of TB, including cough, sputum production, fever and weight loss, generally improve within the first few months of treatment and should be monitored frequently by health-care providers.

***The recurrence of TB symptoms after sputum conversion, for example, may be the first sign of treatment failure****.*

For children, height and weight should be measured regularly to ensure that they are growing normally. A normal growth rate should resume after a few months of successful treatment.

The chest radiograph may be unchanged or show only slight improvement, especially in re-treatment patients with chronic pulmonary lesions.

Chest radiographs/x-ray (CXR) should be taken:

- on initiation of treatment
- at least every six months
- when a surgical intervention is being considered,
- or whenever the patient’s clinical situation has worsened.

***NB: A central Clinical team from the TB hospital will visit diagnostic centres on a monthly basis to provide support and review patients.***

## Follow-up bacteriological examinations (Microscopy and Culture)

The most objective evidence of response to therapy is conversion of the sputum smear and culture from positive to negative.

All patients receiving MDR-TB treatment should be monitored monthly using both sputum smear and mycobacterial culture.

Persistently positive sputum smears and cultures for AFB should be assessed for mycobacteria other than tuberculosis (MOTT), as overgrowth with MOTT in lung damage secondary to TB is not uncommon. In such cases, although DR-TB may be adequately treated, treatment may need to be directed towards the NTM as well.

Sputum conversion is slower in DR-TB than in drug-susceptible TB. Paucibacillary culture results should not be automatically regarded as negative when treating DR-TB. Acquired drug resistance and treatment failure often begin with the growth of one or two colonies on a sputum culture.

**Culture conversion** should **not** be considered to be **equivalent to cure**. *A certain proportion of patients may initially convert and later revert to positive sputum culture.*

### Culture conversion

Culture conversion is defined as two consecutive negative smears and cultures taken 30 days apart. After the end of the intensive phase, the minimum period recommended for bacteriological monitoring is monthly smears and cultures **(*See Table 7.1*),** but more often if clinically indicated.

Protocol for collection of sputum samples in the follow up of Drug Resistant TB patients:

- Fill in the Laboratory Request Form;
- Adult patients should be instructed to collect 2 sputum samples **(two spot samples**): Both samples should then be sent to the laboratory in compliance with SOPs.
- If an on-site laboratory is not available, the sputum samples should be stored to await transportation to the NRL for culture only or culture and DST.
- Following smear microscopy, results should be reported to the requesting Health Facility.
- Follow up samples for culture and First Line DST will be done at the NRL.
- In the case of follow up samples for culture and second line DST: if there is growth, the isolate should be sent to the supra national laboratory in South Africa (South African Medical Research Council laboratory) for second line DST.

### Sputum transportation for culture and DST

Given the risks associated with transport of specimens and/or cultures from patients suspected of having DR-TB, guidelines are provided in the laboratory manual for safe packaging and transportation of the samples.

- In transporting sputum samples or isolates for second line DST to the Supra National Reference Laboratory (SNRL) in South Africa, regulations recommended by the Universal Postal Union, the International Civil Aviation Organization and the International Air Transport Association should be strictly adhered to.
- Health Care Workers and drivers should be trained on the guidelines for safe handling of infectious substances.
- Sputum samples will be picked from Diagnostic sites twice a week and dropped at the NRL. Samples for second line DST will be picked from the NRL once a week and sent to the South African Medical Research Council (MRC) laboratory using contracted commercial courier.
- The cold chain requirements should be maintained during sample transportation and storage to reduce on bacterial contamination during cultures.

## Monitoring XDR-TB treatment

Patients being managed for XDR-TB should be monitored through both clinical and bacteriological assessments much the same way as MDR-TB patients.

All patients receiving XDR-TB treatment should be monitored monthly using both sputum smear and mycobacterial culture. Second line DST should be requested every three months.

## Monitoring for adverse effects during treatment

Close monitoring of patients is necessary to ensure that the adverse effects of second-line drugs are recognized quickly by health-care personnel. The ability to monitor patients for adverse effects daily is one of the major advantages of DOT over self-administration of MDR-TB treatment.

The majority of adverse effects are easy to recognize. If well-informed, patients will indicate when they are experiencing adverse effects. However, it is important to have:

- a systematic method of patient interviewing since some patients may be reticent about reporting even severe adverse effects. (This is important in view of the fact that some patients may be distracted by one adverse effect and forgets to tell the health-care provider about others).
- All DOT workers, hospital; clinic or community health worker trained to screen patients regularly for symptoms and signs of common adverse effects; management of adverse effects and when to refer cases to a higher level. Some of the common adverse effects symptoms include (see section 7.3 for details)
- Fever
- rashes,
- gastrointestinal symptoms (nausea, vomiting, diarrhoea),
- psychiatric symptoms (psychosis, depression, anxiety, suicidal tendencies),
- jaundice,
- ototoxicity
- peripheral neuropathy
- symptoms of electrolyte wasting (muscle cramping, palpitations)
- Laboratory screening is invaluable for detecting certain adverse effects that are more occult.

The recommendations in **Table 6.1** are an estimate of the minimal frequency of essential laboratory screening. More frequent screening is advisable, particularly for high-risk patients.

**Nephrotoxicity:** is a known complication of the injectable drugs, both of the aminoglycosides and of capreomycin. This adverse effect is occult (not obviously noted by taking the history of the patient or by physical examination) in onset but can be fatal. The optimal timing for checking serum creatinine (creatinine clearance) is unknown, but we advise checking serum creatinine at least monthly. In addition, patients with a history of renal disease (including co-morbidities such as HIV and diabetes), advanced age or any renal symptoms should be monitored more closely, particularly at the start of treatment. An estimate of the glomerular filtration rate may help to further stratify the risk of nephrotoxicity in these patients.

**Glomerular filtration rate** (**GFR**): is the volume of fluid filtered from the renal (kidney) glomerular capillaries into the Bowman's capsule per unit time.

**Electrolyte wasting:** is a known complication of the anti-tuberculosis injectable drugs, especially capreomycin. It is generally a late effect occurring after months of treatment, and is reversible once the injectable drug is suspended. Since electrolyte wasting is often occult in the early stages and can be easily managed with electrolyte replacement, serum potassium should be checked at least monthly in high-risk patients, and in all those taking capreomycin.

**Hypothyroidism:** is a late effect provoked by PAS and ethionamide. It is suspected by clinical assessment and confirmed by testing the serum level of thyroid stimulating hormone (TSH). The use of these agents together can produce hypothyroidism in up to 10% of patients (3). Since the symptoms can be subtle, it is recommended that patients are screened for hypothyroidism with a serum TSH at baseline, 3 months, and then tested again every 6 months or sooner if symptoms arise. The dosing of thyroid replacement therapy should be guided using serum levels of TSH.

Table 7.1: Recommended laboratory monitoring schedule in MDR-TB treatment

| **Base Line** | **Follow up** |
| --- | --- |
| Sputum smear microscopy | Monthly |
| Sputum Culture | Monthly until conversion then 2 monthly |
| Drug susceptibility | If remain smear /culture-positive repeat DST after 3 months of treatment. Repeat DST at 6 monthly and thereafter. there is a positive culture after initial conversion  (Months 0, 4 and 8) |
| Renal Function Tests (Serum creatinine& K+) | Every two weeks for the first two months when initiating an injectable drug, then monthly while receiving injectable. Patients with baseline renal insufficiency should be monitored frequently. |
| Thyroid Stimulating  Hormone (TSH) | Every 3 months if receiving PAS and ETO and PTO:TSH is sufficient for screening for hypothyroidism; it is not necessary to measure hormone thyroid levels |
| Liver serum enzymes | To be monitored regularly in patients   - receiving pyrazinamide - HIV infected - patients at risk or with symptoms of hepatitis   Once every two months during continuation phase |
| HIV screening,CD4  Viral load monitoring | Repeat as clinically indicated (every 3-6 months)  Suspected ART failure. In ART naïve patients, virologic failure is defined as follows:  VL>400 copies/ml at six months  VL>50 copies/ml at 48 weeks  Repeated detectable VL (>1,000 copies/ml) on two consecutive measurements, one to three months apart, after prior undetectable VL |
| Pregnancy tests  (women of child bearing age) | Repeat as clinically indicated. All women of child bearing age should be provided with family planning. |
| Full Blood Count | Monthly or as clinically required  For HIV-infected patients on ART, monitor monthly initially and then as needed based on symptoms |

*Source: Guidelines for Programmatic Management of Drug-resistant tuberculosis. Emergency update 2008. WHO/HTM/TB/200.402, and Guidelines for antiretroviral therapy for the treatment of adults and adolescents. WHO 2010 revision.*

## Management of adverse effects

### General approach

Treatment of drug-resistant tuberculosis (DR TB) involves the use of multiple medications, and most patients will experience some difficulty tolerating them.

As the response of an individual patient cannot be predicted, medications should not be withheld because of fear of a reaction. Patients should be well-informed and engaged as partners in their own treatment.

Proper management of adverse effects begins with patient education. Before starting treatment, the patient should be instructed in detail about the potential adverse effects that could be produced by the prescribed drug regimen, and if and when to notify a health-care provider.

If the adverse effect is mild and not dangerous, continuing the treatment regimen, with the help of ancillary drugs if needed, is often the best option. In patients with highly resistant TB, a satisfactory replacement drug may not be available, so that suspending a drug will make the treatment regimen less potent. Some adverse effects may disappear or diminish with time, and patients may be able to continue receiving the drug if sufficiently motivated. The adverse effects of a number of second-line drugs are highly dose dependent.

Prompt evaluation, diagnosis and treatment of adverse effects are extremely important, even if the adverse effect is not particularly dangerous as this may influence adherence. Patients may have significant fear and anxiety about an adverse effect if they do not understand why it is happening. These emotions in turn may augment the severity of the adverse effect, as in the case of nausea and vomiting. Long periods of time without medical evaluation also promote feelings of isolation and abandonment by the health-care system.

The following approach is recommended:

- **Prior to initiating a treatment regimen, it is essential to discuss the benefits and risks of therapy and obtain informed consent.**
  - The patient should understand the need for treatment, the importance of each medication in the treatment regimen, and the possible side effects and toxicities.
- **Psychosocial support** is an important component of the management of adverse effects. This is one of the most important roles played by DOT workers, who educate patients about their adverse effects and encourage them to continue treatment. Patient support groups are another means of providing psychosocial support to patients.
- **Assure patients that every possible attempt to make their treatment as easy as possible will be made, but stress that having enough effective drugs in the treatment is essential to achieving a cure.**
  - While side effects will be addressed and treated as aggressively as possible, patients should be mentally prepared for likely discomfort that they may experience, and should brace themselves for the long road ahead.
- **Ensure measures to avoid breaks in treatment to maximize the effectiveness of treatment.**
  - Reported side effects of each patient should be given due attention. Most patients will be willing to continue medication despite symptoms when they understand the benefit of the medication, know that many of these symptoms improve after the first several weeks, and are assured that the HCWs are doing their best to evaluate and address their problems.
  - Express appreciation for the patient’s efforts to cooperate. This recognition often helps a patient to continue therapy.
- Permanent dose reduction or definitive elimination of a drug from the regimen is a serious step and should be considered only after all other possibilities have been exhausted, used as a last resort,
  - i.e., in cases of significant organ dysfunction or intractable symptom intolerance. Ideally, any drug eliminated should be replaced with an equally effective drug so as not to compromise the overall effectiveness of the regimen.
- It is often difficult to ascertain whether a given side effect is due to a single medication or the result of several drugs given simultaneously.
  - If after following the various recommended treatment schemes, the patient remains intolerably symptomatic, a dose reduction or elimination of one of the drugs may be necessary.
  - Reducing the dose of the most likely offending drug for one week to see whether the symptoms diminish or disappear; if symptoms persist, the drug is returned to its original dose and the same process repeated for the other drugs in the regimen, until all potentially responsible drugs have been tested. This should be done in a systematic manner. E.g. with cycloserine and ethionamide, for example, a patient may be completely intolerant at one dose and completely tolerant at a slightly lower dose. Unfortunately, given the narrow therapeutic margins of these drugs, lowering the dose may also affect efficacy, so every effort should be made to maintain an adequate dose of the drug according to body weight. Lowering the dose by more than one weight class should be avoided.
  - Pyridoxine (vitamin B6) should be given to all patients receiving cycloserine or terizidone to help prevent neurological adverse effects. The recommended dose is 50 mg for every 250 mg of cycloserine (or terizidone) prescribed.
  - Systematic dose reduction of multiple drugs simultaneously would be the next option.

### Management of specific conditions

#### Management of Nausea and Vomiting

While the majority of patients experience nausea and/or vomiting as an adverse effect during MDR TB therapy, these symptoms rarely prevent delivery of adequate therapy.

Symptoms should be controlled, and any medications lost due to vomiting should be recovered. Volume and electrolyte management is also essential if vomiting is significant.

The following approach is recommended in case of nausea and vomiting:

- Ask the patient’s opinion about which drug may be responsible;
  - Patients may have strong ideas about which medication is causing them problems. Their opinions should be respected (even if no change can be made).
- Encourage the patient to continue to take the medication and assure them that for most patients many adverse reactions lessen over the first several weeks and may become tolerable or resolve entirely.
  - If signs of dehydration (thirst, dry mouth, sunken eyes, low blood pressure, orthostasis, weakness). Aggressive hydration: Administer 1-2 liters of NaCl 0.9% over first 6 hours. Consider hospitalization
  - If jaundice, pruritis, right-sided abdominal pain, rule out Hepatitis
  - If vomiting blood or emesis with the appearance of coffee grounds, consider possible gastrointestinal hemorrhage.
- If the drug suspected of causing the symptoms is Ethionamide, para-aminosalicylate (PAS) or clofazimine, decrease the dose (ethionamide 250 mg, PAS 4 to 2 grams) to see if the lower dose is better tolerated.
  - Advise the patient that this is a test to determine which drug is causing side effects and that the drug dose will be increased back to therapeutic dose in a manner that will be better tolerated.
  - The dose of medication can then be gradually increased over the next 2 weeks. Both medications can be given in 2 or 3 doses over the day, which may improve tolerance. Many patients tolerate the higher dose of ethionamide better in the evening (ethionamide 250 mg in a.m., 500 mg at bedtime; or may only tolerate 500 mg at bedtime). The goal should be to increase the ethionamide dose to at least 500 mg daily and the PAS dose to at least 6 to 8 grams daily
- Treat gastritis or acid reflux. Proton pump inhibitors or H2-receptor blockers may be helpful in many patients.
- Minimize use of non-steroidal anti-inflammatory drugs (NSAIDs).
  - This may be difficult if the patient also has arthralgias and myalgias from medications. Acetaminophen may be helpful, but with caution in view of its potential risk of increasing isoniazid (INH) hepatotoxicity.
- Encourage hydration.
- Evaluate and monitor electrolytes, Urea and creatinine, and corrected any inbalance.
- Refractory nausea and vomiting may suggest the need for further investigation, including addressing the possibility of liver involvement e.g hepatitis.
- If patient can be treated ambulatory, administer anti-emetics or antacids prior to medication or as needed. Note: Antacids cannot be given within 2 hours of fluoroquinolones.

The following are some specific options (adult doses):

- **1^st^ option:** Metoclopramide 10 to 20 mg PO, IM or IV every 4 to 6 hours as needed, given 30 minutes before morning and/or afternoon dose of anti-TB drugs
- **2^nd^ option:** Promethazine 12.5 to 25 mg PO, IV, or PRN 30 minutes before the dose and every 6 hours as needed
- **3^rd^ option:** Ondansetron 8 mg PO 30 minutes before the dose and again 8 hours after the dose; for refractory nausea 24 mg 30 minutes before the dose can be tried

The medications should be spaced out during the day to lessen the pill burden, or administer the drug in three doses

***Pregnancy should be considered as the possible etiology of nausea and vomiting, especially if the symptoms occur after a period of initial tolerance****.*

### Management of Diarrhea

Diarrhea is characterized by frequent watery bowel movements. Since many patients use the term diarrhea to describe bowel movements that are more frequent or loose than normal, it is important to ascertain whether or not the stool is truly watery with frequency of more than three or four times a day.

PAS often causes diarrhea with the initiation of medication. Inform patients that diarrhea usually resolves or improves considerably after several weeks.

Fluoroquinolones can also cause loose stools or diarrhea, along with increased flatulence. This can improve, but may persist in part for the duration of therapy

In the case of **mild** **to moderate** diarrhea:

- Oral Rehydration Solution (ORS) should be administered as a first measure of tolerable; or
- Give Loperamide 4 mg stat dose orally, then followed by 2mg after each loose stool to a maximum of 10mg in 24 hours.
- The patients fluid intake and hydration status should be evaluated.

In cases with **severe** diarrhea:

- if accompanied by bloody stools, severe abdominal pain, or fever greater than 38.5 °C, consider other causes such as acute bacterial enteritis, or pseudo-membranous colitis related to fluoroquinolones and treat according to the diagnosis.
- The electrolytes status should be checked
- Administer ORS or IV fluids to maintain the right hydration status.

**A**

#### Management of Gastritis

Gastritis covers a broad spectrum of entities that induce inflammatory changes in the gastric mucosa. Anti-TB drugs may potentially cause such inflammations which may result in symptoms like gnawing, burning epigastric pain or distress, occasionally accompanied by nausea and/or vomiting. The pain may improve or worsen with eating.

In the case of patients presenting with symptoms of gastritis, the following should be observed:

- Administer anti-tuberculosis medications with small amount of food or after eating
- Evaluate possible gastrointestinal hemorrhage (blood or “coffee ground” emesis , black, tarry stools)
- Advise patient to avoid caffeine (coffee, tea, soda), cigarettes
- If symptoms occur in the morning, advise patients to eat before going to bed and sleep with head elevated;
- Administer gastric-acid suppressants such as omeprazole 20 mg, once a day, before breakfast; If no improvement, administer antacids: - Calcium carbonate for patients who need a calcium supplement (elderly, pregnant women, etc.), aluminum hydroxide helpful in cases with diarrhea, magnesium hydroxide may improve constipation.
- Advise patients to take fluoroquinolones at least 3 hours apart from antacids to minimize reduced fluoroquinolone absorption
- If no improvement, and patient is receiving Ethionamide and/or clofazimine, consider reduction in dose;
- IF REFRACTORY AND SEVERE SYMPTOMS, consider treatment for *Helicobacter pylori.*

#### Management of Hepatitis

Any signs or symptoms of hepatitis (including nausea, severe vomiting, scleral icterus, jaundice, dark urine, pale stool) merit immediate evaluation of serum liver tests.

The ALT (SGPT) is more specific for hepatocellular injury than the AST (SGOT). Elevations in the AST may indicate abnormalities in the muscle, heart, or kidney. If the ALT is elevated more than the AST, this is consistent with liver inflammation. When the AST is elevated more than the ALT, the possibility of alcohol-related elevation of the transaminase should be considered

- If the hepatocellular enzymes are less than 5 times the upper limit of normal and there is no evidence of jaundice, continue the medications using strategies for managing nausea and vomiting and observe carefully.
  - If symptoms continue, consider repeating liver enzymes again to exclude hepatotoxicity.
  - If the bilirubin is increased but the hepatocellular enzymes are only mildly elevated, this could still represent significant drug-induced liver injury.
  - An evaluation for causes of direct and indirect hyperbilirubinemia should be done, and hepatotoxic medications should be stopped.
- If the enzymes are more than 5 times the upper limit of normal, hold all potentially hepatotoxic medications. If at least 3 medications remain in the treatment regimen that are not hepatotoxic, then these can be continued. If not, then all anti-tuberculosis medications should be held.
  - Monitor the LFTs weekly.
- If liver enzymes plateau or revert to normal and symptoms resolve, anti-TB drugs may be restarted sequentially beginning with the agents least likely to be hepatotoxic: Cm or aminoglycosides, Ethionamide, Fluoroquinolone and Cycloserine.
  - Carefully observe for clinical reactions and repeat liver enzymes twice weekly until the medication has been taken for at least a week and enzymes are stable. The next medication can then be added to the regimen and monitored.
  - All remaining medications should be reintroduced in this manner.
- If reintroduction of a medication leads to clinical symptoms of hepatotoxicity and enzymes increase, stop that medication and eliminate it from the regimen.
- Even if a medication is identified as causing hepatotoxicity, reintroduce each additional medication one at a time, because in some instances, more than 1 medication may be responsible for the hepatotoxicity.
- Monitor liver enzymes at least monthly for the remainder of the treatment course.

***Rule out other etiologies, viral hepatitis, alcohol, and non antitubercuolsis drugs (e.g., antiepileptics,***

***acetaminophen, sulfa drugs, griseofulvin, ketoconazol, fluconazol etc.)***

#### Management of Peripheral Neuropathy

Peripheral neuropathy is characterized by symmetrical polyneuropathy in nearly all cases. The first symptoms are tingling, prickling, and burning in the balls of the feet or tips of the toes. With further progression, sensory loss can occur. Ankle reflexes may be lost and weakness of dorsiflexion of the toes may be present. Symptoms may progress centripetally and also involve the fingers and hands. Unsteadiness of gait may develop due to proprioceptive loss.

The diagnosis can usually be made clinically. The drugs most commonly implicated are INH, ethionamide, cycloserine, and linezolid. Fluoroquinolones and ethambutol have rarely been associated with the development of neuropathy.

Pyridoxine prophylaxis (100 mg daily) should be included for all patients (including a weight proportionate dose for children) receiving treatment for MDR-TB who take INH, ethionamide, cycloserine, or linezolid, but especially those taking ethionamide and/or cycloserine.Prescribe 50 mg of pyridoxine for every 250 mg of cycloserine used.

If symptoms develop or progress, doses of 150 to 200 mg may be tried. Caution should be exercised with individuals with end-stage renal disease, as pyridoxine may develop toxic levels in these cases and cause neurologic symptoms

Additional interventions include:

- Correct nutritional deficiencies.
- Address additional medical problems (diabetes, alcoholism, vitamin deficiencies, HIV, hypothyroidism, uremia, other drugs, etc)
- Evaluate and correct electrolytes.
- Identify and change other medications that may cause peripheral neuropathy, if possible.
- Physical therapy may be helpful but is often not available.
- NSAIDs or acetaminophen may be helpful.
- A low dose of tricyclic antidepressant, amitriptyline 25 mg PO at bedtime can be tried if there are no contraindications**.** The dose of amitriptylinemay be increased (25 mg each week to 150 mg maximum) if lower doses are not helpful. (Linezolidcannot be given with tricyclic drugs or selective serotonin reuptake inhibitors [SSRIs]due to its mild monoamine oxidase (MAO) activity contributing to the risk of theserotonin syndrome.)
- Carbamazepine orally at 100 to 600 mg twice daily can be given.
- As Blood dyscrasias and elevated liver function may complicate therapy, a complete blood count (FBC) and liver function should be routinely monitored inpatients on this medication.
- Patients who fail to respond to a tricyclic may respond to gabapentin. Adults should be treated initially with a single dose of 300 mg PO on Day 1, increased to 300 mg twice a day on Day 2, and 300 mg 3 times a day on Day 3. The dose may be titrated up to 1800 mg as needed for relief. Gabapentin is also associated with a wide range of adverse effects, including nausea and vomiting, as well as arthralgias and CNS symptoms. Decrease dosage with renal insufficiency.
- Consider whether the dose of ethionamide or cycloserine can be reduced without compromising the regimen, and resume normal dose once pain controlled.
- If the patient is on Km and is known to be susceptible to Cm, consider changing the parenteral agent to Cm.

***Rarely, medication may be discontinued, but only if an alternative drug is available or the regimen is not compromised***

#### Management of Psychosis

Psychosis refers to a constellation of symptoms that reflect a disintegration of personality or a loss of contact with reality. Visual or auditory hallucinations, paranoia, catatonia, delusions, and bizarre behavior are hallmarks of psychosis.

Cycloserine is the medicine most commonly associated with psychosis; however, H, fluoroquinolones, and thiamides have also been associated with it. Other causes could be psychosocial stressors, depression, hypothyroidism, and other medications (benzodiazepines, certain antidepressants), as well as illicit drug and alcohol use.

Prior history of psychiatric disease is not a contraindication to the use of the above agents, though psychiatric side effects are more likely. Some patients may need anti-psychotic medication throughout the duration of anti-TB therapy, though side effects are generally reversible upon discontinuation of treatment.

Once psychosis has been diagnosed, other possible causes should be ruled out (especially in HIV positive patients with low CD4, consider Cryptococcosis!).

Twenty-four-hour surveillance and possible hospitalization should be considered for all patients with florid psychosis and/or suicidal or homicidal tendencies.

In the cases of acute psychosis:

- If the patient is at risk of harming himself/herself or others:
  - urgent hospitalization
  - Give haloperidol 2,5 mg orally or intramuscularly
  - If no improvement after 20 minutes give 2,5 mg and if no improvement after 20 minutes give 5 mg.
  - If no response and/or risk of harming herself or others, use Clorpromazine 25 mg intramuscularly (IM) up to 100 mg total dose until the patient is calm.
  - If good response, start haloperidol 2.5mg orally once daily and increase pyridoxine to 300 mg/day. Haloperidol may be increased by 2.5mg per day to control symptoms, to a maximum dose of 10 mg orally per day.
  - As risperidone is as effective and causes less extra-pyramidal effects than haloperidol, can be used instead of haloperidol: start with 0.5mg to 5mg twice or three times per day. The usual dose is 2-10 mg per day.
  - **Note:** that Haloperidol has anticholinergic as well as antidopaminergic effects. If patient develops symptoms of neuroleptic syndrome, must discontinue haloperidol immediately.
  - If patients develop dystonia, Parkinsonism, or EPS, administer with diphenhydramine 25 mg PO QD or biperiden or benzotropine
  - Withhold Cycloserine or Terizidone, and reintroduced with careful observation when symptoms resolve.
  - If no alternative drug is available, cycloserine may be tried at low dose. If any recurrence of psychotic behavior occurs, promptly and permanently discontinue cycloserine.
  - When the patient has stabilized, all medications have been successfully restarted, and all symptoms have resolved, the antipsychotic drugs can be tapered with careful observation of the patient.

#### Management of seizures

Seizure is an episode of neurologic dysfunction caused by abnormal neuronal or electrical activity of the brain that results in a sudden change in behavior, sensory perception, or motor activity. The clinical spectrum of seizures includes simple and complex focal or partial seizures and generalized seizures.

Certain anti-TB drugs have been associated with seizures. However, prior history of seizure is not a contraindication to DR-TB treatment if the condition is well controlled on anti-convulsive therapy. Seizures are not a permanent sequel of treatment

The goal of seizure management is to stabilize the patient during an acute episode and the prevention of seizure recurrence.

In general, clinical evaluation is sufficient unless there is suspicion for infectious, malignant, vascular, or metabolic cause is high. However the following should be observed:

- Rule out other likely causes for seizure (e.g. meningitis, encephalitis, illicit drug use, alcohol withdrawal, hypoglycemia, hyper- or hyponatremia, hyper- or hypocalcemia, cerebrovascular accident, or space-occupying lesion)
- Consider checking blood chemistries and laboratory studies (including serum liver tests, urea, creatinine, glucose, electrolytes, calcium, HIV serology);
- Suspend cycloserine and isoniazid if patient is receiving these medications
- Consider suspension of fluoroquinoline

If actively fitting:

- Place the patient in the lateral decubitus position, remove objects nearby that can cause danger for the patient, protect the tongue with a soft object too large to be swallowed, observe until patient stop seizing.
- Ensure airway is protected and not obstructed;
- Give diazepam 5 mg intravenously or intramuscularly immediately, followed by a loading dose of phenytoin (20 mg/kg intravenously, diluted in 200 ml ClNa 0,9%,and not 5% Dextrose solution). Diazepam may be repeated once in 10 minutes if seizures do not cease.
- Monitor the patient carefully for signs of respiratory depression.

If the seizure has already stopped at the time of initial evaluation and the patient is postictal:

- do not give diazepam but give phenytoin loading dose as described above.
  - In both instances, begin phenytoin maintenance dose of 300 mg/day (3-5 mg/kg/day) once the loading dose has been administered.

If seizures recur:

- Phenytoin may be increased to a maximum of 500 mg/day or a second agent (valproic acid, phenobarbital) may be added.
- Increase pyridoxine to 300 mg/day in all cases.
- Initiate anti-epileptic treatment for the remainder of MDR TB therapy
  - Phenytoin (3-5 mg/kg/d) - Potential adverse effects: ataxia, incoordination, confusion, skin rash, cerebellar dysfunction, hepatotoxicity, gingival hyperplasia, lymphadenopathy, hirsutism. Levels increased by H, R, FQs.
  - Valproic acid (750-1250 mg/d) - Potential adverse effects: ataxia, sedation, tremor, hepatotoxicity, bone marrow suppression, GI upset, weight gain
  - Carbamazepine (600-1200 mg/d) - Potential adverse effects: ataxia, dizziness, diplopia, vertigo, GI upset, hepatotoxicity, skin rash
  - Phenobarbitol (60-120 mg/d) - Potential adverse effects: sedation, ataxia, confusion, dizziness, decreased libido, depression, skin rash. - Enhances metabolism of other drugs, including H.
  - Once stabilized, consider reinitiating the suspected drug at lower dose

#### Hypothyroidism

Hypothyroidism may be asymptomatic but commonly manifests as a slowing in physical and mental activity. Symptoms and signs of this disease are often subtle and neither sensitive nor specific. Classic signs and symptoms, such as cold intolerance, puffiness, decreased sweating, and coarse or dry skin, muscle pain, emotional lability, impaired memory, blurred vision and hoarseness of voice.

Individuals can also present with obstructive sleep apnea (secondary to macroglossia) or carpal tunnel syndrome. Women can present with galactorrhea and menstrual disturbances. Consequently, the diagnosis of hypothyroidism is based on clinical suspicion and confirmed by laboratory testing.

Some second line anti-TB medications are associated with causation of hypothyroidism of which the likely agents include: PAS, Prothionamide and Ethionamide (particularly when given in combination).

When hypothyroidism is suspected:

- Assess baseline thyroid function prior to start DR TB treatment and correct if needed.
- Assess thyroid function every 6 months unless clinical assessment indicates the need to evaluate sooner.
- Conduct monthly clinical assessments for hypothyroidism.
  - Clinical assessments may be a better indicator of thyroid function than laboratory values.
  - Enquire about fatigue, weakness, cold intolerance, decreased appetite, constipation, loss of energy, depression, and inability to concentrate.
  - Check physical signs e.g. enlarged thyroid, dry skin, coarse hair, and weight gain.
- Check TSH level if suggestive symptoms or signs are present.
  - If TSH level is greater than 10, then symptomatic hypothyroidism is likely and therapy should be given.
  - Levothyroxine therapy should be initiated at a dose of 0.050 mg daily (or 0.025 mg daily for patients older than 65 years), increasing the dose by 0.025 mg and checking a TSH level every 4 weeks until a normal level is attained.
  - Thereafter TSH should be checked every 4 months until the patient's course of anti-TB therapy has been completed. If TSH testing not available, discontinue levothyroxine after two to three months and follow symptoms
  - If symptoms do not improve, lower Eto dose by 250 mg or decrease PAS to 4 gm once daily. Discontinue the drug(s) if above measures are ineffective and equally effective drug can be substituted.
  - Continue to follow TSH until the treatment is completed, and discontinue levo-thyroxine according to TSH results;
  - ***Note:*** do not give levothyroxine at same time as antacids or phenytoin, as these impair GI absorption
- Hypothyroidism is reversible upon discontinuation of PAS and/or Eto, i.e., the TSH level is expected to normalize after 2-3 months.

#### Management of myalgia and arthralgias

Pain and tenderness of the muscles and joints are relatively common side effects associated with a variety of drugs used to treat drug-resistant TB patients. One or more of the following drugs may be implicated: PZA, fluoroquinolones, rifabutin, INH, and ethionamide.

Electrolyte disturbances associated with the aminoglycosides and Capreomycin may also cause muscle pain and cramping. Hypothyroidism may also contribute.

Serum uric acid levels may be elevated, but this is of little clinical relevance and anti-hyperuricemic therapy is of no proven benefit in these patients.

Therefore:

- Do not discontinue medications.
- If moderate to severe, NSAIDs may be given (indomethacin 50 mg orally twice daily or ibuprofen 400-800 mg orally thrice daily).
- If mild, Paracetamol 500-1,000 mg orally 2- 4 times daily may provide some relief
- If acute swelling, erythema, and warmth are present, evaluate for the presence of inflammatory diseases:
  - Aspirate joint for diagnosis if fluid is present
  - Evaluate for infection
  - Evaluate for hypothyroidism or hyperthyroidism.
  - Draw serum electrolytes, calcium, and magnesium. Correct deficiencies.

#### Management of electrolyte abnormalities

All of the aminoglycosides and capreomycin can cause electrolyte disturbances due to renal tubular wasting of potassium, magnesium, and calcium salts. These effects are most pronounced with capreomycin. Chloride and hydrogen losses may also occur with resulting alkalosis. A defect in renal tubular resorption of chloride may be caused by these drugs. Nausea, vomiting, and diarrhea may also contribute to electrolyte abnormalities.

Although often asymptomatic, low serum potassium and magnesium may present as fatigue, myalgias, cramps, paresthesias, lower extremity weakness, behavior or mood changes, somnolence, and confusion. More severe disturbances can lead to tetany, paralysis, and life-threatening cardiac arrhythmias

***Hypokalemia – Hypomagnesemia:***

- Mild-to-moderate hypokalemia (i.e., 2.5 < K < 3.5 mEq/L, asymptomatic) and mild hypomagnesaemia (1.4 < Mg < 1.8 mg/dl, asymptomatic) can be treated with oral supplements, with repeat monitoring in 24-48 hours.
- Where it is not possible to measure magnesium, if a patient has hypokalaemia it should be assumed that he also has some degree of hypomagnesaemia. Untreated hypomagnesaemia may lead to a syndrome of "resistance" to correction of hypokalemia.
- Severe hypokalemia (K < 2.5 mEq/L or symptomatic) and moderate-to-severe hypomagnesaemia (Mg < 1.4 mg/dl or symptomatic) should be treated with parenteral or combined parenteral/oral supplementation, with repeat monitoring in 6-24 hours depending on the severity of the symptoms.

Table 7.2: Blood Potassium levels and recommended monitoring protocol

| Potassium level meq/L | Quantity of KCL | When to do next control (sooner if patient has vomiting or diarrhea) |
| --- | --- | --- |
| 4.0 or more | None | Monthly |
| 3.7 – 4.0 | None | Monthly |
| 3.4 – 3.6 | 20 - 40 meq | Monthly |
| 3.0 – 3.3 | 60 meq | Two weeks |
| 2.7 – 2.9 | 80 meq | One week |
| 2.4 – 2.6 | 60 meq IV and 80 meq orally every 6 hours for 24 hours |  |
| 2.0 - 2.3 | 60 meq IV and 100 meq orally | Every 6 hours with aggressive IV replacement. Consider holding injectable until >2.4 |

*Source: Guidelines for Programmatic Management of Drug-resistant tuberculosis. Emergency update 2008. WHO/HTM/TB/200.402.*

**Note:**

- Every pill of Slow K 600 mg contain 8 meq of K
- Every pill of Magnesio 150 mg Magnesium oxide
- **Potassium IV Supplementation**: should NOT exceed more than 20 meq/hr of KCl. Normal preparation is 40 meq in 1 liter of NaCl 0.9%, maximum preparation is 60 meq/L.
- **Magnesium IV Supplementation**: maximum concentration: 5 g or 40 meq MgSO4 in 1 liter of NaCl 0.9% or dextrose 5%. Do NOT exceed 150 mg per minute.If not emergency:2 g in 100 ml administered over 1–2 hours or4 g in 250 ml administered over 2–4 hours
- Amiloride or spironolactone are useful in resistant cases .

***Hypocalcemia:***

Symptomatic hypocalcemia should be treated on an emergency basis with 2 grams of calcium gluconate (180 mg elemental calcium or 20 ml 10% calcium gluconate) IV over 10 minutes, followed by infusion of 6 grams calcium gluconate in 500 ml D5% over 4–6 hrs.

- The IV infusion should be tapered.
- The initial oral dose during the transition from IV to oral therapy is 1–2 g elemental calcium three times a day.
- For long-term therapy the typical dose is 500mg to 1g orally administered thrice daily.
- Hypomagnesemia must be treated if present.
- Total serum calcium levels need to be adjusted for low albumin (ionized levels of calcium do not need to be adjusted). The total serum calcium can be corrected by adding 0.8 mg/dL for every 1 g/dL decrease of serum albumin below 4 g/dL. By doing this calculation one can determine if true hypocalcemia is present: Corrected calcium for hypoalbuminemia = 0.8(4.0 -measured albumin) + reported calcium

#### Management of nephrotoxicity

All of the aminoglycosides and capreomycin can cause nephrotoxicity. Ongoing assessment of renal function is therefore important.

Concurrent medical conditions such as diabetes or chronic renal failure are not a contraindication to DR-TB treatment, though greater caution must be exercised in such circumstances.

**Acute renal failure**: Creatinin clearance < 30 ml/min

- Suspend nephrotoxic medications (S, KM, AMK, CM);
- Check electrolytes including K, Mg and HCO3. Consider checking Ca and phosphorus.
- Rule out other causes of renal failure (e.g., diabetes, dehydration, congestive heart failure, urinary obstruction, urinary tract infection, prostatic hypertrophy, other medications such as NSAIDs, ACE inhibitors, sulfa drugs, diuretics)
- **Treatment**
  - Follow serum urea and creatinine and clinical exam for signs of improvement;
  - Consider inpatient management (hospitalization) in patients with severe renal failure;
  - Treat symptoms, fluid and electrolyte disturbances as needed;
  - Follow up for clinical improvement and normalization of serum urea and creatinine prior to considering reinitiation of injectable medications;
  - Once symptomatic improvement and documented stabilization of renal function; if the patient is receiving an aminoglycoside, it should be substituted with Capreomycin if infecting strain is susceptible;
  - If change to CM cannot be made, reduce dose of injectable according to creatinine clearance or replace with equally efficacious oral anti-tuberculosis drug if possible;
  - In the case of severe renal failure, discontinue all nephrotoxic medications and replace with equally efficacious oral anti-tuberculosis drugs if possible;
- **Adjust dose of all TB medications according to creatinine clearance**
  - Follow serum urea and creatinine every 2- 4 weeks thereafter;
  - Maintain close surveillance for treatment failure and/or resistance amplification if there is a period of irregular therapy during acute management

#### Management of ototoxicity

Ototoxicity refers to damage of cranial nerve VIII, usually manifested by hearing loss and/or tinnitus. Other vestibular symptoms such as nystagmus, ataxia, and disequilibrium can also occur.

All of the aminoglycosides and capreomycin are toxic to the eighth cranial nerve and can cause both vestibular and auditory toxicity.

Although hearing loss is irreversible, progression can be prevented once the offending agent is discontinued. However, continuation of injectable therapy despite hearing loss may be warranted in patients with significant resistance and/or disease. In such cases, capreomycin may replace an aminoglycoside agent if the infecting strain is susceptible. Using the injectable three times a week can also be considered.

**In Vestibular toxicity:**

- Observe the patient closely,
- If tinnitus and unsteadiness develop and these are attributed to vestibular toxicity, reduce or stop the injectable agent, according the severity of the symptoms.
  - This is one of the few adverse reactions that cause permanent intolerable toxicity and, sometimes, necessitate discontinuation of a class of agents. Drug induced vestibular toxicity is not reversible;
- At least monthly, assess vestibular toxicity;
- Fullness in the ears and intermittent ringing in the ears are early symptoms of vestibular toxicity.
  - When these are reported, it is sometimes possible to change the dosing to 2 or 3 times a week and continue the injectable agent for another month or more;
- Watch the patient carefully. Toxicity is related to the total dose and is cumulative. It is impossible to predict for an individual patient what dose is tolerated;
- A degree of disequilibrium can be caused by cycloserine, fluoroquinolones, ethionamide, INH, or linezolid.
  - Prior to stopping the injectable agent, evaluate whether these and/or other medications are causing the symptoms.
  - Stopping the injectable should be done after carefully excluding other causes of the symptoms.
  - Other drugs or all drugs can be held for several days to see if the symptoms improve. Symptoms of vestibular toxicity generally do not improve with holding medication.

**Auditory Toxicity**

Some degree of loss occurs in nearly all patients treated for drug-resistant TB. High-frequency loss usually occurs first. The effects are cumulative, and hearing loss may be reversible or permanent.

In case of suspected auditory toxicity:

- Perform a baseline audiogram and repeat monthly, for patients on IP;
- Consider change of the injectable to 3 times a week, after 3 to 4 months, when the cultures are negative;
- Avoid loop diuretics (furosemide) because they increase eighth nerve toxicity;
- Streptomycin has less auditory toxicity, but has more vestibular toxicity;
- Resistance to streptomycin is common and should be excluded before substituting it for another injectable.

Some patients must tolerate significant hearing loss in order to achieve a cure of their drug-resistant TB. The decision to continue therapy with an injectable when significant hearing loss occurs should be discussed with the patient and relatives.

Table 7.3: Common adverse effects of second line anti-TB drugs and suggested management strategies.

| **Adverse Effect** | **Drugs** | **Recommendations** | **Treatment** |
| --- | --- | --- | --- |
| Nausea, vomiting and  gastrointestinal upset | PAS, Eto/Pto, Clofazimine,  H, E, Z | - Take medications with food or before going to bed - Assure proper hydration - Antiacids should not be taken at the same time as TB meds (especially if R is kept) - To improve tolerance Eto can be given in 2 doses during the first 2 weeks - Consider if it can be the first sign of hepatotoxicity | *Metoclopramide* 0.1 – 0.2 mg/kg 30’ before medication  *Promethazine* (not to be used in less than 2 years old)??  >2y 0.25-0.5mg/kg (max: 25 mg/dose) every 4—6 hours  *Ondasentron* 0,125mg/kg  *Omeprazol*  5- <10kg 5mg OD  10 to <20kg 10mg OD  >20kg 20mg OD  *Ranitidine* 2-8mg/kg/day in 2 doses |
| Diarrhoea | PAS, Pto / Eto | If fever and bloody stools consider bacterial infection. | |
| Drug-induced hepatitis | Z, H, R,  Pto / Eto,  Fluoroquinolones | - Consider other causes: viral hepatitis, substance abuse - If severe affection stop all TB drugs.   Reintroduce gradually | |
| Dermatitis | Any agent can cause it | - Rule out other causes: scabies, atopia, etc - If severe rash, discontinue treatment - Avoid dry skin | *Chlorpheniramine*  2y-6y: 1mg tds/qid  Max 6mg/day  >6y-12y: 2 mg tds/qid  Max 12mg/day  >12: adult dose |
| Seizures | Cs, H, and Ofx, Lfx and Mfx | - Differential diagnosis with tuberculoma, toxoplasma, existing seizure problem - Stop suspected drugs. If not possible, maintain anticonvulsants until the end of the use of the offending drug | *Phenytoin:* 5 mg/kg/day bd/tds  Max 300mg/day  *Valproic Acid:* initial dose may be 5-10 mg/kg per day, bid or tid  *Carbamazepine*  *<6y: Initial:* 10-20 mg/kg/day bid or tid as tablets. Increase weekly to achieve optimal clinical response .  >6y***:*** *Initial:* 100 mg b.i.d. Increase at weekly intervals by adding up to 100 mg/day using a t.i.d. or q.i.d. until the optimal response is obtained.  Max dose: 1000 mg daily. Maintenance: Adjust dosage to the minimum effective level, usually 400-800 mg daily.  *Phenobarbital*: 1-6 mg/ |
| Psychosis or depression | Cs, fluoroquinolones  Ethionamide and H. | - Differential diagnosis with existing conditions and social factors   Psychosocial support strongly recommended in children | |
| Peripheral Neuropathy | S, Km, Cm, H, FQ, Cs, and E | Pyridoxine: 1-2 mg/kg.  Give 50 mg for every 250 mg of cycloserine/terizidone | |
| *Optic Neuritis (*Visual impairment) | Mainly Ethambuthol  Rarely Cs, Pto/Eto, linezolid, | In case it’s suspected, stop Ethambutol permanently | |
| Hearing loss  vestibulo-cochlear toxicity  (dizzness, vertigo, ataxia) | S, Km,Am and Cm, | - Hearing loss is related to the total dose and generally not reversible upon discontinuation of therapy. - Baseline audiometry is strongly recommended (especially for those children exposed to Streptomycin) and monthly thereafter, up to 6 months after stopping injections. - Consider reducing the dose (3 days per week) or discontinuing the agent provided this does not compromise the effectiveness of the regimen. | |
| *Arthralgia, arthritis* | Fluoroquinolones, Z (very frequent) | - Rule out other causes of arthralgia: arthritis, osteomyelitis and TB osteomyelitis. - Encourage some physical exercise - Check that patient is receiving correct doses of PZA/fluoroquinolone | *Ibuprofen* 5-10mg/kg/dose every 6-8h  *Paracetamol* 10-15mg/kg/dose every 6-8h |
| *Hypothyroidism* | PAS, Pto / Eto  (particularly when given in combination) | - Keep a high index of suspicion - If possible monitor TSH | *L-thyroxine* Children with severe hypothyroidism should be started at 25 mcg/day; adjust dose by 25 mcg every 2-4 weeks. In older children, hyperactivity may be decreased by starting with 1/4 of the recommended dose and increasing by 1/4 dose each week until the full replacement dose is reached. Refer to adult dosing once growth and puberty are complete **0-6 months**: 8-10 mcg/kg/day **6-12 months**: 6-8 mcg/kg/day **1-5 years**: 5-6 mcg/kg/day **6-12 years**: 4-5 mcg/kg/day **>12 years**: 2-3 mcg/kg/day |
| *Renal Failure* | Rifampicin  Aminoglycosides and Cm | Assure correct fluid intake in small children.  Management follows the same principles as adults | |

*Source: Adapted from Guidelines for Programmatic Management of Drug-resistant tuberculosis. Emergency update 2008. WHO/HTM/TB/200.402.*

**Note:** For the detailed algorithm for management of common side effects, please refer to the respective annexes.

Table 7.4: Commonly used ancillary medications in managing side effects of second line anti-TB drugs:

| **Indication** | **Drug** |
| --- | --- |
| Nausea, vomiting, abdominal upset | ***Metoclopramide***, dimenhydrinate, prochlorperazine, ***promethazine***, bismuth subsalicylate |
| Heartburn, acid indigestion, sour stomach, ulcer | H2-blockers (***ranitidine***, cimetidine, etc.), proton pump inhibitors (***omeprazole***, , etc.) Avoid antacids because they can decrease absorption of flouroquinolones |
| Oral candidiasis (non-AIDS patient) | ***Fluconazole, clotrimazole lozenges*** |
| Diarrhoea | ***ORS, Loperamide where necessary*** |
| Depression | Selective serotonin reuptake inhibitors (***fluoxetine***,), tricyclic antidepressants (***amitriptyline***) |
| Severe anxiety | ***diazepam, clonazepam*** |
| Insomnia | ***Dimenhydrinate*** |
| Psychosis | ***Haloperidol***, , risperidone (consider ***benzotropine*** to prevent extrapyramidal effects) |
| Seizures | Phenytoin, ***carbamazepine***, valproic acid, phenobarbital |
| Prophylaxis of neurological complications of cycloserine | ***Pyridoxine*** (vitamin B_6_) |
| Peripheral neuropathy | Amitriptyline |
| Vestibular symptoms | Meclizine, , prochlorperazine, promethazine |
| Musculoskeletal pain, arthralgia, headaches | Ibuprofen, paracetamol, paracodeine |
| Cutaneous reactions, itching | Hydrocortisone cream, calamine, caladryl lotions |
| Systemic hypersensitivity reactions | Antihistamines (diphenhydramine, chlorpheniramine, dimenhydrinate), corticosteroids (prednisone, dexamethasone) |
| Bronchospasm | Salbutamol, Inhaled beta-agonists (salbutamol, albuterol, etc.), inhaled corticosteroids (beclomethasone, etc.), oral steroids (prednisone), injectable steroids (dexamethasone, methylprednisolone) |
| Hypothyroidism | Levothyroxine |

*Source: Adapted from Guidelines for Programmatic Management of Drug-resistant tuberculosis. Emergency update 2008. WHO/HTM/TB/200.402.*

## Management of irregular and noncompliant patient

The use of directly observed therapy (DOT) in the treatment of MDR TB is required. Even with DOT, however, there are still patients who are irregular or noncompliant. Such patients present a challenge to the DOT worker, nurse, and physician.

The following approach should be observed:

- At the first sign that a patient is not complying with the treatment regimen, the medical team should analyze the problems responsible for the patient’s non-compliance.
- Arrange a doctor’s visit with the patient, DOT worker, and nurse to discuss in detail why the patient is missing doses. If the missed doses are due to a scheduling problem, often accommodations can be made (e.g., delivering a dose at work).
- Careful attention must be paid to the development of psychiatric symptoms.
  - Psychological support is often helpful, either in the form of an appointment with a psychiatrist or counselor or through participation in group therapy.
  - If patients have severe symptoms of depression or psychosis and a psychiatrist is not available, the doctor taking care of the patient may need to start psychiatric medicines until a psychiatrist is available. Often doses are missed because side effects are not being adequately addressed. If this is the case, more aggressive side effect control is indicated.
- Patients should be assessed for alcohol and drug abuse. Although such behaviors are difficult to modify, education and counseling on addiction may be helpful. In addition, support groups and twelve step programs, such as Alcoholics Anonymous, have a proven record of helping such patients and should be used in smear-negative patients when available.
- Enablers and incentives may be used to improve adherence.
  - The difference between an enabler and an incentive is that an enabler allows the patient to comply with treatment (for example, money for a bus to come to the clinic) while an incentive rewards the patient for being compliant e.g a food basket at the end of a week of regular treatment, and clothing;
- When all means of facilitating adherence have been exhausted with no result, the patient is asked to sign a contract with rules regarding adherence (e.g., no more than two missed doses in a one-month period).

### Abandoning or interrupting DR-TB treatment

When a patient shows tendencies to abandon therapy early, every effort should be made to explain the importance of completing the full therapeutic regimen.

The reasons behind the patient’s attitude of wanting to stop treatment should be adequately discussed with patient, and, if possible, try to improve any difficult situations that may be contributing to the patient’s desire to stop therapy. Timely and effective management of side effects or changes in scheduling time of doses can help improve the situation.

Change of the treatment regimen should be avoided as much as possible, as this can undermine the importance of taking all the medicines and may cause other patients to request similar changes.

As with the irregular patient, an evaluation should be done that includes an assessment of the patient for depression and/or substance abuse. Enablers and new incentives can be considered.

### Re-initiation of treatment

On rare occasions, the clinician may decide to reinitiate treatment in patients for whom therapy has been suspended due to noncompliance or in patients who are lost to follow up during therapy.

There are no concrete guidelines for the re-initiation of MDR TB therapy following treatment interruption. The following is a reasonable protocol that has been reached by consensus:

- Have the patient sign a new adherence contract (see above);
- Perform a full history and physical exam;
- Obtain a smear and culture;
- If positive, culture should be sent for DST;
- Obtain a radiograph and repeat the initial laboratory data;
- The treatment regimen and duration to be used for patients restarting therapy depends on the month at which the patient abandoned therapy and the clinical state at which the patient returns to therapy.
  - Patients who have been off therapy for longer than six months should be evaluated for active disease, and, if it is present, the patient should be started on a completely new course of treatment.
  - If no active disease is present, clinical judgment should be used to decide whether to reinitiate therapy.
  - If therapy is not restarted, the patient should be followed regularly for signs of relapse.

1. MANAGEMENT OF CHILDHOOD MDR-TB

## MDR-TB in children

MDR-TB in children is mainly the result of transmission of a strain of *M. tuberculosis* that is MDR from an adult source case, and therefore often not suspected unless a history of contact with an adult pulmonary MDR-TB case is known.

Children with DR-TB generally have primary resistance transmitted from an index case with DR-TB. Children who are contacts of index cases should be properly evaluated in line with NTCP protocol ***(See section 11.4)*.**

Children with clinical evidence of active TB disease and history of contact with a documented case of DR-TB with or without culture result should be treated based on the DST result of the index case.

Nevertheless, every effort should be made to confirm DR-TB bacteriologically by the use of DST and to avoid exposing children unnecessarily to toxic drugs. In general, anti-tuberculosis drugs should be dosed according to body weight **(*see Table 8.2*).**

Monthly monitoring of body weight is therefore especially important in paediatric cases, with adjustment of doses as children gain weight.

All drugs, including the fluoroquinolones, should be dosed according to the standardized dosage regime in the table.

Treatment failure in children is difficult to assess given the limitation of monitoring by culture result. Persistent abnormalities on chest radiograph do not necessarily signify a lack of improvement. Treatment failure should be suspected in children if:

- there is weight loss or,
- failure to gain weight (failure to thrive)
- deteriorating clinical condition or new signs and symptoms.

Children suspected of failure should be referred to the National TB Hospital or the nearest Regional MDR-TB Clinical management team. The child should be assessed appropriately for the worsening MDR-TB condition as well as other possible clinical entities.

Evidence suggests that adolescents are at high risk for poor treatment outcomes. Early diagnosis, strong social support, individual and family counseling and a close relationship with the medical provider may help to improve outcomes in this group.

### MDR-TB case finding in children

Due to the delay access to diagnose, children with MDR TB have poor outcomes, therefore early diagnose is essential to reduce morbidity and mortality:

- All children who are close contacts of known MDR TB patients should be screened for TB, with appropriate children screening tools.
- Any children screened positive for TB should be referred for further investigations:
- Chest X-Ray
- TST: A positive result is 5mm of induration in HIV positive children or 10mm of induration in HIV negative children.
- Sample collection for any rapid diagnosis method available (Gene Xpert, LPA) and culture/DST

In case a child is diagnosed with MDR TB and when a clear contact can’t be identified, reverse tracing is necessary, in order to identify the possible source case.

**For children less than 5 years old or HIV-infected irrespective of age, close follow up is recommended for a period of at least two years after the exposure**

## Diagnosis of MDR-TB in Children

Microbiological confirmation of DR TB in children is complicated as they usually have paucibacillary disease, samples other than sputum are difficult to obtain and those samples have a significant lower yield.

In order to diagnose DR TB we need to have a high index of suspicion, specially is a known/suspected DR TB contact and when there is poor response to treatment.

### When to suspect DR TB in children

*When starting TB treatment*

Most of the DR TB in children is transmitted from an adult case, so whenever we are initiating any child on standard TB treatment, we need to do a thorough assessment to identify possible DR TB contacts.

As most children (>90%) develop TB within 12 months of the primary infection, a DR TB contact case is especially significant in the last 2 years, always assess on the first visit:

- If there any active cases of TB with poor response (persistent symptoms, died while on treatment, receiving injections etc;
- Any known contact of MDRTB, currently or in the last 2-3 years;
- Any contact treated more than once for TB.

*During the follow-up of TB treatment*

DR-TB should be suspected if there is poor clinical response:

- Persistent TB symptoms: cough, fever, night sweats, reduced playfulness, poor appetite…
- Poor weight gain or failure to thrive for 2 or 3 months.
- Worsening of the chest X-ray.

If there is suspected DR-TB and no clear contacts, we need to assess the social situation to verify that the child has been taking the TB medication correctly and identify possible causes of poor adherence.

**Whenever DRTB is suspected, all efforts have to be made to obtain a sample for smear, Xpert MTB/Rif and culture/DST to confirm the diagnosis.**

The best ways to obtain respiratory specimens are given in the table below:

Table 8.1: Techniques and methods for obtaining respiratory specimens from Children

|  | **Gastric Aspirate** | **Nasopharyngeal Aspirate** | **Induced Sputum** |
| --- | --- | --- | --- |
| **Yield** | 30-40% for 3 GA, increased in younger and sicker children | Similar yield reported to induced sputum | 1 sputum same yield as 3 GA |
| **Specimen collected** | Gastric fluid | Upper secretions (lower secretions can be obtained if the child coughs) | Lower respiratory secretions |
| **Characteristics** | Invasive, doesn’t needs special equipment | Simple, non-invasive, but needs special equipment | Needs special equipment and well ventilated areas |

Any respiratory specimen should be sent for smear, GeneXpert and culture/DST (as per National guidelines)

In case extra pulmonary DR TB is suspected, any specimen obtained should be sent for culture: cerebrospinal fluid for TB meningitis, lymph node biopsy, and swabs in draining sinuses)

A lack of a positive DR TB sample, doesn’t exclude DR TB. If clinical suspicious remains, refer/consult a TB specialist to evaluate access to empiric treatment.

**Diagnosing drug-resistant TB in children:**

- Isolating *M. tuberculosis* and demonstrating drug resistance on DST remains the only way to confirm DR TB.
- *Probable* DR TB can be diagnosed when a child with TB reports recent close contact with and adult with DR TB.
- DR TB should be suspected in any child who fails to improve while adherent to firs-line and anti-TB therapy or if the adult source case is a treatment failure, a retreatment case or recently died from TB.
- Although children usually develop transmitted DR TB, some children develop lung cavities with high bacillary loads and may acquire drug resistance if the treatment regimen is inadequate, supply of drugs is irregular or treatment adherence is poor.

## Treatment of Paediatric MDR-TB

Treatment childhood DR-TB is usually difficult and most of the recommendations are based on studies done in adults or experts advice. Health Care workers should therefore seek specialist advice or refer to a specialist centre in the event of any difficulty with initiating DR-TB treatment in children. However, the ttreatment of pediatric MDRTB follows the same principles than adult protocols, in terms of drugs and duration.

Generally, Children with MDR-TB should be treated with the first-line drugs to which their *M. tuberculosis* strain (or that of their source case) is susceptible, including pyrazinamide. Ethambutol is bactericidal at higher doses, so daily doses up to 25 mg/kg should be used in children being treated for MDR-TB.

The specific principles to be considered in the treatment of childhood MDR-TB include the following:

- Treat the child according to the drug susceptibility pattern (and using the treatment history) of the source case’s M. tuberculosis strain if an isolate from the child is not available. If a DST result is obtained once on treatment, the regimen will be tailored to the new results. (This is particularly important where rapid DST test cannot be obtained for the Child due to inability to produce sputum).
- Use at least 5 drugs to which the patient is susceptible from the different groups.
  - If the child received previously treatment for more than 1 month on a failing regimen, it’s unlikely that those drugs will be effective any more
- Total treatment should be 20 months of which the initial intensive phase should be 6 months;
- Do not add a single drug to a failing regimen.
- Pyrazinamide should be maintained whenever possible.
- Use if possible Amikacin from the group 2, as it has fewer side effects. Injectables should be administered for 6 months unless there are positive cultures during this period.
- Treatment should be administered once daily whenever possible;
- Directly observed therapy (DOT) is essential, and should be actively encouraged;
- Counsel the child’s caregiver at every visit, to provide support, advice about adverse events and the importance of compliance and completion of treatment.
- Follow-up is essential: clinical, radiological and bacteriological (mycobacterial culture for any child who had bacteriologically confirmed disease at diagnosis). Monthly smear microscopy and cultures should be done until confirmed negative on 3 consecutive occasions.
- In children with a negative culture on initiation, monthly cultures are recommended during the 6 first months.
- With correct dosing, few long-term adverse events are seen with any of the more toxic second line drugs in children, including ethionamide and the fluoroquinolones.

### Second line drugs for MDR-TB treatment in Children

Table 8.2: Second line drugs for MDR-TB treatment in Children

| **Drug group** | **Drug name** | **Daily dose (mg/kg)** | **Frequency** | **Maximum daily dose** |
| --- | --- | --- | --- | --- |
| Group 1 | Rifampicine (R) | 10-20 | Once daily | 600 mg |
|  | Isoniazid (H) | 10-15 | Once daily | 300 mg |
|  | Pyrazinamide (Z) | 30-40 | Once daily | 2 g |
|  | Ethambuthol (E) | 15-25 | Once daily | 2.5g |
| Group 2 | Streptomycin (S) (1 g vial) | 15–20 | Once daily | 1 g |
|  | Kanamycin (Km) (1 g vial) | 15–20 | Once daily | 1 g |
|  | Amikacin (Am) (1 g vial) | 15-20 | Once daily | 1 g |
|  | Capreomycin (Cm) (1 g vial) | 15–20 | Once daily | 1 g |
| Group 3 | Ofloxacin (Ofx) (200 mg) | 15–20 | Twice daily | 800 mg |
|  | Levofloxacin (Lfx) (250 mg, 500 mg) | 7.5–10 | Once daily | 750 mg |
|  | Moxifloxacin (Mfx) (400 mg) | 7.5–10 | Once daily | 400 mg |
| Group 4 | Ethionamide (Eto) (250 mg) | 15–20 | Twice daily | 1 g |
|  | Protionamide | 15-20 | Twice daily | 1 g |
|  | Cycloserine (Cs) (250 mg) | 10–20 | Once or twice daily | 1 g |
|  | Terizidone (Trd) (250 mg) | 10–20 | Once or twice daily | 1 g |
|  | PASER (4 g sachets) | 150 | Twice or thrice daily | 12 g |
| Group 5 | High Dose INH | 15-20 | Once daily |  |
|  | Amoxicillin/Clavulanic Acid | 80 | Twice daily |  |
|  | Clarithromycin | 7.5-15 | Twice daily | 1 g |
|  | Linezolid | 10-12 | Every 8 hours | 600mg |

*Source: Adapted from Guidelines for Programmatic Management of Drug-resistant tuberculosis. Emergency update 2008. WHO/HTM/TB/200.402.*

### Adherence support for MDR-TB children

Due to the limited treatment options, we need to ensure adequate adherence when starting MDR TB treatment.

Before starting treatment we need to identify a principal care-giver that lives with the child who will assure an adequate follow up.

In many cases, hospitalization may be required for the first 2-3 months, to administer the injectables, monitor side effects or due to social circumstances. If possible, a home visit pre initiation is recommended to assess the family situation.

As there are no child-friendly formulations, providing MDR TB treatment can be challenging. Intensive pre initiation counseling should be done concerning:

- duration of the treatment,
- possible side effects,
- a plan in case there is a change in the caregiver

The treatment should be assessed regularly:

Each child should be assessed at least at the following intervals:

- 2 weeks after treatment initiation,
- at the end of the intensive phase and every 2 months until treatment completion.

The assessment should include, as a minimum: s

- symptom assessment,
- assessment of treatment adherence,
- enquiry about any adverse events and weight measurement.

Medication dosages should be adjusted to account for any weight gain. Adherence should be assessed by reviewing the treatment card. A follow-up sputum sample for smear microscopy at 2 months after treatment initiation should be obtained for any child who was smear-positive at diagnosis.

Follow-up CXRs are not routinely required in children, particularly as many children will have a slow radiological response to treatment.

A child who is not responding to MDR-TB treatment should be referred for further assessment and management. These children may have drug-resistant TB, an unusual complication, other causes of lung disease or problems with treatment adherence.

### Chemoprophylaxis

Currently there are no standardized recommendations regarding preventive therapy. In cases of pregnant mothers receiving MDR TB treatment, consult a specialist to evaluate different options.

### Side effects

In general, adverse events caused by anti-TB drugs are much less common in children than in adults. The most important adverse event is the development of hepatotoxicity, which can be caused by isoniazid, rifampicin or pyrazinamide.

- Serum liver enzyme levels should not be monitored routinely, as asymptomatic mild elevation of serum liver enzymes (less than five times the normal values) is not an indication to stop treatment. However, the occurrence of liver tenderness, hepatomegaly or jaundice should lead to investigation of serum liver enzyme levels and the immediate stopping of all potentially hepatotoxic drugs.
- Patients should be screened for other causes of hepatitis, and no attempt should be made to reintroduce these drugs until liver functions have normalized. An expert (experienced in managing drug-induced hepatotoxicity) should be involved in the further management of such cases.
- If treatment for TB needs to be continued for severe forms of TB, nonhepatotoxic anti-TB drugs should be introduced (e.g. ethambutol, an aminoglycoside and a fluoroquinolone).
- Isoniazid may cause symptomatic pyridoxine deficiency, particularly in severely malnourished children and HIV-infected children on highly active ART. Supplemental pyridoxine (5–10 mg/day) is recommended in:
  - malnourished children,
  - HIV-infected children
  - breastfeeding infants and
  - pregnant adolescents.

### Managing HIV/DR-TB co-infection in Children

#### Provision of ART

All child TB suspects should be screened for HIV infection, at the beginning of the evaluation and repeatedly if they are considered to be at risk for HIV infection. HIV co-infected children with MDR-TB should be timely initiated on ART as concomitant ART markedly improves TB outcome in children.

ART should be started within 2-8 weeks after starting anti-TB treatment in HIV-infected children with MDR TB in line with WHO recommendation.

In addition to early ART initiation, all co-infected child TB cases should receive cotrimoxazole prophylaxis and pyridoxine supplementation.

Drug interactions are usually not a problem with regimens not containing rifampicin. (d4T with peripheral neuropathy; AZT with anemia; Efaverenz Cs, are interactions sometimes relevant, and poor diagnosis in adults.

#### Reasons to postpone ART

Reasons for postponing ART initiation include:

- Confusion of overlapping drug, adverse effects and
- the risk for immune reconstitution inflammatory syndrome (IRIS), a paradoxical worsening of symptoms and signs with improvement in the body’s immune response. IRIS, if severe, could be managed by corticosteroids;
- to arrange for adequate social support.

To ensure the best outcomes of both the DR TB treatment and ART it is critical that the child has the best family support possible. Health care workers need to assure proper and timely counseling at initiation and throughout the treatment.

### Recommended ART regimens for children on DR TB treatment:

For children who are treatment naïve or on any first-line ART regimen:

Table 8.3: Recommended ART regimens for Treatment naive Children

| under 3 years of age | Age 3 and over |
| --- | --- |
| **AZT–3TC–NVP^a^** | **AZT–3TC–EFV** |
| ^a^If patient is on TB therapy and is ART naïve, NVP should be initiated at twice-daily dosing. Due to enzyme induction by rifampicin, lead-in dosing is not indicated (and will increase the risk of developing NVP resistance). | |

For HIV-exposed children on Kaletra exposed to long prophylaxis with Nevirapine. After treatment with Rifamipicin should be switched back to Kaletra.

### Alternative TB/HIV co-treatment ART regimen options for special situations:

Table 8.4: Alternative TB/HIV cotreatment Child regimen options in special situations

| Special Situation | Alternative Regimen | Comments |
| --- | --- | --- |
| **Child is currently on or qualifies for an LPV/r-based first-line regimen** | May use LPV/r  boosted 1:1 with ritonavir,  if available | Only use if ritonavir availability can be ensured for the duration of TB treatment. |
| **Child is on second-line therapy** | If current regimen contains  LPV/r, continue with  1:1 ritonavir-boosting | Consult a TB or HIV specialist if ritonavir is not available. |
| **Children > 3 years old already on NVP-based regimen before initiating  TB treatment** | AZT–3TC–NVP | Studies in adults show  that NVP-based regimens maintain viral suppression  as well as EFV-based regimens in this situation. |
| **NVP or EFV toxicity** | AZT–3TC–ABC | Immediately switch to  an LPV/r-based regimen when TB therapy is completed. |
| **Severe anemia *(Hb<8 g/dl)*** | Use d4T instead of AZT | Switch to AZT when stable |

*Source: WHO guidelines for antiretroviral therapy for the treatment of HIV infection in infants and Children. 2010 revision; and Swaziland National ART guidelines.*

To reduce pill burden and facilitate adherence children should be switched from EFV to NVP once Rifampicine is not contained in the regimen (no loading dose of NVP is needed in these cases).

### Provision of Cotrimoxazole

Table8.5: Table showing dosage of Cotrimoxazole for CPT provision in Children

| **Dosing for Cotrimoxazole Given Once Daily (TMP/SMX.CTX,Bactrim,Cotrim,Cozole)** | | | | |
| --- | --- | --- | --- | --- |
| **Age** | Weight of child | Suspension 5ml (200mg/40mg) | Pediatric Tablet (100mg/20mg) | Dose |
| **< 6 months** | <5kg | 2.5ml | 1 tablet | 120mg |
| **6m – 5yr** | 5 - 15kg | 5ml | 2 tablets | 240mg |
| **6yr – 14 yr** | 15 - 30kg | 10ml | - | 480mg |
| **>14yr** | >30kg | - | - | 960mg |

*Source: WHO guidelines for antiretroviral therapy for the treatment of HIV infection in infants and Children. 2010 revision; and Swaziland National ART guidelines.*

1. ORGANIZATION OF DR-TB TREATMENT DELIVERY AND COMMUNITY-BASED CARE

## Treatment delivery models

There are two (2) main DR-TB treatment delivery models namely:

- Hospitalization;
- Ambulatory care
  - Peripheral Clinic-based model;
  - Community-based care model;

However, irrespective of the mode of treatment delivery, supply of medicines should be ensured to all patients in need, free of charge to be taken with DOT support, consistent monthly clinical follow up including sputum, smear microscopy and culture assessments, maintenance of infection control to prevent further transmission of the disease.

### Hospitalization: The National TB Hospital

The national tuberculosis hospital is responsible for specialized management of tuberculosis that includes drug resistant tuberculosis. It is equipped with highly skilled professionals and will be responsible for coordinating the implementation of the clinical components of the drug resistant tuberculosis guidelines.

The main indications for hospitalization include the following:

- Initiation of treatment
- Adherence problems
- patient very sick (clinically and physically unfit)
- Severe adverse effects
- Immobility
- Vulnerable patients e.g. disadvantaged-orphan, mentally , socially or physically handicapped

#### Referral of DR-TB patients

Referral of DR-TB patients will essentially follow a two-way system i.e. upward to or downward from the National TB Hospital or Regional MDR-TB Centres depending on the circumstances.

Upward referrals may be necessitated by any of the indications for specialized consultation or hospitalization as listed above, while the downward referrals may be to enable continuation of treatment nearer to patients home.

In the event of referrals to the National TB Hospital, the referring health facility should inform the outpatient department of the TB hospital before transporting the patients for admission.

Similarly, the national TB Hospital should contact and make prior arrangements with facilities that shall be receiving patients referred from the hospital for continuation of treatment and care.

Transport for clients referred to the TB hospital should be arranged by the referring facility in consultation with national TB Hospital.

***All confirmed XDR-TB patients will be admitted and managed in isolation until sputum culture conversion.***

#### Hospitalization

**During the admission period,** the following will be ensured:

- Full clinical assessment including investigation and treatment of other medical conditions; and stabilize patient’s clinical state if very sick.
- initiation on MDR-TB intensive treatment,
- Provide patient with HIV testing and counseling (HTC) or ascertain HIV status
- Initiate patient on ART if confirmed to be HIV positive after 2 weeks of anti-TB treatment.
- arrange for community treatment supporter for administration of DOT,
- Arrange for administration of daily injections at a site nearest to the patient’s home.
- evaluate patient’s household for infection control and contact screening;
- Monitor for development of drug adverse effects and drug interactions e.g between Anti-TB drugs and ARVs etc.
- Treat patients with initial adverse effects or drug reactions until stable
- Discharge Planning; Confirm:
  - that the Household has been assessed for infection control;
  - that a treatment supporter is available and trained;
  - a follow up mechanism decided;
  - administration of injectables defined (in terms of where and by who?);
  - nutritional support plan in place;

#### Discharging hospitalized patients

**The discharge criteria are:**

- Patient’s clinical status improved to the extent that he/she can be managed on ambulatory basis;
- Adequate infection control measures in the home are ensured
- Treatment supporter for DOT during the intensive phase identified and trained;
- Adequate nutritional and social patient support provided;
- Measures to ensure transport to the MDR-TB hospital for regular follow up is taken care of
- Two consecutive negative sputum culture at the end of intensive phase of treatment;

**Upon discharge;**

Complete the following:

- A discharge summary form should be completed;
- A transfer letter be written to the TB unit in triplicate (1 copy remains at TB hospital in patient’s file, 1 copy sent with the patient to take to TB unit, 1 copy sent from the TB hospital to the TB unit).
- Provide to treatment supporter:
- One month supply of drugs and a drug carrier bag;
- Recording forms for treatment adherence and side effects recording;
- Patient appointment card;
- Two (2) sputum containers
- Patient information and education materials;

Arrange:

- Facilitate arrangement for transport to patient’s home in consultation with the relatives.

***Follow up after discharge;***

- During intensive phase—monthly at the TB hospital.
- During continuation phase—every 3^rd^ month at any of the DR-TB Management Centres, and monthly in between the 3 months at TB unit nearest to patient’s home. However, in the event of any serious problem, the patient should be seen at the TB Hospital.

***Items to be brought by the patient on review:***

- Patient to come with Patient card for monitoring of adherence.
  - The Treatment supporter accompanying the patient, should also be requested to provide the Treatment Supporter card for monitoring treatment adherence.
- Patient to bring 2 sputum specimens for smear and culture. The patient will come with an early morning sputum and Spot examination to be done on one of the specimens for smear results.

Termination of treatment should beat the national TB hospital.

### Ambulatory Care

#### Clinic-based treatment:

This refers to a situation where taking medications from a Hospital or health centre is both feasible and about the best or only option available to the patient. This is applicable where the clinic is very near the patient’s home. It involves the patient travelling to the clinic each day to receive DOT by the health care worker including administration of injectable agents.

A separate area with infection control measures is recommended in facilities providing such daily DOT for DR-TB as well as smear positive patients.

Special attention must be taken in clinic-based programmes to avoid exposure of PLHIV to smear-positive patients.

#### Community-based care

Patients may receive community-based care when

- Adequate infection control measures in the home are ensured - minimizing further risk to family and friends
- Adequate treatment support is assured
- Adequate nutritional and social patient support are organized
- Measures to ensure transport to the MDR-TB hospital for regular follow up is in place

With respect to the community-based care of DR-TB patients, the following should be observed:

- The Treatment Supporter will provide support to the patients to ensure that they take their medications as prescribed twice daily and record any unfavourable experiences reported or observed. In the event of a serious condition, the treatment supporter should assist in getting the patient seen at the nearest clinic.
- Patients should be reviewed monthly (at OPD) of the nearest DR-TB Management Centre by a clinician trained in DR-TB management.
- Infection control is also very important for ambulatory care in the intensive phase. Adequate patient education and measures surrounding infection control include:
- cough hygiene education - including not to go to school/work until conversion
- isolation - separate sleeping area must be arranged including away from spouse and children
- adequate ventilation in home,
- use of masks (N95 for the care giver and Surgical masks to the patients);
- Community and family education.

In addition special attention must be given to congregate settings: prisons, army, school, churches, workplace and education on ventilation, education, isolation (not going/appropriate situation).

All working MDR-TB suspects and confirmed MDR-TB patients should be recommended for long term sick leave until culture conversion.

## Ensuring adherence to therapy

DR-TB patients are at increased risk of non-adherence to treatment as they may have already had similar experience with treatment in past. Adherence is an essential element in preventing the generation of pan-resistant strains capable of community-wide spread that leave virtually no possibility of cure for the patient. DR-TB treatment can be successful, with high overall rates of adherence, when adequate support measures are provided.

To ensure adherence to therapy, measures including provision of enablers and incentives for delivery of DOT, disease education, DOT, socioeconomic support, emotional support, prompt management of adverse effects and effective monitoring systems.

### Health education

Patients and their families should receive education about DR-TB, its treatment, potential adverse drug effects and the need for adherence to therapy. This should begin at the start of therapy and continue throughout the course of treatment.

Education should be provided by physicians, nurses, lay and CHWs and other health-care providers using a culturally sensitive IEC materials and methods.

### Directly observed therapy (DOT)

It is recommended that all patients receiving treatment for DR-TB receive directly observed treatment (DOT) either in the community, at Health Centres, clinics, or within the hospital setting. DOT should be provided in a way that does not place undue burdens on patients and their families.

Long travel times and distances, short clinic operation hours and difficulty in accessing services may all reduce the efficacy of DOT.

#### Who should deliver DOT?

DOT can be provided by health-care workers, trained community members etc. It is recommended that, if situations allow, the patient’s DOT worker should not be a family member as family relationships are often complicated for the DR-TB patient. This is because a family observer could be subject to subtle manipulation by the patient, relatives, employers, etc.

#### Maintaining confidentiality:

The DOT worker should be educated on the need to maintain strict confidentiality regarding the patient’s disease. This may entail working out a system whereby the patient can receive medication without the knowledge of others.

### Socioeconomic interventions

Socioeconomic problems, including hunger, homelessness and unemployment, should be addressed to enable patients and their families to adhere to treatment. Enablers e.g. goods or services that make it easier for patients to adhere to treatment, such as the provision of transportation vouchers; or incentives e.g. goods or services that are used to encourage patients to adhere to therapy, such as the provision of clothing should be provided where necessary. Maximal interventions should be given to patients with the most need. These packages may include:

- health care free of charge;
- food parcels for DR-TB patients and their dependents;
- temporary shelter in a housing facility or in a rented home for DR-TB patients;
- school fees for dependent children;
- transportation fees;
- advice and assistance in administrative matters relating to the treatment;
- assistance in defending rights and/or reinforcing the responsibilities of patients;
- providing skills training and livelihood to patients both while on treatment as well as to prepare them with skills that can support them as they reintegrate into the community upon treatment completion.

### Psychosocial and emotional support

Having DR-TB can be associated with severe emotional stress for patients and their families. Considerable stigma is attached to the disease and this may interfere with adherence to therapy. In addition, the long duration of DR-TB therapy coupled with the adverse effects of the drugs may contribute to depression, anxiety and further difficulty with treatment adherence.

The provision of emotional support to patients may increase the likelihood of adherence to therapy. This support may be organized in the form of support groups or interpersonal counseling by trained providers.

Informal support can also be provided by physicians, nurses, DOT workers and family members. It is recommended to use a multidisciplinary “support to adherence” team (social worker, nurse, health educator, companion and doctor).

### Early and effective management of adverse drug effects

Although rarely life-threatening, the adverse effects of second-line drugs can be debilitating for patients. Patients experiencing high rates of adverse effects may be at increased risk of non-adherence.

Therefore, early and effective management of adverse effects should be part of adherence-promotion strategies in the management of DR-TB ***(See section 7.6.3 and Table 7.3).***

### Monitoring and follow-up of the non-adherent patient

Patient should be consistently followed up throughout the duration of their treatment. When a patient fails to attend a DOT appointment, a system should be in place that allows prompt patient follow-up. This may involve a DOT worker visiting the patient’s home the same day to find out why the patient has missed an appointment and to ensure that treatment is resumed promptly and effectively. The situation should be addressed in a sympathetic, friendly and non-judgmental manner. Every effort should be made to listen to reasons for the patient missing a dose(s) and to work with patient and family to ensure continuation of treatment. Transportation problems should be addressed.

### Community-based care and support

Community-based care and support is any action or help provided by, with orfromthe community, including situations in which patients are receiving ambulatorytreatment. This support contributes to, and may even be necessary to,patient recovery. This is crucial in the management of DR-TB. Functions of Community treatment supporters

**— *Clinical management.***This can be in the form of: (i) early detection of potentially serious adverse reactions and prompt referral of such reactions to health workers; (ii) provision of simple, non-medical measures to manage adverse reactions, e.g. oral hydration in mild diarrhoea, or counseling on the avoidance of alcohol while taking drugs that have hepatic effects, etc; and (iii) psychological encouragement. Patients and former patients who endured the same adverse effects while on treatment are usually most effective in this respect.

**— *DOT.***Community-based support in DOT can be highly effective, especially if provided by former patients acting as treatment partners for daily DOT, who are living proof that adherence to daily DOT pays and that there is hope for cure if they persevere with their treatment.

**— *Contact investigation.***New cases can be discovered by community-care supporters through contact tracing if they are adequately trained to do so. Early diagnosis of new cases may improve cure rates and acts as an important infection control measure.

- ***Administration of injectables:*** following recognition by the Ministry of Health; and appropriate training on injection techniques;

***— Facilitating infection control.***Community-based support in infection control includes providing health education to patients on simple infection control practices that can be done in the home, such as observing cough etiquette (covering the mouth and nose when coughing, or sneezing), keeping one’s room well ventilated by opening windows or staying outdoors as much as possible while visiting others.

**— *Recording and reporting.***Data obtained within the family and community can contribute to better comprehensive management. Community DOT supporters should be trained to document processes occurring outside the health centre and closer to the patient’s home.

Recording should include assessment of certain variables during a home visit can better assess risks for the patient and family (such as leaky roofs, insufficient living space or poor sanitary conditions).

Close supervision and validation of recording and reporting by community-care providers should be done by health facility staff, and in a collaborative and supportive manner.

**— *Training/education.*** Community-based training and education can come in the form of peer educators (i.e. former patients) or trained advocates.

Topics can include general information on TB, how DR-TB develops, the treatment of DR-TB and the importance of adherence, TB/HIV interaction and infection control.

Training and education on DR-TB will be most effective with the aid of materials written in lay language.

— ***Advocacy and decreasing stigma.*** Community-based supporters, often in the form of patients, give a voice and face to TB.

The establishment of patient peer education groups (community care club) and perhaps eventually a local organization or association can help reduce stigma and dispel inaccurate information about the disease. The groups can often influence decision-makers for policy change either in the clinics that they attend or in the wider community where they live.

— ***Social support****.* Community care supporters help identify socioeconomic and psychosocial needs and help channel support in a timely and more effective manner. They also help develop community resources that may provide useful support, and encourage patients to contribute to the community by upholding their responsibilities.

### Post-treatment follow-up of DR-TB cases

All DR-TB cases successfully treated should be followed up after discharge to ensure continuity of care and support including psychological support and HIV prevention (if HIV negative).

In the first year of discharge, patients should be followed up on a three monthly-basis, then 6 monthly assessments for at least 5 years period.

The follow up will include assessment of clinical condition, and management of any detected problems. Audiometric assessments should be conducted as part of the follow up clinical examinations.

In the event of recurrence of symptoms and signs suggestive of TB, culture and DST should be requested immediately.

1. DRUG RESISTANT TB AND HIV

## General considerations

HIV co-infection is a significant challenge for the prevention, diagnosis and treatment of DR-TB, especially in the case of MDR-TB and XDR-TB. This is especially in view of the reported high mortality rates associated DR-TB with HIV co-infection, and alarming mortality rates in patients co-infected with XDR-TB and HIV. Recent global drug resistance surveillance suggests an association between HIV and MDR-TB in some parts of the world; although specific factors involved in this association have not been determined HIV is a powerful risk factor for all forms of TB, and DR-TB outbreaks, including XDR-TB outbreaks in HIV-infected patients (e.g. the XDR-TB outbreak associated with high mortality rates in Tugella Ferry, KwaZulu Natal province of South Africa reported in March 2006). This underscores the importance of early diagnosis of DR-TB and HIV, timely initiation of treatment with adequate regimens for both second line treatment and ART, sound patient support and strong infection control measures are all essential components in the management of DR-TB in HIV-infected people.

The concurrent use of ART and DR-TB treatment has been reported to improve outcomes of DR-TB in the HIV-infected.

## Recommended collaborative activities

The collaborative activities to be carried in DR-TB management are based on the standard WHO recommended framework with12 intrevntions aimed at decreasing the joint burden of TB and HIV/AIDS on the same patient. Key among these activities relevant to management of DR-TB includes:

- Perform provider-initiated HIV testing and counseling in all TB suspects.
- Use standard algorithms to diagnose pulmonary and extra-pulmonary TB.
- Use mycobacterial cultures and, where available, newer more rapid methods of diagnosis.
- Perform DST at the start of anti-tuberculosis therapy.
- Initiate DR-TB/HIV patients on ART as early as possible following commencement of anti-TB treatment regardless of CD4 cell-count.
- Consider empirical therapy with second-line anti-tuberculosis drugs.
- Provide CPT for patients with active TB and HIV.
- Arrange treatment follow-up by a specialized team.
- Implement additional nutritional and socioeconomic support.
- Ensure effective infection control.

## Clinical features and diagnosis of DR-TB in HIV-infected patients

The diagnosis of TB (including MDR-TB and XDR-TB) in HIV-infected people is more difficult and may be confused with other pulmonary or systemic infections since the signs and symptoms may not be typical of pulmonary TB. The presentation of TB in HIV-infected individuals is more likely to be extra-pulmonary or sputum smear-negative than in HIV-uninfected TB patients, especially as immune-suppression advances. Therefore misdiagnosis or delays in diagnosis often results, and in turn, leading to higher morbidity and mortality among these patients. The new WHO recommended algorithms emphasize the use of clinical criteria first and, if needed, the use of additional laboratory data (culture) and radiography to diagnose TB with very good positive predictive values and should be used to prevent diagnostic delays.

For patients with advanced HIV disease, mycobacterial culture of other fluids (e.g. blood, pleural fluid, ascitic fluid, cerebrospinal fluid and bone-marrow aspirates) and histopathology (e.g. lymph node biopsies) may be helpful in diagnosis. All HIV patients with TB are screened for drug- resistance with DST using rapid drug-resistance testing to enable prompt diagnosis of MDR-TB.

## Concomitant treatment of DR-TB and HIV

The treatment of DR-TB in HIV positive patients is essentially same as for patients without HIV co-infection. However, the following should be considered:

- ART plays a crucial role, as mortality in MDR-TB/HIV patients without the use of ART is extremely high;
- Adverse effects are more common in patients with HIV. The multiple medicines involved in DR-TB with recognized high toxicity risks, often combined with ART, results in a high incidence of adverse effects. Some toxicity is common to both anti-tuberculosis treatment and ART, which may result in added rates of adverse events.
- Monitoring needs to be more intense for both response to therapy and adverse effects.
- The use of thioacetazone is not recommended for patients with HIV or for routine use in populations with high rates of HIV.
- Immune Reconstitution Syndrome (IRIS) may complicate therapy.

### Initiating ART treatment in patients with DR-TB

The use of ART in HIV-infected patients with TB improves survival for both drug-resistant and susceptible disease.

Simultaneous initiation of both DR-TB treatment and ART carries the likelihood of overlapping toxicities that could compromise the treatment of either condition. However, immune-compromised patients are at high risk for dying without both treatments. Mortality can be as high as or exceed 90% in patients treated for DR-TB without ART.

HIV co-infected DR-TB patients should therefore be initiated on ART as soon as second-line TB drugs are tolerated irrespective of CD4 cell count. This could be as soon as one week in asymptomatic patients. The optimal timing for the introduction of ART in patients receiving MDR-TB treatment should be based on proper evaluation by the Medical Officer.

The preferred regimen is: **AZT+3TC+EFZ,** however AZT should be changed to d4T in case of anaemia; if the patient is diagnosed with severe peripheral neuropathy d4T can be substituted by abacvir or tenofavir. If the toxicity due to efaverenz is unbearable, it can be substituted with Saquinavir or Lopinavir.

In DR-TB Patients who are already on Tenofavir-based regimen and are stable, there is no need to replace TDF with AZT, instead same regimen should be continued while monitoring renal function test closely.

### DR-TB in patients already receiving ART

When a patient is diagnosed with DR-TB while on ART, it is necessary to evaluate whether or not a modification of ART regimen will be required on account of potential drug–drug interactions or overlapping toxicities.

It is also essential to determine whether the presentation of active DR-TB in a patient that is already on ART constitutes failure of the ART regimen.

If ART failure is diagnosed, it is not recommended to begin a new second-line ART regimen at the same time as initiation of a DR-TB regimen. Instead, the current ART regimen should be continued and switched to the second-line ART regimen in about 2–8 weeks after the start of DR-TB treatment.

### Important drug–drug interactions in the treatment of HIV and DR-TB

Drug–drug interactions between second-line anti-tuberculosis agents and antiretroviral therapy can occur, and is capable of complicating treatment. The common interactions between drugs used to treat HIV and TB are summarized below.

**Rifamycin derivatives:** While rifamycin derivatives are not routinely used in DR-TB treatment, they are used in the treatment of rifampicin-sensitive poly- and mono-resistant TB.

**Quinolones and didanosine:** Buffered didanosine contains an aluminium/magnesium-based antacid and, if given jointly with fluoroquinolones, may result in decreased fluoroquinolone absorption; it should be avoided, but if it is necessary it should be given six hours before or two hours after fluoroquinolone administration. The enteric coated (EC) formulation of didanosine can be used concomitantly without this precaution.

**Ethionamide/protionamide:** Based on limited existing information of the metabolisim of the thiamides (ethionamide and protionamide), this drug class may have interactions with antiretroviral drugs. Ethionamide/protionamide is thought to be metabolized by the cytochrome (CY P450) system, although it is not know which of the CYP enzymes are responsible. Whether doses of ethionamide/protionamide and/or certain antiretroviral drugs should be modified during the concomitant treatment of DR-TB and HIV is completely unknown.

**Clarithromycin:** Clarithromycin is a substrate and inhibitor of CYP3A and has multiple drug interactions with protease inhibitors and NNRTIs. If possible, the use of clarithromycin should be avoided in patients co-infected with DR-TB and HIV because of both its weak efficacy against DR-TB and multiple drug interactions.

### Potential drug toxicity in the treatment of HIV and DR-TB

There is limited evidence on the frequency and severity of toxicities and adverse events from ART and second-line anti-tuberculosis therapy. In general, HIV patients have a higher rate of adverse drug reactions to both TB and non-TB medications, and the risk of adverse drug reactions increases with the degree of immunosuppression.

Identifying the source of adverse effects in patients receiving concomitant therapy for DR-TB and HIV may be difficult. Many of the medications used to treat DR-TB and HIV have overlapping, or in some cases additive, toxicities. Often, it may not be possible to link adverse effects to a single drug, as the risk of resistance for ART therapy precludes the typical medical challenge of stopping all medications and starting them one by one.

Adverse effects that are common to both antiretroviral and anti-tuberculosis drugs are listed in *Table 10.1* below*.* It should be noted that relatively very little is known about the rates of adverse effects in the concomitant treatment of DR-TB and HIV. Table 8.1 is meant to alert the clinician to potentially overlapping and additive toxicities, and as of the writing of these guidelines is based on preliminary, non-published data and expert opinion.

When possible, avoid the use of agents with shared adverse effect profiles. Often, however, the benefit of using drugs that have overlying toxicities outweighs the risk. Therefore, if two drugs with overlapping toxicities are determined to be essential in a patient’s regimen, these guidelines recommend increased monitoring of adverse effects rather than disallowing a certain combination.

Table 10.1: Potential overlapping and additive toxicities of ART and anti-tuberculosis therapy(Drugs that are more strongly associated with adverse effects appear in bold)

| **Toxicity** | **Anti-retro-viral agent** | **Anti-tuberculosis agent** | **Comments** |
| --- | --- | --- | --- |
| **Peripheral neuropathy** | **D4T, ddI, ddC** | **Lzd, Cs, H,** Aminoglycosides, Eto/Pto, E | Avoid use of D4T, ddI and ddC in combination with Cs or Lzd because of theoretically increased peripheral neuropathy.  If these agents must be used and peripheral neuropathy develops, replace the ARV agent with a less neurotoxic agent. |
| **Central nervous system (CNS) toxicity** | **EFV** | **Cs,** H, Eto/Pto, Fluoroquinolones | Efavirenz has a high rate of CNS adverse effects (confusion, impaired concentration, depersonalization, abnormal dreams, insomnia and dizziness) in the first 2–3 weeks, which typically resolve on their own. If these effects do not resolve on their own, consider substitution of the agent. At present, there are limited data on the use of EFV with Cs; concurrent use is accepted practice with frequent monitoring for CNS toxicity. Frank psychosis is rare with EFV alone. |
| **Depression** | **EFV** | **Cs,**Fluoroquinolones, H, Eto/Pto | Severe depression can be seen in 2.4% of patients receiving EFV. Consider substituting for EFV if severe depression develops.  The severe socioeconomic circumstances of many patients with chronic disease can also contribute to depression. |
| **Headache** | **AZT, EFV** | **Cs** | Rule out more serious causes of headache such as bacterial meningitis, cryptococcal meningitis, CNS toxoplasmosis, etc. Use of analgesics (ibuprofen, paracetamol) and good hydration may help. Headache secondary to AZT, EFV and Cs is usually self-limited. |
| **Nausea and vomiting** | **RTV, D4T,** NVP, and most others | **Eto/Pto, PAS, H, E, Z** and others | Nausea and vomiting are common adverse effects and can be managed.  Persistent vomiting and abdominal pain may be a result of developing lactic acidosis and/or hepatitis secondary to medications. |
| **Abdominal pain** | **All ART treatment has been associated with abdominal pain** | **Eto/Pto, PAS** | Abdominal pain is a common adverse effect and often benign; however, abdominal pain may be an early symptom of severe adverse effects such as pancreatitis, hepatitis or lactic acidosis. |
| **Pancreatitis** | **D4T, ddI, ddC** | **Lzd** | Avoid use of these agents together. If an agent causes pancreatitis suspend it permanently and do not use any of the pancreatitis producing anti-HIV medications (D4T, ddI, or ddC) in the future.  Also consider gallstones or alcohol as a potential cause of pancreatitis. |
| **Diarrhea** | **All protease inhibitors, ddI (buffered formula)** | **Eto/Pto, PAS,** Fluoroquinolones | Diarrhoea is a common adverse effect. Also consider opportunistic infections as a cause of diarrhoea, or clostridium difficile (a cause of pseudomembranous colitis). |
| **Hepatotoxicity** | **NVP, EFV, all protease inhibitors** (RTV > other protease inhibitors),  **all NRTIs** | **H, R, E, Z,** PAS, Eto/ Pto, Fluoroquinolones | Follow hepatotoxicity treatment recommendations in ***Section 8.7*.**  Also consider TMP/SMX as a cause of hepatotoxicity if the patient is receiving this medication.  Also rule out viral etiologies as cause of hepatitis (Hepatitis A, B, C, and CMV). |
| **Skin rash** | **ABC, NVP, EFV, D4T** and others | **H,R, Z, PAS,**Fluoroquinolones, and others | Do not re-challenge with ABC (can result in life-threatening anaphylaxis). Do not re-challenge with an agent that caused Stevens-Johnson syndrome.  Also consider TMP/SMX as a cause of skin rash if the patient is receiving this medication.  Thioacetazone is contraindicated in HIV because of life-threatening rash. |
| **Lactic acidosis** | **D4T, ddI, AZT, 3TC** | **Lzd** | If an agent causes lactic acidosis, replace it with an agent less likely to cause lactic acidosis. |
| **Renal toxicity** | TDF (rare) | **Aminoglycosides, Cm** | TDF may cause renal injury with the characteristic features of Fanconi syndrome, hypophosphataemia, hypouricaemia, proteinuria, normoglycaemic glycosuria and, in some cases, acute renal failure. There are no data on the concurrent use of TDF with aminoglycosides or Cm. Use TDF with caution in patients receiving aminoglycosides or Cm.  Even without the concurrent use of TDF, HIV-infected patients have an increased risk of renal toxicity secondary to aminoglycosides and Cm. Frequent creatinine and electrolyte monitoring every 1 to 3 weeks is recommended.  Many ARV and anti-tuberculosis medications need to be dose adjusted for renal insufficiency. |
| **Nephrolithiasis** | **IDV** | None | No overlapping toxicities regarding nephrolithiasis have been documented between ART and anti-tuberculosis medications. Adequate hydration prevents nephrolithiasis in patients taking IDV. If nephrolithiasis develops while on IDV, substitute with another protease inhibitor if possible. |
| **Electrolyte disturbances** | TDF (rare) | **Cm, Aminoglycosides** | Diarrhoea and/or vomiting can contribute to electrolyte disturbances.  Even without the concurrent use of TDF, HIV-infected patients have an increased risk of both renal toxicity and electrolyte disturbances secondary to aminoglycosides and Cm. |
| **Bone marrow suppression** | AZT | **Lzd**, R, Rfb, H | Monitor blood counts regularly. Replace AZT if bone marrow suppression develops. Consider suspension of Lzd.  Also consider TMP/SMX as a cause if the patient is receiving this medication.  Consider adding folinic acid supplements, especially if receiving TMP/SMX. |
| **Optic neuritis** | ddI | **E**, Eto/Pto (rare) | Suspend agent responsible for optic neuritis permanently and replace with an agent that does not cause optic neuritis. |
| **Hyperlipidemia** | **Protease inhibitors, EFV** | None | No overlapping toxicities regarding hyperlipidemia have been documented between ART and anti-tuberculosis medications. |
| **Lipodystrophy** | **NRTIs** (especially D4T and ddI | None | No overlapping toxicities regarding lipodystrophy have been documented between ART and anti-tuberculosis medications. |
| **Dysglycemia (disturbed blood sugar regulation)** | **Protease inhibitors** | **Gfx**, Eto/Pto | Protease inhibitors tend to cause insulin resistance and hyperglycaemia. Eto/Pto tends to make insulin control in diabetics more difficult, and can result in hypoglycaemia and poor glucose regulation.  Gatifloxacin is no longer recommended by the GLC for use in treatment of TB because of this side-effect. |
| **Hypothyroidism** | D4T | **Eto/Pto, PAS** | There is potential for overlying toxicity, but evidence is mixed. Several studies show subclinical hypothyroidism associated with HAART, particularly stavudine. PAS and Eto/Pto, especially in combination, can commonly cause hypothyroidism. |

*Source: Adapted for Guidelines for programmatic management of drug-resistant tuberculosis. Emergency update 2008. WHO/HTM/TB/2008.402*

### Monitoring of DR-TB and HIV therapy in co-infected patients

HIV treatment must be taken daily without exception to prevent the evolution of drug resistance. Since DOT is an important component of DR-TB therapy, programmes would be advised to explore the provision of TB medications and ARVs through concomitant DOT or other methods of adherence support. This is particularly important in the setting of second-line anti-tuberculosis therapy, since it can result in a large pill burden and numerous adverse effects that make taking ARVs more difficult.

The complexity of antiretroviral regimens and second-line anti-tuberculosis treatment, each with its own toxicity profiles and some of which may be potentiated by concomitant therapy, demands rigorous clinical monitoring. **Chapter 10, Table 10.1** describes the monitoring requirements while on DR-TB therapy and indicates where any extra monitoring is required for patients co-infected with HIV and/or on ART.

If the patient shows signs of anti-tuberculosis treatment failure, the same evaluation described in this guideline should be undertaken. In addition, the ART regimen should be evaluated for possible treatment failure, as described in national ART guidelines.

Patients with HIV-associated DR-TB may require special socioeconomic, nutritional and psychosocial support in order to successfully complete treatment.

### Immune reconstitution inflammatory syndrome (IRIS)

This syndrome can present as a paradoxical worsening of the patient’s clinical status, often due a previously subclinical and unrecognized opportunistic infection. These reactions may present as fever, enlarging lymph nodes, worsening pulmonary infiltrates, respiratory distress or exacerbation of inflammatory changes at other sites. It generally presents within three months of the initiation of ART and is more common with a low CD4 cell count (<50 cells/mm^3^). It is relatively common in mild to moderate forms in patients with TB started on ART (seen in up to one third of patients in some studies; however, it is relatively rare in its severe forms. It is important to note that IRIS is a diagnosis of exclusion.

Patients with advanced AIDS may show clinical deterioration for a number of other reasons. New opportunistic infections or previously subclinical infections may be unmasked following immune reconstitution and cause clinical worsening. IRIS can also be confused with TB treatment failure, and co-infected patients may be demonstrating progression of TB disease due to drug resistance.

The management of IRIS is complex and depends on the clinical status of the patient and the site and extent of involvement. Various treatment modalities have been employed, including non-steroidal anti-inflammatory drugs in mild disease and corticosteroids in moderate-severe disease. Most patients can be treated without interruption of ART.

1. MANAGEMENT OF DR-TB CONTACTS

## General considerations

Available data indicate that close contacts of MDR-TB patients who develop active TB most commonly have drug-resistant disease. Therefore, the lack of investigation of contacts of MDR/XDR-TB cases, and failure to ask patients with active TB disease about any history of exposure to MDR-TB cases amounts to a missed opportunity to halt transmission of DR-TB in the communities. While all contacts of TB require investigation, DR-TB requires the most vigilance.

Due to the severe risk of morbidity and mortality of XDR-TB, contact tracing of cases of XDR-TB should be given the highest level of alertness and priority. Contact investigation of XDR-TB should be considered as an emergency activity.

By definition, close contacts of MDR-TB patients are people living in the same household, or spending eight (8)hours a day together with the patient in the same indoor living space since the patient became symptomatic with cough (or within the last 3 months if this is not known).

These include:

- People spending nights in the same room as patient including spouses, children, carers etc.
- People spending time in common living areas ( kitchen etc)
- Casual contacts would include most work, school and social contacts. These may not require routine screening unless they are thought to be susceptible (< 5 years old, immuno-compromised) or unless the index case (patient) seems to have been highly infectious (> 10 % of close contacts are found to be infected).

## Contact investigation procedure

Initial contact tracing should be done at the time of diagnosis. Doctors, nurses or adherence officers, treatment supporters all have roles in contact investigation. HCWs should be trained in contact investigation procedures according to national guidelines.

Initially the patient should be informed of who may be at risk by explaining the risk factors (as above). He/She should then be given to time to compile a list of contacts. A contact investigation form should be completed at the time of registration for each patient detailing the contacts they have, any known risk factors the contacts may have, any known symptoms they may be having.

All the close contacts indicated on the contact investigation form should be systematically traced through an initial home visit usually by a trained adherence officer with knowledge and experience of contact investigation and infection control.

When the patient is diagnosed at a facility away from his/her home, the list of his/her contacts should be communicated to a contact tracer nearest to the patient’s home. Where possible, this visit should be combined with home assessment for infection control in collaboration with the regional TB coordinators.

The remaining sections of the contact investigation form will be completed upon symptomatic question screen, and identifying further close contacts at the home.

Note that it is not uncommon that patients may not consider a spouse, or a child who is staying with them to be at risk.

This visit should fulfill the following:

- Identifying the contacts
- Symptomatic screening of the contacts
- health education of family members including how TB is spread, infection control, effective isolation of the patient when on ambulatory care, nutrition, family planning, adherence and TB/ HIV issues.
- Education of family and contacts on symptoms of TB and encouragement to present to hospital as soon as they develop symptoms; and to explain that they are a close contact for the next 2 years;
- Record the contact tracing on the patient’s health card;
- encouragement to have HIV tests if they do not currently know their status

Any symptomatic patient discovered should be referred to the nearest TB clinic for sputum microscopy, Gene Xpert test, culture and DST and referred to a clinician for a more thorough assessment. Alternatively, the sputum could be collected and transporter to the laboratory for examination.

If persons other than household members of the case are to be traced and screened, this should be done appropriately to ensure protection of the individual’s confidentiality. If the number of people are few, where feasible, initial contact should be attempted by telephone and appointment made to assess them in a clinical setting. If this cannot be achieved then a home, school, or workplace visit may be necessary.

In all cases every attempt should be made to maintain patient confidentiality but informing line managers, head teacher etc may be necessary to perform inspection of the facility and interview staff can be discreetly.

The patient should be kept fully informed and should be assured of that every attempt will be made to maintain confidentiality and any information imparted will be kept minimal. It often helps to discuss with the patient the extent to which they are happy for information relating to their condition to be shared with others.

## Management of symptomatic adult contacts of a patient with MDR-TB

All close contacts of MDR-TB cases should be identified through contact tracing and evaluated for active TB by a health-care provider. If active TB disease is suspected, DST using the Gene Xpert or Line Probe Assay should be performed immediately. If Xpert or LPA results show positive for rifampicin, or rifampicin and Isoniazid, the patient should be started on an empiric treatment based on the resistance pattern of the source case, or the standardized MDR-TB regimen. Conventional DST should be requested immediately, and treatment can be modified based on the results.

If any DST cannot be obtained, or while DST results are awaited, an empirical regimen based either on the resistance pattern of the index case or on the most common resistance pattern in the community may be started. Delay in the diagnosis of MDR-TB and start of appropriate treatment can lead to increased morbidity and mortality as well as unchecked amplification and transmission of drug-resistant strains of TB.

When investigation of a symptomatic adult contact yields no evidence of TB, a trial of a broad-spectrum antibiotic, particularly one that is not active against TB, such as trimethoprim/sulfamethoxazole, can be used.

If the patient continues to have symptoms, the patient should be referred for thorough clinical assessment by a medical officer (may include depending on feasibility directed bronchoscopy, CT scan etc). Where these diagnostic tools are not available or the results are not conclusive, a diagnosis should be based on the clinical information at hand. If the initial investigation is not suggestive of active TB but the contact remains symptomatic, repeat physical examinations, smears and cultures should be performed monthly with repeat chest X-ray as needed.

## Management of symptomatic Paediatric contacts of patients with MDR-TB

MDR-TB should be suspected in children with active TB in the following situations:

- A child who is a close contact of an MDR-TB patient.
- A child who is a contact of a TB patient who died while on treatment when there are reasons to suspect that the disease was MDR-TB (i.e. the deceased patient had been a contact of another MDR-TB case, had poor adherence to treatment or had received more than two courses of anti-tuberculosis treatment).
- Children with TB who are not responding to first-line drugs given with direct observation.

The diagnosis of TB is more difficult in children than in adults. Symptoms of TB in young children can be nonspecific, e.g. chronic cough or wheeze, failure to thrive and recurrent fevers. Bacteriological confirmation may be difficult to obtain because of the inability of children to generate a sputum sample, as well as the paucibacillary nature of paediatric TB and the increased likelihood of extrapulmonary TB in children. While every effort should be made to establish a bacteriological diagnosis (and obtain DST) in a child with suspected MDR-TB, in practice paediatric cases are often not confirmed bacteriologically.

Symptomatic paediatric household contacts should receive:

- An evaluation by a physician, including history and physical examination.
- Tuberculin skin testing with purified protein derivative (PPD).
- A chest X-ray examination (computerized tomography if available is helpful especially in documenting hilaradenopathy).
- Sputum smear, culture and DST: every effort should be made to establish a bacteriological diagnosis (and obtain DST) in a child with suspected DR-TB. Bacteriological confirmation may include more aggressive measures such as induced sputum, gastric aspirate, lymph node aspirate or other relevant sample, plus culture and DST.
- For better preservation of specimen, NaHCO_3_ should be added;
- HIV counseling and testing should be routinely offered for all cases.

When the tuberculin (PPD) skin test result is >5 mm but the chest radiograph and gastric aspirate or sputum smear are negative, the symptomatic child can be treated with a broad-spectrum antibiotic that is not active against TB, such as trimethoprim/sulfamethoxazole. The child should be followed closely, with evaluations including smear test and culture on samples from induced sputum or gastric aspirates, or sputum samples whenever possible, as well as chest X-rays. The optimal frequency of these evaluations has not yet been determined.

Children with MDR-TB who are incorrectly entered in SCC may suffer significant and protracted morbidity as a result of ongoing active disease, with the possibility of lifelong disability or even death. Because children with TB may never become sputum smear-positive, it is reasonable to initiate empirical MDR-TB therapy based on the DST pattern of the contact.

## Chemoprophylaxis of contacts of MDR-TB index cases

Close contacts of DR-TB patients should receive careful clinical follow-up for a period of at least two years. If active disease develops, appropriate treatment should be promptly initiated as a new case of MDR-TB as outlined in this guideline. Note that the universal use of second-line drugs for chemoprophylaxis in MDR-TB contacts is not recommended and therefore should not be practiced.

1. DR-TB AND INFECTION CONTROL

## When is TB infectious?

Persons with Tuberculosis of the lungs or larynx are the most infectious and constitute potential sources of spread to others. In general any TB suspect should be considered infectious until proven otherwise through a laboratory confirmation by negative sputum smear result.

## The priorities of infection control

DR-TB is transmitted in the same manner as drug-susceptible TB (aerosol). Well-documented outbreaks of highly drug-resistant strains of TB constitute convincing evidence that DR-TB is transmissible, especially among highly vulnerable populations and in institutional settings. Moreover, because DR-TB patients may respond to treatment slowly and remain sputum smear-positive longer than other TB patients, they may infect more contacts, hence the need to ensure adequate and effective infection control measures.

Health care settings pose require special infection control considerations for the following:

- Potentially contagious undiagnosed and untreated TB cases are often seen and managed in health care settings;
- Persons with HIV-associated immune-suppression may become infected or re-infected with TB if they are exposed to someone with infectious TB disease.
- Health care workers and other staff are also at particularly high risk of infection with TB because of frequent exposure to patients with infectious TB disease. Health care workers and staff may themselves be immune-suppressed due to HIV infection and be at higher risk of developing TB disease once infected.
- There are already documented evidence of multiple TB outbreaks affecting HIV-infected patients and health care workers due to health care facility exposures.

## Infection Control measures

Infection Control measures are designed based on the high likelihood of persons with infectious TB being attended to in health care settings, and the possibility of them spreading *M. tuberculosis* to other persons, especially immune-compromised patients or staff. Infection control interventions are meant to significantly reduce this risk of such transmission in health care and other settings.

In general, there are two main approaches by which chances of transmitting M. tuberculosis can be reduced in health care settings:

- Administrative (work place) control measures
- Environmental control measures.

### Administrative controls

Administrative control measures serve as the first line of defense for preventing the spread of TB in Health care settings, and usually have the greatest impact on preventing TB transmission within settings. These measures prevent droplet nuclei containing M. tuberculosis from being generated in the facility, and thus reduce exposure of patients and staff to TB.

Administrative controls are therefore considered priority irrespective of availability of resources.

Administrative controls have 5 main elements:

1. Infection control plan;
2. Administrative support for procedures in the plan, including quality assurance;
3. Training of staff;
4. Education of patients and increasing community awareness; and
5. Coordination and communication with the TB program.

#### Infection Control plan

Each facility should have a written TB infection control plan that outlines a protocol for the prompt recognition, separation, provision of services, investigation for TB and referral of patients with suspected or confirmed TB disease.

The plan should designate a staff member as an ***Infection Control Officer*** who is responsible for overseeing the implementation of the infection control procedures in the health facility.

The plan should address the following policy and practice areas:

1. Screening all patients as soon as possible after arrival at the facility to identify persons with symptoms of TB disease or persons who are being investigated or treated for TB disease.
2. Instructing the persons identified through screening on respiratory hygiene/cough etiquette. This includes instructing them to cover their nose and mouth when coughing or sneezing, and when possible and if acceptable, providing face masks or tissues to assist them in covering their mouths.
3. Surgical masks could be provided to persons who have a positive symptom screen to wear until they leave the facility. Alternatively, tissues can be provided to these persons, with instructions to cover their mouths and noses when coughing or sneezing. These are less costly and also less likely to identify people as TB suspects with attendant risk of stigma. Tissues and face masks to be disposed properly in waste receptacles.
4. Clients and especially staff should be encouraged to wash their hands after contact with respiratory secretions. Placing TB suspects and cases in a separate well-ventilated waiting area such as a sheltered open-air space if possible;.
5. Speeding up management of persons with cough so that they spend as little time as possible at the facility.
6. Ensuring rapid diagnostic investigation of TB suspects, including referring TB suspects to TB diagnostic services if not available on site; and ensuring that persons reporting TB treatment are adhering with their treatment.
7. Appropriate use and maintenance of the environmental control measure facilities; Training and educating all staff on TB and the TB infection control plan (training should include special risks for TB for HIV-infected persons, and need for diagnostic investigation for those with signs or symptoms of TB).
8. Provision of voluntary, confidential HIV counseling and testing for staff with adequate access to treatment.
9. Monitoring the TB infection control plan’s implementation and correcting any inappropriate practices or failure to adhere to institutional policies.

### Environmental controls

Because administrative controls may not eliminate all possible exposure, environmental control measures must be added to reduce the concentration of droplet nuclei in the air. Environmental controls are therefore considered as the second line of defense for preventing the spread of TB in health care settings.

It is important to recognize that if administrative controls are inadequate, environmental controls **will not eliminate the risk.**

Environmental controls include:

- - Ventilation (natural and mechanical),
  - Filtration, and
  - Ultraviolet germicidal irradiation.

Ventilation is the movement of air in a building and replacement of air in a building with air from outside. Natural ventilation relies on open doors and windows to bring in air from the outside. When fresh air enters a room it dilutes the concentration of particles in room air, such as droplet nuclei containing M. tuberculosis. Controlled natural ventilation can reduce the risk of spreading M. tuberculosis.

Controlled ventilation is when:

- checks are in place to make sure that doors and windows are maintained in an open position that enhances ventilation through ensuring directional flow of air;
- Fans are used to assist in distributing or extracting the air the air; and
- Mechanical systems are used to control air-exchanges using the High Efficiency Particulate Air (HEPA) filtration system.

The following should be observed with respect to ventilation systems:

- Natural ventilation can be used as much as possible provided there is a good directional flow of air that ensures good ventilation (open windows);
- Efforts should be made to ensure that extraction and mechanical ventilation systems are maintained and functions correctly;
- Laboratories that process specimens that may be DR-TB require particularly strict environmental controls (safety cabinets).

Ventilation can be supplemented with upper-room Ultra-violet Germicidal Irradiation (UVGI), which has been known to be extremely effective in inactivating infectious particles in the air above people’s heads, while not exposing them to skin or eye irritation. It is therefore meant to decontaminate air while the infectious source and other occupants are present.

The UVGI should be properly maintained to avoid adverse reactions, such as acute and chronic skin and eye changes from overexposure if the UVGI is not installed and maintained properly.

### Personal respiratory protection (special masks)

Masks that prevent TB transmission are known as ***“particulate respirators”*** or simply “respirators”. They are designed to protect the wearer from tiny (1–5 μm) airborne infectious droplets.

Respirator masks can protect health care workers from inhaling *M. tuberculosis* only if standard work practice and environmental controls are in place. However, personal respiratory protection (i.e., the selection, training, and use of respirators) should be restricted to specific high risk areas in hospitals and referral centers, such as rooms where spirometry or bronchoscopy are performed or specialized treatment centers for persons with MDRTB. Depending on the indication, a certified N95 (or greater e.g. N100) or EU-certified FFP2 (or greater) respirator should be used.

To optimize the benefit of the respirator masks, they must fit tightly on the face, especially around the bridge of the nose. Ideally, respirators should be “fit tested” for individual wearers. In addition to choosing the proper model for each worker, this process serves to educate workers.

It should be noted that respirators are different from surgical masks, or other masks made of cloth or paper. Use of a surgical mask does not protect health care workers, other staff, patients, or visitors against TB. Therefore, surgical masks are NOT recommended as a protective device against DR-TB infection for health care workers and other staff or visitors in HIV care settings.

Furthermore, because they are visible and relatively expensive, it is sometimes assumed that personal respirators alone will prevent TB transmission. However, they cannot be worn continuously and are likely not to be in use when unsuspected TB cases, or unsuspected DR-TB, are encountered. For these reasons, administrative controls that aim to detect and separate cases, and engineering controls that can reduce the risk even for unsuspected cases, are more important.

## High Risk areas

Special precaution will be observed in the following high risk areas: laboratory, medical wards, X-ray department, out patients departments, TB units, ART units, pharmacy and other congregated areas with the health care facilities.

Baseline health status of the health care workers will be required at the time of enrolment and at regular intervals, pregnant and HIV positive staff to be relocated to less risk sections within the department, ensure use of personal protective equipment especially respirators and gowns, close monitoring of mechanical ventilation for functionality.

On medical wards, patient cohorting should be done based on level of infectivity, type of resistance and patents who have converted separated from those who have not converted. Cough hygiene should strictly be enforced.

Protective clothing will be required while caring for isolated patients. Patients who are DR-TB accessing x-ray services should be encouraged to wear masks while accessing the service.

In OPD, especially at the DR-TB Management Centres , triaging on should be done based on whether the patients have MDR-TB, Mono/poly resistance or XDR-TB. Likewise those who are of known HIV status separated and attended to as priority to prevent cross infection with a more resistant strain.

## Measures to reduce infection transmission in community settings where there is congregation

Patient education should emphasize minimizing opportunities for transmission of DR-TB to the community in situations of congregation by observing cough hygiene and where possible avoiding places such as church, public transport, markets, and funerals before culture conversion.

Screening for TB is encouraged for persons staying in congregate institutions for example school children joining boarding schools and on prisoners on entry and at regular 6 monthly intervals.

1. DR-TB MONITORING AND EVALUATION SYSTEM

## Aims of the information system and performance indicators

The aims of the information system are two-fold:

- To allow NTCP at national and local level to monitor overall programme performance (such as patients started on treatment and treatment results), to follow trends in the number of cases notified, to plan drug supply, and to provide the basis for programme and policy developments.
- To aid clinical providers in management of individual patients.

Four minimum indicators have been identified to assess the pattern of enrolment of TB patients on second-line drug treatment, including that among children and females. These are grouped under:

- Detection
- Enrolment
- Interim results
- Final results

An additional stratification for HIV-positive MDR-TB patients assesses the proportion of them on antiretroviral treatment (ART). Confirmed XDR-TB patients should be put on adequate medication.

Table 13.1: Summary of indicators for monitoring the DR-TB programme

| Component | **Indicator** | **Indicator definition** | **Period of assessment** | **Data source** |
| --- | --- | --- | --- | --- |
| DETECTION  This indicator is meant to assessearly detection of resistance to ensure a correct drug regimen from the start and lower risks of further amplification of drug resistance.it also indicates how the targeting and timeliness of DST, as well as the yield of MDR cases, vary by the risk category of the patient targeted.  The indicators should be calculated for all cases tested and as many risk categories in the national guideline. | Confirmed MDR-TB cases detected among TB patients tested for isoniazid and rifampicin DST | Number of confirmed MDR-TB cases by each risk category according to national guideline during the period of assessment) divided by the Number of TB cases in each respective risk category with DST result for both isoniazid and rifampicin during the period of assessment. | Based on Six Calendar month cohort.  (Indicators are measured three (3) months after the end of the six-month period) | DR-TB register, DR-TB Treatment card; and DR-TB Laboratory register for culture and DST. |
| ENROLLMENT  The set of indicators are meant to assess the extent to whichidentified MDR-TB cases have access to care (albeit that patients started on treatment may have been detected prior to the period of assessment). | Confirmed MDR-TB cases enrolled on MDR-TB treatment regimen. | Defined as Number of confirmed MDR-TB cases registered and started on a prescribed MDR-TB treatment regimen during the period of assessment divided by the Number of confirmed MDR-TB cases detected during the period of assessment.  This indicator is computed for (i) all cases, (ii) cases with HIV on ART, and (iii) cases with HIV but not known to be on ART. | Based on Six Calendar month cohort.  (Indicators are measured three (3) months after the end of the six-month period) | MDR-TB Register |
| INTERIM RESULTS  These indicators provide an indication of how patients are faring well before final outcomes can be assessed typically two to three years after the start of enrolment.  Culture conversion (for confirmed pulmonary cases), loss to follow up and death by six months will be used as a proxy of final outcomes.  The number of patients started on second-line drugs for MDR turned out not to be MDR should also be assessed, and likewise for XDR. The aim is to evaluate the effectiveness of the treatment algorithm in reserving treatment for patients who really need it and avoiding a potentially toxic regimen in patients who do not | MDR-TB cases on MDR-TB treatment regimen with negative culture by six months: | Number of confirmed pulmonary MDR-TB cases registered and started on a prescribed MDR-TB treatment with negative results for culture during month 6 of their treatment divided by the Number of confirmed MDR-TB cases registered and started on treatment for MDR-TB during the period of assessment. | Based on Six Calendar month cohort.  (Indicators are measured three (3) months after the end of the six-month period) | MDR-TB treatment register and the laboratory register for culture and DST |
| The first indicator would only apply to pulmonary cases. To simplify, the denominator for all indicators is all cases started on treatment. The three indicators should include XDR-TB cases started on prescribed treatment with second-line drugs. | MDR-TB cases on MDR-TB treatment regimen who died by six months: | Number of confirmed MDR-TB cases registered and started on a prescribed MDR-TB treatment that died of any cause by the end of month 6 of their treatment divided by the Number of confirmed MDR-TB cases registered and started on treatment for MDR-TB during the period of assessment. |  | MDR-TB treatment register |
| The indicator should be measured nine months after the end of the quarter of assessment. This gives sufficient time for culture results at month 6 to be issued and retrieved. | MDR-TB cases on MDR-TB treatment regimen who are lost to follow up by six months: | Number of confirmed MDR-TB cases registered and started on a prescribed MDR-TB treatment who are lost to follow up by the end of month 6 of their treatment divided by the Number of confirmed MDR-TB cases registered and started on treatment for MDR-TB during the period of assessment. |  | MDR-TB treatment register |
|  | Patients on MDR-TB treatment regimen found not to have MDR | Number of patients started on a prescribed MDR-TB treatment regimen during the period of assessment and later found not to be MDR. |  | MDR-TB treatment register |
|  | Patients on XDR-TB treatment regimen found not to have XDR | Number of patients started on a prescribed XDR-TB treatment regimen during the period of assessment and later found not to be XDR. |  | MDR-TB treatment register |
| FINAL OUTCOMES  All patients should be assigned the first outcome they experience for the treatment being evaluated.  Success (cure and completion) and death should be measured separately for HIV-positive individuals. | Cured | A DR-TB patients who completed the course of DR-TB treatment according to the national guideline without evidence of failure **AND** three or more consecutive cultures taken at least 30 days apart are negative after the intensive phase. |  |  |
|  | Completed treatment | A DR-TB patients who completed the course of DR-TB treatment according to the national guideline without evidence of failure **BUT** no record that three or more consecutive cultures taken at least 30 days apart are negative after the intensive phase | Based on 12 Calendar months cohorts i.e January to December.  Assessed 24 months after the end of the of the cohorting year. | MDR-TB Register |
|  | Died | A DR-TB patient who dies for any reason during the course of MDR-TB treatment. |  |  |
|  | Failed | A DR-TB patient whose treatment is terminated or needs a permanent regimen change of greater than or equal to 2 anti-TB drugs because of any of the following:   - lack of conversion in the continuation phase, *or* - bacteriological reversion* in the continuation phase after conversion** to negative, *or* - evidence of additional acquired resistance, *or*   adverse drug reactions |  |  |
|  | Lost to follow up | A DR-TB patient whose treatment was interrupted for two or more consecutive months for any reason. |  |  |
|  | Not evaluated | Patient with RR or MDR-TB for whom no treatment outcome is assigned. |  |  |

## Scope of the information system

The information system for treatment of DR-TB is based upon, and is an extension of, the basic DOTS information system. The use of the standard recording and reporting forms should be consistent across all health facilities managing DR-TB patients.

The MDR-TB information system will also include electronic medical records for clinical case management as well as a GIS-based mapping system for community-based care and patient follow up.

## Main forms/registers and flow of information

The forms and registers include the following:

1. DR-TB suspect register
2. Request for sputum examination
3. DR-TB Laboratory register
4. MDR-TB Register
5. MDR-TB Treatment Card
6. Patient identity card
7. Treatment supporter card
8. Adherence monitoring card
9. Drug order form

Reports include:

- MDR-TB case finding report
- Quarterly report on MDR-TB detection and enrollment on DR-TB treatment;
- Six-month interim outcome assessment of confirmed MDR-TB cases;
- Annual report of treatment result of confirmed MDR-TB patients starting DR-TB treatment;
- Final outcome results form
- Monthly Drug management report

### MDR-TB Treatment Card

The DR-TB Treatment Card contains the following sections:

*Page 1*

- Basic demographic and clinical information. Records name, address, sex, age, weight and site of disease.
- DR-TB registration number. This is a new unique identification number assigned when the patient is entered in the DR-TB Register.
- Date of registration. Provides registration date in the DR-TB Register.
- Previous BMU TB registration number and date of registration.
- Registration group according to result of previous anti-tuberculosis treatment. See Chapter 2, section 2.4 for definitions.
- Previous TB treatment episodes. Lists and describes any previous anti-tuberculosis treatment and outcomes. Start with the earliest treatment and label it number 1. Use the drug abbreviations given on the front of the treatment card. Also note here the outcome of any previous treatment.
- Previous use of second-line anti-tuberculosis drugs. Documents use of any of the second-line drugs listed at the front of the chart for anti-tuberculosis treatment for more than one month.
- Meetings review panel (Case management committee). These guidelines promote periodic meetings with the group of caregivers involved with DR-TB patients. This section provides a space to record major decisions by the panel.

*Page 2*

- HIV testing information. This section is filled in for all patients. If tested for HIV, include date of testing and results. If HIV-infected, indicate whether patient is on ART and/or CPT.
- HIV flow sheet. This section is only filled in for HIV-infected patients.
- Monitoring of weight. Weight should be recorded at least monthly.
- Monitoring of laboratory data including creatinine, potassium, liver function tests, and thyroid tests. Recommendations regarding the interval for monitoring these indicators can be found in Chapter 8.

*Page 3*

- Medical diagnoses other than TB. All other important medical diagnoses are recorded here, including diabetes, hypertension, cardiomyopathy, HIV, opportunistic infections, etc.
- Monitoring and recording adverse effects. Record date, adverse effects and suspected drug(s).

*Page 4*

- DST results. Record the date of sputum collection and results of all DST performed.
- Monitoring of chest X-ray.
- Monitoring of smear and culture. Record date of sputum collection, sample number in the laboratory register and result of smear and culture. “Prior” refers to the sample used to indicate DR-TB registration; include the date and result of that sample. Month “0” is the b time of specimen collection at the start of the DR-TB regimen. Requirements for monitoring by smear and culture are described in *Chapter 6.*

*Pages 5 and 6*

- Regimen. Record the initial MDR-TB regimen and later changes. Use one line for each date on which a drug(s) is changed. If drug dosage is progressively increased (e.g. starting 250 mg of ethionamide daily and increasing by 250 mg over 2–3 days until the full dose is reached), record this in the patient’s medical record(not on the treatment card).
- Record of daily observed administration of drugs. This is constructed with one line per month to facilitate assessment of adherence. Mark one box for each day the entire treatment is administered. Additionally, if dosing is twice daily, one slash mark could be made for the A.M. dose and a second, intersecting mark could be made for the P.M. dose; if both are received, the box would contain an “x”. An alternative is a more detailed system containing one box for each drug prescribed daily, since there may be some inconsistency in administration among drugs.
- Outcome of treatment. *Chapter 3, Section 3.5* provides definitions. Record the outcome of treatment when the final bacteriology results become available.

### MDR-TB Register

Two TB registers should be maintained as follows: i) a BMU Tuberculosis Register and ii) a DR-TB Register. The DR-TB will contain the record of all patients who start DR-TB treatment (see *Chapter 3 Section 3.1* for a general definition of DR-TB patients). This register allows quick assessment of the implementation of DR-TB, facilitating quarterly reporting and analysis of treatment start and outcomes. However, only the TB hospital will register all MDR-TB patients in the country

The following information is recorded in the DR-TB Register:

DR-TB registration number.

- **Date of DR-TB registration.**
- **Name, sex, date of birth, address (from treatment card, p. 1).**
- **BMU TB registration number.** All patients should have been entered in a BMU Tuberculosis Register. A patient who for any reason has never been registered in the BMU Tuberculosis Register should be registered there and the number transferred to the DR-TB Register.
- **Site of disease (from treatment card, p. 1).** Pulmonary, extrapulmonary or both. Patients with both pulmonary and extrapulmonary TB should be classified as a case of pulmonary TB.
- **Registration group (from treatment card, p. 1).** Described in ***Chapter 3, section 3.4.***
- **Second-line drugs received for more than one month prior to registration (from treatment card, p. 1).**
- **DST (from treatment card, p. 4).** Date sample taken, date of DST result and the results. Enter the DST that resulted in the patient being registered as a DR-TB patient. Follow-up DSTs are not recorded in the register. If the patient has more than one DST, results are recorded on the treatment card. If DST is performed in a staged fashion (e.g. of rifampicin and isoniazid first, followed by other first-line drugs, and then of second-line drugs) all results from the same sample should be recorded in the register.
- **DR-TB regimen (from treatment card, p. 5).** Record the initial DR-TB regimen using the drug abbreviations. Include milligram doses and number of tablets.
- **Date of start of DR-TB treatment (from treatment card, p.5).**
- **Smear and culture monitoring results (from treatment card, p.4)**. Record all smear and culture results, even if done more often than the recommended frequency.
- **Final outcome (from treatment card, p.6). See Chapter 3, section 3.5** for definitions.
- **HIV status (from treatment card, p.2)** Testing results, CPT and ART treatment information.
- **Comments.**

### Request for sputum examination

The upper portion is for requesting smear microscopy, the middle portion for culture and the lower portion for DST; the last section is used for reporting the results. When DST is requested, the registration group should be added. Results should be sent stepwise as they become available.

### Laboratory Register for culture and DST

Laboratories will have separate registers for sputum smear microscopy and culture, while reference laboratories carrying out DST should have additional space in the culture register for DST results (see Form 04). The Laboratory Register should be compared regularly with the DR-TB Register to ensure that all confirmed MDR-TB cases are entered in the DR-TB Register.

### Quarterly report on MDR-TB detection and DR-TB treatment

This report is used to assess the number of MDR-TB cases detected (distribution and trends) and the number of MDR-TB cases who start treatment. The report should be made quarterly in line with the routines of the NTCP. The report should be made by the unit managing MDR-TB. The quarterly report includes:

- The number of patients, with date of result showing MDR-TB during the relevant quarter taken from the Laboratory Register. Optionally, the patients could be split by registration group.
- The number of MDR-TB patients started on DR-TB treatment during the quarter, taken from the DR-TB Register.
- If relevant, the number of XDR-TB cases registered (after cross-checking DST results with type of resistance) and the number of XDR-TB cases started on XDR-TB treatment should be added. Since there may be a considerable delay between DR-TB registration and the start of DR-TB treatment, patients who start treatment during the quarter may not be the same as those detected with DR-TB. The information provides an approximation of treatment coverage. The NTCP will calculate the average delay between detection of DR-TB and treatment start.

### Six-month interim outcome assessment of confirmed MDR-TB case (Form 11)

Considering the fact that, MDR-TB treatment takes on average two years before final results are known, the NTCP needs updated information on treatment outcome. The Interim Outcome result form can be used to report bacteriological status (negative, positive or no information) of those still on treatment at 6 months, and for those who have already lost to follow up, died or transferred out, this can be recorded as the final outcome.

Bacteriological results are based on the smear and culture data during months 5 and 6 of treatment. Consider the 6-month outcome assessment unknown for a particular patient if a culture or smear result is unknown for either month 5 or 6.

All cases from the DR-TB Register should be included in this report. The form should be completed 9 months after the closing day of the cohort. This allows culture information at month 6 of treatment to be included for all patients in the cohort. For instance, TB patients who started treatment during the first quarter of a year (1 January to 31 March), should have the form filled in from 1 January of the following year.

### Annual report of treatment result of confirmed MDR-TB patients starting DR-TB treatment

This report is made by the central unit and shows the final result of treatment by year of treatment start. All the patients are classified by previous use of anti-tuberculosis drugs (none, only first-line drugs, also second-line drugs). If relevant, results for patients with XDR-TB could be added. All data can be extracted from treatment cards and DR-TB Register. Form 07 is first completed at 24 months after the last patient in the cohort started treatment. Most of the patients will have finished treatment by 24 months, allowing preliminary assessment of cure rates. Since a few patients may be on treatment for longer than 24 months, the form may be completed again at 36 months, which will then be considered the final result.

# ANNEXES

## Algorithm for management of depression:

## Algorithm for management of Nausea and vomiting:

## Algorithm for management of Hepatitis:

## Algorithm for management of Hypothyroidism:

## Algorithm for management of Nephrotoxicity:

## Algorithm for management of Psychosis:

## Algorithm for management of Hypolalaemia:

## Algorithm for management of Haemoptysis:

## Algorithm for management of Haemoptysis -2:

## Algorithm for management of respiratory insufficiency:

**Algorithm for management of respiratory insufficiency:**

**Algorithm for management of respiratory insufficiency - 2:**

# REFERENCES

Al-Dabbagh, Mona, Keswadee Lapphra, Rumi McGloin, Kelsey Inrig, H. Simon Schaaf, Ben J. Marais, Laura Sauve, Ian Kitai, y Tobias R. Kollmann. «Drug-resistant Tuberculosis». The Pediatric Infectious Disease Journal (February 2011): 1.

Francis J. Curry National Tuberculosis Centre 2007. Tuberculosis Infection Control, A practical manual for preventing TB. [www.nationaltbcenter.edu](http://www.nationaltbcenter.edu)

International Standards for Tuberculosis Care World Health Organization; 2006. <http://www.who.int/tb/publications/2006/istc/en/index.html>

Ministry of Health Swaziland: National Guidelines for Isoniazid (INH) Prophylaxis; 2010.

Paed(Stellenbosch), H. Simon Schaaf MBChB(Stellenbosch) Mmed Paed(Stellenbosch) DCM(Stellenbosch) MD, y Alimuddin Zumla BSc.MBChB.MSc.PhD.FRCP(Lond).FRCP(Edin).FRCPath(UK), eds. Tuberculosis: A Comprehensive Clinical Reference, 1e. 1.a ed. Saunders, 2009.

PIH/WHO 2010. Management of MDR-TB: A field guide. WHO/HTM/TB/2008.402a. <http://whqlibdoc.who.int/publications/2009/9789241547765_eng.pdf>

The UNION 1998: Epidemiological basis of tuberculosis control. First Edition1999: p.8.

WHO 2008. Guidelines for the programmatic management of drug-resistant tuberculosis. WHO/HTM/TB/2008.402. <http://whqlibdoc.who.int/publications/2008/9789241547581_eng.pdf>

WHO 2008: Implementing the WHO Stop TB strategy: A handbook for national tuberculosis control programmes. World Health Organization; 2008.WHO/HTM/TB/2008.401 <http://www.who.int/tb/publications/2008/en/index.html>

WHO 2009: WHO Policy on TB infection control in health-care facilities, congregate settings, and households. 2009. WHO/HTM/TB/2009.419 <http://www.who.int/tb/publications/2009/en/index.html>

WHO 2010. Antiretroviral drugs for treating pregnant women and preventing infection in infants. Recommendations for a public health approach. 2010 Revision. WHO/HTM/HIV/2010.

WHO 2010. Antiretroviral therapy for HIV infection in infants and Children towards universal access: Recommendations for a public health approach. 2010 Revision. WHO/HTM/HIV/2010.

WHO 2010. Guidance on ethics of tuberculosis prevention, care and control. WHO/HTM/TB/2010.16. <http://whqlibdoc.who.int/publications/2010/9789241500531_eng.pdf>

WHO 2010. Multidrug-resistant tuberculosis (MDR-TB) indicators: A minimum set of indicators for the programmatic management of MDR-TB national tuberculosis control programmes. WHO/HTM/TB/2010.11

WHO 2010. Rapid Advice on the treatment of tuberculosis in children. WHO/HTM/TB/2010.13. <http://whqlibdoc.who.int/publications/2010/9789241500449_eng.pdf>

WHO 2010: Guidelines for intensified tuberculosis case-finding and isoniazid preventive therapy for people living with HIV in resource-constrained settings. World Health Organization; 2010, pp. 14. WHO/HTM/TB 2010.

WHO 2010: Multidrug and extensively drug-resistant TB (M/XDR-TB): 2010 global report on surveillance and response. WHO/HTM/TB/2009.3. <http://whqlibdoc.who.int/publications/2010/9789241599191_eng.pdf>

WHO 2010: The Global Plan to Stop TB 2011-2015. <http://www.stoptb.org/global/plan/>

WHO 2011. Commercial serodiagnostic tests for diagnosis of tuberculosis:
policy statement. WHO/HTM/TB/2011.5

WHO 2011. Guidelines for the programmatic management of drug-resistant tuberculosis - 2011 update. WHO/HTM/TB/2011.6. <http://www.who.int/tb/challenges/mdr/programmatic_guidelines_for_mdrtb/en/index.html>

WHO 2011. Use of tuberculosis interferon-gamma release assays (IGRAs) in low- and middle-income countries: Policy Statement. WHO/HTM/TB/2011.18

WHO 2011: Global Tuberculosis Control 2011. WHO/HTM/TB/2011.16. <http://www.who.int/tb/publications/global_report/en/index.html>

WHO 2011: Rapid implementation of the Xpert MTB/Rif diagnostic test. Technical and operational ‘How to’ Practical considerations. WHO/HTM/TB/2011.2 <http://whqlibdoc.who.int/publications/2011/9789241501569_eng.pdf>

WHO 2011: Towards universal access to diagnosis and treatment of multidrug-resistant and extensively drug-resistant tuberculosis by 2015. WHO progress report 2011. WHO/HTM/TB/2011.3. <http://www.who.int/entity/tb/publications/2011/mdr_report_2011/en/index.html>

WHO 2012: Meeting Report. First meeting of the Global GLC. WHO/HTM/TB/2012.2 <http://www.who.int/entity/tb/publications/Meetingreport1stgGLCmeeting.pdf>

WHO 2012: WHO policy on collaborative TB/HIV activities: guidelines for national programmes and other stakeholders. WHO/HTM/TB/2012.1. <http://www.who.int/entity/tb/publications/2012/tb_hiv_policy_9789241503006/en/index.html>

WHO2009. Treatment of tuberculosis guidelines. Fourth edition. WHO/HTM/TB/2009.420. <http://whqlibdoc.who.int/publications/2010/9789241547833_eng.pdf>

[
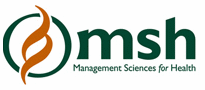
](http://www.msh.org/)


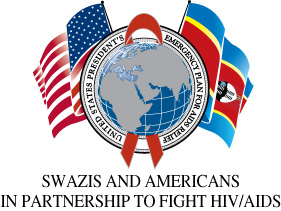


[
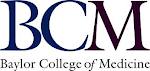
](http://www.google.com/imgres?imgurl=http://ecosystem.news-sap.com/files/2011/01/BCM_Logo_RGB_1191708351.png&imgrefurl=http://www.news-sap.com/baylor-college-of-medicine-cures-it-ailments-with-help-from-sap-communities/&usg=__9mlejhJNfy7VDR2LsGFMEzsSVAI=&h=1412&w=2992&sz=165&hl=en&start=1&zoom=1&tbnid=YrhArPXk-N4uLM:&tbnh=71&tbnw=150&ei=VVonUNHpCIS0hAfqgoDYCw&prev=/images?q=baylor+college+of+medicine+logo&hl=en&gbv=2&tbm=isch&itbs=1)
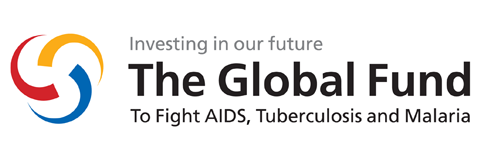


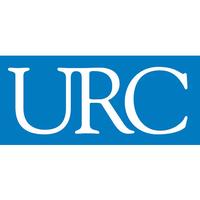


[
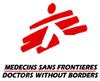
](http://www.google.co.za/imgres?imgurl=http://www.isrreports.com/images/content_images/graphic_logo_msf.gif&imgrefurl=http://www.isrreports.com/about-us/community-involvement&h=102&w=126&sz=2&tbnid=IWdrcd06xDhSFM:&tbnh=81&tbnw=100&prev=/search?q=MSF+Logo&tbm=isch&tbo=u&zoom=1&q=MSF+Logo&usg=__QvqCyKLa0bZc_MubNH3egwSKMNA=&docid=SgBi2fn67Pc81M&sa=X&ei=5f5NUPXeFIGy8QTVwICgDQ&ved=0CCsQ9QEwAw&dur=2951)
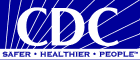

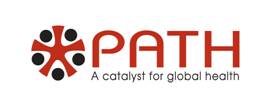

Supplement: Supplementary file 1 — National drug-resistant tuberculosis management guidelines. (DOCX 1345 kb) [file 12879_2019_4384_MOESM1_ESM.docx]
