# Supplementary material for: Factors associated with isoniazid resistant tuberculosis among human immunodeficiency virus positive patients in Swaziland: a case-control study
Source: BMC Infect Dis. 2019 Aug 20;19:731. doi: 10.1186/s12879-019-4384-6 (PMC6701091; doi:10.1186/s12879-019-4384-6)
Supplement: Supplementary file 2 — National tuberculosis programmemanual. (DOCX 1617 kb) [file 12879_2019_4384_MOESM2_ESM.docx]

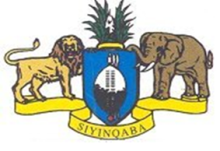


**Kingdom of Swaziland**

**MINISTRY OF HEALTH**

**NATIONAL TUBERCULOSIS PROGRAMME**

**MANUAL**

**
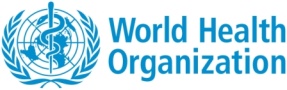
**

**
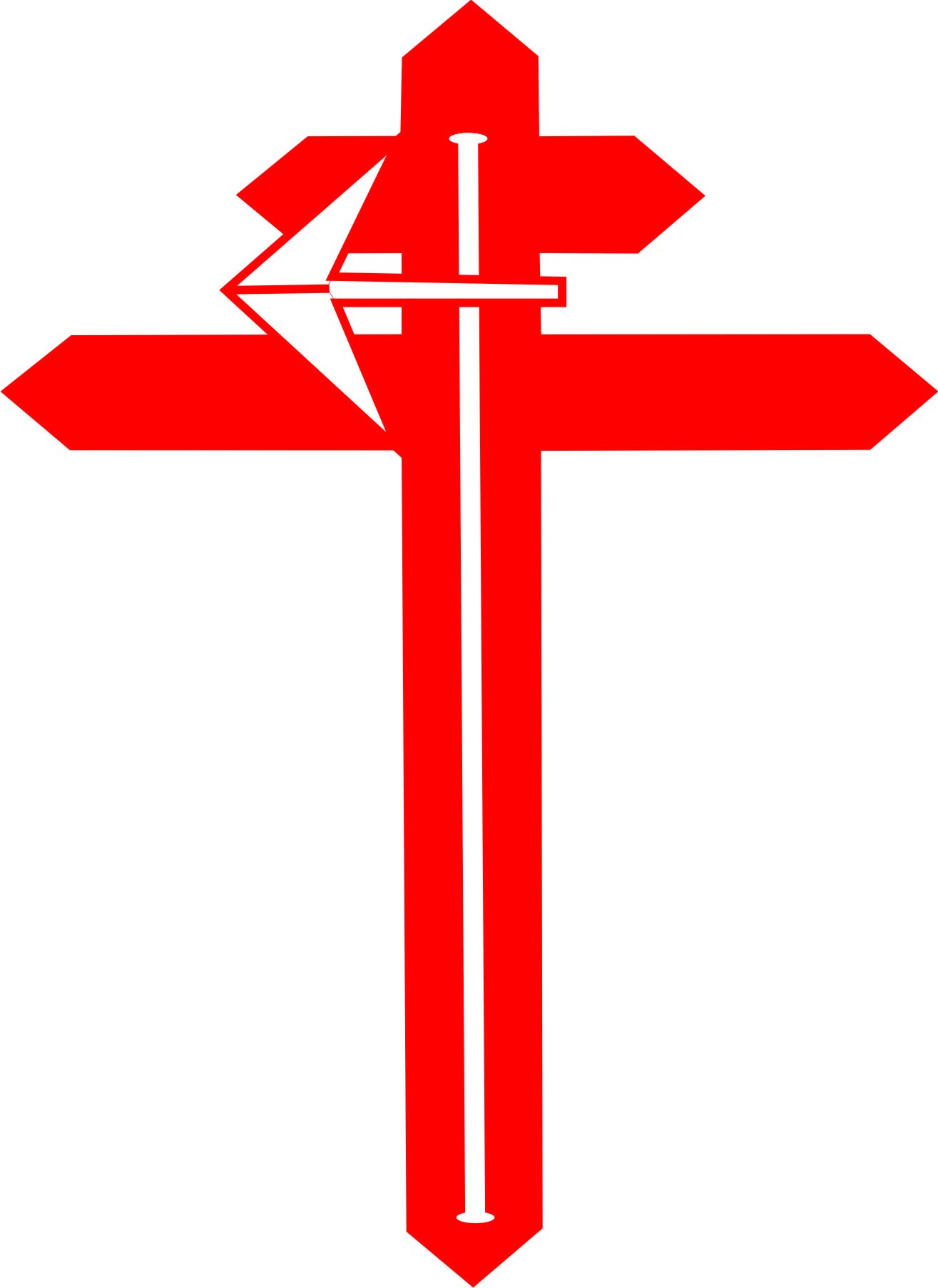
**

National Tuberculosis Control Programme

Printed in 2012

Publication notice

Publication of the Swaziland National Tuberculosis Control Programme. All rights reserved.

Any enquiries regarding this publication or permission to reproduce or translate the material should be addressed to National Tuberculosis Control Programme,

P.O Box 54 Manzini. Telephone +268-25057951, 2505-7454

or e-mail [publications@tbprogram.org.sz](mailto:publications@tbprogram.org.sz); [dlaminit@tbprogram.org.sz](mailto:dlaminip@tbprogram.org.sz)

CONTENTS

[PREFACE 8](#_Toc335208295)

[FOREWORD 9](#_Toc335208296)

[ACKNOWLEDGEMENT 10](#_Toc335208297)

[EXECUTIVE SUMMARY 12](#_Toc335208298)

[1.1 Purpose of the guidelines 12](#_Toc335208299)

[1.2 Target audience 12](#_Toc335208300)

[1.3 Scope 12](#_Toc335208301)

[1.4 Justification for guideline update 12](#_Toc335208302)

[1.5 Highlights of the new revision 13](#_Toc335208303)

[CHAPTER 1 INTRODUCTION 16](#_Toc335208304)

[1.1 The country 16](#_Toc335208305)

[1.2 The health system 16](#_Toc335208306)

[CHAPTER 2 THE NATIONAL TUBERCULOSIS CONTROL PROGRAMME 18](#_Toc335208307)

[2.1 Global Epidemiology of TB 18](#_Toc335208308)

[2.2 The Tuberculosis situation in Swaziland 18](#_Toc335208309)

[2.3 The National TB Control Programme 19](#_Toc335208310)

[2.4 The TB Control Strategy and framework for Swaziland 19](#_Toc335208311)

[2.4.1 Central Unit 19](#_Toc335208312)

[2.4.2 Regional Level 19](#_Toc335208313)

[2.4.3 Basic TB Management Units (BMUs) 20](#_Toc335208314)

[2.4.4 The National TB Hospital 20](#_Toc335208315)

[2.4.5 Intra-ministerial collaboration 20](#_Toc335208316)

[2.4.6 NTP Partners 21](#_Toc335208317)

[2.4.7 NTP funding 21](#_Toc335208318)

[2.5 Goals, Objectives and targets of the National TB Programme 21](#_Toc335208319)

[2.5.1 The Objectives: 21](#_Toc335208320)

[2.5.2 The targets: 21](#_Toc335208321)

[2.5.3 Programme Goal 22](#_Toc335208322)

[2.5.4 Objectives 22](#_Toc335208323)

[2.5.5 Swaziland Stop TB Partnership targets 22](#_Toc335208324)

[CHAPTER 3 TB CASE FINDING AND REGISTRATION 23](#_Toc335208325)

[3.1 Importance of TB case finding and registration 23](#_Toc335208326)

[3.2 Identification of persons with presumptive pulmonary TB: 23](#_Toc335208327)

[3.2.1 Sputum Collection, Labeling, Storage and Transport 23](#_Toc335208328)

[3.2.1.1 Sputum collection procedure 24](#_Toc335208329)

[3.2.1.2 Sputum labeling 24](#_Toc335208330)

[3.2.1.3 Sputum storage 24](#_Toc335208331)

[3.2.1.4 Transportation of sputum specimens 24](#_Toc335208332)

[3.3 Identification of persons with presumtive Extra-Pulmonary TB: 25](#_Toc335208333)

[3.3.1 Common types of Extra-pulmonary TB 25](#_Toc335208334)

[3.4 Diagnosis and management of TB in Children: 26](#_Toc335208335)

[3.5 Defining a case of tuberculosis 26](#_Toc335208336)

[3.5.1 The importance of case definition 26](#_Toc335208337)

[3.5.2 The TB case definitions 26](#_Toc335208338)

[3.6 TB patients registration groups 27](#_Toc335208339)

[3.6.1 Site of TB disease 27](#_Toc335208340)

[3.6.2 Bacteriology (result of sputum smear) in pulmonary TB 27](#_Toc335208341)

[3.6.3 History of previous treatment 27](#_Toc335208342)

[New patients: 27](#_Toc335208343)

[CHAPTER 4 DIAGNOSIS OF TB 29](#_Toc335208344)

[4.1 Approach to TB diagnosis 29](#_Toc335208345)

[4.2 Bacteriological confirmation of pulmonary TB diagnosis 29](#_Toc335208346)

[4.2.1 Microscopy 29](#_Toc335208347)

[4.2.2 Xpert MTB/Rif (Gene Xpert) test 30](#_Toc335208348)

[4.2.2.1 Use of Xpert MTB/Rif in new TB cases 30](#_Toc335208349)

[4.2.2.2 Use of Xpert MTB/Rif in previously treated TB cases 30](#_Toc335208350)

[4.2.2.3 Practical considerations for use of Xpert MTB/Rif 31](#_Toc335208351)

[4.2.2.4 Use of Xpert MTB/Rif results 31](#_Toc335208352)

[4.2.2.5 Repeat of Gene Xpert test 31](#_Toc335208353)

[4.2.3 Use of interferon gamma tests: 32](#_Toc335208354)

[4.2.4 Use of Commercial sero-diagnostics 32](#_Toc335208355)

[4.3 Diagnosis of drug resistant tuberculosis 32](#_Toc335208356)

[4.3.1 Line Probe Assay (LPA) method 32](#_Toc335208357)

[4.3.2 Mycobacterial culture and drugs susceptibility testing (DST) 33](#_Toc335208358)

[4.3.2.1 Thin Layer Agar (TLA) culture and DST method 33](#_Toc335208359)

[4.4 Sputum transportation 33](#_Toc335208360)

[4.5 Role of Other Investigations in TB Control 34](#_Toc335208361)

[4.5.1 Role of Chest x-rays 34](#_Toc335208362)

[4.6 Revised diagnostic Algorithms according to existing TB diagnostic equipment in Swaziland 34](#_Toc335208363)

[4.6.1 Diagnostic Algorithm for settings without Xpert MTB/Rif 35](#_Toc335208364)

[4.6.2 Diagnostic Algorithm for settings with Xpert MTB/Rif 36](#_Toc335208365)

[4.7 Confirming diagnosis of Extra-Pulmonary TB 37](#_Toc335208366)

[4.7.1 Tuberculous Lymphadenopathy 38](#_Toc335208367)

[4.7.2 Miliary TB 38](#_Toc335208368)

[4.7.3 Tuberculous pleural effusions 38](#_Toc335208369)

[4.7.4 TB Meningitis 39](#_Toc335208370)

[4.7.4.1 Clinical Features 39](#_Toc335208371)

[4.7.4.2 Laboratory diagnosis 39](#_Toc335208372)

[4.7.4.3 Treatment of TB Meningitis (TBM) 39](#_Toc335208373)

[4.7.5 Tuberculosis of the spine 40](#_Toc335208374)

[CHAPTER 5 QUALITY ASSURANCE OF LABORATORY SERVICES 41](#_Toc335208375)

[5.1 Importance of Laboratory Services in TB Control 41](#_Toc335208376)

[5.2 TB laboratory Network in Swaziland 41](#_Toc335208377)

[5.2.1 The National Reference laboratory 41](#_Toc335208378)

[5.2.2 The Regional laboratories 42](#_Toc335208379)

[5.3 Assuring Quality of Smear Microscopy 42](#_Toc335208380)

[5.3.1 External Quality Assessment (EQA) 43](#_Toc335208381)

[5.3.2 On-site evaluation of Microscopy Centers: 43](#_Toc335208382)

[5.3.3 Panel Testing 44](#_Toc335208383)

[5.3.4 Random Blinded Rechecking of Routine Slides 44](#_Toc335208384)

[5.4 Conducting visits to microscopy centres 45](#_Toc335208385)

[5.4.1 Preparing for visits: 45](#_Toc335208386)

[5.4.2 Conducting the visit 45](#_Toc335208387)

[5.4.3 Follow up Quality improvement 45](#_Toc335208388)

[5.5 Monitoring documentation related to microscopy examinations and other diagnostic methods 45](#_Toc335208389)

[5.5.1 Laboratory Request form 46](#_Toc335208390)

[5.5.2 Tuberculosis laboratory register 46](#_Toc335208391)

[5.5.3 Disposal of laboratory materials. 47](#_Toc335208392)

[CHAPTER 6 TREATMENT OF TUBERCULOSIS 48](#_Toc335208393)

[6.1 The aims of TB treatment 48](#_Toc335208394)

[6.2 Essential anti-TB drugs 48](#_Toc335208395)

[6.2.1 Fixed-Dose Combinations 48](#_Toc335208396)

[6.2.2 Advantages of FDCs compared to single formulation drugs 48](#_Toc335208397)

[6.3 Standard TB Treatment Regimens for Adults and adolescents 48](#_Toc335208398)

[6.3.1 Treatment of New tuberculosis cases – Adults and adolescents 50](#_Toc335208399)

[6.3.2 Treatment of previously treated cases 50](#_Toc335208400)

[6.3.3 Treatment of extra-pulmonary tuberculosis 51](#_Toc335208401)

[6.3.3.1 Treatment of TB meningitis 51](#_Toc335208402)

[6.3.4 Important drug to drug interactions 51](#_Toc335208403)

[6.3.5 TB Treatment regimens in special circumstances 52](#_Toc335208404)

[6.3.5.1 Treatment for pregnant women 52](#_Toc335208405)

[6.3.5.2 Treatment for breastfeeding women 52](#_Toc335208406)

[6.3.5.3 Treatment for women taking the oral contraceptive pill 52](#_Toc335208407)

[6.3.5.4 Treatment for patients with liver disorders 52](#_Toc335208408)

[6.3.5.5 Treatment of patients with renal failure 53](#_Toc335208409)

[6.4 The Role of Adjuvant Steroid Treatment 53](#_Toc335208410)

[CHAPTER 7 PATIENT SUPPORT AND DOT PROVISION 55](#_Toc335208411)

[7.1 Importance of patient support 55](#_Toc335208412)

[7.2 Community Based DOTS (CB DOTS) 55](#_Toc335208413)

[7.2.1 Identification of treatment supporter 55](#_Toc335208414)

[7.2.2 Orientation of treatment supporters 56](#_Toc335208415)

[7.2.3 Provide enough drugs to last until the next visit. 56](#_Toc335208416)

[7.2.4 Keep regular contact with the patient and supporter 56](#_Toc335208417)

[7.2.5 What to do during the visit of the supporter / patient to health facility? 56](#_Toc335208418)

[7.3 Prevention of treatment interruption 56](#_Toc335208419)

[7.3.1 Role of Adherence Officers 57](#_Toc335208420)

[7.4 Nutritional support to TB patients 57](#_Toc335208421)

[7.4.1 Food by prescription 57](#_Toc335208422)

[7.4.2 Food Prescription Initiation procedure 57](#_Toc335208423)

[CHAPTER 8 MONITORING TUBERCULOSIS TREATMENT 60](#_Toc335208424)

[8.1 Basis for monitoring TB treatment 60](#_Toc335208425)

[8.1.1 Clinical monitoring: 60](#_Toc335208426)

[8.1.1.1 Monitoring Extra-pulmonary TB treatment 60](#_Toc335208427)

[8.1.2 Bacteriological monitoring: 60](#_Toc335208428)

[8.1.2.1 New sputum smear-positive pulmonary TB patients 61](#_Toc335208429)

[8.1.2.2 Previously treated pulmonary sputum smear-positive patients 61](#_Toc335208430)

[8.1.2.3 New sputum smear-negative pulmonary TB patients 61](#_Toc335208431)

[8.2 Management of treatment interruption 64](#_Toc335208432)

[8.2.1 Monitoring of TB Patients for Adverse Effects of Anti-TB Drugs 65](#_Toc335208433)

[8.2.1.1 Prevention of adverse effects of drugs 65](#_Toc335208434)

[8.2.1.2 Adverse effects of anti-tuberculosis drugs 65](#_Toc335208435)

[8.2.1.3 Symptom-based approach to management of drug side-effects 65](#_Toc335208436)

[8.2.1.4 Management of skin itching and rash 66](#_Toc335208437)

[8.2.1.5 Reintroduction of anti-TB drugs following drug reaction 66](#_Toc335208438)

[8.3 Determining TB Treatment Outcomes 67](#_Toc335208439)

[CHAPTER 9 MANAGEMENT OF CHILDHOOD TUBERCULOSIS 68](#_Toc335208440)

[9.1 Importance of childhood TB 68](#_Toc335208441)

[9.2 Approach to diagnosis of TB in children 68](#_Toc335208442)

[9.2.1 Evaluation for paediatric TB disease 69](#_Toc335208443)

[9.2.2 Clinical examination (including growth assessment) 70](#_Toc335208444)

[9.3 Special Investigations for paediatric TB 70](#_Toc335208445)

[9.3.1 Chest X-ray 70](#_Toc335208446)

[9.3.2 Role of a Tuberculin Skin Test 71](#_Toc335208447)

[9.3.3 Bacteriological confirmation of childhood TB 72](#_Toc335208448)

[9.3.3.1 Techniques for obtaining specimens from children 72](#_Toc335208449)

[9.4 Paediatric Extrapulmonary TB 73](#_Toc335208450)

[9.4.1 TB meningitis 73](#_Toc335208451)

[9.4.2 Lymph node TB 73](#_Toc335208452)

[9.4.3 Abdominal TB 74](#_Toc335208453)

[9.4.4 Bone and joint disease 74](#_Toc335208454)

[9.4.5 BCG disease 74](#_Toc335208455)

[9.5 Paediatric DR-TB 75](#_Toc335208456)

[9.6 PadediatricTB treatment 75](#_Toc335208457)

[9.6.1 Treatment of susceptible paediatric TB 75](#_Toc335208458)

[9.6.2 Use of Steroids in Pediatric TB forms 76](#_Toc335208459)

[9.6.3 Paediatric anti-TB drugs dosage 76](#_Toc335208460)

[9.7 Paediatric MDR-TB treatment 77](#_Toc335208461)

[9.8 Follow up of children on TB treatment 77](#_Toc335208462)

[9.9 TB-HIV COINFECTION 78](#_Toc335208463)

[9.9.1 Anti-Retroviral Therapy 78](#_Toc335208464)

[9.9.1.1 Recommended regimens for HIV+ pediatric patients on TB treatment: 78](#_Toc335208465)

[9.9.1.2 Alternative ART regimen options for special situations requiring TB/HIV co-treatment: 78](#_Toc335208466)

[9.9.2 Cotrimoxazole Prophylaxis 79](#_Toc335208467)

[9.9.3 Administering treatment and ensuring adherence 79](#_Toc335208468)

[9.10 Prevention of Paediatric TB 79](#_Toc335208469)

[9.10.1 Paediatric Isoniazid Preventive Therapy 80](#_Toc335208470)

[CHAPTER 10 COLLABORATIVE TB AND HIV ACTIVITIES 81](#_Toc335208471)

[10.1 TB/HIV interaction 81](#_Toc335208472)

[10.2 HIV Testing and Counseling (HTC) 81](#_Toc335208473)

[10.3 HIV prevention in TB patients 81](#_Toc335208474)

[10.4 TB treatment in people living with HIV 81](#_Toc335208475)

[10.5 Co-trimoxazole preventive therapy 82](#_Toc335208476)

[10.6 Antiretroviral therapy 82](#_Toc335208477)

[10.6.1 Interactions with ART Regimens 82](#_Toc335208478)

[10.6.2 When to start ART? 83](#_Toc335208479)

[10.7 Drug susceptibility testing 83](#_Toc335208480)

[10.8 Dealing with TB diagnosed in patients already on ART 84](#_Toc335208481)

[10.9 HIV-related prevention, treatment, care and support 84](#_Toc335208482)

[10.9.1.1 Managing side effects in concurrent TB/HIV treatment 84](#_Toc335208483)

[10.9.1.2 Directly Observed Therapy for concomitant TB/HIV treatment 84](#_Toc335208484)

[10.9.1.3 Monitoring patients on concurrent ART and DOTS 84](#_Toc335208485)

[10.9.1.4 Immune Reconstitution Inflammatory Syndrome among patients with HIV-related TB 86](#_Toc335208486)

[CHAPTER 11 TB INFECTION CONTROL 87](#_Toc335208487)

[11.1 When is TB infectious? 87](#_Toc335208488)

[11.1 Rationale for TB Infection Control 87](#_Toc335208489)

[11.2 Infection Control measures 87](#_Toc335208490)

[11.2.1 Administrative infection control measures 87](#_Toc335208491)

[11.2.1.1 Infection Control plan 88](#_Toc335208492)

[11.2.2 Environmental control measures 88](#_Toc335208493)

[11.2.3 Personal respiratory protection 89](#_Toc335208494)

[11.3 High Risk areas 90](#_Toc335208495)

[11.4 Measures to reduce infection transmission in community settings where there is congregation 90](#_Toc335208496)

[11.5 TB infection control in Prisons 90](#_Toc335208497)

[CHAPTER 12 MANAGEMENT OF MULTI-DRUG AND EXTENSIVELY RESISTANT TUBERCULOSIS 91](#_Toc335208498)

[12.1 Definitions 91](#_Toc335208499)

[12.2 Causes of MDR-TB 91](#_Toc335208500)

[12.3 When to Suspect MDR-TB 92](#_Toc335208501)

[12.4 Laboratory Confirmation of MDR-TB 92](#_Toc335208502)

[12.5 Management of DR TB 93](#_Toc335208503)

[CHAPTER 13 SUPERVISION, MONITORING AND EVALUATION 94](#_Toc335208504)

[13.1 Importance of SME 94](#_Toc335208505)

[13.2 Programme supervision 94](#_Toc335208506)

[13.2.1 Monitoring tools 95](#_Toc335208507)

[13.3 Programme monitoring 95](#_Toc335208508)

[13.3.1 Programme indicators: 96](#_Toc335208509)

[13.3.1.1 Cohort Analysis 96](#_Toc335208510)

[13.3.1.2 Quarterly Report on Case Finding 96](#_Toc335208511)

[13.3.1.3 Quarterly Report on Treatment Outcome 97](#_Toc335208512)

[13.3.1.4 Information Flow 97](#_Toc335208513)

[13.4 Programme evaluation 97](#_Toc335208514)

[13.5 Reporting and Recording system 97](#_Toc335208515)

[CHAPTER 14 ADVOCACY, COMMUNICATION, SOCIAL MOBILISATION 100](#_Toc335208516)

[14.1 Introduction 100](#_Toc335208517)

[14.2 Communication as an overarching theme 100](#_Toc335208518)

[14.3 Advocacy to change political agendas 100](#_Toc335208519)

[14.4 Social mobilization to build partnerships 100](#_Toc335208520)

[14.5 Selection of Advocacy Strategies and Tactics 101](#_Toc335208521)

[14.5.1 Media strategy 101](#_Toc335208522)

[14.5.2 NTP Publications strategy 101](#_Toc335208523)

[14.5.3 Coalitions and working with NGOs 101](#_Toc335208524)

[14.5.4 Insider strategy 102](#_Toc335208525)

[14.6 Message Development and Presentation 102](#_Toc335208526)

[14.7 Role of NGOs , Private Sector and communities in TB control and involvement in social mobilisation 102](#_Toc335208527)

[ANNEXES 105](#_Toc335208528)

[ANNEX 1: TB Programme Organogram 105](#_Toc335208529)

[ANNEX 2: Stop TB Partnership Organogram 106](#_Toc335208530)

[ANNEX 3: SPUTUM COLLECTION 107](#_Toc335208531)

[ANNEX 4: Prerequisites for implementation of XPERT MTB/Rif 108](#_Toc335208532)

[ANNEX 5: Key recommended actions at country level for Xpert MTB/Rif implementation 109](#_Toc335208533)

[ANNEX 6: BASIC GUIDE TO CXR READING 111](#_Toc335208534)

[ANNEX 7: TUBERCULIN SKIN TEST (TST) 112](#_Toc335208535)

[ANNEX 8: FINE NEEDLE ASPIRATION (FNA) 113](#_Toc335208536)

[ANNEX 9: GASTRIC ASPIRATE PROCEDURE FOR CULTURE OFM. *tuberculosis* 115](#_Toc335208537)

[14.8 Assure sample is well labeled and all forms correctly filled before sending the material to the laboratory.ANNEX 10: INDUCED SPUTUM IN CHILDREN 116](#_Toc335208538)

[ANNEX 11: PERFORMINGTHORACENTESIS 120](#_Toc335208539)

[ANNEX 12: PERFORMING LUMBAR PUNCTURE IN ADULTS 122](#_Toc335208540)

[ANNEX 13:PERFORMING LUMBAR PUNCTURE IN CHILDREN 124](#_Toc335208541)

[ANNEX 14: INFECTION CONTROL RISK ASSESSMENT TOOL 126](#_Toc335208542)

[ANNEX 15: HIV TESTING MODELS 127](#_Toc335208543)

[REFERENCES 129](#_Toc335208544)

**List of tables:**

[Table 1.3: Summary of Health Service indicators 19](#_Toc335075949)

[Table 2.1: Table showing symptom-based approach to the diagnosis of Extra-pulmonary TB 39](#_Toc335075950)

[Table 3: Summary of TB treatment regimens for adult cases 52](#_Toc335075951)

[Table 4.5: Prednisolone indication and recommended doses in TB management 56](#_Toc335075952)

[Table 5.1: Recommended Schedule for follow up sputum examinations for PTB patients 64](#_Toc335075953)

[Table 6: Sputum follow-up algorithm for patients on anti-TB treatment 66](file:///C:\Users\samsonk\Documents\TB%20Guidelines%20-%20Print%20version%20Mar%202012-1\Swaziland%20TB%20Manual-%20Print%20version_11%20SEP%202012.docx#_Toc335075954)

[Table 7.4: Guide to performing anti-TB drug challenge and re-introduction 70](#_Toc335075955)

[Table 9:Recommended Regimens for Pediatric Patients on TB treatment 82](#_Toc335075956)

[Table 10: Alternative Paediatric Regimens for Special Situations 82](#_Toc335075957)

[Table 11: Dosing for Cotrimoxazole* 83](#_Toc335075958)

[Table 12: Dosage of INH for prophylaxis in Children 84](#_Toc335075959)

[Table 13: Anti-TB drugs /ARV Regimen Recommendations 86](#_Toc335075960)

[Table 14: Overlapping Side effect adverse reactions to First-line anti-TB and ART drugs 90](#_Toc335075961)

[Table 15: Causes of DRTB 96](#_Toc335075962)

[Table 16: Recording and reporting formats used in the National TB Programme 102](#_Toc335075963)

[Table 17: Analysis of training needs at various levels of the NTP 133](#_Toc335075964)

# PREFACE

Since the World Health Organization declared tuberculosis (TB) a global emergency in 1993, worldwide efforts to fight the disease have intensified considerably. In Swaziland, TB is associated with HIV and AIDS co-infection, social problems, difficulties in patient adherence and the threat of resistance against anti-tuberculous drugs. This is of great concern to the country, hence the need for the National Tuberculosis Control Programme (NTP) of Swaziland to be adequately positioned to face the challenges of controlling the disease.

The commitment of the Government of Swaziland to eliminate TB as a public health problem has resulted in strengthening the NTP at both national and Regional levels and increased allocation of funds to ensure universal access to quality TB services in line with the Abuja declaration. It should be mentioned here, that the efforts of the SG has been enhanced through 2 grants from the Global Fund to Fight AIDS, TB and Malaria (GFATM), which enabled rapid expansion of access to TB diagnostic and treatment services. Furthermore, a formidable in-country partnership has been built for TB which will ensure sustained action in a synergistic manner.

I note with great sense of fulfillment that Swaziland is among the countries of the world that have reached and even exceeded the 70% target for detection of infectious TB cases. However, the treatment success rate of 73% falls below the set target of 85%, which requires urgent implementation of the African TB emergency declaration to improve treatment outcomes.

The NTP produced the first edition of the Tuberculosis Control manual in 2006, am I am convinced that the document has been instrumental to the achievements of the programme so far.

This 2^nd^ Edition which takes into consideration the most recent developments in TB control especially with respect to TB diagnosis and management under the influence of HIV/AIDS, MDR/XDR-TB management, management of TB in Children and issues of laboratory quality-assurance will no doubt add value to the quality of TB care in the country. I therefore congratulate the NTCP and partners for realizing the publication of this second edition, which is sure a product of considerable hard work.

I would like to reiterate that the Ministry of Health considers TB control a priority and is strongly committed to the fight against TB, and will continue to play facilitatory role towards achieving the MDG targets for TB by 2015.

Mr. Benedict Xaba

**Honourable Minister of Health**

# FOREWORD

This manual describes the policy direction as well as guidelines for the diagnosis and management of tuberculosis patients in the Kingdom of Swaziland. It is also meant to serve as a guide for clinicians and other health workers involved in the management of tuberculosis patients with view to optimizing the quality of care for our people.

The clinical knowledge, policy guidelines and programme organization reflected in this document are derived from the strategic orientation of the World Health Organization (WHO), the Stop TB partnership, and other key stakeholders in the global tuberculosis control as reflected in the Stop TB Strategy.

This manual does not intend to provide the kind of comprehensive clinical knowledge on tuberculosis as obtainable from clinical textbooks. The manual, however, does intend to address the major elements relevant to addressing TB as a major public health problem in line with global and regional orientations; and in a user-friendly manner.

While the manual in its entirety is appropriate for TB control programme players at various levels, general practitioners and non-TB specialists may find the manual equally useful for quick reference on specific TB, TB/HIV and MDR-TB management topics.

Likewise, some of its chapters are relevant and useful for policy-makers and for our valuable partners who also support TB control as a public health initiative.

Specifically, the manual is composed of 14 chapters which, taken as a whole, provide an overview of TB control. Together, the chapters cover a broad spectrum of topics ranging from epidemiology, diagnosis, treatment, TB/HIV co-infection and MDR/XDR-TB management to topics such as TB control programme strategy and organization, Advocacy, communication and TB laboratory networks. Individual chapters may be easily consulted as reference on various topics and subtopics, such as case definitions, extra-pulmonary TB, BCG vaccination, managing TB risk groups and adverse anti-TB drug reactions.

In order to keep the manual to a practical length, repetition of the content has been kept to a minimum, and readers should refer to the index for further reference. Furthermore details of the management of other related conditions e.g. HIV and AIDS, MDR-TB, Infection Control etc should be sought in their respective guidelines.

It is our hope that all stakeholders in the fight against tuberculosis will find this manual useful in the planning and implementation of their activities within the framework of the national policy.

Finally, the ministry of health wishes to convey special appreciation to all our partners and stakeholders for the technical and financial contributions towards the successful revision of this National Tuberculosis Control manual.

**Dr. Simon Zwane**

**Director of Health Services,**

**Ministry of Health**

# ACKNOWLEDGEMENT

The development of these guidelines was led by the Central Unit of the Swaziland National Tuberculosis Control Programme and technically supported by our partners. On behalf of the Directorate of Health Services, Ministry of Health, we acknowledge the immense technical contribution of the World Health Organization (WHO); US President’s Emergency Plan for AIDS Relief (PEPFAR) through the United States Centers for Disease Control and Prevention (CDC) and the United States Agency for International Development (USAID) Health Care improvement project managed by University Research Co., LLC; Baylor College of Medicine; and Médecin Sans Frontières (MSF) in the development of this guideline.

The efforts and unparalleled commitment demonstrated by the National TB Technical Working group and Guidelines review task team members towards accomplishment of this important task greatly appreciated. These individuals include: Dr Samson M. Haumba (URC) –Task team Chair; Hayk Karakozian (MSF) – Task team co-chair, Dr Kamal Mohammed (NTCP/TB Hospital), Philile Mdzebele (DOTS Focal point, NTCP), Ms Prudence Gwebu (NTCP, Pharmacist), Thabo Kunene (NTCP), Sandile Ginindza (NTCP, M&E and Research Officer), Ms Gugu Mchunu (NTCP, TB/HIV Focal point), Ms Nonhlahla Dlamini (NTCP, MDR-TB Nurse); Ms Siphiwe Ngwenya (NTCP), Derrick Khumalo (NCLS/TB Hospital), Dr Benjamin Mwangombe (MSF), Dr Natalia (MSF), Dr Yohannes Ghebreyesus (URC), Dr Piluca Pustero (Baylor College); Dr Kunene (RFM); Dr.Stephanie Marton (Baylor College) Dr.Lucia Gonazalez.(Baylor College); Mr Kees Keus (MSF) and Ms Gcinile Mavimbela ( STOP TB Partnership) who provided secretariat support.

We appreciate the inputs of our external peer reviewers to the final draft of the TB Manual who contributed to its improved quality. We especially thank Dr Peter Ehrenkranz (CDC-Swaziland) and Dr Francois Nywagi (URC-South Africa) for their invaluable inputs.

We specially thank Dr Kefas Samson (WHO-Swaziland) for the overall coordination of this guideline revision.

Finally, we are grateful to WHO for supporting the printing of this manual. We also acknowledge the commitment of the USAID health care improvement project managed by University Research Co., LLC to print the accompanying pocket guide to this manual.

**Dr. Steven V. Shongwe**

**Principal Secretary,**

**Ministry of Health****GLOSSARY AND ABBREVIATIONS**

**AIDS** Acquired Immune Deficiency Syndrome

**AFB** Acid Fast Bacilli

**BCG** Bacillus de Calmette et Guérin

**CSF** Cerebrospinal fluid

**DOT** Directly Observed Treatment

**DOTS** Directly Observed Treatment Short-course

DTBC Regional TB Coordinator

DTBO Regional TB Officer

**DTD** Demonstration and Training Regional

**E** Ethambutol

**EPTB** Extra-pulmonary tuberculosis

**FDC** Fixed Dose Combination

**HIV** Human Immunodeficiency Virus

**IEC** Information, Education and Communication

**INH** Isoniazid

**IUATLD** International Union Against Tuberculosis and Lung Disease

**MDR-TB** Multi-drug resistant tuberculosis

**MTB** Mycobacterium Tuberculosis

**NDSO** National Drug Services Organization

**NGO** Non-Governmental Organization

**NNRTI** Non-nucleoside Reverse Transcriptase Inhibitor

**NRTI** Nucleoside Reverse Transcriptase Inhibitor

**NTCP** National TB Control Programme

**PHC** Primary Heath Care

MOH Ministry of Health

**PI** Protease Inhibitor

**PPD** Purified Protein Derivative

**PTB** Pulmonary Tuberculosis

**PZA** Pyrazinamide

**RR**  Recording & Reporting

**R** Rifampicin

**S** Streptomycin

**SCC**  Short Course Chemotherapy

**SCR** Smear Conversion Rate

**TAT**  Turn Around Time

**VCT** Voluntary Counseling Test

**UNAIDS** United Nations Joint Programme on HIV/AIDS

**UNICEF** United Nations Children Fund

URC University Research Corporation

MSF Medicins Sans Frontieres

**WHO**  World Health Organization

**Z** Pyrazinamide

**ZN Stain** Ziehl – Neelsen Stain

EXECUTIVE SUMMARY

Tuberculosis remains a major public health problem in Swaziland. Currently the country has the highest TB incidence of 1287 per 100,000 population, and also have one of the highest HIV prevalence among incident TB cases (83%). With an HIV prevalence rate of 26% among the general population, the TB epidemic is undoubtedly being fuelled by the prevailing HIV epidemic. The National TB programme established in the 1980’s published the first edition of the National TB management guidelines in 2006.

The first edition facilitated the implementation of the DOTS strategy across the country specifically in standardization of laboratory diagnostic and treatment protocols for effective TB management. The increased drive by the HIV epidemic on the TB situation coupled with the emergence of MDR-TB changed the dynamics of TB control in the country, and hence the need for a revision of diagnostic and treatment policies. The second edition of the National TB Control Programme Manual seeks to address the common issues in the diagnosis and management of tuberculosis in the context of high HIV prevalence and taking cognizance of recent development in the area of new TB diagnostic tools as well as recent WHO recommended changes in treatment policies.

## Purpose of the guidelines

The principal purpose of these guidelines is to assist the various clinicians and other health care providers in both public and private sectors to optimize tuberculosis patient cure: curing patients will prevent death, relapse, acquired drug resistance, and the spread of TB in the commu­nity in a context-specific manner and in line with current WHO recommendations. Their further purpose is to guide the national TB programme staff in ensuring adequate coordination of TB control activities in the country.

## Target audience

The primary target audience for the guidelines is the staff of NTCP, Health care professionals and other TB service providers working in public and private health care facilities at the peripheral or referral levels. It is also recommended to students undertaking pre-service training in health care training institutions.

## Scope

These guidelines address the treatment of active TB disease in adults. It includes key topics such as TB diagnosis, laboratory services, treatment of TB, monitoring treatment and patient support, paediatric TB, introduction to MDR-TB, drug procurement and supply management and in­fection control.

## Justification for guideline update

The Stop TB Strategy’s emphasis on universal access for all persons with TB to high-quality, patient-centred treatment. However, highly in­fectious, smear-positive patients remain the primary focus for other aspects of TB control, including contact tracing and infection control. The Patients’ Charter for TB Care specifies that all TB patients have “the right to free and equitable access to TB care, from diagnosis through treatment completion”.

Secondly, WHO published the fourth edition of the guidelines for National TB programme that contained major recommendations for changes in treatment categories, and emphasis on early detection of MDR-TB. For example, the Categories I–IV, which were used to prioritize patients for treatment has been abandoned in the current WHO. Guideline, and instead, to adopt standard regi­mens recommended for each group according to the likelihood of their having drug resistance.

The current guidance also recommends integration of detection and treatment of both HIV infection and MDR-TB, and thus should contribute towards achievement of the Stop TB Strategy’s universal access to high-quality MDR-TB and HIV care.

With regard to HIV detection, this edition incorporates recent WHO recommenda­tions for provider-initiated HIV testing of all persons with diagnosed or presumptive TB.

## Highlights of the new revision

The guideline revision covered both clinical and some key programmatic areas of the National TB Control Programme operations. The current guideline has 17 chapters, the first two of which dealt with background information regarding the national context including a brief description of the health system (Chapter 1); as well as a brief description of the tuberculosis situation and the National programme organization, objectives and targets (Chapter 2).

Chapter 3 to 12 dealt with TB clinical management issues ranging from case detection, diagnosis, treatment, TB/HIV co-management to MDR-TB management and Infection Control. On the other hand, chapters 13 to 17 dealt with key programmatic issues including Supervision, monitoring and evaluation; logistics and supplies; human resource development for TB control as well as Advocacy, communication and Social mobilization (ACSM) and engagement of private care providers.

The key changes by chapter adopted based on the current WHO recommendations are as follows:

**Chapter 3. Case finding and registration:**

In this chapter, the issue of early TB case detection through rapid DST particularly the Xpert MTB/Rif (Gene Xpert) for all persons presumed to have TB regardless of HIV status.

A revision of the TB registration is included with a decision not to use severity of disease as criteria for case definition. Furthermore, the use of the “Diagnostic categories I–IV” in the first edition of the NTCP guideline has been discontinued. Instead, this second edition uses the same patient regis­tration groups used for recording and reporting, which differentiate new patients from those with prior treatment and specify reasons for retreatment.

The TB case definitions and well as registration groups have been revised to include cases diagnosed by the WHO-approved rapid diagnostic tests, which include molecular techniques

**Chapter 4. Diagnosis of TB:**

This chapter introduces the adoption of Xpert MTB/Rif as first initial diagnostic test for TB. It also elaborates on the optimization of the DST for rifampicin and Isoniazid using the LPA and the conventional DST using the MGIT. This edition also lays emphasis on using the results of rapid DST to inform treatment decisions.

The chapter also includes the introduction of two diagnostic algorithms based on availability of Xpert MTB/Rif in the local laboratory, while the other diagnostic pathway starting with microscopy and LPA for positive smear cases.

A section on the non-recommendation of the use of Inteferon Gamma Release Assay (IGRA) tests and commercial sero-diagnostic tests in Swaziland is also included in this chapter.

**Chapter 5. Laboratory services and QA**

This includes a description of the TB laboratory network and the recommended quality-assurance activities including external quality assessments (EQA), panel testing and blinded rechecking. The functions of the National TB Reference laboratory (NRL) in relation to the regional and peripheral levels have been elaborated as well as collaboration with the Supra-national reference laboratory (SNRL).

**Chapter 6. Treatment of tuberculosis:**

This chapter outlined the revised TB treatment regimens based on the new patient registration groups. In the treatment of new TB cases, 6 months rifampicin-containing regimen has been maintained. TB cases, and treatment initiation to be based on results of new rapid molecular-based tests i.e Gene Xpert and LPA.

For the purpose of treatment, previously treated patients are now to be defined by their likelihood of MDR-TB, and rec­ommendations for treatment regimen depend on reason for retreatment (failure, versus relapse and ). The chapter also provides for the possibility that, previously treated patients may have risk levels of MDR-TB that are high enough to warrant an MDR regimen while awaiting results of DST. Or they should be started on MDR-TB standardized treatment based on results of rapid DST (Gene Xpert or LPA).

**Chapter 7. Patient support and DOT provision**

The seventh chapter dealt with issues of treatment support provision for enhancing patient’s treatment adherence. This covers identification and training of treatment supporters as well as provison of enablers to TB patients. Nutritional support to TB control through the Food by prescription initiative as one of the main enablers has been elaborated in this chapter. The chapter concludes with measures to prevent and effectively manage treatment interruption.

**Chapter 8. Monitoring during treatment:**

In this chapter the performance of sputum smear microscopy at the completion of the inten­sive phase of treatment is reaffirmed with a recommendation to request for culture and DST if the smear result is positive. However, the extension of the intensive phase for patients who have a positive sputum smear at the end of the second month remains valid.

This chapter also includes a revised version of the symptom-based approach to side-effects of anti-TB drugs including a guide to anti-TB drug challenge and re-introduction after an adverse reaction.

Updated information on management of treatment interruption taking into cognizance the use of new diagnostic tests has also been incorporated

**Chapter 9: Management of Childhood TB:**

Update on paediatric TB treatment and HIV-TB co-management including contact investigation. The chapter provides various options to optimize bacteriological confirmation of TB in children through various specimen collection techniques like fine needle aspiration (FNA), gastric and nasopharyngeal aspirate. In addition, additional information provided with regard to management of extra-pulmonary TB based on the current WHO recommendations.

The chapter also includes a section on ensuring adequate follow up of TB treatment in Children and a guide on Cortimoxazole prophylaxis.

**Chapter 10. Co-management of HIV and active TB disease:**

This chapter addresses the current recommended approaches to TB/HIV co-management particularly with respect to early initiation of HIV positive TB patients on ART. The current WHO recommendations for antiretroviral therapy and timing of initia­tion are incorporated, while the Provider-initiated HIV testing for all patients with known or presumed TB has been reinforced. It also includes current WHO recommendations to start co-trimoxazole as soon as possible when a person living with HIV is diagnosed with TB.

**Chapter 11. Infection control:**

The national TB Programme has a detailed National TB Infection Control guidelines. This chapter therefore provided a synopsis of the infection control measures described in the national guidelines. For a detailed information on the infection control measures, reference should be made to the National TB Infection Control guidelines.

**Chapter 12. Management of drug-resistant TB:**

This chapter has been extensively revised to reflect recent WHO recommendations for the programmatic management of drug-resistant TB. The 2011 update on the programmatic management of DR-TB was used. It addresses MDR-TB management guidelines including the role of Xpert MTB/Rif, and dealing with MDR-TB in children. It covers regimen design for standardized MDR-TB regimen and dosages for both adults and Children. Also includes monitoring MDR-TB treatment as well as evaluation of treatment outcomes.

**Chapter 13. Supervision, monitoring and evaluation:**

This chapter defines supervision and distinguishes it from monitoring and evaluation. At the same time it highlights their interrelatedness. It includes recommendations on how to plan and execute an effective supervision of TB control activities.

**Chapter 14. Advocacy, Communication and Social Mobilization (ACSM):**

The Chapter elaborates on how to effectively optimize ACSM for TB with the view to empower patients and communities using a target-specific approach. Also gives guidance on how to use ACSM to influencing the political agenda in favour of TB control, as well as developing and utilizing an effective approach to mass media advocacy.

Finally, the guideline also includes a list of annexes outlining key procedures, standard operating procedures on Human Resource development and Supplies and logistics management and protocols as well as essential recording and reporting forms of the National TB Control Programme.

1. INTRODUCTION

## The country

Swaziland is a small landlocked country situated between South Africa and Mozambique covering an area of 17,364km^2^. The country has a predominantly (77%) rural population of about 1.1 million (2006 Census) people.

Swaziland enjoys a tropical to a near-temperate climate along the western highlands, which rises to an altitude of over 1,800 metres above the sea level, while the low-veld areas are generally hot. The country lies in a summer rainfall region. The majority of the population consists of ethnic Swazis.

With a GNI per capita income of USD 2,280, Swaziland is classified by the World Bank as a low-middle income country and therefore placed in the IBRD lending category. However, the real GDP growth rate has fallen in recent years to 2.8% in 2007. However, despite this relative high per capita income, income distribution is markedly uneven (GINI index 51% according to PRSAP 2006). Life expectancy currently stands at 32 years, being 56 years in 1986, and 69% of the population living below the upper poverty line of USD 7.2 /capita/month. About 66% of the population lives below the poverty line (Swaziland Human Development Report, 2000) with high rural-urban disparities in access to basic services. For example, whilst 91% of the urban population has access to safe water, it is only 37% for the rural population. The per capita expenditure on health for the urban population is 3 times that for the rural population.

## The health system

The health care delivery system consists of both formal and informal sectors. The formal sector consists of both public and private service providers including NGOs, mission, industry health services and private practitioners, while the informal sector consists mainly of traditional and other complementary or alternative health care providers. An estimated 45% of health facilities are operated by the public sector, 23% by the private-for-profit sector, while the remaining 32% are operated by private not-for-profit sector including Faith based organizations (FBOs), Non-governmental organizations (NGOs) etc (see tables below).

The service delivery system itself is loosely organized into a three-tier system including (i) three national (referral) and five regional hospitals (total some 1800 beds); (ii) Primary Health Care services, being composed of Health Centres (HCs)^[[1]](#footnote-1)^, Rural Clinics^[[2]](#footnote-2)^ and a network of outreach sites and (iii) Community Based Care, where Rural Health Motivators (RHM), Traditional Birth Attendants (TBAs), Home Based Care (HBC) volunteers and traditional practitioners provide care, support and treatment.

Majority of the Swazi population (85%) live within a range of 8 km for a health facility. However, this does not translate to equitable access in view of internal variability in the distances to facilities, transportation accessibility and the range and quality of care provided by facilities. Furthermore, this still falls short of the 5 km benchmark recommended by the WHO and about 20% of the population have limited or no access to health service.

**Table 1.1: Distribution of hospitals, Health Centres and Clinics by region**

| **REGIONS** | **POPULATION** | **GENERAL HOSPITAL** | **HEALTH CENTRES** | **CLINICS**  **Type A/B** | **TOTAL NO OF HF** | **HF/**  **100.000** |
| --- | --- | --- | --- | --- | --- | --- |
| HHOHHO | 282.734 | 2 | 2 | 62 | 64 | 22.6 |
| LUBOMBO | 207.731 | 1 | 2 | 43 | 45 | 21.7 |
| MANZINI | 319.530 | 2 | 0 | 80 | 80 | 25.0 |
| SHISELWENI | 208.454 | 1 | 1 | 33 | 34 | 16.3 |
| **TOTAL** | **1.018.449** | **6** | **5** | **218** | **223** | **21.9** |

*Source: National Health Sector Strategic Plan, Ministry of Health Swaziland*

HF = Health Facility, being HCs + Clinics

Note: Public Health Units (7) exist in most HCs and in all Hospitals

**Table 1.2: Ownership of health facilities by region**

| **REGIONS** | **Public Sector**  **(Clinic + HC)** | **Industry** | **Facilities** | **NGO** | **Private**  **(Doctor)** | **Private**  **(Nurse)** | **TOTALS** |
| --- | --- | --- | --- | --- | --- | --- | --- |
| HHOHHO | 24+2 | 4 | 11 | 4 | 19 | 0 | 64 |
| LUBOMBO | **21+2** | 8 | 7 | 0 | 4 | 3 | 45 |
| MANZINI | 28+0 | 15 | 12 | 4 | 18 | 3 | 80 |
| SHISELWENI | **22+1** | 1 | 3 | 4 | 3 | 0 | 34 |
| **TOTAL** | **95+5** | **28** | **33** | **12** | **44** | **6** | **223** |
| Percentages % | 45% | 12% | 15% | 5% | 20% | 3% | 100% |

*Source: National Health Sector Strategic Plan, Ministry of Health Swaziland*

HF = Health Facility, being HCs + Clinics

Note: Public Health Units (7) exist in most HCs and in all Hospitals

From a functional perspective, 172 HF provide ANC (77%); 137 provide PMTCT (61%); 70 HF provide ART (31%) and 170 provide AIDS testing and counselling (70%). SAM identified 204 doctors (40% Swazi; 20/100,000) and 1778 Nurses (90/100,000).

The health service indicators are summarized in table below:

Table 1.3: Summary of Health Service indicators

| **Indicator** | **Hhohho** | **Lubombo** | **Manzini** | **Shiselweni** | **Total** |
| --- | --- | --- | --- | --- | --- |
| Population | 331,734 | 249,153 | 360,228 | 241,365 | 1,182,480 |
| # Health facilities | 40 | 35 | 52 | 27 | 154 |
| Health facilities per 100,000 pop | 12 | 14 | 14 | 11 | 13 |
| Facilities with in-patient beds | 9 | 9 | 14 | 4 | 36 |
| # in-patient beds | 363 | 302 | 813 | 257 | 1755 |
| In-patient beds per 100,000 pop | 115 | 121 | 226 | 106 | 148 |
| Doctors per 100,000 pop | 17 | 6 | 10 | 5 | 10 |
| Nurses per 100,000 pop | 70 | 52 | 57 | 41 | 56 |
| Midwives per 100,000 pop | 72 | 47 | 80 | 46 | 64 |
| Estimated population growth rate |  |  |  |  |  |

*Source: MOH Service Availability Mapping 2007*

At the community level, a network of community health workers exists, which consists of Rural Health Motivators to promote community participation in health activities at that level. Health committees are also in place to assist in the general management of health care facilities.

It is estimated that 85% of the population in the country currently lives within an 8km radius of a health facility, while nationally about 20% of the population have limited or no access to a health facility with the rural poor worst affected.

Despite a comparatively reasonable physical access to health services in comparison, quality of health care remains a challenge owing to the high disease burden, chronic shortage of human resources for health, deteriorating infrastructure, inadequate budgetary allocation and weak support supervision systems.

1. THE NATIONAL TUBERCULOSIS CONTROL PROGRAMME

## Global Epidemiology of TB

According to the WHO TB report of 2011, it is estimated that the global burden of disease caused by TB in 2010 are as follows: 8.8 million incident cases, 12 million prevalent cases, 1.1 million deaths among HIV-negative people and 0.35 million deaths among HIV-positive people. Most cases were in Asia (59%) and Africa (26%). An estimated 12–14% of incident cases were HIV-positive; the African Region accounted for approximately 82% of these cases.

Globally, there were 5.7 million notified cases of TB in 2010, equivalent to a case detection rate (CDR, defined as the proportion of incident cases that were notified) of 65% (range, 63–68%). India and China accounted for 40% of the world’s notified cases. At global level, the treatment success rate among new cases of smear positive pulmonary TB was 87% in 2009.

There were an estimated 450 000 prevalent cases of multi-drug resistant TB (MDR-TB) in 2010.

Less than 2% of new cases and 6% of retreatment cases were tested for MDR-TB globally. The four countries that had the largest number of notified cases of MDR-TB in 2010 were China (63 000), India (64 000), the Russian Federation (31 000), and South Africa (9 100). By July 2010, 58 countries and territories had reported at least one case of extensively drug-resistant TB (XDR-TB).

## The Tuberculosis situation in Swaziland

Tuberculosis poses a big public health challenge to the government and the people of the kingdom of Swaziland and constitutes an estimated 20% of institutional deaths. With an estimated TB incidence of 1,287 per every 100,000 of its population, Swaziland is one of the countries of world with the highest incidence of TB. Compared to a 1990 level of 267 new cases (all forms) per 100,000 population per year, TB incidence had increased six-fold, by 2010. and The incidence of the infectious cases (sputum smear positive pulmonary TB cases) tripled in the same period. In terms of prevalence of disease, there were 840 TB cases (all forms) per 100,000 in the country in 2010, which translates to about 9,600 prevalent TB cases at any point in time. Similarly, mortality from TB increased from 76 per 100,000 in 1990 to 304 per 100,000 in 2008. This mortality figure translates to about 2,780 deaths annually due to TB alone; and an estimated 17,000 TB-related deaths by 2015 if drastic actions are not taken. Most deaths were associated to HIV co-infection.

In 2010, the International Classification of Diseases-10 (ICD-10) re-defined mortality to TB by excluding all cases of death due to HIV. This revision subsequently re-adjusted mortality rate due to TB to 32 per every 100,000 cases among HIV negative cases. The prevalence of HIV among newly diagnosed tuberculosis patients was 82% in 2010 according to the national tuberculosis control routine surveillance and has been rising over the last decade. In the general public, however, the HIV prevalence among the 15-49 years age group is estimated to be 26%. Moreover, a country-wide research on drug resistance among TB patients conducted in 2009 showed 7.7% of MDR among new cases of TB, as well as 33.9% among previously treatment cases of TB, which are incomparably high relative to other countries. Cumulatively 503 MDR-TB cases have been reported from 2006 to 2009.

The NTCP overall notified 852 TB cases per 100,000 in 2010 up from 650 per 100 000 in 2006. Case detection rate of new smear positive cases was 69%, in 2010, having increased from 34% in 2006. Of all detected cases in 2009, 68% successfully completed treatment in up from 42% in 2006.

## The National TB Control Programme

The NTP was established in 1986, and has been fully funded by the Government of Swaziland since 2001. At the national level, it operates under the Directorate of Health of Health Services (DHS) in the Ministry of Health, alongside other communicable and non-communicable disease control programs.

The program is headed by an NTP Manager under the responsibility of the Deputy Director Public Health (DDHS), who in turn reports to the Director of Health Services, who reports to the Principal Secretary in the ministry.

At the regional level, the Regional TB Coordinators directly supervises the TB focal points who are responsible for TB case registration and treatment in health facilities. At community level the community DOT supporter reports to the TB regional coordinators.

At community level, various stakeholders including CHWs, CSOs, NGOs, CBOs, TB Treatment Supporters and Traditional Healers etc in their varying capacities deliver TB care in close collaboration with the Regional Health Management Teams.

## The TB Control Strategy and framework for Swaziland

Swaziland adopted the Stop TB Strategy as the framework for TB Control in the country with emphasis on the pursuance of high quality DOTS expansion and enhancement, TB/HIV and MDR-TB. This is based on the Stop TB Strategy.

### Central Unit

The main roles of the National TB Programme at central level includes the following:

1. develop policies and plans, and secure budgets for the NTP
2. Coordinate the NTP, including all governmental and non-governmental organizations working in TB control
3. Plan for anti-TB drugs and supplies for patient management;
4. Prepare training programmes for health workers involved in the NTP
5. Monitor, procure and distribute supplies for the NTP (drugs, equipment, documentation, health education materials)
6. Prepare and develop reporting standards
7. Coordinate national TB surveillance activities
8. Supervise NTP activities at the Regional level
9. Conduct research for promotion of the NTP
10. Plan for TB laboratory equipment and reagents;

In order to effectively perform these roles, the central level is supported technically by WHO and other technical partners.

### Regional Level

The RHMTs are responsible health care planning, supervising health facility facilities (clinics and health centres) and the implementation of TB control activities through the regional TB coordinators. The regional TB control activities are essentially part of the regional health plan, which is coordinated through the Regional Health Management Teams (RHMT) with the regional TB Coordinators as focal points. However, the Central Unit of the National TB Programme provides technical support and capacity building to that level. The Regional coordinators are therefore responsible for planning and budgeting for specific TB Control activities including training of different cadres on TB. The central level as well as regional medical officers provide support in the facilitation of such trainings.

### Basic TB Management Units (BMUs)

Currently, there are currently 66 TB BMUs consisting of regional government hospitals, health centres, mission hospitals, industry health services and private practices across the country, 20 of which have on-site laboratories. About 140 clinics provide only continuation phase treatment. Each of the diagnostic sites has a focal point referred to as the TB nurses who sees the majority of persons with presumptive TB and clients, requests for direct sputum microscopy, diagnose smear positive cases, and refer others as appropriate for confirmation of diagnosis by a medical doctor. A DOTS adherence officer, stationed in each diagnostic site and equipped with a motorbike is responsible for following up patients who interrupt treatment, and where community-based TB care has been supervising community DOT supporters.

### The National TB Hospital

The national tuberculosis hospital is responsible for specialized management of tuberculosis that includes drug resistant tuberculosis. It is equipped with highly skilled professionals and will be responsible for implementing the clinical components of the drug resistant tuberculosis guidelines. The admission criteria to the TB hospital are as follows:

Upon diagnosis, all confirmed MDR cases that are very sick, clinically unstable or have unfavourable home infection control assessment could be hospitalized for up to 4 weeks, and may be prolonged for up to 6 months if clinically indicated.

The referring health facility should inform the outpatient department of the TB hospital before transporting the patients for admission. Transport for clients referred to the TB hospital shall be arranged by the national TB referral hospital.

The main indications for hospitalization include the following:

- Initiation of treatment
- Adherence problems
- patient very sick (clinically and physically unfit)
- Severe adverse effects
- Immobility
- Vulnerable patients e.g. disadvantaged-orphan, mentally , socially or physically handicapped

***All confirmed XDR-TB patients will be admitted and managed in isolation until sputum culture conversion.***

In the event of refusal of treatment by an XDR-TB patient, the NTCP or Directorate of Health Services at the ministry of health headquarters, or the Social Welfare Department under the Deputy Prime Minister’s Office (DPM) should be notified immediately. The following measures should also be tried:

- Counsel the patient on the benefit of the treatment;
- Involve relatives
- Ensure that the patient or relative signs the ***‘Refusal of treatment form’***
- Notify NTCP or Directorate of Health Services of the Ministry of Health Headquarters.
- Notify the Social welfare Department of the DPM’s Office

### Intra-ministerial collaboration

The NTP and the National AIDS Programme (SNAP) are housed in separate locations at national level, hindering close collaboration in planning and implementation of collaborative programme activities. Both programmes should share their comparative advantages to their mutual benefit. E.g., the NTP’s experience in directly observed treatment, recording and reporting can be valuable to the SNAP in implementation and effective monitoring of the ART programme. The latter in turn can assist the NTP with its wealth of experience in advocacy, communication, education and multi-sectoral collaboration.

Since currently about 80% of TB patients are also co-infected with HIV, the NTP can significantly contribute to increasing access of ART by ensuring screening of all persons with presumptive TB and patients in all TB sites. Similarly, mainstreaming of TB activities in HIV/AIDS planning and management is essential, as is providing HIV/AIDS prevention, care and support activities to people with TB disease. To this end, the NTP should closely collaborate with NGOs, CBOs, FBOs and other organisations that provide home-based care services, which will in turn contribute to expanding community-based TB care. The national TB programme also has a functional collaboration with the National Malaria Control Programme (NMCP)

### NTP Partners

The national TB Programme collaborates with technical and financial partners within and outside the country. The technical partners include the World Health Organization, the University Research Corporation (URC), Italian Cooperation, Medicens sans Frontieres (MSF) and the Royal Netherlands Tuberculosis Foundation (KNCV). The NTP has numerous implementation partners who recently transformed into an umbrella body referred to as the Swaziland Stop TB Partnership. The members of the Swaziland Stop TB partnership include Private practitioners, Non-governmental organizations (NGOs), and Community-based organizations; while the MOH, NERCHA and WHO serve as ex-officio partners.

### NTP funding

The national TB Programme is funded mainly by the Swaziland Government with additional funds from the Global Fund (GFATM). The programme has an established budget line that covers human resources, first and second line anti-TB drugs and administrative costs. The WHO, PEPFAR/URC, MSF, KNCV, FIND and MSH provide resources for closing funding gaps in the implementation of programme activities.

## Goals, Objectives and targets of the National TB Programme

The Goal of the Swaziland National TB Programme is to reduce TB mortality, morbidity and disease transmission to a level that it no longer constitutes a Public Health Problem, while preventing the development of drug resistance.

### The Objectives:

To provide standardized short-course chemotherapy provided under strict supervision to at least all identified sputum smear positive cases.

### The targets:

Swaziland subscribes to the global initiative to eliminate TB as a public health problem within the context of the following agreed targets:

- **The World Health Assembly (WHA)targets** to cure at least 85% of newly detected cases of sputum smear-positive TB and to detect 70% of the estimated incidence of sputum smear-positive TB;
- **Millennium Development Goal (MDG) targets:**Target 8 under MDG Goal 6 (tocombat HIV/AIDS, malaria and other diseases), To have halted by 2015 and begun to reverse the incidence of malaria and other major diseases, with the following specific for TB:
  - **Indicator 23:** Prevalence and death rates associated with tuberculosis
  - **Indicator 24:** Proportion of tuberculosis cases detected and cured under DOTS
- **The Stop TB Partnership Targets:**
  - **By 2005:** At least 70% of people with infectious TB will be diagnosed (under the DOTS strategy), and at least 85% of these patients will be cured.
  - **By 2015:** The global burden of TB (disease prevalence and deaths) will be reduced by 50% relative to 1990 levels.
  - **By 2050:** Elimination of TB as a Public Health Problem, defined as a prevalence level below 1 case per million population.

### Programme Goal

To reduce morbidity, mortality, disease transmission and socio-economic burden of TB including the TB/HIV co-infection while minimizing the risk of drug resistance to such an extent that the disease no longer a public health problem to the Swaziland nation.

### Objectives

The National TB Programme shall within the framework of the global Stop TB Strategy aim at achieving the following strategic results by 2015:

- Achieve universal access to high-quality care for all people with TB
- Reduce the human suffering and socioeconomic burden associated with TB
- Protect vulnerable populations from TB, TB/HIV and drug-resistant TB
- Protect and promote human rights in TB prevention, care and control

### Swaziland Stop TB Partnership targets

- Treatment success (cure + completion) rate increased from 68% to 85% for patients with smear-positive tuberculosis by 2015;
- Case detection of patients with smear-positive tuberculosis increased from 68% to 80% by 2014;
- HIV testing of 100% of TB patients;
- enrolment of 100% of HIV-positive TB patients on co-trimoxazole preventive therapy (CPT) and antiretroviral therapy (ART);
- provision of isoniazid preventive therapy (IPT) to all people living with HIV who are attending HIV care services and are considered eligible for IPT;
- testing of 100% of previously treated TB patients for MDR-TB, as well as testing of any new TB patients considered at high risk of having MDR-TB (estimated globally at around 20% of all new TB patients);
- enrolment of all patients with a confirmed diagnosis of MDR-TB on treatment consistent with international guidelines;
- Adequate capacity for operational research and epidemiological surveillance created for programme management by 2012;
- Improve the level of knowledge on tuberculosis disease and services for improved health-seeking behaviour and treatment adherence by 2014.

1. TB CASE FINDING AND REGISTRATION

## Importance of TB case finding and registration

The emphasis of the NTCP is on early TB case detection and provision of timely effective treatment. The NTCP is also responsible for ensuring that identified persons with presumptive TB are properly diagnosed, meet the definition for case or definite case, and are treated appropriately, and that outcomes are evaluated at the end of treatment.

## Identification of persons with presumptive pulmonary TB:

Every person presenting to a health facility with symptoms suggestive of tuberculosis should be considered a “person with presumptive tuberculosis”. The most common symptoms suggestive of pulmonary tuberculosis are:

- Cough of any duration
- Fever
- Night sweats
- Weight loss
- shortness of breath, and chest pain
- loss of appetite
- sputum production which may be blood-stained
- a general feeling of illness (malaise)
- tiredness and loss of motivation

On clinical examination, there may be general signs, such as fever, tachycardia (fast pulse rate) and finger clubbing. Chest signs (heard through a stethoscope) may include crackles, wheezes, and bronchial breathing.

A patient presenting with these symptoms and signs who is, or was in contact with a person with infectious tuberculosis should be considered as a presumptive PTB case.

All persons with presumptive TB who present to health facilities should be recorded on a ***“Register of presumptive TB cases”.***

All health facilities without on-site microscopy services should sent sputum samples collected for TB laboratory investigation (smear microscopy or Gene Xpert) to the nearest accredited laboratory within the Regional.

### Sputum Collection, Labeling, Storage and Transport

At least two sputum specimens should be taken from a person with presumptive TB.

**Option A:** At the first encounter with the patient the first specimen is collected on the spot referred to as the**“ first spot specimen”** is collected and the patient should be provided with a sputum container for collection of the second Spot sample at least one hour apart (**Second Spot Specimen**)

**Options B:** At the first encounter with the patient the first specimen is collected on the spot referred to as the **“spot specimen”** is collected; the patient should be provided with a sputum container for collection of the second sample early morning at home **(‘early morning specimen’)**.

For Gene Xpert test, it is important to ensure collection of **a minimum of** **2 mls of good quality sputum** to ensure good results.

#### Sputum collection procedure

- Collection of sputum samples should be performed outside in an open place;
- The person should rinse the mouth with water;
- Explain the steps fully and slowly
- Demonstrate a deep cough from the bottom of the chest, beginning with deep breathing
- Ask the patient to be very careful to direct the sputum into the container not to contaminate the outside of the bottle
- Supervise the collection, but do not stand in front of the patient
- Do it in a well-ventilated area or outside without others watching
- Give the patient the container without the lid
- Hold the lid yourself, ready to replace it immediately
- Make sure that the lid is securely closed
- Wash hands after handling the sputum specimen
- The person must be encouraged to produce a specimen after deep coughing even if this is saliva.

#### Sputum labeling

Correct labeling is essential and will save time and prevent errors. Where possible, a bar code reader should be used, otherwise a permanent marker should be used to label the sputum container.

Label the container first, very clearly indicating:

- Name of clinic/hospital
- Name of patient and clinic/hospital number
- Indicate whether the specimen is pretreatment, follow-up or end of treatment specimen
- Write clear instructions regarding what investigations are required
- Write the appearance of the sputum (e.g. mucoid, lumpy, green, offensive, etc)
- Date the specimen clearly and time of collection of the specimen

**Note: Labeling should always be done on the body of the container as the lids may easily be mixed up during specimen processing.**

#### Sputum storage

- Place the sputum bottle in a plastic bag if possible to prevent contamination
- Store sputum specimen in a fridge if transport is not available immediately. Do not store in a freezer
- Sputum specimens should not be kept in a fridge for more than a week before transportation, if possible send away as soon as possible
- Record the date on which the specimen has been sent to the laboratory in the “Register of presumptive TB cases”

#### Transportation of sputum specimens

- For rural health facilities that are without laboratory services, sputum specimens have to be transported to laboratories at least on a weekly basis;
- The National Sample Transportation should organize a sputum collection schedule for all facilities in the Regional;
- Transportation of specimens to the laboratory should be in cool sputum transport boxes. High temperatures during transit will kill bacilli;
- During transportation, specimens should be protected from contact with direct sunlight;
- The driver should be properly informed of the reasons for transporting the specimens, thereby ensuring that specimens go direct to the laboratory.

**Note: Every working day, a responsible person should check the Register of presumptive TB cases to confirm which results are pending and then contact the laboratory to find out where the results are.**

## Identification of persons with presumtive Extra-Pulmonary TB:

Symptoms of extra-pulmonary tuberculosis depend on the organ involved. Chest pain from tuberculosis pleurisy, enlarged lymph nodes and sharp angular deformity of the spine are the most frequent signs of extra-pulmonary tuberculosis.

Extrapulmonary tuberculosis is more strongly associated HIV infection than pulmonary tuberculosis. HIV-related extrapulmonary tuberculosis is a WHO clinical stage 4 (advanced AIDS) diagnosis, and patients often have disseminated disease and are at high risk of rapid clinical deterioration and death.

### Common types of Extra-pulmonary TB

The most common types of extra-pulmonary tuberculosis are:

- TB meningitis
- TB lymphadenitis
- Miliary tuberculosis
- TB Pleural effusion
- Tuberculous empyema
- Tuberculous pericardial effusion
- Ascites
- TB of the bones

Table 3.1: the usual clinical features and diagnostic tests of other forms of extrapulmonary TB

| **Site of disease** | **Clinical features** | **Recommended investigation** |
| --- | --- | --- |
| **Spine** | Back pain  Gibbus  Psoas abscess  Radicular pain  Spinal cord compression | Plain X-ray  Tissue biopsy |
| **Bone** | Chronic osteomyelitis | Tissue biopsy |
| **Peripheral joints** | Usually monoarthritis especially hip or knee | Plain X-ray  Synovial biopsy |
| **Gastrointestinal** | Abdominal mass  Diarrhoea | Barium X-ray |
| **Liver** | Right upper quadrant pain and mass | Ultrasound and biopsy |
| **Renal and urinary tract** | Urinary frequency  Dysuria  Haematuria  Loin pain/swelling | Sterile pyuria  Urine culture  Intravenous pyelogram  Ultrasound |
| **Adrenal gland** | Features of hypoadrenalism (calcification)  (hypotension, low serum sodium, normal/high potassium,  raised urea, low glucose) | Plain X-ray  (calcification)  Ultrasound |
| **Upper respiratory tract** | Hoarseness and stridor  Pain in ear  Pain on swallowing | Usually complication  of pulmonary disease |
| **Female genital tract** | Infertility  Pelvic inflammatory disease  Ectopic pregnancy | Pelvic examination  X-ray genital tract  Ultrasound pelvis  Tissue biopsy |
| **Male genital tract** | Epididymitis | Often evidence of  renal/urinary tract TB |

*Source: TB/HIV a Clinical Manual. WHO/HTM/TB/2004.329*

## Diagnosis and management of TB in Children:

Children can present with TB at any age but most commonly under 5 years or during the adolescent stage.

For the full details of TB diagnosis and management, refer to Chapter 10 of this guideline.

## Defining a case of tuberculosis

To ensure proper registration and notifications of detected TB cases, it is important to use the standard NTCP TB case definitions.

### The importance of case definition

The standardized TB case definitions are important for the following reasons:

- proper patient registration and case notification;
- selecting appropriate standard treatment regimens (see Chapter 3);
- standardizing the process of data collection for TB control;
- evaluating the proportion of cases according to site, bacteriology and treat­ment history;
- cohort analysis of treatment outcomes;
- accurate monitoring of trends and evaluation of the effectiveness of TB pro­grammes within and across districts, countries and global regions.

### The TB case definitions

The case definitions which have been adapted from the WHO guidelines is based on the level of certainty of the diagnosis and on whether or not laboratory confirmation is available.

**Persons with presumtive tuberculosis:** Any person who presents with symptoms or signs sugges­tive of TB. The most common symptom of pulmonary TB is cough, which could be of any duration with any accompanying symptoms (fever, weight loss, night sweats, chest pain, malaise etc); or cough of 2 or more weeks duration even without other symptoms.

**Bacteriologically confirmed tuberculosis**. A patient with *Mycobacterium tuberculosis* complex identified from a clinical specimen, either by culture or by a WHO-approved new diagnostic (WRDs) methods (e.g the Xpert MTB/Rif, or molecular line probe assay). A definite case can also be defined as a pulmonary case with one or more initial spu­tum smear examinations positive for acid-fast bacilli (AFB).

**Clinically diagnosed tuberculosis**. A patient in with no bacteriological confirmation of *M. tuberculosis* established, in whom a medical officer has made the diagnosis of TB, and has decided to treat the patient with a full course of TB treatment.

*Note*. Any person initiated on TB treatment on clinical grounds should be recorded as a clinically diagnosed case. Incomplete “trial” TB treatment should not be given as a method for diagnosis.

## TB patients registration groups

The diagnosis of TB refers to the recognition of an active case. Beyond the diagnosis of TB disease, the type of TB case should also be defined to allow appropriate treatment to be given and the outcome of treatment evaluated. Before initiating treatment, health care providers should register TB cases into the various registration groups based on the following determinants:

- Site of TB disease
- Bacteriology (result of sputum smear) including the WRDs
- History of previous treatment of TB

### Site of TB disease

**Pulmonary tuberculosis (PTB)** refers to disease involving the lung parenchyma. A patient with both pulmonary and extra-pulmonary TB should be classified as a case of pulmonary TB. Miliary TB is classified as pulmonary TB because there are lesions in the lungs.

**Extra-pulmonary tuberculosis (EPTB)** refers to tuberculosis of organs other than the lungs, e.g. pleura, lymph nodes, abdomen, genitourinary tract, skin, joints and bones, meninges. Diagnosis should be based on one culture-positive specimen, or histological or strong evidence consistent with active EPTB, followed by a decision by a clinician to treat with a full course of tuberculosis chemotherapy.

### Bacteriology (result of sputum smear) in pulmonary TB

1. **Bacteriologically confirmed tuberculosis:** Consisting of:

- Culture-positive
- Sputum-positive
- WHO-Approved Rapid Diagnostic (WRD) test positive

1. **Clinically diagnosed tuberculosis:** Consisting of:

- Any put on TB treatment without bacteriological confirmation
  - Smear negative
  - Smear not done
  - Extra-pulmonary TB

1. **Retreatments or previously treated tuberculosis**

- Relapse
- After treatment failure
- After lost to follow-up

### History of previous treatment

Based on history of previous treatment with anti-TB drugs, detected TB patients can be assigned to two main registration groups namely:

- New cases
- Previously treated cases.

#### New patients:

These are patients that have never had treatment for TB, or have taken anti-TB drugs for less than 1 month. New patients may have positive or negative bacteriology and may have disease at any anatomical site.

**Previously treated cases:**

These are patients that have received 1 month or more of anti-TB drugs in the past, may have positive or negative bacteriology and may have disease at any ana­tomical site. Previously treated cases should be further classified by the outcome of their most recent course of treatment as follows:

Table 3.2: the usual clinical features and diagnostic tests of other forms of extrapulmonary TB

| \| **Registration group**  **(any site of disease)** \| \| --- \| | | **Bacteriology: Smear** | **Bacteriology: Xpert** | **Outcome of most recent prior treatment** |
| --- | --- | --- | --- | --- | --- |
| **New** | | + or – | + or - | - |
| **Previously treated** | Relapse | + | + | Cured |
|  | Failure | + | + | Treatment failed |
|  | Treatment interrupted | + | + | Treatment interrupted |
| **Transfer in:** A patient who has been transferred from another TB register to continue treatment | | + or – | + | Still on treatment |
| **Other** |  | + or – | + or _- | All cases that do not fit the above definitions, such as patients   - for whom it is not known whether they have been previously treated; - who were previously treated but with unknown outcome of that previous treatment; and/or - who have returned to treatment with smear-negative PTB or bacteriologically negative EPTB |

*Source: Adapted from Treatment of tuberculosis guidelines. 4^th^Edition. WHO/HTM/TB/2009.420*

1. DIAGNOSIS OF TB

## Approach to TB diagnosis

Detection of TB in health facilities should be an ongoing activity. Diagnosis of TB starts with identifying persons with presumptive TB through clinical symptoms and physical examination.

Diagnosis of tuberculosis should include assessment for drug resistance to ensure timely initiation on the most appropriate treatment regimen.

Sputum samples need to be collected for laboratory investigation.

The initial diagnostic tests for all persons with presumptive tuberculosis should include

1. a microscopy preferably using the iLED; or,
2. an Xpert MTB/Rif test.

The subsequent diagnostic tests which will include Line Probe Assay, Culture and DST to further confirm TB, MDR-TB diagnosis shall be applied based on the diagnostic algorithm (See figure ???).

## Bacteriological confirmation of pulmonary TB diagnosis

###

### Microscopy

Microscopy remains an important screening test for persons with presumptive pulmonary TB which aims at demonstration of AFB in sputum.For microscopy to be reliable, consistent quality-assurance is required. The number of bacilli (AFB) seen in a smear reflects the patients infectivity (see 6.6.1). Two sputum samples are required for microscopy.

Two types of microscopy methods are available in Swaziland, namely:

- the direct smear light microscopy using the conventional Ziehl Nelsen’s staining technique; and
- the fluorescent-LED method where the AFB are visualised as fluorescence in a dark background.

The fluorescent LED microscopy has an advantage over the light microscopy in terms of ability to detect the bacilli easier and faster reducing time to diagnosis.

**The main uses of the microscopy are:**

- Initial diagnosis of pulmonary TB for TB suspects who do not have access to the Gene Xpert.
- Follow up the smear status of patients during TB treatment.

Results for microscopy are given quantitatively according to the number of bacilli seen on each smear:

- A positive result is defined as one showing actual number to 3+;
- A positive result in one of the two samples submitted is considered a bacteriologically confirmed case of pulmonary TB;
- All positive sputum results should be recorded in both the laboratory and TB registers in red ink for ease of identification.
- The laboratory iden­tification number and the date the examination was performed should be entered in the column next to that for the result of the examination.

Table 4.1: Guide for grading results of smear microscopy

| **Number of bacilli seen on a smear** | **Fields to examine** | **Results reported** |
| --- | --- | --- |
| No AFB per 100 oil immersion fields | 100 | Neg |
| 1-9 AFB per 100 oil immersion fields | 100 | Indicate actual number (1-9) |
| 10-99 AFB per 100 oil immersion fields | 100 | 1+ |
| 1-10 AFB per 1 oil immersion field | 50 | 2+ |
| >10 AFB per 1 oil immersion field | 20 | 3+ |

*Source: Laboratory services in tuberculosis control. WHO/HTM/TB/98.258*

All positive sputum results should recorded in both the Laboratory and TB Regional registers in **red** ink for ease of identification. The laboratory iden­tification number and the date the examination was performed should be entered in the column next to that for the result of the examination.

### Xpert MTB/Rif (Gene Xpert) test

This is an automated cartridge-based rapid molecular test for mycobacterium tuberculosis as well as detection of rifampicin resistance-conferring mutations directly from sputum providing both results within 2 hours.

The test which was endorsed by WHO in 2010 as an initial diagnostic test for pulmonary tuberculosis has about 40% sensitivity gain over light microscopy, and is useful in diagnosing TB in PLHIV who often have smear negative microscopy tests. A typical 4-module Gene Xpert can perform about 16 tests per a working day.

#### Use of Xpert MTB/Rif in new TB cases

Given the high HIV prevalence among incident TB cases in Swaziland, Gene Xpert should be performed as an initial diagnostic test for all persons with presumptive tuberculosis where the equipment is available or access to an off-site laboratory with the equipment is possible through efficient sample transportation.

- If the result is ***positive for MTB but negative for rifampicin resistance,*** the patient should be started on New TB case treatment regimen;
- If the result is ***positive for MTB and also positive for rifampicin resistance,*** the patient should registered as ***Rifampicin Resistant tuberculosis (RR-TB)*** in the DR-TB register.
- Full DST should be requested for all patients diagnosed and started on treated as RR-TB cases;
- Reference should be made to the algorithm on pages 32-33 for further management decisions.

#### Use of Xpert MTB/Rif in previously treated TB cases

Gene Xpert test should be performed for all previously treated TB cases who fail to convert at the end of three months intensive phase, or having positive smear at 5^th^ month of treatment where the equipment is available or access to an off-site laboratory with the equipment is possible through efficient sample transportation.

- If the result is ***positive for rifampicin resistance***, the case should be registered as ***Rifampicin Resistant tuberculosis (RR-TB)*** in the DR-TB register.
- If the result is positive for ***MTB but negative for rifampicin resistance***, the patient should be continued on the treatment for Previously treated cases;
- Full DST should be requested and treatment adjusted when the results are available.
- Reference should be made to the algorithm on page 32-33 for further management decisions.

The detection of rifampicin resistance using the Xpert MTB/Rif test is considered sufficient to start the patient on the standardized MDR-TB regimen.

Recognizing that detection of resistance to both rifampicin and Isoniazid would have better outcomes than rifampicin alone, full DST for first line drugs should be requested immediately following initiation of second line treatment on the basis of a positive Xpert MTB/Rif test.

#### Practical considerations for use of Xpert MTB/Rif

The following should be observed for any site for placement of an Xpert MTB/Rif equipment:

- Gene Xpert should be used at the peripheral level of the laboratory network as it has similar biosafety requirements to microscopy.
- Xpert should be used for testing of all persons with presumptive tuberculosis
- Ensure stable uninterrupted power supply, and use of UPS for each unit while in operation;
- Ensure adequate and secure storage space for Xpert Cartridges;
- There should be a dedicated staff to perform the Xpert tests;
- Ensure calibration of the Xpert Module after every 2000 tests or one year, whichever comes first;
- The use of Gene Xpert on other samples other than respiratory is not validated yet.

**Gene Xpert should be performed for all patients with symptoms suspected of PTB**

**Gene Xpert should not be used as follow-up tests for monitoring response to TB treatment**

#### Use of Xpert MTB/Rif results

Upon receiving the Xpert results, treatment decisions should be made as follows:

- All patients whose diagnosis of TB has been confirmed by Xpert MTB/Rif but negative for Rifampicin resistance (i.e MTB +ve and Rif –ve) should be registered as bacteriologically confirmed tuberculosis ***(MTB positive)***, and started on first line anti-TB treatment;
- No additional microscopy is required for establishing baseline smear result in persons diagnosed using the Xpert;
- Xpert MTB +ve patients should be monitored while on treatment using smear microscopy at the recommended intervals until completion of treatment;
- Patients with TB and rifampicin resistance confirmed by Xpert should be registered as ***Rifampicin-resistant tuberculosis (RR-TB).***
- RR-TB diagnosis is considered a proxy for MDR-TB; and should therefore be started on the standardized MDR-TB treatment regimen;
- Xpert diagnosed MDR-TB patients should be monitored by sputum microscopy and culture as per the National MDR-TB management guidelines.

***Note: registration of diagnosed TB cases using conventional TB diagnostics remains unchanged if the results of Xpert MTB/Rif is not available*.**

#### Repeat of Gene Xpert test

A repeat of the Gene Expert test should be requested only in the following circumstances:

- When the test is negative but patient’s symptoms highly suggestive of TB;
- Patient does not show improvement on broad spectrum antibiotics;
- If the repeat Xpert MTB/Rif test is negative, culture and DST should be requested, while patient is started on New case TB treatment regimen.

### Use of interferon gamma tests:

The identification of genes in the *M. tuberculosis* genome that are absent from *M. bovis,* BCG and most non-tuberculous mycobacteria has supported the development of more specific and sensitive tests for detection of *M. tuberculosis* called interferon gamma radioassay tests (IGRAs).

Currently available commercial IGRAs work on the principle that the T-cells of an individual who have acquired TB infection will respond to re-stimulation with *M. tuberculosis*-specific antigens by secreting interferon-gamma.

The tests include:

- The QuantiFERON-TB Gold (QFT-G, Cellestis, Australia) and the newer version QuantiFERON-TB Gold In-Tube (QFT-GIT, Cellestis, Australia) are whole-blood based enzyme-linked immunosorbent assays (ELISA) measuring the amount of IFN-ᵧ produced in response to specific *M. tuberculosis* antigens (QFT-G: ESAT-6 and CFP-10, QFT-GIT: ESAT-6, CFP-10, TB7.7).
- The enzyme-linked immunospot (ELISPOT)-based T-SPOT.TB (Oxford Immunotec, UK) measures the number of peripheral mononuclear cells that produce IFN-ᵧ after stimulation with ESAT-6 and CFP-10.

Both IGRAs and the TST are surrogate markers of *M. tuberculosis* infection, indicating a cellular immune response to recent or remote sensitization with *M. tuberculosis.*

However, currently, there is no gold standard for the detection of *M. tuberculosis* infection, and neither the TST nor IGRAs can distinguish TB infection from active TB disease.

IGRAs have not been endorsed by WHO, and hence not recommend in this guideline for the diagnosis of TB in adults or children.

### Use of Commercial sero-diagnostics

Serological tests for diagnosis of tuberculosis are tests developed on the basis of antibody recognition of antigens of Mycobacterium tuberculosis by the humoral immune response, as opposed to antigen recognition by the cellular immune response (e.g. interferon-gamma release assays).

Sero-diagnostic tests for tuberculosis are currently not endorsed by WHO, and hence not recommend in this guideline for the diagnosis of TB in adults or children.

## Diagnosis of drug resistant tuberculosis

### Line Probe Assay (LPA) method

The Line Probe Assay (LPA) is molecular method for rapid detection of resistance to both rifampicin and INH (MDR-TB) in a sputum sample. LPA indirectly detects presence of *Mycobacterium tuberculosis* by amplifying DNA present in the sputum by polymerase chain reaction (PCR), which can be subsequently be visualized on a strip by the presence or absence of bands. Results of LPA tests are obtainable within a 48 to 72 hours period, which will enable timely initiation of MDR-TB treatment.

The indications to send sputum samples for Line Probe Assay are:

- Smear positive diagnostic samples from facilities that are yet to have access to Gene Xpert.
- Sputum from patients with a Xpert MTB +ve and Rif -ve

### Mycobacterial culture and drugs susceptibility testing (DST)

Mycobacterial culture method is considered the gold standard. Culture significantly increase the number of TB cases found (often by 30–50%) and allow earlier detection of cases (often before they become infectious). Culture also provides the necessary isolates for conventional drug susceptibility testing (DST). The disadvantage of this method lies in the relatively high turn-around-time (TAT) for obtaining the result.

Two culture methods have been adopted in Swaziland namely:

- The conventional Solid Culture method (L-J techniques)
- Liquid culture using the Mycobactria Growth Indicator Tube (MGIT), an automated system, which has about 10% sensitivity gain over solid media culture.

The MGIT method therefore reduces the TAT for culture results from about 60 days in the case of L-J to about 15-30 days. The results can be reported as early as 10 days (if the culture is positive), or up to 42 days to report a final culture-negative result. However, the system is more prone to contamination, and the manipulation of large volumes of infectious material.

Good laboratory practice should be ensured to maintain the delicate balance between the yield of mycobacteria and contamination by other microorganisms.

Mycobacterial cultures should always be performed in containment laboratories with biosafety level BSL III.

Positive cultures must be speciated to differentiate M. tuberculosis from non-tuberculous mycobacteria (MOTT), which are more common in HIV-infected patients.

#### Thin Layer Agar (TLA) culture and DST method

Thin Layer Agar (TLA)uses solid media which is impregnated with isoniazid and rifampicin to allow bacilli to grow in their favourable environment, and few days later to observe the specimen under direct microscopy.

The growth of MTB in media containing Rifampicin or Isoniazid is then directly visualized, thereby giving DST results for R and INH.

The advantage of this method lies in its rapidity, with culture and DST results being reported simultaneously in 7-10 days, and it’s relatively low cost per test.

This method has been introduced in Nhlangano Health centre as a demonstration site, with the potential for scale up depending on its overall benefits in relation to other culture/DST tests available.

## Sputum transportation

The quality of sputum sample submitted for bacteriological examination for tuberculosis is critical in determining the correct outcome of the test.

The Xpert MTB/Rif is particularly sensitive, and require a very good quality sputum sample without particles to avoid error reading by the equipment. The sputum sample for Xpert MTB/Rif should be processed and read with 72 hours of collection.

The National Sample Transportation System should ensure collection of sputum samples from the requesting health facility to the referral laboratories, where the following takes place:

1. Samples for microscopy/Gene Xpert are processed at the referral laboratories and results are sent back to the clinics.
2. Samples that require culture and LPA will be sent to NTRL through DHL courier service.

NB: The triple packaging system should be strictly observed when transporting sputum samples

## Role of Other Investigations in TB Control

### Role of Chest x-rays

Chest X-rays are useful to identify presumptive tuberculosis in routine screening and assessing the extent of complications of lung diseases. The common Chest X-ray findings associated with pulmonary tuberculosis include upper lobe infiltrates, bilateral infiltrates, cavitations, pulmonary fibrosis and shrinkage.

However, the sensitivity of Chest X-ray in the diagnosis of tuberculosis is low. Furthermore, there is NO Chest X-ray appearance that is typical for PTB as many other conditions mimic tuberculosis. These conditions include but are not limited to the following: bacterial pneumonia, lung abscess, fungal infection, bronchial carcinoma, connective tissue disease, occupational lung disease, sarcoidosis, and lymphoma. Additional specific tests might therefore be needed to rule out these diseases or conditions.

The use of Chest X-ray in the diagnosis of pulmonary tuberculosis is therefore relatively unreliable, but can be useful in diagnosing extra-pulmonary TB e.g miliary tuberculosis, pleural TB with effusion, pneumothorax, pericarditis, etc.

Chest X-ray is not necessary in a case where the sputum smear result is positive.

The absence of a chest X-ray should not be an obstacle to diagnose and initiate TB treatment.

Chest X-rays are not necessary for the routine follow-up of a patient on TB treatment.

They are not required to change to continuation phase or to stop treatment in patients who are clinically responding well to TB therapy.

Chest x-rays are contra-indicated in pregnancy especially during the first trimester.

## Revised diagnostic Algorithms according to existing TB diagnostic equipment in Swaziland

The National programme has adopted the Xpert MTB/Rif as the initial diagnostic test for all persons with presumptive tuberculosis in view of the high HIV and MDR rates among incident cases, while bacteriological monitoring of treatment will be through LED microscopy. However, the roll-out of the Xpert MTB/Rif and other new TB diagnostic tests described in 6.4.1 shall be phased until the ultimate goal is achieved.

Two algorithms are therefore currently recommended for use in Swaziland:

1. Algorithm 1: for health facilities without the Gene Xpert
2. Algorithm 2: for health facilities with Gene Xpert

### Diagnostic Algorithm for settings without Xpert MTB/Rif

1. Includes MDR TB suspects

2. For discrepant results between genotypic and phenotypic tests refer to guidelines

Sputum microscopy / LED microscopy (2 smears)

Negative or smear not done

Positive

Further Clinical assessment

Course of antibiotics

Suggestive of TB

R+/H+

R+/H-

R-/H+

LPA

Treat with FLD.

R-/H-

DST for FLD & SLD^2^ and adjust SLD regimen.

Follow up 2, 5 months with Sputum microscopy

Positive

Negative

Continue treatment

Culture/DST for FLD

INH and RIF Resistance

Susceptible or other resistance

Treat with SLD

and send for SLD DST

Initiate appropriate treatment. Assess adherence

Follow up monthly with sputum microscopy and culture

Treat as Clinically diagnosed TB and treat with FLD.

Modify treatment.

Do FLD & SLD DST. Adjust treatment accordingly

Not suggestive of TB or improved on antibiotics

Consider other diagnosis

### Diagnostic Algorithm for settings with Xpert MTB/Rif

HIV test and Xpert MTB/Rif^2^

Xpert MTB+/Rif+

Xpert MTB+/Rif-

Xpert MTB-/Rif-

Treat with Standard MDR-TB regimen

Treat for TB with FLD

Send for LPA. IF INH Resistant modify R and send for FLD DST

Further clinical assessment including Chest X-Ray, Antibiotics

DST for FLD&SL and adjust SLD regimen according to results

lts

Suggestive of TB

Not suggestive longer considered TB suspect

Investigate for EPTB or other disease;

Initiate TB treatment on clinical grounds and

Send for MGIT Culture /DST for FLD

Exit algorithm.

Retest with Xpert

MTB+

MTB-

Follow up 2, 5 months with Sputum microscopy

Culture / DST for FLD^2^

Positive

Negative

Continue with FLD treatment

1. Includes MDR TB suspects

2. One sputum specimen

3. For discrepant results between genotypic and phenotypic tests refer to guidelines

**Note**

- Infection control measures should commence immediately
- In the absence of Rapid resistance testing, the algorithm can be followed using liquid culture

Initiate appropriate treatment. Assess adherence

No Rifampicin and INH Resistance

Rifampicin and INH Resistance

Treat with SLD and send for SLD DST

Follow up monthly with sputum microscopy and culture

lts

## Confirming diagnosis of Extra-Pulmonary TB

Extra-pulmonary tuberculosis diagnosis is confirmed under the following situations:

- One specimen from an extra-pulmonary site smear positive for AFB or culture-positive for *M. tuberculosis;*

**OR**

- Histological or strong clinical evidence consistent with active extra-pulmonary tuberculosis **and**
- Laboratory confirmation of HIV infection **or**
- Strong clinical evidence of HIV infection;

**And**

- A decision by a clinician to treat with full course of anti-tuberculous chemotherapy.

All EPTB cases including those diagnosed by histological examination should be reported under ***clinically diagnosed cases of TB.***

Table 2.1: Table showing symptom-based approach to the diagnosis of Extra-pulmonary TB

| **TYPE OF EPTB** | **SYMPTOMS** | **DIAGNOSIS** |
| --- | --- | --- |
| **TB Lymphadenitis (most common EPTB site)** | Fever, weight loss, fatigue and occasionally night sweats or no symptoms at all.  Enlarged lymph nodes (>2cm). They can break down due to the formation of caseous pus. Cervical LN are the most common.  Mediastinal lymph nodes or abdominal lymph nodes, if large may obstruct nearby organs thereby producing such symptoms as cough, dysphagia, intestinal obstruction, etc. | Lymph node aspiration of the caseous pus and send for microscopy, culture and DST.  If aspirate is dry then a lymph node biopsy should be taken, sending the material for histopathology and culture/DST. |
| **Pleural TB (Extra-pulmonary TB)** | Acute or sub-acute illness varying from a few days to few weeks. Pleuritic chest pains, non-productive cough and dyspnoea, sometimes fever.  In an empyema, the patient is acutely ill with chest pains, breathlessness, and cough with expectoration, fever and toxaemia. Occasionally it may present as a chest wall mass or draining sinus tract. | By therapeutic/diagnostic tap and sending the fluid for microscopy and culture / DST.  If the patient has empyema, he must be admitted; the empyema drained and send the material for microscopy, culture and DST. |
| **TB meningitis** | Meningism (neck stiffness, kerning’s sign), irritability, anorexia, vomiting, fever and sometimes seizures.  Complete or partial loss of vision is a major complication of TBM. Without treatment the patient may descend into a coma and death would follow in five to eight weeks. Thus patient needs immediate admission. | Lumbar puncture for CSF (increased lymphocytes, increased proteins and decreased glucose).  CSF should be sent for microscopy and culture/DST.  If the patient is HIV + do also India ink and crptococcal antigen. |
| **Abdominal TB** | Symptoms are non-specific and depend on the site and extent of the disease.  Loss of appetite, malaise, diarrhea, low grade fever, weight loss, night sweats, ascitis, masses or abscess, obstructive jaundice, etc | Diagnostic/therapeutic tap and send off for microscopy and culture/DST . |
| **Pericardial TB** | Fever, weakness, pericardial rub, vague chest pains, dyspnoea, cough, elevated jugular venous pressure, weight loss. | Clinical diagnose, cardiomegaly in the chest Xray and echocardiogram.  In case of pericardiocenthesis, liquid should be sent for microscopy and culture/DST. |
| **TB of the bones and joints** | Spinal TB (Potts disease) is the most common, usually constitutional symptoms (weakness, loss of appetite and weight, night sweats) will be present before any signs of spinal involvement (chronic back pain and gibbous deformity). If untreated, patient can develop neurological deficits and paraplegia.  TB of the joints affects the movement of the affected joints and produce pain. | X ray spine will show erosion of adjacent vertebral bodies and disc space narrowing.  These patients need to be referred to a specialist for treatment. |
| **Miliary TB** | Constitutional symptoms (fever, night sweats, loss of weight) and hepato-splenomegaly. They may also have a cough. | Chest X-ray: diffuse military nodules.  Sputum can be negative if not lung parenchyma is compromised. |

### Tuberculous Lymphadenopathy

Tuberculous lymphadenitis should be suspected in any patient with enlarged lymph nodes that are firm, asymmetrical, more than 2 cm in diameter, or where a node has become fluctuant or developed a fistula over several months. It most commonly affects the nodes in the neck(cervical region) and is difficult to distinguish clinically from other causes of enlarged nodes, such as reactive and/or HIV-related lymphadenopathy, malignancies and other lymph node infections, which are also common. Therefore,

needle aspiration using recommended techniques should be carried out at the first outpatient visit for all patients.

Diagnosis can be confirmed by biopsy and demonstration of histological evidence. Where the capacity for histology does not exist, the patient can be started early on anti-TB treatment based on the decision of a Medical Officer to treat as extra-pulmonary TB.

### Miliary TB

Miliary TB results from widespread blood borne dissemination of TB bacilli. This is either the consequence of a recent primary infection or the erosion of a tuberculous lesion into a blood vessel.

The patient presents with constitutional features (fever, night sweats and weight loss). Hepatosplenomegaly may be present and choroidal tubercles on fundoscopy. Miliary TB is an under-diagnosed cause of end stage wasting in HIV-positive individuals. Diagnosis should be established using Chest x-ray findingshowing diffuse, uniformly distributed, small miliary nodules (“miliary” means “like small millet seeds”) which is pathogneumonic of that form of the disease.

### Tuberculous pleural effusions

Inflammatory tuberculous effusions may occur in any of the serous cavities of the body, i.e. pleural, pericardial or peritoneal cavities. They are a common form of TB in HIV- positive patients.

- Patients usually have systemic and local features.
- Microscopy of the aspirates from tuberculous serous effusions rarely show AFB because the fluid forms as an inflammatory reaction to TB lesions in the serous membrane.
- Finding of a straw coloured fluid from the pleural tap is highly indicative of TB pleural effusion and should be treated as such.

### TB Meningitis

TB meningitis results from rupture of a cerebral tuberculoma into the subarachnoid space or blood-borne. It is a life threatening condition with serious complications if not treated promptly. Diagnosis is confirmed by the demonstrating the relevant clinical signs backed with positive laboratory results:

#### Clinical Features

- - - - Patients present with gradual onset of headache and decreased consciousness.
      - Examination reveals neck stiffness and positive Kernig’s sign (flex one of the patient’s legs at hip and knee with the patient lying on back, and then straighten the knee - resistance to straightening the knee and pain in the low back and posterior thigh suggest meningeal inflammation).
      - Cranial nerve palsies resulting from exudates around the base of the brain.
      - Tuberculomas and vascular occlusion may cause focal neurological deficits and seizures.
      - Obstructive hydocephalus may develop.
      - Spinal meningeal involvement causes paraplegia (spastic or flaccid)

#### Laboratory diagnosis

- Lumbar puncture to examine cerebrospinal fluid and the following features indicate a positive test:
  - Clear CSF
  - Elevated pressure
  - High levels of protein (>1g/ l)
  - High lymphocyte count (30-300/mm³)
  - Low glucose
  - AFBs on microscopy in a minority of cases.
  - Some of the CSF findings may be normal, especially in HIV-positive patients.

Patients with presumtive TB meningitis should be referred to hospital without delay.

#### Treatment of TB Meningitis (TBM)

Patients diagnosed with TB meningitis should be treated with the following regimen: **2SRHZE / 7RH**

In view of the intensity of the inflammatory and fibrotic reactions at the meningeal site, adjunctive corticosteroid therapy, in addition to anti-TB treatment is recommended.

Patients with low state of consciousness (a Glasgow Coma Scale score of less than 15) or who have a focal neurological deficit should be treated with intravenous dexamethasone for 4 weeks (0.4 mg/kg per day in week 1, 0.3 mg/kg per day in week 2, 0.2 mg/kg per day in week 3, and 0.1 mg/kg per day in week 4), followed by a taper of oral dexamethasone (4 mg/day, 3 mg/day, 2 mg/day and 1 mg/day, each for a period of 1 week).

Patients with a normal mental status and no neurological findings receive intravenous dexamethasone for 2 weeks (0.2 mg/kg per day in week 1, then 0.1 mg/kg per day in week 2), followed by the same oral taper as described above.

It is recommended that the steroid treatment should start as soon as possible after initiation of appropriate first-line anti tuberculosis drugs.

### Tuberculosis of the spine

This is a severe form of tuberculosis when there are neurological sequelae. It is seen both in children, usually within three years following primary infection, and in adults. In many cases more than one intervertebral disc space is involved.

As the disease develops, the vertebral body adjacent to the disc space is affected; an abscess is formed and spreads either forward towards the mediastinum or the retroperitoneal space, to the vertebral body with compression of the spinal cord, or back along the vertebral column eventually appearing as a subcutaneous “cold” abscess. Collapse of adjacent vertebral bodies affected by tuberculosis may lead to angulated kyphosis. The sites most commonly involved are the lower thoracic, lumbar and lumbo-sacral areas.

The main differential diagnoses are malignancy and pyogenic spinal infections. Malignant deposits in the spine tend to erode the pedicles and spinal bodies, leaving the disc intact. Pyogenic infection tends to be more acute than TB, with more severe pain. Diagnosis can be confirmed through X-ray of the spine revealing typical findings consistent with destruction of inter-vertebral disc.

1. QUALITY ASSURANCE OF LABORATORY SERVICES

## Importance of Laboratory Services in TB Control

A well-functioning laboratory is the first requirement for successful management of tuberculosis. This is in view of the fact that a reliable diagnosis and treatment follow-up is necessary to inform treatment decisions that ultimately leads to cure of infectious TB cases; thereby interrupting transmission of the disease. The laboratory also has a major role in surveillance of the TB situation in the community, incidence, prevalence, drug susceptibility patterns, etc).

TB diagnosis should be made as close as possible to the patient’s residence, while maintaining the proficiency of the testing procedures.

The Gene Xpert test has been adopted as the initial diagnostic test for TB in view of the increased sensitivity gain over the Ziehl-Neelsen method. However, culture and drug susceptibility testing (DST) may be performed at the start of treatment especially for HIV coinfected TB patients. Smear microscopy should be used in the serial monitoring of the patient’s response to treatment.

For this reason, it is necessary for the entire population to have access to quality-assured TB laboratory services. Culture should be selectively used in the following circumstances^[[3]](#footnote-3)^:

1. Surveillance of tuberculosis drug resistance as an integral part of the evaluation of control programme performance
2. Diagnosis of cases with clinical and radiological signs of pulmonary tuberculosis where smears are repeatedly negative
3. Diagnosis of extra-pulmonary and childhood tuberculosis
4. Follow-up of tuberculosis cases who fail a standardized course of treatment and why may be at risk of harbouring drug resistant organisms
5. Investigation of high-risk individuals who are symptomatic, eg. Laboratory workers, health care workers looking after multi-drug resistant patients

## TB laboratory Network in Swaziland

Tuberculosis laboratory services in Swaziland forms part of overall laboratory services in the country, and is organized according to the technical complexity, activities performed and functional roles as follows:

1. Central level (the National Reference laboratory )
2. Intermediate level (the Regional Laboratories/x
3. Peripheral laboratories (health centre and clinic laboratories)

### The National Reference laboratory

The National TB Reference Laboratory (NRL) is situated within the National Reference Laboratory and Blood Bank Complex in Mbabane. The NRL performs mycobacterial species identification, LPA, MGIT culture and first line drug susceptibility testing (FLD DST). Beside the core technical activities, the NRL should provide capacity building and training for laboratory staff, external quality assessment (EQA), contribute to surveillance of tuberculosis including drug resistance and participate in epidemiological and operational research. Establishment of tuberculosis culture facilities at in the country aims to achieve at least 1 centre per 500 000 population.

### The Regional laboratories

The regional laboratories, which are located within the hospitals, health centres and some clinics in the country primarily perform direct sputum smear microscopy using either the conventional Z-N technique or the LED microscopy. The national TB programme and the National clinical laboratory service aims to have at least one microscopy centre is serving about 50,000 population.

## Assuring Quality of Smear Microscopy

Results of TB laboratory investigations are critical for diagnosis and follow up of patients on treatment according to national guidelines. Hence, the credibility, success and sustainability of the programme depends on the capacity of TB laboratory network to produce reliable results.

Poor quality diagnosis results in failure to detect persons with infectious TB, who will continue to spread infection in the community, or unnecessary treatment of “non-TB cases.” Errors in the reading of follow up smears may result in wrong outcome of patients often with severe consequences to the community.

In order to achieve the required technical quality in laboratory diagnosis, a continuous system of quality assurance needs to be established. Intermediate laboratories should supervise the peripheral network, while the central or reference laboratory should supervise the intermediate network.

An effective quality assurance (QA) system of sputum smear microscopy network is of crucial importance for the programme. QA is a comprehensive system consisting of internal quality control (QC), assessment of performance using external quality assessment (EQA) methods, and continuous quality improvement (QI) of laboratory services. To optimize QA, the supervision and monitoring of the laboratory network is essential. This process requires the active support and participation of the NRL and regional laboratories. The definitions of QC, EQA and QI are explained below.

**Quality Control (QC)** or Internal Quality Control, includes all the ‘bench-top’ procedures by which the laboratory personnel performing TB smear microscopy control the process, including checking of instrument, new lots of staining solutions, smear preparation, grading etc. It is a systematic internal monitoring of working practices, technical procedures, equipment, and materials, including quality of stains.

**External Quality Assessment (EQA)** A process to assess laboratory performance. EQA includes ‘on-site evaluation’ (OSE) of the laboratory to review QC and evaluation of entire process of smear microscopy, and random blinded re-checking of routine smears. EQA also allows participant laboratories to assess their capabilities by comparing their results with those obtained in other laboratories in the network (intermediate and central laboratory) through panel testing and rechecking of patient slides, using both un-blinded and blinded procedures. EQA is also termed “Proficiency Testing” as described by IUATLD.

**Quality Improvement (QI)** A process by which all components of smear microscopy diagnostic services are carefully analyzed, periodically, with the aim of looking for ways to permanently remove obstacles to success. Appropriate data collection, data analysis, correct interpretation of the results and creative problem solving, are the key components of this process. It involves continued monitoring, identifying defects, followed by remedial action including retraining when needed, to prevent recurrence of problems. QI mostly relies on effective on-site evaluation visits.

### External Quality Assessment (EQA)

External Quality Assessment is one of the most important components of a laboratory QA program.

The national TB reference laboratory should play an essential role in the organization and maintenance of the network in terms of developing guidelines, ensuring high quality and standardized smear microscopy, and therefore must have the capacity to provide training and External Quality Assessment, including providing panel testing and rechecking to intermediate and peripheral laboratories.

EQA should focus on identification of laboratories where there may be serious problems resulting in poor performance, and not on the identification of individual slide errors or the validation of individual patient diagnosis. It is also a very important tool for communication with and motivation of laboratory technicians who may otherwise feel isolated in their work. There are three methods that should be combined to evaluate laboratory performance:

• On-site Evaluation

• Panel Testing

• Blinded Rechecking

### On-site evaluation of Microscopy Centers:

The on-site evaluation includes a comprehensive assessment of laboratory safety, condition of the binocular microscope, adequacy of supplies as well as the technical components of sputum smear microscopy, including preparation, staining and reading of smears. On-site evaluation should always include macroscopic as well as microscopic examination of randomly selected 5 stained positive and 5 negative smears.

Checklists should be used to assist supervisors during the field visit and to allow for the collection and analysis of standard data for subsequent remedial action. The copies of the checklist, duly completed by the Supervisors, should be handed over to in-charge of the laboratory as well as the Hospital authorities. This will provide written documentation of the visit, its findings and proposed corrective actions for improvement.

The On-site Evaluation visits should be conducted to every regional and peripheral laboratory semiannually. A comprehensive checklist for on-site evaluation of Microscopy centers is provided as annexure.

##

### Panel Testing

Panel testing is a method of EQA that evaluates a technician’s individual performance in staining and reading, and not the whole laboratory activities. Utilization of panel testing for EQA is considered to be less effective than random blinded rechecking of routine slides because it does not monitor routine performance.

Panels are to be prepared and distributed to all laboratories on a quarterly basis, which should be followed by analysis of results and feedback to facilities.

The panels should consist of a set of 10 panel slides, including negatives and covering all the positive grades of test smears. These slides should be read and graded within the normal routine programme conditions. Based on the results, gaps and remedial actions including training will be determined to address technical skill deficiencies and errors to achieve higher level of proficiency.

Insert paragraph on panel testing system

### Random Blinded Rechecking of Routine Slides

Blinded rechecking is a process of re-reading a statistically valid sample of slides from a laboratory to assess whether that laboratory has an acceptable level of performance. This method provides reliable assurance that NTP is supported by an efficient and reliable sputum microscopy laboratory network.

Random blinded rechecking involves selection of a representative sample of slides from a Microscopy Center (both positives and negatives). The results of the slides are blinded before being read by a supervisor (first controller) in an un-biased manner. The discrepant results are resolved by a higher supervisor (umpire reader). A timely feedback is provided every month to the laboratory staff heads of labs for improvement in the quality of microscopy.

The Central and intermediate laboratories would also be supervising the peripheral Microscopy Laboratories on a routine basis; and reports of their visits should be handed over to in-charges at all levels. Corrective measures should be implemented based on findings of these reports.

**Operation of the blinding rechecking using the IQLS system**

All regional and peripheral labs participate in blinded rechecking EQA and on a quarterly basis, each lab provides 40 slides for rechecking. A sample size of 40 slides per lab (microscopy centers) for the EQA per site was determined using the Lot Quality Sampling method basing on the annual volume of slides read. A blinded rechecking team of 8 lab technologists, was established to pick slides and re-read them.

Each quarter, 40 slides are randomly picked at each participating lab and their results recorded on the IQLS blinded rechecking form. The form is sent for data capture into the IQLS system and filing a Data Analyst at the NRL while the slides are being re-read. The results of second reading are also recorded independently including details on the quality of stain on slide, thickness, evenness and stain grade.

The results entered into the IQLS system will generate a comparison table for the two readings for each lab. The Data Analyst prints the comparison tables which are given to the rechecking team and discordant slides picked for a third reading. The slides are re-read by a different technologist and the result is recorded and captured as the tie-breaker and final. The final comparison list is now sent back to their original laboratories through rechecking teams who will also give feedback and technical assistance on the findings.

## Conducting visits to microscopy centres

Microscopy Laboratories are supervised by supervisors from the national and provincial level. The NTP will work with the Supervisors to make sure that tuberculosis-related laboratory services are performed according to national guidelines. Visits to the microscopy centres must be adequately planned, and a checklist should be used.

### Preparing for visits:

1. Supervisory visits to be planned in advance such that all laboratories are visited at least every quarter from National laboratory supervisor. Information should be given in advance about the visit to the laboratory.
2. Review the recommendations made during previous visits and the actions taken.
3. Ensure availability of an updated lab supervision checklist (Laboratory supervisory check-lists: given at annexure).

### Conducting the visit

Visiting the laboratory requires good time management to ensure a productive supportive visit without significantly disrupting the daily work schedule of the supervisee. The supervisor should be focused and be systematic in conducting the visit. The following techniques could be employed to check the laboratory operations:

- 1. Review the Tuberculosis Laboratory Register for completeness, consistency and accuracy of recording; and verify that monthly summaries are made correctly.
  2. Discuss with the laboratory technicians: to verify their understanding of the national guidelines concerning the correct number of sputum specimens required for diagnosis and follow up examinations; the importance of limiting administrative errors and accurately recording the results of sputum smear examinations on the Laboratory Form for Sputum Examination; and storing the examined sputum smear slides of all patients until the EQA purposes.
  3. Examine supplies: to determine if there are adequate numbers of sputum containers, slides, reagents, forms and other laboratory supplies for the expected patient turnover.

### Follow up Quality improvement

The findings of the supervision visit should be discussed with the supervisee with the view to finding solutions to problems detected. This should include on the job capacity building where required.

The supervisor should within one week produce a report of the supervisory visit and forward it to the higher authority. A copy of such a report should also be made available to the head of the Hospital and the laboratory visited.

Adequate follow up should be ensured concerning the recommendations of the report.

## Monitoring documentation related to microscopy examinations and other diagnostic methods

Every TB Microscopy laboratory must have a Tuberculosis Laboratory Register, which should be filled up completely and accurately to ensure that the results are entered for the right persons.

- In processing sputum sample for examination, the sputum containers and slides should be marked correctly with Laboratory Serial Number, and accurately record the results of sputum smear examinations on the form.
- Futhermore, all examined slides should be kept *serially* in the box without segregation of positive and negative slides, until the Laboratory Supervisor reviews them for quality assurance.
- During the on-site visit, the STLS should select five smear-positive and five smear-negative slides randomly and review them as per QA protocol.
- Ensure that the Microscopy laboratories and health facilities which collect and transport sputum are visited at least once every month. Other health facilities which collect specimens and transport them to an off-site laboratory should assign Specimen Identification Numbers and write it on the side of the containers.

### Laboratory Request form

The laboratory request form is the first line of communication between the specimen submitting facility, agency, or physician and the laboratory. These forms are available upon request to the laboratory. Correctly completing this form will insure your patient and specimen are properly identified and matched, the requested procedures are performed in a timely manner, and the results get back to the facility, agency, or physician as requested. All results will be delivered by had electronically or faxed to the ordering facility, agency, or physician only.

The request form includes a place to enter many identifying elements. The national clinical laboratory service adopted a single the laboratory request form- **clinical laboratory services general request form** (see annex 16) , for all specimens including sputum testing for tuberculosis. It has a place for the ordering physician or agency, the patient information, specimen information, medical necessity justification, and procedures requested.

### Tuberculosis laboratory register

The Tuberculosis Laboratory Register is used to record the results of sputum smear examinations. The register should contain the patient’s personal data as well as name of the treatment facility, reason for examination and the results of the examinations. The following information about the patient is then recorded:

- Date of sputum smear examination
- Full name
- Sex
- Age
- Name of the health facility (e.g. primary health centre, private practitioner, NGO, etc.) that requested the examination
- Complete address
- Reason for examination (diagnosis, repeat diagnosis and follow-up of chemotherapy).
- Results of sputum smear examinations (results of specimens 1, 2 and 3 can be recorded).

The last two columns of the Tuberculosis Laboratory Register are for the Laboratory Technician’s (LT) remarks and signature.. The remarks column can also mention in brief the action taken for patients belonging to other treatment units or Regionals, e.g., “Referral.

Every week the in Charge of the laboratory should review the Tuberculosis Laboratory Register to ensure that correct numbers of sputum smear examinations (i.e. 3 per person with presumptive TB) are being performed for diagnosis.

Regional TB Coordinators should endeavour to compare sputum results mentioned in the Tuberculosis Laboratory Register with those mentioned in the TB Treatment Cards and TB Registers. This can be done by randomly selecting cases from the facilities to cross-check their results in the laboratories.

Laboratory staff should not use the Tuberculosis Laboratory Register to record the results of any other laboratory examinations. All results of sputum smear examinations done in a Microscopy Centre should be written only in one Tuberculosis Laboratory Register, and not in any other register.

**Ensure that the patients for diagnosis have two sputum samples examined and follow-up cases have at least one sputum sample examined.**

**Up to three-sputum specimen examination results can be recorded for each**

**patient on one row of the Tuberculosis Laboratory Register.**

The laboratory technician should summarize the information on sputum smear examinations done during that month. This information should be summarized in the monthly summary form (See annex) at the end of each month, printed in the Laboratory Register itself. Patients from the following month should be started from the next new page.

### Disposal of laboratory materials.

Sputum specimens examined in the laboratory are potentially infectious. Hence, after examination, they must be disinfected and destroyed so that the risk of infection is avoided. All disposable containers must be used only once.

Sputum cups which contain sputum can be disposed of by any one of the following methods:

- - - - **Disinfection:** After the sputum smears are examined, all sputum cups should be kept in a bucket containing 5% hypochlorite, or 10% bleach solution (freshly prepared), or 5% phenol solution. Caps of the sputum cups must be removed and the cups, caps and wooden sticks completely submerged in the solution in a secure place for at least 18 hours. After this, the solution, cups, caps and broom sticks can be discarded with other hospital waste. This bin/bucket should have a lid which is foot operated.
      - **Incineration:** Wherever incinerators exist, the type specified under Biomedical Waste Management & Handling Rules of the country, with combustion efficiency of 99%, it should be used. Sputum cups made of polypropylene should be used wherever available. (Note: If sputum cups are made of other varieties of plastic, they should be disinfected and destroyed as per the hospital waste management rules). Burning is not recommended.
      - **Autoclaving:** The sputum cups and lids, with the lids removed, along with wooden sticks can be autoclaved at the end of each day’s laboratory work. The autoclave cycle should have a holding time of 15 minutes at 121 °C HTAT (Holding time at temperature), 10 minutes at 126 °C HTAT or 3 minutes at 134 °C HTAT. The material can be discarded with other waste after proper cooling.

If none of the above is available, cotton and broom sticks can be disinfected and buried at a safe distance away from inhabited areas in a landfill site ensuring deep burial as specified by the infectious material disposal rules of the country.

Used slides should not be broken. They should be disposed through the hospital waste management system or in a secured pit for sharps in accordance to prevailing guidelines. Slides once used for sputum microscopy should not be reused.

1. TREATMENT OF TUBERCULOSIS

## The aims of TB treatment

The key to interrupting the spread of TB in the community is early detection and effective treatment of persons who are coughing up viable TB bacilli.

The National TB programme aims to provide timely and appropriate treatment for all forms of tuberculosis under standard case management conditions to cure the patient of active disease, prevent death from TB or its complications, decrease transmission of the disease to others, and to prevent the development of drug resistance. This requires that correct combination of anti-TB medications are prescribed and administered at the right doses for the correct duration.

## Essential anti-TB drugs

Anti-tuberculosis drugs have three main properties namely bactericidal, sterilizing activity and the ability to prevent resistance. For anti-TB treatment to be effective, a combination of these properties is required in a treatment regimen.

- Isoniazid and rifampicin are the most powerful bactericidal drugs, active against all populations of TB bacilli.
- Pyrazinamide and streptomycin are also bactericidal against certain populations of TB bacilli.
- Pyrazinamide is active in an acid environment against TB bacilli inside macrophages.
- Streptomycin is active against rapidly multiplying extra-cellular bacilli.
- Ethambutol is bacteriostatic and is effective in preventing development of resistance against other anti-TB drugs. The following are the recommended first line anti-TB drugs and the dose range.

### Fixed-Dose Combinations

Fixed-Dose Combination (FDC) tablets are tablets containing 2 or more anti-TB drugs combined in fixed doses. Their advantages and disadvantages compared to single formulation drugs are shown below.

### Advantages of FDCs compared to single formulation drugs

- Prescription errors are less likely or less frequent because dosage recommendations are more straightforward and adjustment of dosages according to patient weight is easier.
- The pill burden on the patient is smaller and may thus encourage patient adherence.
- If treatment is not supervised, the patient cannot be selective in the choice of drugs to ingest.

Table 6.1: Essential anti-TB drugs

| **Essential TB drugs** | **Recommended Daily Dose**  **(Dose range in mg/kg)** | |
| --- | --- | --- |
|  | **ADULTS** | **CHILDREN** |
| Isoniazid (H) | 5 (4-6) | 10 (10-15) |
| Rifampicin (R) | 10 (8-12) | 15 (10-20) |
| Pyrazinamide (Z) | 25 (20-30) | 35(30-40) |
| Streptomycin (S) | 15 (12-18) | 15 (12-18) |
| Ethambutol (E) | 15 (15-20) | 20 (15-25) |

*Source: Treatment of tuberculosis guidelines. 4^th^Edition. WHO/HTM/TB/2009.420*

## Standard TB Treatment Regimens for Adults and adolescents

Treatment of all forms of TB in Swaziland is based on the WHO-recommended treatment regimens for the respective case registration groups.

For the purpose of treatment, TB patients fall into three broad groups based on previous TB treatment history namely:

1. **New TB cases:** All diagnosed cases with no history of prior treatment or received less than 1 month anti-TB treatment regardless of method of diagnosis;
2. **Previously treated TB cases:** All cases with prior history of TB treatment lasting at least 1 month;
3. **DR-TB Cases: A**ll cases diagnosed with rifampicin, or both rifampicin and Isoniazid resistance using rapid molecular DST methods.

Standardised treatment regimens have been adopted for the first two groups of patients based on efficacy and feasibility, and the need to minimize prescription errors, reduce costs, enhance training of staff and improve drug estimation, purchasing, distribution and monitoring.

All TB cases (New or previously treated) should have DST at or before initiation of TB treatment to determine the presence of resistance to any of the first line anti-TB drugs.

**Note:**

Obtaining specimen for conventional DST should not delay initiation of treatment and ALL relapse patients should be initiated on 2HREZ. Previously treated patients returning after failure should be referred for empirical MDR-TB treatment as DST results are awaited.

Treatment of third group requires design of the appropriate DR-TB regimen which is elaborated in the national MDR-TB management guidelines.

Treatment consists of two phases, an initial (or intensive) phase and a continuation phase.

The aim of the intensive phase in which 4-5 drugs are administered is to ensure rapid killing of the bacilli, while the continuation phase, which usually consists of fewer drugs (3-4) given for a longer period is to sterilise lesions and prevent relapse.

All medications in the Swaziland TB treatment regimens (in both intensive and continuation phases) are for **daily** administration.

The summary of the treatment regimens is as in table 6.2 below:

Table 3: Summary of TB treatment regimens for adult cases

| **TB Registration** | **TB Patients** | **Treatment Regimens** | |
| --- | --- | --- | --- |
|  |  | **Intensive Phase**  **(daily)** | **Continuation Phase (daily)** |
| **New Cases** | - All new cases of TB, regardless of site, type or severity of disease | **2HRZE** | **4HR** |
| **Previously treated** | - 2HRZE (and then treat based on DST) | **3RHZE** | **5HRE** |
|  | - Treatment failure | **Evaluate for MDR** |  |

*Source: Treatment of tuberculosis guidelines. 4^th^Edition. WHO/HTM/TB/2009.420*

TB is curable, regardless of site of disease or HIV status.

This very important should always be communicated to patients.

DOTS and patient support should be strengthened at each visit

Previously treated cases have a higher likelihood of harbouring drug-resistant TB. Sputum specimen should therefore be obtained for DST at or before initiation of TB treatment.

Fixed-Dose Combination (FDC) tablets are tablets containing 2 or more anti-TB drugs combined in a single tablet. FDCS have the advantage of easy of prescription and reduced pill burden, which may potentially enhance treatment adherence.

### Treatment of New tuberculosis cases – Adults and adolescents

All new TB cases (patients who have never been treated for TB in the past or who has taken anti-tuberculosis drugs for less than one month) should receive RHZE in the first 2 months initial phase; and RH in the 4 months of continuation phase.

TB treatment regimens have a standard code. Anti-TB drugs are abbreviated as shown below:

**2(RHZE) / 4(RH)**

The code indicates the following:

- both treatment phases (intensive and continuation) which are separated by a slash;
- the treatment duration in each phase in months denoted by the number preceding the bracket.
- anti-TB medications represented by the letters within the brackets
- brackets denoting that all drugs within it are in a fixed dose combination form.

The code includes both treatment phases, which are separated by a slash. A number is placed before a phase to indicate the duration of that phase in months. Letters enclosed in brackets indicate fixed-dose combinations.

Table 6.3: Recommended treatment regimen and anti-TB drug dosages for New TB cases

| **Phase of treatment** | **Drugs** | **Weight in Kg** | | | |
| --- | --- | --- | --- | --- | --- |
|  |  | **30-39** | **40-54** | **55-70** | >70 |
| Intensive phase of 2 months | **(RHZE)*** (150mg/75mg/400mg/275mg) | 2 | 3 | **4** | **5** |
|  | | | | | |
| Continuation phase of 4 months | (RH) (**150mg/75mg)** | **2** | **3** | **4** | **5** |

*Source: Treatment of tuberculosis guidelines. 4^th^Edition. WHO/HTM/TB/2009.420*

***Fixed-dose combination (FDC) drugs**

**Note:** It is important to note that the duration of treatment for TB Meningitis, Miliary TB, and TB of the Bones and Joints is **9 months**, whilst all other forms of TB are treated for a period of 6 months for new cases. In the context of HIV (HIV/TB – co-infection), CNS TB should be treated for 9 – 12 months.

### Treatment of previously treated cases

All preveiously treated TB cases should be initiated on the 8-month regimen consisting of an initial three (3) months intensive phase and five (5) months continuation phase coded as follows:

**3(RHZE) / 5(RHE)**

Table 6.4: Recommended treatment regimen and dosages for Adult cases of relapse and return after loss to follow up.

| **Phase of treatment** | **Drugs** | **Weight in Kg** | | | |
| --- | --- | --- | --- | --- | --- |
|  |  | **30-39** | **40-54** | **55-70** | >70 |
| Intensive phase of 3 months | **(RHZE)** (150mg/75mg/400mg/275mg) | 2 | 3 | 4 | **5** |
| Continuation phase of 5 months | (RHE)**(150mg/75mg/400mg)** | **2** | **3** | **4** | **5** |

*Source: Treatment of tuberculosis guidelines. 4^th^Edition. WHO/HTM/TB/2009.420*

Patients whose treatment has failed or grouped under the Óther’group may have high likelihood of MDR-TB, and can be started on standardized MDR-TB treatment according to the National MDR-TB management guidelines.

### Treatment of extra-pulmonary tuberculosis

Pulmonary and extra-pulmonary disease should be treated with the same regimens. However, this guideline recommends 9–12 months of treatment for TB meningitis and other serious forms of EPTB given the serious risk of disability and mortality, and 9 months of treatment for TB of bones or joints because of the difficulties of assessing treatment response.

Provider-initiated HIV testing is recommended as part of the evaluation of all TB patients and patients in whom the disease is suspected. HIV testing is especially important in persons with or with presumptive EPTB because of the increased frequency of extra-pulmonary involvement in persons with immunosuppression. Extra-pulmonary TB is considered to be WHO clinical stage 4 HIV disease.

Unless drug resistance is suspected, adjuvant corticosteroid treatment is recommended for TB meningitis and pericarditis. Surgical intervention may be required in diagnosis of some EPTB, but mainly indicated in the management of late complications of disease such as hydrocephalus, obstructive uropathy, constrictive pericarditis and neurological involvement from Pott’s disease (spinal TB).

For large, fluctuant lymph nodes that appear to be about to drain spontaneously, aspiration or incision and drainage is beneficial.

#### Treatment of TB meningitis

### Important drug to drug interactions

Many TB patients have concomitant illnesses. At the start of TB treatment, all patients should be asked about medicines they are currently taking.

The most important interactions with anti-TB drugs are due to rifampicin. Rifampicin induces pathways that metabolize other drugs, thereby reducing the concentration and effect of those drugs. To maintain a therapeutic effect, dosages of the other drug(s) may need to be increased. When rifampicin is discontinued, its metabolism-inducing effect resolves within about 2 weeks.

Rifampicin substantially reduces the concentration and effect of the following drugs:

- **anti-infectives**(including certain antiretroviral drugs), mefloquine, azole antifungal agents, clarithromycin, erythromycin, doxycycline, atovaquone, chloramphenicol);
- **hormone therapy,** including ethinylestradiol, norethindrone, tamoxifen, levothyroxine (Rifampicin interacts with oral contraceptive medications leading to lowered protective efficacy. A woman receiving oral contraception may choose between two options while receiving treatment with rifampicin: following consultation with a clinician, an oral contraceptive pill containing a higher estrogen dose (50 μg), or another form of contraception); methadone; warfarin; cyclosporin; corticosteroids; anticonvulsants (including phenytoin);
- **cardiovascular agents** including digoxin (among patients with renal insufficiency), digitoxin, verapamil, nifedipine, diltiazem, propranolol, metoprorol, enalapril, losartan, quinidine, mexiletine, tocainide, propafenone; theophylline; sulfonylurea hypoglycaemics; hypolipidaemics including simvastatin and fluvastatin; nortriptyline, haloperidol, quetiapine, benzodiazepines (including diazepam, triazolam), zolpidem, buspirone.

### TB Treatment regimens in special circumstances

#### Treatment for pregnant women

The benefit of treating an active TB disease in a pregnant woman far outweighs the risks that the drugs may pose to both the mother and the foetus. Most TB drugs are safe for use in pregnant women withthe exception of**streptomycin** which is ototoxic to the foetus and should therefore not be used in pregnancy.Every woman of child bearing age diagnosed with TB shouldbe asked of pregnancy status before starting TB treatment.

#### Treatment for breastfeeding women

A woman who is breastfeeding and has TB should receive a full course of TB treatment. Timely and properly applied chemotherapy is the best way to prevent transmission of tubercle bacilli to the baby. ***All the TB drugs are compatible with breastfeeding and a woman taking them can safely continue to breastfeed her baby***. The mother and baby should stay together and the baby should continue to breastfeed in the normal way, but be given prophylactic isoniazid for at least six months (Isoniazid 10mg/ kg). BCG vaccination of the newborn should be postponed until the end of isoniazid prophylaxis.

Pyridoxine supplementation is recommended for all pregnant or breastfeeding women taking isoniazid.

#### Treatment for women taking the oral contraceptive pill

Rifampicin interacts with the contraceptive pill with a risk of decreased protective efficacy against pregnancy. A woman who is receiving contraception may choose between the following two options while receiving treatment with rifampicin. Following consultation with a physician, she could take an oral contraceptive pill containing a higher dose of oestrogen*(50* mcg), alternatively she could use another form of contraception.

#### Treatment for patients with liver disorders

Isoniazid, rifampicin and pyrazinamide are all associated with hepatitis. Of the three, rifampicin is least likely to cause hepatocellular damage, although the drug is associated with cholestatic jaundice, pyrazinamide is the most hepatotoxic. The patients with the following conditions can receive the usual short-course chemotherapy regimen provided that there is no clinical evidence of chronic liver disease: hepatitis virus carriage, a past history of acute hepatitis, excessive alcohol consumption. However, hepatotoxic reactions to TB drugs may be more common in these patients and should be anticipated.

Possible regimens include:

1. Two hepatotoxic drugs (rather than the three in the standard regimen):

- 9 months of isoniazid and rifampicin, plus ethambutol (until or unless isoniazid susceptibility is documented);
- 2 months of isoniazid, rifampicin, streptomycin and ethambutol, followed by 6 months of isoniazid and rifampicin;
- 6–9 months of rifampicin, pyrazinamide and ethambutol.

1. One hepatotoxic drug:
   - 2 months of isoniazid, ethambutol and streptomycin, followed by 10 months of isoniazid and ethambutol.
2. No hepatotoxic drugs:
   - 18–24 months of streptomycin, ethambutol and a fluoroquinolone.

Expert consultation is advisable in treating patients with advanced or unstable liver disease.

Clinical monitoring (and liver function tests, if possible) of all patients with pre-existing liver disease should be performed during treatment.

#### Treatment of patients with renal failure

Isoniazid, rifampicin and pyrazinamide are either eliminated almost entirely by biliary excretion or metabolized into non-toxic compounds. These drugs can, therefore, be given in normal dosage to patients with renal failure. In severe renal failure, patients should receive pyridoxine with isoniazid in order to prevent peripheral neuropathy. Streptomycin and ethambutol are excreted by the kidney. Where facilities are available to monitor renal function closely it may be possible to give streptomycin and ethambutol in reduced doses. The safest regimen to be administered in patients with renal failure is 2 HRZ/ 4 HR. All patients that fall under the category “special circumstances” should be referred to and managed by to a specialist.

## The Role of Adjuvant Steroid Treatment

Adjuvant steroid treatment is steroid treatment given in addition to anti-TB drug treatment. Studies in the pre-HIV era confirmed the benefit of steroids for TB meningitis and pleural and pericardial TB. Steroids arealso of benefit in HIV-positive patients with pericardial TB.

**Adjuvant steroid therapy is recommended in the following conditions:**

- TB meningitis (decreased consciousness, neurological defects, or spinal block).
- TB pericarditis (with effusion or constriction).
- TB pleural effusion (when large with severe symptoms).
- Hypoadrenalism (TB of adrenal glands).
- TB laryngitis (with life-threatening airway obstruction).
- Severe hypersensitivity reactions to anti-TB drugs.
- Renal tract TB (to prevent ureteric scarring).
- Massive lymph node enlargement with pressure effects.

Rifampicin being a potent inducer of hepatic enzymes that metabolize steroids,the effective dose of prednisolone is half the prescribed treatment dose given to the patient. The suggested treatment doses of prednisolone depending on the condition is as follows:

Table 4.5: Prednisolone indication and recommended doses in TB management

| **Indication** | **Prednisolone treatment**  **(dose for children in brackets)** |
| --- | --- |
| **TB meningitis** | 60 mg (1–2 mg/kg) daily for weeks 1–4,  then decrease over several weeks |
| **TB pericarditis** | 60 mg (1–2 mg/kg) daily for weeks 1–4  30 mg (0.5–1 mg/kg) daily for weeks 5–8, then  decrease over several weeks |
| **TB pleural effusion** | 30 mg (0.5–1 mg/kg) daily for 1–2 weeks |

*Source: TB/HIV. A Clinical Manual WHO/HTM/TB/2006.420*

Steroids are immunosuppressants. Steroids may further depress immunity and increase risk of opportunistic infections in HIV-positive patients. However, on balance, TB/HIV patients are still likely to benefit from the use of steroids in the presence of the above conditions.

1. PATIENT SUPPORT AND DOT PROVISION

## Importance of patient support

The public health priority for TB control is to cure the patients and ultimately interrupt transmission of the disease within communities. A patient-centred approach to Directly observed therapy (DOT), and measures to prevent interruption of treatment that are important to support patients on treatment are critical to ensure adherence to the prescribed anti-TB treatment regimen.

Adherence to TB treatment is crucial to achieving cure while avoiding the emergence of drug resistance. Regular and complete intake of medications gives the individual TB patients the best chance of being cured; and results in the reduction of spread of TB within the community.

The emergence and spread of MDR- and XDR-TB further reinforces the absolute necessity of supporting TB patients to not miss any drug doses. Standardized treatment including patient support remains a critical core element of high quality DOTS enhancement in the Stop TB Strategy to achieve the treatment success target of 85%.

In addition to supervised treatment (or DOTS), measures to support patient adherence to regular and complete treatment include:

- A regular supply of drugs
- Accessible, high-quality, continuous ambulatory TB cares
- Positive action to remove barriers to treatment and care
- Availability of hospitalization

Hospitalization is essential for severely ill patients and for those with complications or associated conditions requiring closer clinical monitoring

## Community Based DOTS (CB DOTS)

Patients should have the option to come to the health facility for their daily DOT or they can take their treatment at home with a treatment supporter of their own choice (community-based DOTS).

The patient and the treatment supporter needs to keep records of daily intake of medications to ensure that treatment is taken as prescribed.

### Identification of treatment supporter

The first step is to assist the patient in identifying a treatment supporter that is acceptable to him or her. For TB patients who live close to a health facility, the treatment supporter will be one of the staff in the health facility, and this is the ideal choice if convenient to the patient.

For TB patients who live in a distant location from a health facility, the treatment observer could be a community health worker or a trained and supervised local community member.

ART community care providers can observe TB treatment.

The treatment supporters should receive training according to the NTCP protocols.

The treatment supporter should:

- Remind and watch the patient take their drugs everyday
- Mark the Identity card after the drugs are taken
- Collect the drugs every week from the health facility
- Inform the health worker of any problems encountered
- Accompany the patient to the health facility when needed

### Orientation of treatment supporters

- Carefully explain the tasks above to the supporter.
- Check that the supporter can carry out the tasks.
- Demonstrate how to provide DOT and how to mark the drug intake.
- Explain possible side-effect and what needs to be done.
- Make sure that the supporter fully understand the tasks.

### Provide enough drugs to last until the next visit.

- Cut up the blister pack to prepare daily blister strips, which contain the exact number of tablets that a patient needs to take each day.
- Explain how many tablets the patient should take each day.
- Agree on the date of the next visit and note this on the Identity and treatment cards.
- Ask patients to bring back the empty blister packs and Identity card during each visit to the health facility.
- Put an ‘X’on the collection date and fill the doses on the treatment card (White Card)

### Keep regular contact with the patient and supporter

- Ask the supporter and patient to return every week to collect more drugs in the intensive phase and every two weeks in the continuation phase.

### What to do during the visit of the supporter / patient to health facility?

- Take the time to talk to the supporter and patient on each visit.
- Help them to resolve any problems that they have encountered
- Check the daily treatment record when re-supplying drugs, and transfer information from the treatment supporter card (Yellow card) to the patient’s treatment card (White Card)
- Check that the TB Identity Card record corresponds to the empty blister packs.
- Discuss any problems in filling out the treatment record.
- Check and put **“0”** on the TB treatment card where patient did not swallow the drugs;
- Provide the supporter with enough drugs until the next scheduled visit

## Prevention of treatment interruption

Promoting adherence through a patient-centred approach is probably more effective in preventing treatment interruption than devoting resources to tracing patients who are lost to follow up.

Whenever the patient visits the health facility, the need for regular and complete intake of treatment should be reinforced and any problems that may cause interruption should be identified.

At registration, sufficient time should be set aside for meeting with the patient (and preferably also the patient’s family members or a designated treatment supporter). This initial meeting provides an important opportunity to inform the patient about the duration of treatment. During the meeting, it is vital to record the patient’s address and other relevant addresses (e.g. partner or spouse, parents, place of work or study, or private doctor who may be consulted) as well as explain the need to consult ahead of time in case of a change of address. This maximizes the likelihood of locating patients who interrupt treatment. Recording mobile telephone numbers for the patient and family has proved valuable in many settings.

### Role of Adherence Officers

The Adherence Officers have a special role in providing support in ensuring continuation of treatment for all patients who happen to interrupt treatment. In such situation, the Clinic staff or treatment supporter should liaise with the Adherence Officer in order to trace the patient and encourage him/her to resume treatment.

## Nutritional support to TB patients

Although conclusive evidence that nutritional support reduces mortality is lacking, it is known to speed client’s recovery of health after initiating ART and or TB treatment and to improve the nutritional health of the client.

### Food by prescription

This is a joint initiative of the NTCP, the Swaziland Nutritional Council, WFP, WHO and other partners to provide food by prescription to TB clients.

The food by prescription programme was necessitated by the nutritional challenges resulting from the high HIV and TB prevalence. It seeks to improve treatment adherence, contribute to meeting the body’s increased demand for nutrients and compensate for the deleterious effects of diseases and medication on nutrient absorption.

The overall objectives of the food by nutrition includes:

- Increase adherence to treatment of malnourished members of the target populations.
- Improve the nutritional status of malnourished members of the target populations and their families.

**NOTE: Acutely malnourished children should be referred to the nearest IMAM site**

Admission criteria for food by prescription vary based on the treatment programme which refers the client, according to:

Admission criteria:

- Adults on ART and or TB Treatment:
  - BMI≤ 18.5 or
  - MUAC≤ 23cm or
  - Loss of weight/BMI of ≥ 10% in the previous month.

The criteria used for admission is due to the fact that BMI and MUAC do not identify a 100% congruent set of clients as malnourished. It could easily happen that a client is malnourished according to one criterion but not according to another.

All clients who come to participating health facilities will be screened for malnutrition according to the standard procedure (See below).

If found to be malnourished, they will be eligible to receive the services described in stage three and four below.

### Food Prescription Initiation procedure

**Stage 1: screen**

1. During the physical examination, the appropriate person (nurse or expert client, depending on procedure at the facility) should measure the client’s weight, height and MUAC. This person should also find the client’s BMI chart.If the client is an adult ART client, adult TB client or adult Maternal and Child Health Nutrition client, the clinician should admit them if any or all of the following three conditions apply
2. Their BMI is less than or equal to 18.5
3. Their MUAC is less than or equal to 23
4. They have lost 10% or more of their weight (or BMI) in the previous month.
5. If the client meets any of the malnutrition criteria above, proceed to stage 2.

**Step 2: Admit**

1. The examining clinician should fill in the client card carefully and give it to the client.

**Stage 3: provide nutritional treatment**

1. Each client should also be registered in the registration book in the food room. This is important because it enables the client’s admission and exit status to be tracked, and allows for home visits by the food by prescription organizations. **The client must sign against the NUMBER OF KILOGRAMS received**. It is not sufficient to say simply “one family ration” or “one individual ration”

**Stage 4: health education and follow up**

1. Be sure the client understands the roles of the foods they are receiving (the individual ration is their medicine, and the family ration is the food security of their family), the goal of the programme, and the reasons for admission. Provide the client with an information pamphlet about the programme for them to take home. Inform the local treatment supporters of the client’s enrolment and ask them to follow up. This should be encouraged most strongly if the client is malnourished.

**Stage 5: Make next appointment**

1. Before the client returns home, the next appointment should be emphasized (the same date as the next monthly ART and or TB review). The importance of adherence should be stressed, and clients who are admitted should receive a note in their files as well. This note will guard against the danger of clients losing the card and against the possibility that the client may forget to take their food.
2. **Discharge procedures:**

The procedure for discharging a client as cured starts in the month before the actual discharge. If the client’s anthropometrics are above the programme admission criteria for the first month, they should be informed that their condition has improved significantly. The client should receive food for this month, but must be informed that after the next month, food supplementation will cease.

**Lost to follow up:**

A client who has missed two consecutive monthly visits is considered in . The following procedure should be used if a Food by prescription client s:

1. Standard procedure for follow up from community health workers should be followed, according to the procedure of the unit initiating the client. If the client cannot be found or refuses to return after counseling, proceed to step 2.
2. Any client who is declared Lost to follow up under the criteria of the unit referring them to Food by prescription should also be discharged from Food by description. “Lost to follow up” entry for the client should be recorded in the “discharge status” field of the registration book entry for the client.
3. If a client is discharged as a er returns to the health facility in the future and is eligible to be initiated on food by prescription again, he/she may rejoin the programme.

**Death:**

If a client passes on while enrolled on the programme, the client should be removed from the register of beneficiaries on account of death.

1. MONITORING TUBERCULOSIS TREATMENT

## Basis for monitoring TB treatment

Monitoring patient’s clinical as well as bacteriological response to anti-TB therapy is an essential element of TB treatment and care. Regular monitoring of patients also facilitates treatment completion and allows the identification and management of adverse drug reactions.

All patients on anti-tuberculosis treatment should be monitored systematically throughout the duration of treatment. Patients, their treatment supporters and health workers should be instructed to report the persistence or reappearance of symptoms of TB (including weight loss), symptoms of adverse drug reactions, or treatment interruptions.

### Clinical monitoring:

Monitoring the improvement in the patients’ clinical state provides a guide to treatment response. During regular follow up visits, clinical assessment in the form of focused history and physical examination should be conducted and the results documented in the patient’s treatment card.

Smear-negative pulmonary TB patients or those enrolled without sputum result, whose sputum smears are negative at end of 2 months intensive phase need no further sputum monitoring. They should be monitored clinically; and their body weight should be taken and recorded monthly.

Evidence of clinical improvement includes reduction or disappearance of symptoms including cough, fever, tiredness and weight gain. A sudden unexplained drop in the patient’s weight should be investigated by the clinician.

#### Monitoring Extra-pulmonary TB treatment

Response to treatment is usually monitored clinically and depending on the organ affected, radiology may play an important role. As in pulmonary smear-negative disease, the weight of the patient is also a useful indicator in monitoring clinical response in extra-pulmonary disease.

### Bacteriological monitoring:

Patients with sputum smear-positive PTB or positive Xpert MTB/Rif tests should be monitored by sputum smear examination. These are usually adults and sometimes older children.

Xpert MTB/Rif test should not be requested for the purpose of monitoring patients on treatment.

Routine monitoring of treatment response by CXR is also not recommended.

Serial sputum smear examinations should be performed at the recommended intervals to verify the effectiveness of the treatment in killing the bacilli.

Two sputum sample should be obtained from the patient for examination at the end of the second and fifth month and at the end of treatment for all sputum smear positive TB patients. The sample should be collected as ‘**spot or early morning samples’.**

- Sputum specimens should be collected without interrupting treatment and transported to the laboratory as soon as possible thereafter;
- In the event of an unavoidable delay, specimens should be refrigerated or kept in as cool a place as possible.

Table 5.1: Recommended Schedule for follow up sputum examinations for PTB patients

| **When to monitor** | **Regimen for New TB cases**  **6-month treatment regimen** | **Regimen for Previously treated**  **Cases (8-month Regimen)** |
| --- | --- | --- |
| **At time of diagnosis** | sputum smear microscopy or  Xpert MTB/Rif test | **sputum smear microscopy or**  **Xpert MTB/Rif test** |
| **At end of initial phase** | sputum smear (end month 2) | **sputum smear (end month 3)** |
| **In continuation phase** | sputum smear  (end month 5) | **sputum smear**  **(end month 5)** |
| **During last month of treatment** | **sputum smear**  **(end month 6)** | **sputum smear**  **(end month 8)** |

*Source: Treatment of tuberculosis guidelines. 4^th^Edition. WHO/HTM/TB/2009.420*

#### New sputum smear-positive pulmonary TB patients

Follow up sputum smears should be performed at the end of the second and fifth months, and in the last month of treatment.

- If the sputum smear result is positive at the end of the second (2^nd^) month, a DST should be performed immediately, and treatment should be modified based on the results. A rapid DST method e.g the LPA should be used.
- The intensive phase should be extended by up to one (1) month while results of DST is being awaited for further treatment decisions.
- If the sputum smear result is still positive at the end of the fifth (5^th^) month, this constitutes treatment failure. The treatment should be discontinued, and the patient’s sputum sample should be obtained for culture and drug susceptibility testing. A rapid DST method e.g the LPA should be used, and full DST requested immediately.

#### Previously treated pulmonary sputum smear-positive patients

Sputum smear examination is performed at the end of the initial phase of treatment (at the end of the third month), at the end of the fifth month; and at the end of treatment.

If the patient is sputum smear-positive at the end of the third month, the initial phase of treatment with 4 drugs, a rapid DST method e.g the LPA should be used, and full DST should be requested immediately. Treatment should be modified based on the DST results.

The intensive phase should be extended by up to one (1) month while results of DST is being awaited for further treatment decisions.

Positive smears at the end of the fifth (5^th^) month indicate failure of treatment and, the patient should have their sputum examined by culture and DST. Treatment should be guided by the results of DST.

#### New sputum smear-negative pulmonary TB patients

Sputum smear-negative patients should be monitored clinically; body weight is a useful progress indicator.

Sputum smears should be checked at the end of the second month in case of the following possibilities:

- disease progress due to non-adherence to treatment, or
- an error at the time of initial diagnosis (i.e. a true smear-positive patient misdiagnosed as smear-negative) plus
- drug resistance.

A patient initially diagnosed as sputum smear-negative and becoming positive at the second month should be investigated for MDR-TB using the rapid DST methods e.g LPA. A full conventional DST should also be requested.

- In case of any MDR result, the treatment is declared “failure” and the patient is referred to DR unit.
- In case of any positive smear result during the treatment, follow the steps described in the box above.

Table 6: Sputum follow-up algorithm for patients on anti-TB treatment

Pulmonary TB patient

**(+)**

**(-)**

**(-)**

- Continue treatment
- Collect sputum at end of 5 months

**(+)**

- Declare treatment failed
- Collect sputum for culture/DST

**(-)**

- Continue treatment
- Collect sputum at end of 6 months

**(-)**

**(+)**

- Declare treatment failed
- Collect sputum for culture/DST

Smear negative or Xpert MTB-/Rif- but clinically diagnosed

Smear positive

or Xpert MTB +/Rif -

- Send sample to LPA
- Start 2HREZ/4HR
- Collect sputum at end of 2 months
- Start 2HREZ/4HR
- Collect sputum at end of 2 months

Continue treatment till end of 6 months

- Collect sputum for culture/ FLD DST
- Continue the treatment of RHEZ for one month then continue with 3RHE or as directed by DST results

Declare cured

## Management of treatment interruption

When a patient misses an arranged appointment to receive treatment, such a patient should be contacted immediately to ensure that the treatment can be continued.

The NTCP Adherence Officer should ensure that the patient is contacted within a day after missing treatment during the initial phase, and within a week during the continuation phase.

The patient can be traced using the locating information previously obtained. It is important to find out the cause of the patient’s absence so that appropriate action can be taken and treatment can continue.

The management of patients who have interrupted treatment takes into consideration several factors, such as the time at which the treatment was interrupted, the length of treatment interruption, the smear and LPA/Gene Xpert status after return, as shown in Table 26.

Culture and DST should be performed upon return of patients who interrupted treatment for more than 2 consecutive weeks.

A simple decision matrix is suggested in Table below:

Table 8.1: Management of TB treatment interruption

| **Length of treatment** | **Length of**  **interruption** | **Sputum status upon return** | **Actions to be taken** | | |
| --- | --- | --- | --- | --- | --- |
|  |  |  | **Treatment**  **outcome** | **DST** | **Registration and further**  **treatment** |
| < 1  Month | <2 weeks |  |  |  | Continue treatment started at the point it was stopped |
|  | 2-7 weeks |  |  |  | - Restart treatment without new registration |
|  | > 7 weeks |  | er | Perform (Gene Xpert or LPA)  **AND** culture/DST by MGIT | - If no resistance, register again as new case of TB and start Cat I |
| > 1  Months | <2 weeks |  |  |  | - Continue treatment started at the point it was stopped |
|  | 2-7 weeks | (-) |  |  | - Continue treatment started, at the point it was stopped |
|  |  | (+) |  | Perform (Gene Xpert or LPA)  **AND**  culture/DST by MGIT | - If no resistance, continue treatment started |
|  | > 7 weeks |  | er |  | - If no resistance, register again as Treatment after er and start treatment for cases previously treated with FLD |

*Source: Adapted from Treatment of tuberculosis guidelines. 4^th^Edition. WHO/HTM/TB/2009.420*

- In any case of drug resistance detected by LPA or Gene-Xpert, refer the patient to DR treatment unit.

### Monitoring of TB Patients for Adverse Effects of Anti-TB Drugs

Most TB patients complete their treatment without any significant adverse effects of drugs. However, a few patients do experience adverse effects. It is therefore important that patients be clinically monitored during treatment so that adverse effects can be detected promptly and managed properly. Routine laboratory monitoring is not necessary.

Health personnel can monitor adverse effects of drugs by teaching patients how to recognize symptoms of common adverse effects and to report if they develop such symptoms, and by asking about symptoms when patients report to collect drugs or on follow-up visit.

#### Prevention of adverse effects of drugs

Health personnel can prevent some drug-induced side-effects, for example Isoniazid-induced peripheral neuropathy. This usually presents as a numbness, tingling or burning sensation of the feet and occurs more commonly in pregnant women and in people with the following conditions: HIV infection, alcoholabuse, malnutrition, diabetes, chronic liver disease. These patients should receive preventive treatment with pyridoxine, 10 mg daily, along with their anti-tuberculosis drugs.

#### Adverse effects of anti-tuberculosis drugs

Adverse effects associated with anti-TB are classified as minor or major. In general, a patient who develops minor adverse effects should continue the TB treatment, sometimes at a reduced dose. The patient also receives symptomatic treatment. If a patient develops a major side-effect, the treatment or the offending drug is stopped. Further management depends on the nature of the adverse reaction. Patients with major adverse reactions should be managed in a hospital. Table below provides a symptom-based approach to the management of adverse effects.

#### Symptom-based approach to management of drug side-effects

Table 8.2: Guide to Management of side effects of first-line anti-TB drugs

| Side effects | Drugs (probably responsible) | Management |
| --- | --- | --- |
| Major |  |  |
| Skin rash with or without itching | Streptomycin, Isoniazid, Rifampicin, Pyrazinamide. | Stop anti-TB drugs |
| Deafness (no wax on otoscopy) | Streptomycin | Stop Streptomycin |
| Dizziness (vertigo and nystagmus) | Streptomycin | Stop Streptomycin |
| Jaundice (other causes excluded), hepatitis | INH, Pyrazinamide, Rifampicin | Stop anti-TB drugs |
| Confusion (suspect drug induced acute liver failure if there is jaundice) | Most anti-TB drugs | Stop anti-TB drugs |
| Visual impairment (other causes excluded) | Ethambutol | Stop Ethambutol |
| Shock, purpura, acute renal failure | Rifampicin | Stop Rifampicin |
| Decreased urine output | Streptomycin | Stop Streptomycin |
| Minor |  |  |
| Anorexia, nausea, abdominal pain | Pyrazinamide, rifampicin, INH | Give drugs with small meals or just before bedtime, and advise patient to swallow pills slowly with small sips of water. If the symptoms persist or worsen, or there is protracted vomiting or any sign of bleeding, consider the side-effect to be major and refer to the Clinician urgently. |
| Joint pains | Pyrazinamide | Aspirin or other non-steroidal anti-inflammatory drugs or Paracetamol. |
| Burning, numbness or tingling sensation in the hands or feet | INH | Give Pyridoxine 50-75mg daily |
| Drowsiness | INH | Reassure the patient. Give drugs before bedtime. |
| Orange/red urine | Rifampicin | Reassure patient. Patients should be informed about the urine change when initiating TB treatment. |
| Flu-like syndrome (fever, chills, malaise, headache, bone pain) | Intermittent dosing of Rifampicin | Reassure patient. When staring TB treatment, patients should be informed about the possibility of such occurring and that it is normal. |

*Source: Treatment of tuberculosis guidelines. 4^th^Edition. WHO/HTM/TB/2009.420*

Note that first line drugs cannot be substituted with any secondline drug or any other in the event of adverse reaction management.

Table 8.3: When to stop anti-TB drugs

| **Reaction** | **Drug Responsible** |
| --- | --- |
| **Hearing loss or disturbed balance** | Streptomycin |
| **Visual disturbance (poor vision and colour perception)** | Ethambutol |
| **Renal failure, shock, or thrombocytopenia** | Rifampicin |
| **Hepatitis** | Pyrazinamide |

#### Management of skin itching and rash

In case of skin itching, it is necessary to determine if the reaction was present before initiation of anti-TB treatment, as many HIV-positive patients have itchy skin lesions as a result of HIV infection.

Other causes of itching should also be excluded, give antihistamines, continue anti-TB treatment and observe closely. In the event that rash develops, anti-TB drugs should be stopped until the rash resolves. In case of severe reaction, supportive treatment should be provided as appropriate.

#### Reintroduction of anti-TB drugs following drug reaction

Drug challenge should be done to identify the drug responsible for the reaction. The process should start with the anti-TB drug least likely to be responsible for the reaction (i.e.isoniazid). The initial challenge should start with a small dose of the drug. If a reaction occurs to a small challenge dose, it will not be such a severe reaction as to a full dose. Gradually increase the dose over 3 days. Repeat the procedure, adding in one drug at a time. A reaction after a particular drug is added identifies that drug as the one responsible for the reaction.

Table 7.4: Guide to performing anti-TB drug challenge and re-introduction

| **Drug** | **Likelihood of causing a reaction** | Challenge doses | | |
| --- | --- | --- | --- | --- |
|  |  | **Day 1** | **Day 2** | **Day 3** |
| **Isoniazid** | Least likely | 50 mg | 300 mg | 300 mg |
| **Rifampicin** |  | 75 mg | 300 mg | Full dose |
| **Pyrazinamide** |  | 250 mg | 1 gr | Full dose |
| **Ethambutol** |  | 100 mg | 500 mg | Full dose |
| **Streptomycin** | Most likely | 125 mg | 500 mg | Full dose |

*Source: TB/HIV. A Clinical Manual WHO/HTM/TB/2006.*

If the drug responsible for the reaction is pyrazinamide, ethambutol, orstreptomycin, resume anti-TB treatment without the offending drug. If possible, replace it with another drug. It may be necessary to extend the treatment regimen. Consider the start of the resumed regimen as a new start of treatment. This prolongs the total time of TB treatment, but decreases the risk of recurrence.

## Determining TB Treatment Outcomes

. The table below (Table 7.3) shows the definitions of standardized treatment outcomes.

Table 8.5: Definitions of TB treatment outcomes

| **Outcome** | **Definition ^a^** |
| --- | --- |
| **Cured** | A **bacteriologically-confirmed** case, who is smear or culture-negative in the last month of treatment and on at least one previous occasion |
| **Treatment completed** | A patient who completed treatment but who does not have a negative sputum smear or culture result in the last month of treatment and in at least one previous occasion ^b^ |
| **Treatment failure** | - A patient whose sputum smear or culture is positive at 5 months or later during treatment. - Patients found to harbour a multidrug-resistant (MDR) strain at any time during the treatment, whether they are smear-negative or –positive. - Smear negative or extra-pulmonary patients with clinical condition not improving or worsening (clinically judged) |
| **Died** | A patient who dies for any reason during the course of treatment |
| **Lost to follow up** | A patient whose treatment was interrupted for 2 consecutive months or more |
| **Not evaluated** | Patient for whom no treatment outcome is assigned  Include former "transfer-out" in this category |
| **Treatment success ^c^** | Sum of cured and completed treatment |
| (a) These definitions apply to pulmonary smear-positive and smear-negative patients, and to patients with extra-pulmonary disease.  (b) The sputum examination may not have been done or the results may not be available  (c) For smear or culture positive patients only | |

*Source: WHO STAG 2012 recommendations.*

1. MANAGEMENT OF CHILDHOOD TUBERCULOSIS

## Importance of childhood TB

Childhood TB accounts for almost 15-20% of all TB cases. It represents active TB transmission in the community, mainly from adult sources. Usually children become infected with TB after household exposure to a sputum positive adult or adolescent, although smear negative cases can also transmit TB. Children (especially <10 years of age) rarely develop lung cavities and they are therefore less likely to transmit the TB organism. However, the absence of a potential source case does not exclude childhood TB especially in a high prevalence setting like Swaziland as infections may occur outside the household. This, usually coupled with inadequate capacity for effective paediatric TB diagnosis, often results in under-detection and treatment of childhood TB, leading to high morbidity and mortality.

These guidelines outlines:

- the approach to diagnosis of paediatric TB
- TB treatment of childhood TB
- diagnosis and treatment of resistant TB in children

## Approach to diagnosis of TB in children

Diagnosis of active TB disease in children is often difficult, and should be made based on careful and thorough assessment of all the findings from a careful history, clinical examination and relevant investigations, e.g. TST, chest X-ray (CXR), Gene Xpert and sputum smear microscopy. As most children with TB have pulmonary TB, bacteriological confirmation should be sought whenever possible.

Any child presenting with symptoms and signs suggestive of TB should be referred to a Medical Officer for further evaluation; once the decision to start TB treatment has been made, the child should be treated with a full course of therapy. Diagnosis can be done at any level of care, even when X-Rays are not available.

However, a trial of treatment with anti-TB medications is not recommended as a method to diagnose TB in children.

**Clinical symptoms and a positive TB contact can be enough to start TB treatment.**

The key risk factors for TB include:

- household contact with a newly diagnosed smear-positive case
- age less than 5 years
- HIV infection
- severe malnutrition.

The key features suggestive of TB are:

- chronic symptoms suggestive of TB
- physical signs highly of suggestive of TB
- a positive tuberculin skin test
- chest X-ray suggestive of TB
- failure to thrive

The approach to diagnose TB in children follows the usual standard protocol in clinical practice. This includes:

- Careful history (including history of TB contact and symptoms consistent with TB)
- Clinical examination (including growth assessment)
- Tuberculin skin testing
- Bacteriological confirmation whenever possible
- Investigations relevant for establishing the diagnosis of pulmonary TB and extra-pulmonary TB
- HIV testing (in high HIV prevalence areas)

**Note:**

A child should start TB treatment when there are TB symptoms not responding to adequate antibiotic therapy, even in the absence of a CXR.

This is especially important in infants and young children, as symptoms are less specific and the mortality is higher.

### Evaluation for paediatric TB disease

The clinician should ensure that careful history is taken including history of TB contact and symptoms consistent with TB.

***a. Contact***

This refers to a child living in the same household as or in frequent contact with a source case

(e.g. the child’s caregiver) with sputum smear-positive pulmonary TB or sputum

smear-negative but culture-positive TB.

The following actions are key to trace TB contacts in children.

- All children aged 0–4 years and children aged 5 years and above who are symptomatic, who have been in close contact with a smear-positive TB case, must be screened for TB.
- Effort should be made to detect the source case (usually an adult with sputum smear-positive pulmonary TB) and any other undiagnosed cases in the household when any child (aged less than 15 years) is diagnosed with TB.
- If a child presents with infectious TB, child contacts must be sought and screened, as for any smear-positive source case. Children should be regarded as infectious if they have sputum smear-positive pulmonary TB or cavitary TB on CXR.

***b. Symptomatic children***

Children with symptomatic TB often have already developed chronic disease. The commonest symptoms to be considered are as follows:

Clinical symptoms:

- Persistent and unremitting cough (with or without previous antibiotic treatments).
- Weight loss or failure to thrive (absence of appropriate weight gain for infants). Malnutrition can be the first sign of TB in children, especially if there is poor response after 1 week of nutritional support.
- Persistent fever
- Drenching night sweats
- Fatigue, reduced playfulness, decreased activity.
- Enlarged liver and spleen (disseminated TB).
- Enlarged LN.

A child should start TB treatment when there are TB symptoms not responding to adequate antibiotic therapy, even in the absence of a CXR. This is especially important in infants and young children, as symptoms are less specific and the mortality is higher.

### Clinical examination (including growth assessment)

There are no specific features on clinical examination that are typical of TB in children or can confirm that the presenting illness is due to pulmonary TB. Some signs, although uncommon, are highly suggestive of extra-pulmonary TB (i.e. TB of organs other than the lungs). Other signs are common and should prompt an investigation into the possibility of childhood TB. Important physical signs are:

*a. physical signs highly suggestive of extra-pulmonary TB:*

- gibbus, especially of recent onset (resulting from vertebral TB)
- non-painful enlarged cervical lymphadenopathy with fistula formation;

*b. physical signs requiring investigation to exclude extra-pulmonary TB:*

- meningitis not responding to antibiotic treatment, with a sub-acute onset or raised intracranial pressure
- pleural effusion
- pericardial effusion
- distended abdomen with ascites
- non-painful enlarged lymph nodes without fistula formation
- non-painful enlarged joint
- signs of tuberculin hypersensitivity (e.g. phlyctenular conjunctivitis, erythema nodosum).

**Note:** Documented weight loss or failure to gain weight, especially after being treated in a nutritional rehabilitation programme, is a good indicator of chronic disease in children, of which TB may be the cause.

## Special Investigations for paediatric TB

### Chest X-ray

Chest X-ray in children is a useful tool to assist in the diagnosis. Lateral X-rays should be routinely done in children.

The most common signs are:

- Enlarged lymph nodes in the hilar region or compression of the airway.
- Lateral X-rays can identify lymph nodes in the hilar region or posterior pneumonias.
- Opacification of the lung tissue, with or without cavities.
- Miliary pattern.
- Pleural effusion in older children.

***False negatives:***

**A normal Chest X-Ray in a child does not rule out active TB disease:** this is a very frequent presentation in HIV positive children with a low CD4 count and/or malnourished children.

***False positives:***

HIV-infected children can present with lymphoid interstitial pneumonia (LIP) and bronchiectasias, which can mimic TB in an X-ray.

### Role of a Tuberculin Skin Test

The tuberculin skin test (TST) is a tool for detection of latent TB infection (LTBI). The test involves intradermal injection of purified protein derivative (PPD), a crude mixture of mycobacterial antigens, which stimulates a delayed type hypersensitivity response and causes induration at the injection site within 48 to 72 hours.

However the **TST detects only infection with MTB, not necessarily active disease** (See Annex ??).

- A positive test indicates infection with TB, but not necessarily TB disease.
- In a child under 5 years, a strongly positive skin test indicates recent (6 weeks or more) infection that is a risk factor for progression to disease. In the presence of other features, i.e. history of TB contact, signs and symptoms of TB and x-ray changes, a positive tuberculin skin test is suggestive of TB disease in children.

A positive reaction occurs after previous BCG immunization and should remain positive for several years thereafter. This reaction is usually weaker than the reaction to natural infection with *M. tuberculosis*. A positive reaction is only one piece of evidence in favor of the diagnosis in children.

A negative tuberculin skin test does not exclude TB. Various conditions may cause a negative reaction even if a child has TB.

If the chest x-ray is suspicious of TB and the skin test is negative, TB can be diagnosed in children. Conditions that may suppress the tuberculin skin test and give a false negative result include: HIV infection, malnutrition, severe viral infections (e.g. measles, chicken pox), cancer, immuno-suppressive drugs (e.g. steroids), severe disseminated TB.

A positive result is 5mm of induration in HIV positive children or 10mm of induration in HIV negative children.

False negative results can be found in severely malnourished children, those with low CD4 count, in meningitis or miliary TB and in cases of recent TB infection (less than 10 weeks).

False positive results can be found in mycobacteria other than tuberculosis (MOTT), BCG vaccination and due to an incorrect interpretation of the TST.

TST should not be used routinely. It should be used to assist with diagnosis of MDR TB (where it is not always possible to obtain a sample for culture) and in research settings.

TST should be done using 2 TU of tuberculin PPD RT23. A TST should be regarded as positive as follows:

- in high-risk children (includes HIV-infected children and severely malnourished children, i.e. those with clinical evidence of malnutrition): >5 mm diameter of induration;
- in all other children (whether they have received a Bacille Calmette–Guérin (BCG) vaccination or not): >10 mm diameter of induration.

**Note:** There can be false-positive as well as false-negative TSTs. Sometimes it is useful to repeat the TST in children once their nutritional status has improved or their severe illness (including TB) has resolved, as they may be initially TST negative, but positive after 2–3 months on treatment. A negative TST never rules out a diagnosis of TB in a child.

### Bacteriological confirmation of childhood TB

Diagnosis of TB in a child should be confirmed using whatever specimens and laboratory facilities are available. Bacteriological confirmation is especially important for children who have:

- presumptive drug-resistant TB
- HIV infection
- complicated or severe cases of disease
- an uncertain diagnosis.

Pulmonary TB in children tends to be smear negative and obtaining samples for laboratory investigations can be challenging. However *all children starting TB treatment should have a specimen collected for culture*

Appropriate specimens from the probable sites of involvement should be obtained for microscopy and, where facilities and resources are available, for culture (and also histopathological examination). Appropriate clinical samples include sputum, gastric aspirates and certain other material (e.g. lymph node biopsy or any other material that is biopsied). Fine-needle aspiration (FNA) of enlarged lymph glands – for both staining of acid-fast bacilli and histology – has been shown to be a useful investigation, with a high bacteriological yield.

#### Techniques for obtaining specimens from children

Common ways of obtaining samples for smear microscopy include the following.

***With the increased rates of DR TB, routine culture should be requested for all children starting TB treatment whenever possible***

The main 3 available techniques used to obtain respiratory samples are: gastric aspirate, nasopharyngeal aspirate (NPA) and induced sputum (See Annexes 5 and 6).

Table 9.1: Sample collection techniques in children

|  | **Gastric Aspirate** | **Nasopharyngeal Aspirate** | **Induced Sputum** |
| --- | --- | --- | --- |
| **Yield** | 30-40% for 3 GA, increased in younger and sicker children | Similar yield reported to induced sputum | 1 sputum same yield as 3 GA |
| **Specimen collected** | Gastric fluid | Upper secretions (lower secretions can be obtained if the child coughs) | Lower respiratory secretions |
| **Characteristics** | Invasive, doesn’t need special equipment | Simple, non-invasive, but needs special equipment | Needs special equipment and well ventilated areas |

*When DR TB is probable, the child should be referred for evaluation and to assure a specimen collection.*

***a. Expectoration***

Sputum should always be obtained for smear microscopy (and mycobacterial culture if available) to exclude the possibility of TB.

***b. Gastric aspiration***

Gastric aspiration using a nasogastric feeding tube can be performed in young children who are unable or unwilling to expectorate sputum. The procedure can be done by nurse or doctor. . Gastric aspirates should be sent for smear microscopy and mycobacterial culture. A gastric aspirate can be obtained on the same day 4 hours apart. For in patients, two gastric aspirates should be obtained on each of two consecutive mornings.

***c. Sputum induction and nasopharyngeal aspirate***

Several recent studies have found that sputum induction is safe and effective in children of all ages and the bacterial yields are as good as or better than for gastric aspirates. However, training and specialized equipment are required to perform this procedure properly.

***d. Fine needle aspiration***

Fine needle aspiration is a useful technique to collect samples from an enlarged lymph node for both histology and staining for acid-fast bacilli (AFB). It should be mainly use for children with persistent LN not responding to adequate TB treatment (See Annex 6).

## Paediatric Extrapulmonary TB

Young children (<2-3 years of age) with immature cellular immune responses are at highest risk of developing extra-pulmonary forms of disease.

A review of the natural history of TB disease in children demonstrated that up to 50 % of infants progress to active TB disease after primary TB infection (in the first few months), with 10 to 20 % developing miliary TB and/or TB meningitis (TBM).

Extra-pulmonary disease may also develop years later (e.g. osteo-articular or renal involvement) following reactivation of organisms sub-clinically disseminated during primary infection.

### TB meningitis

TB meningitis is a very severe form of TB disease, which is more common in children under 5 years.

Symptoms are very nonspecific and include: headache, early morning vomiting and convulsions, neck stiffness, reduced consciousness and cranial nerve palsies. Hydrocephalus frequently develops as a complication of TB meningitis (TBM), and needs urgent referral to a referral hospital.

Clinical diagnosis can be made whenever a child with meningitis is not responding to standard therapy (especially in HIV positive children). Diagnosis can be supported by a lumbar puncture where specific findings are: raised proteins, low glucose and lymphocytosis.

Any CSF sample needs to be sent for smear and culture/DST.

**Due to its poor prognosis treatment for TB meningitis has to be initiated as soon as it is suspected.**

### Lymph node TB

This type of tuberculosis represents the most common extra-thoracic manifestation of TB. Lymph node TB presents with persistent painless glands for more than 2 weeks, with no response to antibiotics. Glands may become fluctuant prior to spontaneous drainage and sinus formation (scrofula). Laboratory confirmation is not needed to start treatment. However, if there is no adequate response to TB treatment, additional laboratory tests (FNA and/or biopsy) can be performed to rule out other less common conditions.

### Abdominal TB

May present as peritonitis, malnutrition, abdominal distension with ascites, or bowel, biliary or lymphatic obstruction due to the compressive effects of enlarged intra-abdominal nodes.

Whenever possible, an abdominal ultrasound should be done, as **abdominal lymph nodes in a child are highly suggestive of abdominal TB.**

### Bone and joint disease

Most cases arise in older children who may present with painful limbs or joints or a limp frequently misattributed to trauma. Spinal TB represents 50% of all osteo-articular TB and can present as a backache of a few weeks duration, with spine deformity.

### BCG disease

Currently there is one attenuated vaccine for TB (M.bovis BCG), which has proven to offer a significant protection against disseminated TB (miliary and TBM) in HIV-uninfected children. In HIV-infected children, there are concerns regarding the possible complications related to the vaccine.

Due to the high TB prevalence in Swaziland, BCG Vaccination is recommended in all children at birth.

A normal reaction to the BCG vaccine includes a small area of redness, followed by a raised papule and a shallow ulcer that will heal up to around 14 weeks after vaccination.

BCG disease can present (especially in HIV infected children) as:

- **Local BCG disease**: abscess at the site of injection more than 10mm or ulceration that lasts more than 14 weeks.
- **Regional BCG disease:** Involvement of regional lymph nodes (ipsilateral axillary, supraclavicular, cervical and upper arm glands) more than 15mm. (Ipsilateral axillary LN in infants are likely BCG disease).
- **Distant BCG disease:**  Involvement of any site beyond a local or regional ipsilateral process (BCG confirmed from sputum, CSF, urine, bone or distant skin lesion). Clinical relevant symptoms may be present.
- **Disseminated disease:**  BCG confirmed from more than 1 remote site and/or from at least one blood or bone marrow culture.

BCG disease can also present as IRIS during the first 3 months after initiation of ART therapy.

*If BCG disease is suspected, refer the child to a hospital or specialized center, as full TB treatment may be needed in severe forms.*

The decision to treat BCG disease depends on the immune system of the child and the extent of the disease.

***HIV-uninfected children*:** Usually they do not require treatment and therapeutic FNA can be considered if the node is fluctuating. They need to be followed up every 3 months until it has been totally cured.

***HIV-infected children*:** Treatment is recommended. As M.Bovis is resistant to pyrazinamide, a Fluorquinolone (Levofloxacine 7.5-10mg/kg or Ofloxacine 15mg/kg) has to be added. Co-infection with MTB has been reported, so children who need treatment should start with a five-drug regimen (RHZE + Fluorquinolone) for 2 months and complete further 7 months with RH.

Mortality in HIV positive children with BCG disease is very high, so prompt initiation of HAART is strongly recommended.

## Paediatric DR-TB

DR-TB can also affect children, but due to the difficulty in obtaining samples, they are rarely diagnosed. It is crucial that we make all possible efforts to collect a specimen for Gene Xpert/culture and DST and refer the children for further assessment.

DR-TB is suspected if:

- A positive MDR-TB contacts exists.
- There is poor response to DS TB treatment-persistent symptoms or failure to gain weight.
- It is a retreatment case.

## PadediatricTB treatment

### Treatment of susceptible paediatric TB

Due to the high HIV prevalence and INH resistance, all children starting TB treatment need to receive a four-drug regimen (HRZE) during the initial phase, followed by a continuation phase of 2 drugs (RH) for a minimum period of 4 months.

Table 9.2: Anti-TB drugs doses for children

| **Drug** | **Doses (mg/kg)** | **Range (mg/kg)** |
| --- | --- | --- |
| **Rifampicine (R)** | 15 | 10-20 |
| **Isoniazid (H)** | 10 | 10-15 |
| **Pyrazinamide (Z)** | 35 | 30-40 |
| **Ethambuthol (E)** | 20 | 15-25 |
| **Streptomycine (S)** | 15 | 12-18 |

*Source: WHO 2010. Rapid Advice on the treatment of tuberculosis in children. WHO/HTM/TB/2010.13.*

**All children starting TB treatment should be initiated on:**

**2RHZE / 4RH**

**Children with severe immune-suppression and severe forms of TB disease need to complete 9 months of treatment on:**

**2RHZE / 7RH**

Table of severe immunosuppression in children:

Table 9.3: Scale of immuno suppression in children

|  | Under 5 years old | 5 years and up |
| --- | --- | --- |
| **CD4%** | ≤25% | ≤350 cells/mm^3^ |
| **Absolute CD4** | ≤750 cells/mm^3^ |  |

**Children with TBM and osteoarticular TB should complete 1 year of treatment on**

**2RHZE / 10RH**

In all children previously treated for TB, **all efforts will be put in place to assure a sample is obtained for culture and DST**.

In case resistant TB is probable, the child has to be referred to a facility where MDRTB treatment is available for evaluation.

### Use of Steroids in Pediatric TB forms

1-2mg/kg of prednisone is recommended in severe forms: TB meningitis, TB pericarditis, obstruction of the airway and sick children with miliary TB.

Corticoids should be given for 4-6 weeks, with tapering over 2 weeks.

### Paediatric anti-TB drugs dosage

**Children less than 15 KG:**

RHZ (60/30/150), H 100mg and E 100mg

| **Weight bands** | **INITIATION PHASE** | | | **CONTINUATION PHASE** | |
| --- | --- | --- | --- | --- | --- |
|  | RHZ (60/30/150) | INH 100mg | Ethambuthol | RH (60/30) | INH 100mg |
| 2 - 2.9 kg | 0.5 | 0.25 | 50mg | 0.5 | 0.25 |
| 3 - 5.9 kg | 1 | 0.5 | 100mg | 1 | 0.5 |
| 6 - 7.4 kg | 1.5 |  |  | 1.5 |  |
| 7.5 - 8.9 kg | 2 |  | 200mg | 2 |  |
| 9 - 11.9 kg | 2.5 |  |  | 2.5 |  |
| 12 - 14.9 kg | 3 |  |  | 3 |  |
| All children on TB treatment should receive 1 tablet of pyridoxine per day (12.5mg) | | | | | |

*Source: Rapid advice: Treatment of tuberculosis in children. WHO/HTM/TB/2010.13*

**Children from 15 to 39.9kg:**

RHZE (150/75/400/275) and H 100mg

| **Weight bands** | **INITIATION PHASE** | | **CONTINUATION PHASE** | |
| --- | --- | --- | --- | --- |
|  | RHZE (150/75/400/275) | INH 100mg | RH (150/75) | INH 100mg |
| 15 - 20.9 kg | 1.5 | 1 | 1.5 | 1 |
| 21 - 25.9 kg | 2 | 1 | 2 | 1 |
| 26 - 29.9 kg | 2.5 | 2 | 2.5 | 2 |
| 30 - 34.9 kg | 3 | 2 | 3 | 2 |
| 35 - 39.9 kg | 3.5 | 2 | 3.5 | 2 |
| All children on TB treatment should receive 1 tablet of pyridoxine per day (25mg) | | | | |

*Source: Rapid advice: Treatment of tuberculosis in children. WHO/HTM/TB/2010.13*

## Paediatric MDR-TB treatment

Children diagnosed with MDRTB will be initiated following the same regimen as for adults.

If there is a known contact case, the child will be put on the same regimen as the contact case, while waiting for his DST result.

Any child with an indication of having MDR-TB has to be referred to a MDR-TB Centre for assessment and possible initiation on empiric MDR-TB treatment.

Before initiating treatment, other possible causes for poor response to therapy need to be investigated:

- - Severe immune-suppresion.
  - Poor adherence, due to lack of adequate support.
  - Treatment failure of ARVs.
  - IRIS

## Follow up of children on TB treatment

For children starting TB treatment, the first follow up is recommended at 2 weeks, 4 weeks and monthly thereafter. To assure a good outcome, on each visit the following should be monitored:

- **Weight:** It has to be checked each visit and documented in the TB card. An increase in the weight is one of the best indicators we have of successful treatment.
- **Doses:** They need to be adjusted every visit, according to the weight.
- **Adherence:** Good adherence is essential to assure good treatment outcomes. The HCW need to asses in each visit:
  - Who is the main caregiver.
  - Who is in charge of giving the tablets (DOT is highly encouraged).
  - What happens when the main caregiver is not at home.
  - If there is any other problem compromising the adherence.

Orphans are especially vulnerable, and they need special attention to assure good adherence. If adherence is compromised due to the social situation, we can consider long term hospital admissions while the social situation is solved together with the child welfare services.

CXR is not required routinely in the follow up of a child if there is good clinical response to anti-TB treatment, as they can have slow radiological response.

## TB-HIV COINFECTION

All children on TB treatment should be tested for HIV. ***Consent for paediatric testing can be given by any parent, guardian, caregiver, health care worker or social worker when it’s in the best interest of the child.***

### Anti-Retroviral Therapy

All HIV positive children diagnosed with TB are clinically eligible for ART initiation.

High pill burden, poor supervision, inadequate social support and lack of disclosure can jeopardize the adherence of children on TB and HIV medication. To assure a good long-term outcome on ARVs, a thorough social assessment needs to be done before starting ARVs, identifying 2 caregivers whenever possible and disclosing the HIV status of the child if they are more than 10 years old.

#### Recommended regimens for HIV+ pediatric patients on TB treatment:

For children who are treatment naïve or on any first-line ART regimen:

NEED TO CLARIFY ABOUT LONG TERM EXPOSURE TO NVP

Table 9:Recommended Regimens for Pediatric Patients on TB treatment

| **under 3 years of age** | **Age 3 and over** |
| --- | --- |
| AZT–3TC–NVPa | AZT–3TC–EFV |
| If a patient is on TB therapy NVP should be initiated at twice-daily dosing. Due to enzyme induction by rifampicin, lead-in dosing is not indicated (as will increase the risk of developing NVP resistance).  Children who had long term exposure to NVP for PMTCY prophylaxys, will be changed to a NNRTI spared regimen as soon as they complete the TB treatment (Lopinavir/ritonavir based regimen) | |

#### Alternative ART regimen options for special situations requiring TB/HIV co-treatment:

Table 10: Alternative Paediatric Regimens for Special Situations

| Special Situation | Alternative Regimen | Comments |
| --- | --- | --- |
| **Child is currently on or qualifies for an LPV/r-based first-line regimen** | May use LPV/r  boosted 1:1 with ritonavir,  if available | Only use if ritonavir availability can be ensured for the duration of TB treatment. |
| **Child is on second-line therapy** | If current regimen contains  LPV/r, continue with  1:1 ritonavir-boosting | Consult a TB or HIV specialist if ritonavir is not available. |
| **Children > 3 years old already on NVP-based regimen before initiating  TB treatment** | AZT–3TC–NVP | Studies in adults show  that NVP-based regimens maintain viral suppression  as well as EFV-based regimens in this situation. |
| **NVP or EFV toxicity** | AZT–3TC–ABC | Immediately switch to  an LPV/r-based regimen when TB therapy is completed. |
| **Severe anaemia*(Hb<8 g/dl)*** | Use d4T instead of AZT | Switch to AZT when stable |

*Source: WHO guidelines for antiretroviral therapy for HIV infection in children 2010 revision and South African ART guidelines.*

To reduce pill burden and facilitate adherence children should be switched from EFV to NVP once TB treatment is complete (no leading dose of NVP is needed in these cases).

### Cotrimoxazole Prophylaxis

Cotrimoxazole has been shown to reduce HIV related morbidity and mortality. Therefore, all HIV positive children should receive lifelong prophylaxis with Cotrimoxazole.

Table 11: Dosing for Cotrimoxazole*

| **Age** | **Weight of child** | **Suspension 5ml (200mg/40mg)** | **Pediatric Tablet (100mg/20mg)** | **Dose** |
| --- | --- | --- | --- | --- |
| **< 6 months** | <5kg | 2.5ml | 1 tablet | 120mg |
| **6m – 5yr** | 5 - 15kg | 5ml | 2 tablets | 240mg |
| **6yr – 14 yr** | 15 - 30kg | 10ml | - | 480mg |
| **>14yr** | >30kg | - | - | 960mg |

*Source: WHO guidelines for antiretroviral therapy for HIV infection in children 2010 revision and South African ART guidelines.*

*To be administered as single once daily dose.

### Administering treatment and ensuring adherence

Treatment of TB in children should be administered on an ambulatory basis. Children, their parents and other family members, and other caregivers should be educated about TB and the importance of completing treatment. The support of the child's parents and immediate family is vital to ensure a satisfactory outcome of treatment.

Children with severe forms of TB should be hospitalized for intensive management where possible e.g. in:

- respiratory distress,
- Spinal TB, and (iv) severe adverse events, such as clinical signs of hepatotoxicity (e.g. jaundice).

If it is not possible to ensure good adherence and treatment outcome on an outpatient basis, some children may require hospitalization for social or logistic reasons.

## Prevention of Paediatric TB

Early diagnosis and treatment of adults (especially for smear-positive cases) is essential to reduce the number of TB infections in children. In addition, isoniazid preventive therapy (IPT) for children exposed to tuberculosis infection but without disease has been proven effective to prevent TB in HIV positive and negative children.

### Paediatric Isoniazid Preventive Therapy

All children *with a negative screening for PTB* should receive IPT for 6 months if:

- They are under 5 years old, irrespective of their HIV status, when there is a documented exposure to PTB. For every new TB contact, IPT should be repeated.
- They are HIV positive and more than 1 year old, even if there is no contact (if no prophylaxis in the previous 2 years).
- Newborn babies born to a mother with tuberculosis in pregnancy. Vertical transmission of TB (*Congenital TB*) can happen, especially if the mother is diagnosed in the last 3 months of pregnancy and is sputum smear-positive. In these cases, *if there are no TB symptoms in the newborn*, INH prophylaxis for 6 months is recommended for the newborn. This children should withhold BCG vaccination until completion of IPT.

Table 12: Dosage of INH for prophylaxis in Children

| **Dosing of Isoniazid**  **(INH should be given at a dose of 10mg/kg together with Vitamin B_6)_.** | | |
| --- | --- | --- |
| **Weight range (Kg)** | **Number of 100mg tablets of INH to be administered per dose** | **Dose given (mg)** |
| **<5** | ½ tablet | 50 |
| **5.1 – 9.9** | 1 tablet | 100 |
| **10 – 13.9** | 1½ tablets | 150 |
| **14 - 19.9** | 2 tablets | 200 |
| **20 – 24.9** | 2 ½ tablets | 250 |
| **>25** | 1 ADULT dose | 300 |

*Source: WHO 2010. Rapid Advice on the treatment of tuberculosis in children. WHO/HTM/TB/2010.13.*

**FOR ANY QUESTION REGARDING PEDIATRIC TB/HIV,**

**PLEASE CONTACT THE FREE BAYLOR HOT LINE:**

**2404 8569**

1. COLLABORATIVE TB AND HIV ACTIVITIES

## TB/HIV interaction

TB and HIV are inextricably linked. While TB is the leading cause of mortality among people living with HIV virus, HIV remains the most powerful risk factor for developing TB.

TB can occur at any point in the course of progression of HIV infection and the risk of developing TB rises sharply with worsening immune status. A person infected with HIV has over 20 times increased risk of developing TB disease. Mortality in HIV+ TB patients is 2-4 times higher (6% to 39% in SSA) than in HIV- TB patients. In SwazilandHIV is considered to be the major factor fueling the TB epidemic. Currently 83% of incident TB cases are also co-infected with HIV.

Early detection and effective treatment of TB among HIV-infected patients is critical to prolong the lives of people living with HIV/AIDS.

Swaziland has made significant progress in implementation of the WHO interim policy on TB/HIV collaborative activities. Current HIV testing rate among TB patients is at 94%, 95% of registered patients are on CPT, and about 50% of co-infected patients have been initiated on ART.

This guideline emphasizes the need to ensure early ART initiation in co-infected TB patients within 8 weeks of staring anti-TB treatment.

***Note: Same tuberculosis treatment should be administered to both HIV + and HIV – TB patients. HIV positive patients can be completely cured of tuberculosis.***

## HIV Testing and Counseling (HTC)

TB is often the first clinical indication that a person may have an underlying HIV infection; hence the importance of TB services as an entry point to HIV prevention, care and treatment.

HIV testing and counseling should be offered to all patients of all ages who present with signs or symptoms suggestive of tuberculosis or have confirmed TB (Standard 14 of the ISTC).

The family-centered approach to HIV testing should be employed such that once a family member is identified as having HIV, health workers should encourage and actively facilitate HIV testing for other family members.

Appropriate post-test counseling should be ensured, with a strong focus on HIV prevention; as this will also help prevent the spread of TB.

Results of HIV testing should be properly documented in the appropriate columns of the Tuberculosis register.

## HIV prevention in TB patients

The HIV prevention package in Swaziland includes behavior change campaigns; promotion of the ABC Strategy (i.e Abstinence, Being faithful to one partner, and Condom use); as well as Male Circumcision (MC). Provision of health promotion messages and distribution of condoms should be reinforced to all TB patients regardless of their HIV status. Similarly information on Male circumcision should be offered to all male patients who test negative.

## TB treatment in people living with HIV

Treatment of tuberculosis is essentially same for HIV co-infected and HIV negative TB patients.

All drug-sensitive HIV positive TB cases should be treated with the standard 6-month regimen as outlined in the Treatment section of this guideline page???

All central nervous system (CNS) related tuberculosis cases should have treatment extended to 9 months as outlined in the treatment section ???

Drug resistant disease should be treated by the specialized centres in line with the MDR-TB treatment guidelines.

## Co-trimoxazole preventive therapy

Co-trimoxazole preventive therapy substantially reduces morbidity in HIV-positive TB patients by reducing the risk for recurrent bacterial infections, malaria and Pneumocystis jirovecii.

All TB/HIV co-infected patients should receive co-trimoxazole preventive therapy throughout the duration of the anti-tuberculous therapy and continue lifelong thereafter.

## Antiretroviral therapy

Antiretroviral therapy improves survival in HIV-positive patients. In addition, antiretroviral therapy reduces TB rates by up to 90% at an individual level, by 60% at a population level and it reduces TB recurrence rates by 50%.

ART should be initiated for all people living with HIV with active TB disease irrespective of CD4 cell count.

TB treatment should be initiated first, followed by ART as soon as possible preferably within the first 8 weeks of starting TB treatment.

### Interactions with ART Regimens

Standardized, simplified ART regimens are used to support HIV treatment programmes so they can reach as many people living with HIV as possible.

Guidance for concomitant administration of first-line anti-retrovirals is given below. There are few long-term clinical outcome data to support use of these TB/HIV drug combinations.

Table 13: Anti-TB drugs /ARV Regimen Recommendations

| ARV group | Recommendations |
| --- | --- |
| Nucleoside/nucleotide reverse transcriptase inhibitors (NRTIs) | There are no major interactions between rifampicin and lamivudine (3TC), emtricitabine (FTC), tenofovir, abacavir, zidovudine (AZT) or didanosine (ddI).  Stavudine (d4T) should not be given because of the increased risk of peripheral neuropathy with concomitant TB therapy. |
| Non-nucleoside reverse transcriptase inhibitors (NNRTIs) | The preferred regimen for patients who have no contraindication is:  ***Rifampicin + Efavirenz*** - Use standard dose 600mg/day in patients  Alternative regimen is:  ***Rifampicin + Nevirapine - Not recommended***, but if given then use standard doses and perform Nevirapine therapeutic drug monitoring |
| Protease inhibitors (PI) | ***Rifampicin + un-boosted PI - DO NOT USE***  ***Rifampicin + boosted PI - Not recommended*** due to evidence of poor pharmacokinetics and high rates of hepatotoxicity seen in studies with healthy volunteers. |

*Source: WHO guidelines for antiretroviral therapy guidelines for treatment of HIV infection in adults and adolescents 2010 revision and Swaziland National ART guidelines.*

Swaziland HIV treatment guidelines (2010) recommend as first line TDF+3TC+EFV, with alternatives being AZT+3TC+EFV (for those who cannot tolerate TDF).

For patients who cannot use Efavirenz (1^st^ trimester pregnant women, patients with mental health disease), it is substituted with Nevirapine.

Alternatives for patients who are intolerant to efavirenz or are infected with a strain of HIV that is resistant to NNRTIs, a triple NRTI regimen (AZT+3TC+ABC or AZT+3TC+TDF) may be used for the duration of the anti-tuberculosis treatment and patient MUST be switched back to their original regimen.

In individuals who need TB treatment and who require an ART regimen containing a boosted protease inhibitor (PI), it is recommended to give a regimen containing lopinavir or saquinavir with additional ritonavir dosing ; this regimen should be closely monitored as the PI serum concentration is very much reduced by the rifampicin. Patients should be monitored for treatment failure.

### When to start ART?

Currently available evidence suggests that early provision of ART reduces morbidity and mortality in TB/HIV co-infected patients. However, starting ART during TB treatment may be complicated by overlapping toxicities, drug-to-drug interactions, immune reconstitution disease, as well as high pill burden, which may negatively affect adherence. On the other hand, delaying ART may lead to prolonged or worsening immune suppression. Clinicians need to balance these risks when deciding when to initiate concomitant HAART and TB treatment. The revised

- START All TB patients with HIV on antiretroviral therapy (ART) as soon as possible (and within the first 2 weeks of starting anti-TB treatment) regardless of immune system measurements and not later than 8 weeks of starting anti TB treatment.

## Drug susceptibility testing

High mortality rates have been reported among people living with HIV who have drug resistant-TB, and death rates can exceed 90% in patients co-infected with extensively drug-resistant TB (XDR-TB) and HIV.

Prompt initiation of appropriate TB treatment (and subsequent initiation of ART) can reduce mortality among people living with HIV who have drug-resistant TB.

A drug sensitivity testing (DST) should be requested at the start of TB therapy for all HIV-positive TB patients, to avoid mortality due to unrecognized drug-resistant TB. (refer to Chapter 4: Diagnosing TB).

## Dealing with TB diagnosed in patients already on ART

Patients receiving any HIV care including ART should be continuously screened for TB using the standard screening tool, and if positive on screening should be properly investigated to establish TB diagnosis.

When TB is diagnosed in patients already receiving ART, ant-TB treatment should be started immediately.

There are two issues to consider in such cases:

- Whether ART needs to be modified because of drug–drug interactions or to reduce the potential for overlapping toxicities (see section on ART)
- Whether the presentation of active TB in a patient on ART constitutes ART failure that requires a change in the ART regimen. (Refer Swaziland HIV Treatment guidelines section on Diagnosis and management of ART failure)**.**

## HIV-related prevention, treatment, care and support

The recommended package of HIV-related prevention, treatment, care and support services and support for people living with HIV as outlined in the Swaziland HIV Package of care guideline should be provided either by TB clinics or by referral to HIV/AIDS programmes.

To improve treatment success, the special needs of particular groups (e.g. drug users, prisoners, migrant populations and other marginalized groups) should be assessed and addressed; their care should be integrated with other services.

#### Managing side effects in concurrent TB/HIV treatment

Patients on concurrent anti-tuberculous therapy and ART may experience overlapping toxicities due to both treatments.

These patients should therefore be closely monitored especially for evidence of hepatic damage through monthly ALT assay.

Due to the high risk of peripheral neuropathy associated with the use of INH, and D4T regimens, it is recommended that patients be placed on pyridoxine (vitamin B6) as a routine part of anti-tuberculous therapy in co-infected patients using the following dosages:

***Pyridoxine:***

*Adults and Children > 3 years of age:* 25mg po daily

*Children < 3 years of age:* 12.5mg po daily

#### Directly Observed Therapy for concomitant TB/HIV treatment

Due to the difficulties of taking anti-tuberculous therapy and ART as well as the risk of developing drug-resistance, it is recommended that all patients receive directly observed therapy (DOT) during the course of their TB treatment.

DOT should be provided by a paid treatment supporter who fills out the DOT card. The importance of patient education and empowerment around medication taking is a key part in improving adherence as is addressing socioeconomic barriers.

#### Monitoring patients on concurrent ART and DOTS

**Clinical Monitoring**

Clinical assessment should be the primary tool for monitoring adults both before and after initiation of ART.

After starting ART, clinical assessments should take place by a doctor or nurse at 2 weeks, 1 month, 2 months, 3 months, 6 months, and at least every 6 months thereafter

A focused history and physical should be performed during routine visits. Important features of regular clinical assessments should include:

- monitoring of
  - weight (done at every visit)
  - height (in children, done every 3 months)
  - head circumference (in children < 3 years of age, measured every 3 months)
  - developmental status in children;
  - nutritional status in children;
- diagnosis and management of interim or new illnesses
  - OIs that may suggest immune reconstitution syndrome or treatment failure;
  - other co-morbidities, including STIs, Hepatitis B, substance abuse, psychiatric illness
- medication review
  - side effects
  - adherence and dosing
  - other medications, including traditional medicines and other medications that may interact with ARVs
- early diagnosis of pregnancy
- changes in social situation that might affect adherence to ART

**Laboratory Monitoring**

Laboratory monitoring should complement the clinical assessments. Baseline laboratory tests will help to determine which regimen a person should be initiated on. However, the absence of the capacity to perform laboratory testing should not preclude a person from starting ART.

**Baseline Laboratory Investigations**

Where possible, the following baseline laboratory investigations should be obtained prior to starting ART:

- CD4 count, or percentage (in children < 5 years)
- Full blood count (FBC)
- ALT
- Serum Creatinine when Tenofovir (TDF) is being considered in adults, followed by calculation of the rate of Creatinine Clearance *(for details of calculation method for Creatinine clearance, please refer to the National ART guidelines)*
- Pregnancy test in all women of child-bearing age

**Routine Laboratory Investigations**

The following laboratory tests should be performed routinely depending on the specific ARVs that are included in the patient's regimen:

- If on AZT, Haemoglobin (Hb) should be checked at 1 month, 2 months, 3 months, 6 months, and every 6 months thereafter.
- If on NVP, ALT should be checked at 1 month, 2 months, 6 months, and every 6 months thereafter. If the CD4 count at initiation is between 250-350, there is an increased risk of hepatotoxicity, so additional ALT testing is recommended at 2 weeks and 3 months.
- If on Tenofovir (TDF), serum creatinine (and rate of Creatinine Clearance) should be checked 6 months after initiation, and every 6 months thereafter.
- CD4 counts should be checked every 6 months, to help determine efficacy of treatment

Additional laboratory tests can be requested depending on the results of the clinical assessments, but should only be done if the result is required to further guide management. These include, but are not limited to:

- Lactate measurement, if the patient is on a NRTI (especially d4T or ddI) for > 4 months and losing weight, and/or having other symptoms that suggest hyperlactatemia^[[4]](#footnote-4)^
- Glucose and lipid measurements, if the patient is taking a Protease Inhibitor, such as Lopinavir/ritonavir (Kaletra) or Atazanavir/ritonavir

#### Immune Reconstitution Inflammatory Syndrome among patients with HIV-related TB

- Fever
- New or worsening adenitis - peripheral or centralnodes
- New or worsening pulmonary infiltrates, including respiratory failure
- New or worsening pleuritis, pericarditis, or ascites
- Intracranial tuberculomas, worsening meningitis
- Disseminated skin lesions
- Epididymitis, hepato-splenomegaly, soft tissue abscesses

Table 14: Overlapping Side effect adverse reactions to First-line anti-TB and ART drugs

| **Side Effects** | **Possible causes** | |
| --- | --- | --- |
|  | **Anti-TB Drugs** | **ARV Drugs** |
| Skin rash | PZA, RIF, INH | NVP, EFZ, ABC |
| Nausea, vomiting | PZA, RIF, RBT, INH | ZDV, RTV, AMP, IDV |
| Hepatitis | PZA, RIF, RBT, INH | NVP, PIs, Immune  reconstitution |
| Leukopenia, anemia | RBT, RIF | ZDV |

*Source: WHO guidelines for antiretroviral therapy guidelines for treatment of HIV infection in adults and adolescents 2010 revision and Swaziland National ART guidelines.*

1. TB INFECTION CONTROL

## When is TB infectious?

Persons with Tuberculosis of the lungs or larynx are the most infectious and constitute potential sources of spread to others.

In general any person with presumptive TB should be considered infectious until proven otherwise through a laboratory confirmation by negative sputum smear result.

## Rationale for TB Infection Control

Persons with undiagnosed, untreated and potentially contagious TB are often seen and managed in Health care settings; and such frequent exposure to patients with infectious TB disease may put the health worker at risk. Furthermore Health care workers and staff may themselves be immunosuppressed due to HIV infection and be at higher risk of developing TB disease once infected.

Nosocomial transmission of M. tuberculosis has been linked to close contact with persons with TB disease during aerosol-generating or aerosol-producing procedures, including bronchoscopy, endotracheal intubation, suctioning, other respiratory procedures, open abscess irrigation, autopsy, sputum induction, and aerosol treatments that induce coughing.

All health facilities should be made aware of the need for preventing transmission of M. tuberculosis especially in settings where persons infected with HIV might be encountered or might work. All HCWs should be sufficiently informed regarding the risk for developing TB disease after being infected with M. tuberculosis.

All health-care settings should develop a TB infection-control plan designed to ensure prompt detection, airborne precautions, and treatment of persons who have presumptive or confirmed TB disease. TB infection control measures can be divided into three categories namely: Administrative, Environmental (or engineering) and Personal respiratory protection controls.

## Infection Control measures

Infection Control measures are designed based on the high likelihood of persons with infectious TB being attended to in health care settings, and the possibility of them spreading *M. tuberculosis* to other persons, especially immune-compromised patients or staff. Infection control interventions are meant to significantly reduce this risk of such transmission in health care and other settings.

In general, there are two main approaches by which chances of transmitting M. tuberculosis can be reduced in health care settings:

- Administrative (work place) control measures
- Environmental control measures.

### Administrative infection control measures

Administrative control measures serve as the first line of defense for preventing the spread of TB in Health care settings, and usually have the greatest impact on preventing TB transmission within settings. These measures prevent droplet nuclei containing M. tuberculosis from being generated in the facility, and thus reduce exposure of patients and staff to TB. Administrative controls are therefore considered priority irrespective of availability of resources.

Administrative controls have 5 main elements:

1. Infection control plan;
2. Administrative support for procedures in the plan, including quality assurance;
3. Training of staff;
4. Education of patients and increasing community awareness; and
5. Coordination and communication with the TB program.

#### Infection Control plan

Each facility should have a written TB infection control plan that outlines a protocol for the prompt recognition, separation, provision of services, investigation for TB and referral of patients with presumptive or confirmed TB disease.

The plan should designate a staff member as an ***Infection Control Officer*** who is responsible for overseeing the implementation of the infection control procedures in the health facility.

The plan should address the following policy and practice areas:

1. Screening all patients as soon as possible after arrival at the facility to identify persons with symptoms of TB disease or persons who are being investigated or treated for TB disease.
2. Instructing the persons identified through screening on respiratory hygiene/cough etiquette. This includes instructing them to cover their nose and mouth when coughing or sneezing, and when possible and if acceptable, providing face masks or tissues to assist them in covering their mouths.
3. Surgical masks could be provided to persons who have a positive symptom screen to wear until they leave the facility. Alternatively, tissues can be provided to these persons, with instructions to cover their mouths and noses when coughing or sneezing. These are less costly and also less likely to identify people as having presumtive TB with attendant risk of stigma. Tissues and face masks to be disposed properly in waste receptacles.
4. Clients and especially staff should be encouraged to wash their hands after contact with respiratory secretions. Placing persons with presumptive TB and confirmed TB cases in a separate well-ventilated waiting area such as a sheltered open-air space if possible;.
5. Speeding up management of persons with cough so that they spend as little time as possible at the facility.
6. Ensuring rapid diagnostic investigation of persons with presumptive TB, including referring them to TB diagnostic services if not available on site; and ensuring that persons reporting TB treatment are adhering with their treatment.
7. Appropriate use and maintenance of the environmental control measure facilities; Training and educating all staff on TB and the TB infection control plan (training should include special risks for TB for HIV-infected persons, and need for diagnostic investigation for those with signs or symptoms of TB).
8. Provision of voluntary, confidential HIV counseling and testing for staff with adequate access to treatment.
9. Monitoring the TB infection control plan’s implementation and correcting any inappropriate practices or failure to adhere to institutional policies.

### Environmental control measures

Because administrative controls may not eliminate all possible exposure, environmental control measures must be added to reduce the concentration of droplet nuclei in the air. Environmental controls are therefore considered as the second line of defense for preventing the spread of TB in health care settings. It is important to recognize that if administrative controls are inadequate, environmental controls **will not eliminate the risk.**

Environmental controls include:

- - Ventilation (natural and mechanical),
  - Filtration, and
  - Ultraviolet germicidal irradiation.

Ventilation is the movement of air in a building and replacement of air in a building with air from outside. Natural ventilation relies on open doors and windows to bring in air from the outside. When fresh air enters a room it dilutes the concentration of particles in room air, such as droplet nuclei containing M. tuberculosis. Controlled natural ventilation can reduce the risk of spreading M. tuberculosis.

Controlled ventilation is when:

- checks are in place to make sure that doors and windows are maintained in an open position that enhances ventilation through ensuring directional flow of air;
- Fans are used to assist in distributing or extracting the air the air; and
- Mechanical systems are used to control air-exchanges using the High Efficiency Particulate Air (HEPA) filtration system.

The following should be observed with respect to ventilation systems:

- Natural ventilation can be used as much as possible provided there is a good directional flow of air that ensures good ventilation (open windows);
- Efforts should be made to ensure that extraction and mechanical ventilation systems are maintained and functions correctly;
- Laboratories that process specimens that may be DR-TB require particularly strict environmental controls (safety cabinets).

Ventilation can be supplemented with upper-room Ultra-violet Germicidal Irradiation (UVGI), which has been known to be extremely effective in inactivating infectious particles in the air above people’s heads, while not exposing them to skin or eye irritation. It is therefore meant to decontaminate air while the infectious source and other occupants are present.

The UVGI should be properly maintained to avoid adverse reactions, such as acute and chronic skin and eye changes from overexposure if the UVGI is not installed and maintained properly.

### Personal respiratory protection

Masks that prevent TB transmission are known as ***“particulate respirators”*** or simply “respirators”. They are designed to protect the wearer from tiny (1–5 μm) airborne infectious droplets.

Respirator masks can protect health care workers from inhaling *M. tuberculosis* only if standard work practice and environmental controls are in place. However, personal respiratory protection (i.e., the selection, training, and use of respirators) should be restricted to specific high risk areas in hospitals and referral centers, such as rooms where spirometry or bronchoscopy are performed or specialized treatment centers for persons with MDRTB. Depending on the indication, a certified N95 (or greater e.g. N100) or EU-certified FFP2 (or greater) respirator should be used.

To optimize the benefit of the respirator masks, they must fit tightly on the face, especially around the bridge of the nose. Ideally, respirators should be “fit tested” for individual wearers. In addition to choosing the proper model for each worker, this process serves to educate workers.

It should be noted that respirators are different from surgical masks, or other masks made of cloth or paper. Use of a surgical mask does not protect health care workers, other staff, patients, or visitors against TB. Therefore, surgical masks are NOT recommended as a protective device against DR-TB infection for health care workers and other staff or visitors in HIV care settings.

Furthermore, because they are visible and relatively expensive, it is sometimes assumed that personal respirators alone will prevent TB transmission. However, they cannot be worn continuously and are unlikely to be in use when persons with presumptive TB or DR-TB are encountered. For these reasons, administrative controls that aim to detect and separate cases, and engineering controls that can reduce the risk even for unidentified presumptive TB cases, are more important.

## High Risk areas

Special precaution will be observed in the following high risk areas: laboratory, medical wards, X-ray department, out patients departments, TB units, ART units, pharmacy and other congregated areas with the health care facilities.

Baseline health status of the health care workers will be required at the time of enrolment and at regular intervals, pregnant and HIV positive staff to be relocated to less risk sections within the department, ensure use of personal protective equipment especially respirators and gowns, close monitoring of mechanical ventilation for functionality.

On medical wards, patient cohorting should be done based on level of infectivity, type of resistance and patents who have converted separated from those who have not converted. Cough hygiene should strictly be enforced.

Protective clothing will be required while caring for isolated patients. TB patients who need x-ray services should be encouraged to wear masks while accessing the service.

In OPDs, triaging on should be done identify patients have cough or are known to be HIVpositive so that they can be attended to as priority to prevent spread of infection to other patients.

## Measures to reduce infection transmission in community settings where there is congregation

Patient education should emphasize minimizing opportunities for transmission of DR-TB to the community in situations of congregation by observing cough hygiene and where possible avoiding places such as church, public transport, markets, and funerals before culture conversion.

Screening for TB is encouraged for persons staying in congregate institutions for example school children joining boarding schools and on prisoners on entry and at regular 6 monthly intervals.

## TB infection control in Prisons

Tuberculosis occurs up to 100 times more commonly in prisons than in civilian populations.

- The spread of tuberculosis is worsened by late diagnosis and treatment of infectious cases, and poor prison living conditions such as overcrowding
- The main strategies for achieving these goals of TB control are the early diagnosis of TB cases and their prompt and effective treatment.
- It is thus vitally important to screen new inmates by history and sputum smear microscopy if the inmates are symptomatic for TB.
- Penal reforms and improvement in prison living conditions are also important strategies for early case detection, rapid effective treatment which will reduce morbidity and mortality in prisons and so interrupt the chain of transmission
- There should be “Equivalence” of care in the prisons, i.e., all prisoners have the right to the same standard of health care as the state provides for the general community
- There should be particular attention on integrating prison and civilian TB services

#

1. MANAGEMENT OF MULTI-DRUG AND EXTENSIVELY RESISTANT TUBERCULOSIS

## Definitions

Multi-Drug-resistant TB is said to be present only through laboratory confirmation of in-vitro resistance to one or more first-line anti-tuberculosis drugs.

The national TB drug-resistance survey conducted in 2009-2010, revealed high 7.7% prevalence of MDR among new TB cases (never treated before); and 33.9% among previously treatment cases. In general, it can be projected that more than 41% of all TB patients enrolled on treatment would require a modification of their treatment due to the problem of drug resistance.

Based on the number and classes of anti-TB drugs to which the bacilli are resistant to, the various forms of anti-TB drug resistance are defined as follows:

- Mono-resistance: resistance to one anti-tuberculosis drug.
- Poly-resistance: resistance to more than one anti-tuberculosis drug, other than both isoniazid and rifampicin.
- Rifampicin Resistance: resistance to Rifampicin only without established resistance against INH.
- Multi-drug-resistance: Mycobacterium tuberculosis complex isolates with in vitro resistance against isoniazid and rifampicin, with or without resistance to additional first and second-line anti-TB drugs.
- Extensive drug-resistance: resistance to any fluoroquinolone, and at least one of three injectable second-line drugs (capreomycin, kanamycin and amikacin), in addition to multidrug-resistance.

The emergence of multi-drug resistant (MDR) TB and lately extensively drug resistant (XDR-TB) is the most serious aspect of the TB epidemic. MDR TB is difficult and expensive to treat, whilst XDR-TB is almost untreatable.

It is therefore essential to prevent the development of MDR TB. As with other forms of drug resistance, MDR TB is a largely man-made problem, being the consequence of human error in any of the following:

- prescription of chemotherapy
- management of drug supply
- patient management
- patient adherence.

## Causes of MDR-TB

From a microbiological perspective, resistance is caused by a genetic mutation that makes a drug ineffective against the mutant bacilli. From a clinical and programmatic perspective, it is inadequate or poorly administered treatment regimen that allows a drug-resistant strain to become the dominant strain in a patient infected with TB. The common causes of MDR-TB are summarized in **Table 21 below.**

Primary resistance to anti-TB drugs is also common, where the patient is infected with resistant strains from a DRTB patient. The genotypic analysis of the samples collected during the National Drug Susceptibility Survey showed that MDR epidemic in Swaziland is largely driven by recent transmission of resistant strains.

Table 15: Causes of DRTB

| Health-care providers: - Inadequate regimens  - Inappropriate guidelines or  - Non-compliance with guidelines  - Absence of guidelines  - Poor training  - No monitoring of treatment  - Poorly organized or funded TB control programmes | Drugs: inadequate supply or quality - Poor quality  - Unavailability of certain drugs (stock-outs or delivery disruptions)  - Poor storage conditions  - Wrong dose or combination of drugs | Patients: inadequate drug intake - Poor adherence (or poor DOT) - Lack of information on treatment , - Adverse effects of treatment; - Social barriers (stigma, restrictions) - Malabsorption due to other causes - Substance dependency disorders - Mental disorder; - Non-cooperative |
| --- | --- | --- |

*Source: WHO guidelines for programmatic management of drug resistant tuberculosis 2008 emergency update and Swaziland National MDR-TB guidelines.*

## When to Suspect MDR-TB

The occurrence of MDR should be suspected clinically in the following situations:

- Without prior history of TB treatment
  - Health care worker with new tuberculosis
  - Household contact of known MDR-TB case
  - Patients who have a history of migrant work
- With prior history of TB treatment:
  - Treatment after relapse or
  - Treatment failure in HIV-negative patients (sputum smear positive after five months of therapy)
  - Treatment failure in HIV-positive patients (sputum smear positive or lack of clinical improvement after two months)
  - Patients with history of multiple previous treatments in public or private sectors

The above cases should be investigated by *M. TB* Culture and Drug Susceptibility Testing (DST).The clinician should obtain two new sputum samples from the person with presumptive TB using standard operating procedures and send a request for culture and DST. See Annex for the Request Form for Smear, Culture and DST.

Two sputum samples are needed in view of high contamination rates when transporting raw sputum samples over long distances.

The clinician may initiate treatment with second-line anti-tuberculosis drugs in some of while DST results are being awaited.

## Laboratory Confirmation of MDR-TB

Drug-resistant tuberculosis is confirmed through laboratory tests that demonstrate growth in-vitro of infecting isolates *of Mycobacterium tuberculosis* in the presence of one or more anti-tuberculosis drugs. This is known as **drug-susceptibility testing**.

DR-TB can be diagnosed using one of the following bacteriological methods:

- Line Probe Assay (LPA) test indicating resistance to both Rifampicin and INH
- Liquid culture using the Mycobacterium Growth Indicator Tube technique (MGIT)
- Xpert MTB/Rif (GeneXpert) indication resistance to INH as a proxy for MDR-TB

Two (2) sputum samples should be obtained and sent to the laboratory with an accompanying Sputum examination request form for culture and DST. See Annex for the Request Form for Smear, Culture and DST.

## Management of DR TB

The management of MDR should focus both on improving the quality of case management for TB patients on first line treatment to prevent emergence of resistance as well as appropriate management of the diagnosed resistant cases.

Reference should be made to the National MDR-TB management guidelines for details of case management. However, the following basic principles should be observed in the treatment of MDR cases:

- A ‘consent to treatment’ should be obtained prior to initiation of treatment;
- Treatment regimen should consist of at least 5 drugs anti-TB drugs (both first and second line) to which the organisms have proven susceptibility.
- The drugs should be administered for at least 6 days per week, usually twice daily to minimize side effects,
- The treatment should be started preferably with the high ended recommended doses;
- Total duration of treatment should be at least 20 months including an 8-month intensive phase when injectables would be administered.
- Each dose must be given under direct observation by a treatment supporter;
- All treatment records should be properly documented and preferably kept in a database.
- Patients should not be admitted with other normal TB patients at all or admitted in general medical wards.
- When necessary and especially in very sick patients, hospitalization can be indicated for two (2) months to stabilize clinical condition as well as HIV testing and counseling and other baseline investigations;

For details of clinical management of DR-TB please refer to the National DR-TB Management guidelines.

.

1. SUPERVISION, MONITORING AND EVALUATION

## Importance of SME

A key element of the DOTS Strategy is the establishment and maintenance of a system to monitor case detection and treatment outcomes. It is essential for efficient programme management since it provides a basis for evaluating the progress made in achieving programme targets, supervision of staff and for monitoring and surveillance.

A successful monitoring and evaluation of TB prevention and control activities assumes a clear definition of roles and responsibilities (tasks) of the TB staff at all levels, e.g. national, regional, and peripheral levels. This means that each staff is expected to perform a set of activities over a defined period of time and to report progress to his/her immediate supervisor on a regular basis.

## Programme supervision

Supervision is a systematic process for increasing the efficiency of health workers by developing their knowledge, perfecting their skills, improving their attitudes towards their work, and increasing motivation, and not merely the observation of health workers to ascertain whether activities are carried out to recommended standards.

The Swaziland NTP should ensure sustenance of task-oriented supervision at all levels to increase the efficiency of health workers by developing their knowledge, perfecting their skills, improving their attitudes towards their work and increasing motivation. The NTP Central Unit should provide technical supervision support to the Regional level, while the Regionals provide same to the health facility level.

Supervision The TB Central unit team, TB laboratory coordinator, and the M&E officer should visit each TB diagnostic facility at least once in a quarter.

The emphasis of supervision to the regional level should be on supporting the Regional TB Coordinators in the discharge of their technical and managerial functions, while that of health facilities should focus on identification of TB cases and administration of treatment including follow up of cases according to national guidelines.

**Supervisory visits must be planned carefully.** Before each visit the supervisor should review the health centre’s reports, the correspondence about the reports, the findings of the last supervisory visit and corrective actions already taken.

Supervision should be conducted using the appropriate supervision tool that assesses the relevant tasks. The facilities to be visited should be notified in advance of the date and purposes of the supervisory visit. The number of supervisory visits should be planned before the start of the fiscal year, for inclusion in the annual-programme budget.

The team together with the Regional TB coordinator should:

- **Observe performance of tasks** and general adherence to national guidelines in diagnosis and managemnent of TB;
- **Review records** e.g Register of presumptive TB cases, treatment cards andTB Registers etc for accuracy, completeness, consistency. Pay attention to results of sputum examinations that were not recorded or sputum examinations that were not carried out at the correct intervals, omission of age of patients or treatment outcomes, misclassification of previously treated cases etc.
- **Examine supplies**e.g anti-TB drugs stock management, expiry, storage conditions etc; also treatment related supplies and forms
- **Discuss with health care staff;** e.g, training needs
- **Discuss with patients:** e.g how they feel about the services they receive, their understanding of cause of TB, treatment duration, the need for adherence etc.

At the end of the visit, the team should summarize their observations, and discuss them with the TB supervisor.

The Regional TB Coordinator should visit the TB diagnostic facilities and the clinics at least every month. At the clinics, the regional coordinator should also assess their case holding practices.

### Monitoring tools

Monitoring and evaluation are key activities for assessing the performance of the programme. Recording and reporting are part of the monitoring and evaluation process. Key elements of a

Recording and Reporting System include:

- 1. Collection of individual, patient-based data
  2. Region-based cohort analysis on a quarterly basis, using standardized formats and definitions
  3. Interim reports (smear conversion) and treatment outcome reports based on quarterly diagnosis
  4. Calculation and analysis of key performance indicators
  5. Use of data at facility and region-levels for performance assessment and improvement.

Standardized forms are necessary to capture all relevant data. The following is the complete list of forms:

- 1. Register of presumptive TB cases, to record patients with a positive chronic history and measure the quality of diagnosis
  2. Laboratory Sputum Request Form, to indicate the examinations requested;
  3. TB Laboratory Register, kept at laboratories performing bacteriology tests;
  4. Patient Treatment Card, a patient-held card to record key patient information, replicating the Clinic/Hospital Card
  5. TB Treatment Card, the key data collection tool to record characteristics of the patient, the episode of TB disease, treatment progress and outcome; this is kept at the facility where the TB patient is registered
  6. TB Register is used to record key diagnostic and treatment information on each registered patient. It includes basic demographic and treatment information as well as treatment outcome. It forms the basis for cohort analysis.
  7. Transfer Form, used to ensure that vital patient information is communicated from one facility to another in an effort to improve continuity of care.
  8. Daily Treatment Supporter Card (yellow card), used by community-based treatment supporter to record directly observed treatment.
  9. er Tracing Form (pink card) used for tracing ers
  10. Referral form

## Programme monitoring

**Monitoring** programme performance to ascertain whether activities are accomplished as planned, and identification of problems should be conducted periodically in collaboration with partners. It aims to identify problems quickly so that they can be solved without delay.

### Programme indicators:

The programme indicators are basically categorized as follows:

**Impact indicators:**

The three impact indicators include:

1. TB Incidence
2. TB Prevalence
3. TB Mortality

**Outcome indicators**

These include:

1. TB Case notification for all cases
2. TB case notification rate for new
3. TB treatment success rate

**Output indicators:**

As may be dictated by the various activities in the National TB Control Programme annual operational plans.

#### Cohort Analysis

Each group of patients diagnosed and registered for treatment during a particular period of time (e.g., a quarter) is called a cohort. The case detection and outcome of treatment of each cohort is recorded every quarter.

The most important cohort to monitor is that of sputum smear-positive cases, which is used as the major indicator of the programmer’s quality. It is also useful to monitor the outcome of other forms of TB such as smear-negative PTB and EPTB. These patients tend to have much higher mortality than smear-positive PTB. With the advent of ARVs, it is expected that mortality in these patients will decrease.

Cohort analysis is the key management tool for evaluating the effectiveness of NTCP performance. By identifying problems, it allows corrective measures to be taken. Evaluation of treatment outcomes and trends must be done peripheral, regional and central levels so that appropriate measures can be taken at the appropriate level. The Regional TB coordinator should perform cohort analysis every three months and at the end of every year.

The information collected by cohort analysis is in the form of quarterly reports. The electronic TB register is in place in all the TB diagnostic facilities. It has a built-in analysis programme that automatically produces the whole cohort analysis (case finding and treatment outcome). It is important to note that the information that is entered into the electronic TB register is obtained from the manual TB registers, which must therefore be correctly and completely filled. All reports prepared from the Tuberculosis Register are only as accurate as the information recorded in the manual TB register.

#### Quarterly Report on Case Finding

This report is completed by systematically counting the number of cases recorded in the Tuberculosis Register within the quarter that has just ended. Any case classified as “Transfer in” or “Other” is not reported.

New tuberculosis cases (all forms) should be analysed separately from previously treated cases. Likewise, new smear-positive PTB cases should be analyzed separately from previously treated cases, and should be recorded by age groups and sex.

#### Quarterly Report on Treatment Outcome

Evaluation of outcome at the end of treatment should be done three months after all patients in the cohort have had time to complete treatment, a period of about 15 months. The treatment outcomes of interest are those of sputum smear positive cases.

Smear positive PTB cases are analyzed by category: new cases and retreatment cases (relapse, treatment after and treatment after failure). Those cases recorded, as “Transfer in” must not be included in the report, as the results of treatment of such cases should be sent to the unit from which the patient was transferred and reported in that unit.

The total number of cases evaluated within each category (according to the type of case and treatment regimen) should be equal to the number entered in this section, obtained from theQuarterly Report on Case-finding. Where the number is different, an explanation must be provided.

In completing the report, the information should be obtained from the Tuberculosis Register under the section entitled “Results of treatment”. The result for every case should have been recorded at this point. Where more than one result occurs for a single patient, the result that will be recorded is that event which occurs first. That is to say, if an. individual remained smear positive at 5 months but subsequently died (or lost to follow up; or was transferred out) the patient must be evaluated as smear positive (failure).

At the time of preparation of the report, if no other result is recorded, the patient must be reported as ***‘Not evaluated’.*** When a patient has been transferred to another unit to continue treatment, the outcome of the treatment at the unit to which the patient was transferred should be obtained and entered into the register at the referring unit. Patients should only be recorded as “Transferred” only when their treatment outcome is not known.

#### Information Flow

The TB supervisor compiles the quarterly reports including cohort analysis, which is transmitted to the regional TB coordinator. Peripheral quarterly reports should be transmitted to the regional TB coordinators, who should ensure that they are correct, complete and consistent.

The Regional TB coordinator should then compile cohort analysis for the region, give feedback to the peripheral units and transmit this report to the national level. The NTCP central unit should then compile a national cohort analysis from these data and provide feedback to all programme levels.

Regular supervisory visits and annual impact assessment meetings are also crucial for monitoring programme performance. Reports must be compiled to document all the meetings and field visits, using a standard supervisory checklist.

## Programme evaluation

**Programme Evaluation should be conducted at the end of a plan period to** assess progress towards operational targets and epidemiological objectives. The evaluation should ensure measurement of all programme indicators, such as percentage of patients cured, to assess progress in achieving targets and objectives.

###

## Reporting and Recording system

The adequate care of tuberculosis cases requires that records be kept on each individual patient, with periodic reporting of the results of case-find­ing and of treatment. This is essential to ensure that the patient is correctly treated and that adequate supplies of essential materials are provided. In addition, the information that is routinely collected and reviewed allows problems that may arise with the management of the patients and of the system to be identified. The documents used to record and report the care of the patients should be simple, clear and kept to the absolute minimum that is required for adequate care. The following description provides a guide for the recording of patients as they appear to the health facility, and comprises the minimum number of records and reports necessary to ensure the proper care of the patients.

Table 16: Recording and reporting formats used in the National TB Programme

| S/No. | **M&E format** | **Data requirement** | **Level** | **Responsible** | Frequency of entry |
| --- | --- | --- | --- | --- | --- |
| **1** | Register of Presumptive TB cases | Records of patients presenting with chronic cough | Health facility | General Health Care staff | Daily |
| **2** | General Laboratory request form that includes the Sputum Examination request section | Results of AFB smear microscopy  Request for DST | Health facility | General Health Care staff | Daily |
| **3** | TB Laboratory register | Results of AFB smear microscopy | Laboratory | Laboratory Scientist or technician | Daily |
| **55a** | TB Treatment Card | Patients treatment records and progress | Health facility | General Health Care staff | Daily |
| **5b** | Childhood TB monitoring card | Child’s treatment records and progress | Health facility | General Health Care staff | Daily |
| **6** | TB appointment Card | Daily patient’s treatment records | Health facility Home | General Health Care staff | Daily |
| **7** | TB referral/ Transfer Form | Patient’s up to date treatment status | Health facility Home | General Health Care staff | Based on need. |
| **8** | TB Treatment Register | Patient’s daily treatment records | Health facility | General Health Care staff | Daily |
| **9** | TB screening Form | Records symptom screening among populations vulnerable to tuberculosis | Facility | General Health Care staff | Daily |
| **10a** | Quarterly Report on TB Intensified Case finding form | Report on TB screening among PLWA/vulnerable groups in a quarter by category. | BMU/National | Regional TB Coordinator | Quarterly, Annual |
| **10b** | Quarterly Report on TB Case finding form | Report on TB cases detected in a quarter by category. | BMU/Regional/National | Regional TB Coordinator | Quarterly, Annual |
| **11** | Quarterly Report on Sputum Conversion form. | Report on treatment outcome of TB cases started on treatment 3-6 months earlier. | BMU/Regional/National | Regional TB Coordinator | Quarterly, Annual |
| **12** | Quarterly TB Cohort Report form. | Report on treatment outcome of TB cases started on treatment 12-15 months earlier. | BMU/Regional/National | Regional TB Coordinator | Quarterly, Annual |
| **13** | TB drugs Returns form. | Quarterly Regional or State drug utilization and request | Health facility/ Regional/National | Pharmacist | Quarterly, Annual |

# ADVOCACY, COMMUNICATION, SOCIAL MOBILISATION

## Introduction

Advocacy, communication, and social mobilization (ACSM) strategies can be most effectively concentrated to help address four key challenges to TB control **at country level**:

- Improving case detection and treatment adherence
- Combating stigma and discrimination
- Empowering people affected by TB
- Mobilizing political commitment and resources for TB.

## Communication as an overarching theme

The term “communication” is overarching one meaning the processes people use to exchange information about TB. All communication activities make use of some form of mediator channel of communication (e.g. mass media, community media, interpersonal communication). While much of the communication effort on TB is concerned with transmitting series of messages to people affected by TB, nearly all communication practitioners stress that to be effective, communication should be understood as a two-way process, with “participation” and “dialogue” as key elements.

***Programme communication to inform and empower***

In the context of TB control, *programme communication* is concerned with informing and creating awareness among the general public or specific populations about TB, and empowering people to take action.

Programme communication also works to create an environment through which communities, particularly affected communities, can discuss, debate, organize, and communicate their own perspectives on TB. It is aimed at changing behaviours (such as persuading people with symptoms to seek treatment) but can also be used to catalyze social change(such as supporting community or other communication-for social-change processes that can spark debate, and other processes to shift social mores and barriers to behavior change).

## Advocacy to change political agendas

Advocacy denotes activities designed to place TB control high on the political and development agenda, foster political will, increase financial and other resources on a sustainable basis, and hold authorities accountable to ensure that pledges are fulfilled and results achieved.

*Policy advocacy* includes data and approaches to advocate to senior politicians and administrators about the impact of TB at the national level, and the need for action.

*Programme advocacy* is used at the local, community level to convince opinion leaders about the need for local action.

*Media advocacy* generates support from governments and donors, validates the relevance of a subject, put issues onto the public agenda, and encourage the media to cover TB-related issues regularly and in a responsible manner*.*

## Social mobilization to build partnerships

Social mobilization is the process of bringing together all feasible and practical intersectoral allies to raise awareness of and demand for a particular programme, to assist in the delivery of resources and services and to strengthen community participation for sustainability and self-reliance.

“Allies” include decision - and policy - makers, opinion leaders, nongovernmental organizations (NGOs) such as professional and religious groups, the media, the private sector, communities and individuals. Social mobilization generates dialogue, negotiation and consensus, engaging a range of players in interrelated and complementary efforts, taking into account the needs of people.

To achieve TB control advocacy objectives, the main obstacles to TB control and the tools available for overcoming them should be identified. Some of the constraints in Swaziland are:

- The DOTS strategy is not being implemented
- Financial and human resources are lacking
- Prevalence of MDR TB is increasing
- Prevalence of HIV/AIDS is increasing and directly affecting TB morbidity and transmission of TB infection.

There is a need to identify ways of overcoming these constraints and why do they exist?

## Selection of Advocacy Strategies and Tactics

**According to the WHO^^[[5]](#footnote-5)^^; there are four priority advocacy strategies. They are:**

- A media strategy
- A publications strategy
- Coalition-building and working with NGO’s
- An insider strategy

Deciding which strategy or which combination of strategies to use should take into account the benefits and risks, the time frame, and the expertise and financial resources needed for effective implementation.

### Media strategy

Media coverage should focus on the country’s most important media (press, radio and television). Tactics might involve:

- Using World TB Day as an opportunity for a media event;
- Holding news conferences
- Conducting media tours to DTDs.
- Developing background materials for the media such as fact sheets
- Purchasing advertising space and placing newspaper supplements
- Using articulate and eloquent TB patients as speakers in media interviews and visits.

### NTP Publications strategy

Activities should include:

- Publishing quality and informative TB programme reports;
- Publishing TB programme brochures including reading materials for the community
- Producing a newsletter
- Disseminating WHO TB reports
- Developing and distributing Information, Education and Communication (IEC) materials for the community and patients

### Coalitions and working with NGOs

**Activities could include:**

- Encourage grassroots participation in TB control efforts
- Involve community organizations
- Coordinate education, communication, training and advisory activities with those of organizations working on related issues such as AIDS, asthma, anti-smoking and diabetes;
- Approach corporations, professional associations and workers unions for political and financial support for TB control;
- Conduct a preparatory World TB Day workshop for NGOs and professional associations;
- Request prestigious personalities from scientific circles and performing arts celebrities to serve as advocates.
- Establish an advocacy steering committee that should include representatives from the Health Education Unit.

###

### Insider strategy

The “Insider strategy” refers to making direct contact with the principal “targets” of advocacy activities, particularly politicians, government officials at decision making level.

Activities should include:

- Networking and lobbying
- Arranging meetings, workshops and seminars to reach key people
- Maintaining regular and frequent communication with international cooperation and technical agencies.

## Message Development and Presentation

Messages should take into account the audience, i.e. the key persons for whom they are intended.

**Message content:**

- Ensure that the message is technically sound and defensible;
- Always emphasize the severity of the TB problem and that DOTS is the best solution;
- Provide examples of DOTS successes and demonstrate its advantages;
- Emphasize the threat of MDR TB

**Resource mobilization**

Advocacy implies some budgetary risk. But failure to undertake any advocacy activity will probably mean that the NTP continues to operate at the same or even a lower level of funding.

In order to secure financial resources:

- Assign regular budget funds for advocacy activities;
- Investigate and understand potential donors.

In order to secure human resources:

- Involve those MOHSW departments (e.g. AIDS programme, Health Education) that have expertise in advocacy and related issues;
- Use WHO to guide the programme

## Role of NGOs , Private Sector and communities in TB control and involvement in social mobilisation

The government of Swaziland encourages Public-Private Partnership (PPP) especially in the health sector with the aim of complementing the government efforts to expanding access to quality health care to the population. The guiding principle for the partnership and collaboration is to strengthen ownership, ensure transparency and social responsibility. The involvement of the private sector and NGO’s in TB control activities is very crucial. However, there is need for strong coordination to ensure synergy and complimentarity in implementation of activities. It is necessary to ensure that partners are adequately oriented in the Stop Strategy, follow the national policy with respect to standard case management as well as the NTP information system.

Stakeholders in diagnosis and treatment of TB patients should also be involved in all activities of tuberculosis control, including training, monitoring and evaluation. The main output indicators of the involvement of other partners is the number and proportion of private hospitals, clinics and individual doctors, who notify new cases, implement DOTS and report treatment outcomes in a collaborative agreement with the MOH.

At the community level, Community-based organizations (CBOs) have a significant role to play with respect to:

1. Supporting patients throughout treatment until cure
2. Patient, family and community education
3. Case finding
4. Lobbying for government commitment to TB control
5. Increasing accountability of local health services to the community.

Based on the available information on the key lessons learned from community contribution as documented by by WHO^^[[6]](#footnote-6)^^, the determinants for success includes:

1. Good collaboration between the general health services, NTP and the community groups;
2. Good education of the TB patients and their family members;
3. Training of community members and the health services staff;
4. A system of regular supervision of community members by NTP staff.

The main challenges identified include: identification of the leadership responsible for managing the change process and of the appropriate community group; maintaining adequate level of community motivation; and ensuring good communication links between the different elements of service provision.

NGOs often play an important role in mobilizing community contribution, as they are usually closer to the community than the formal health care sector. However, regular supervision and monitoring is still required to achieve the desired impact. Before the programme decides to involve the community in TB care, it is necessary to ensure that a system of follow-up is established. Regular monitoring and evaluation of treatment outcomes needs to be conducted. The key areas that communities can contribute in TB care are:

- Direct Observation of Treatment
- Support and motivation of patients
- General support and home visits
- Case detection
- tracing
- Increasing community awareness

# ANNEXES

## ANNEX 1: TB Programme Organogram

**
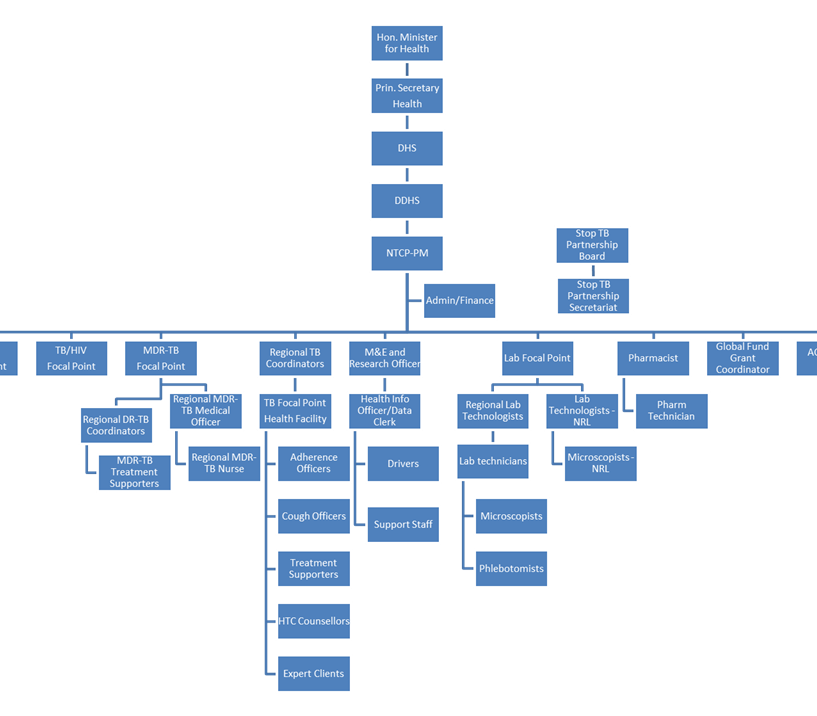
**

## ANNEX 2: Stop TB Partnership Organogram

## ANNEX 3: SPUTUM COLLECTION

Clean containers that are free from paraffin and other waxes or oils should be used for sputum collection. The containers should have an opening that is 2 cm or more across and have at least 50 ml capacity. They should be leak proof and rigid to avoid crushing during transport to the laboratory. An identification label must be placed on the side of the container and should include the *patient’s name number*, the *date of specimen collection*, the *name of the health facility* sending the specimen, and the *test requested*. Fill in the relevant details on the label before the specimen is submitted.

Sputum collection should be conducted in open air and away from other people. The following instructions should be given to the patient in order to obtain sputum of good quality:

- Rinse the mouth before producing the specimen.
- Take two deep breaths, holding the breath for a few seconds after each inhalation and then exhaling slowly.
- Breathe in a third time and forcefully blow the air out.
- Breathe in again and then cough.

Specimens must be sent to the laboratory as soon as possible after collection. If this is not possible, specimens should be refrigerated but not frozen. During transport, specimens should be surrounded by absorbent packing and should be kept cool. Exposure to sunlight should be avoided because mycobacteria are killed by ultraviolet light. For each sputum specimen collected, a laboratory request form should be filled to accompany the specimen.

## ANNEX 4: Prerequisites for implementation of XPERT MTB/Rif

| **1. Key prerequisites before country implementation of the Xpert MTB/RIF assay Prerequisite** | |
| --- | --- |
| **Epidemiological**  **data** | 1. Data available on prevalence of MDR-TB and HIV-associated TB to allow for decision making on **prioritizing** placement of the technology and **optimising** use of Xpert MTB/RIF in high-risk patient groups |
| **Diagnostic policy reform** | 2. Plan to modify existing diagnostic algorithms as part of the NTP strategy to introduce Xpert MTB/RIF testing. |
| **Laboratory network** | 3. Existing capacity and referral network to provide quality assured laboratory services with: **a)** culture and DST to determine resistance to first- and second-line drugs at central level (at least), quality assured through an established link with a Supranational Reference Laboratory; **b)** sputum smear microscopy for TB testing and treatment response monitoring; **c)** culture to monitor response to MDR-TB treatment. |
| **Laboratory workload** | 4. Potential number of samples from high-risk groups for Xpert MTB/RIF testing in the facility where implementation is intended ranges 10-20 a day or 2000-4000 annually, in order to ensure optimal efficiency3 |
| **Infrastructure** | 5. Stable electricity supply in the facilities where implementation is intended or sufficient measures to ensure uninterrupted supply (generator, solar panels, battery/UPS backup, etc.) |
| 6. Secure premises for the equipment to prevent theft of the GeneXpert unit and the computer/laptop. | |
| 7. Adequate storage of cartridges at recommended temperature range (2-28°C). | |
| 8. Appropriate measures to prevent ambient temperature exceeding 30°C in the room where equipment will be installed (e.g. ventilation, air conditioning). | |
| **Bio-safety** | 9. Bio-safety requirements similar to sputum smear microscopy. |
| **Personnel** | 10. 1-2 staff per site with basic computer literacy and knowledge of laboratory registers who can be trained to perform the testing and equipment maintenance. |
| **Treatment capacity** | 11. Sufficient capacity for treatment of identified TB and MDR-TB patients is available and is in line with international recommendations. |
| **Financing** | 12. Secure funding from national budget or donors/partners |
| **Procurement** | 13. Country importation procedures allowing for procurement of both equipment and consumables (regulatory registration or waiver) and exchange of modules for annual calibration. |

## ANNEX 5: Key recommended actions at country level for Xpert MTB/Rif implementation

| **2. Key actions necessary at country level for implementation of Xpert MTB/RIF assay Action** | |
| --- | --- |
| **Policy reform update** | 1. Incorporate Xpert MTB/RIF testing in the NTP diagnostic strategy and algorithms, including identifying placement of Xpert MTB/RIF at the appropriate level of diagnostic network. Identify appropriate pre-test screening strategies where necessary. |
| **Logistics** | 2. Identify adequate premises for the equipment (as per prerequisites 5,6) |
| 3. Allocate storage for cartridges (as per prerequisite 7) | |
| 4. Identify procedure for cartridge disposal (for example, incineration) as part of current laboratory waste disposal plan. | |
| **Procurement** | 5. Register the GeneXpert system and Xpert MTB/RIF assay OR obtain waiver for importation |
| 6. Forecast needs based on expected demand and period of implementation | |
| 7. Calculate first and subsequent orders for the period of implementation | |
| 8. Quantify buffer stock (at least to cover 3 months of expected workload) taking into account the shelf life of cartridges. | |
| 9. Place order for equipment and cartridges to manufacturer directly or certified distributor insisting on preferential pricing where relevant. | |
| Financing | 10. Secure sustainable funding from national budget or donors/partners to ensure continued use of the Xpert MTB/RIF testing. |
| **Training** | 11. Identify and train staff to perform Xpert MTB/RIF assay |
| 12. Train staff to ensure timely referral and/or proper treatment, infection control measures and contact tracing. | |
| **Reporting** | 13. Adapt request and reporting forms to include Xpert MTB/RIF result |
| 14. Develop system for reporting to the clinic on the same day when results are available | |
| 15. Develop system for regular reporting to WHO (quarterly or semi-annual) | |
| **Validation** | 16. Use GLI (Global Laboratory Initiative) validation kit provided by Cepheid after first installation and after each module calibration. Report results to WHO/GLI. |
| **Maintenance** | 17. Send each module for annual calibration or after performing 2,000 tests on the same module. |

## ANNEX 6: BASIC GUIDE TO CXR READING

To evaluate a CXR properly the following needs to be done:

1. Name of the patient and date should be double checked to ensure one is reading the right xray.
2. Locate the right and left sides of the xray
3. Assess the quality of the x-ray by commenting on the exposure – one should be able to see the thoracic vertebrae and vessels behind the heart shadow.
4. Ensure that there is correct positioning, by assessing whether both left and right clavicles and scapulae are symmetrically located i.e. in line with each other.
5. Make sure there is good inspiration – one should see the posterior 5 posterior ribs in the film.
6. Then with a good knowledge of the normal anatomy and bearing in mind that air is black on xray and solid organs, fluid and bones appear white; one proceeds to look at :
   - The heart – the central white shadow, assess it for its size relative to the size of the chest, its shape, as well as its location (most of the heart should be in the left lung field normally).
   - The lungs – on each side of the heart, normally are black on the xray. One should assess them for good inspiration, any abnormal white shadows (infiltrates) bearing in mind that the blood vessels at the hilum appear white as well. One should also assess the angle between the chest wall and the diaphragm, it should be an acute angle, if it is fuzzy or filled with white shadows there may be effusion. If the lung field is too black with no blood vessels visible, there may be the possibility of a lung collapse or no lung at all.
   - The bones – comment on the number of posterior ribs present as it gives an indication of the inspiratory effort, as well as hyperinflation (as in COPD). Then comment on the appearance of the bones themselves i.e. any undue dark lesions (osteolytic lesions) or white lesions (osteoblastic lesions); any fractures present.
   - Trachea and main bronchi for any obstruction or deviation.
   - Soft tissue – breast shadows, axillae and the skin shadow for any masses or fluid filled cavities.
   - The gastric bubble is usually on the left. And also assess for air under the diaphragm, between diaphragm and liver/stomach. Note that the right diaphragm is slightly higher than the left due to the underlying liver.

## ANNEX 7: TUBERCULIN SKIN TEST (TST)^[[7]](#footnote-7)^

Thetuberculin skin test (TST) is administered to detect the presence of Mycobacterium tuberculosis, the bacterium that causes tuberculosis (TB). It’s sometimes call Mantoux or PPD.

Material needed:

- 1 vial of purified protein derivative (PPD). Once the vial is open, it lasts for one month, so make sure you write on the label date you open the vial and expiry date.
- 1ml syringe
- A pen

Procedure:

1. Use a 1mL syringe to aspirate out 0.1mL of PPD
2. Inject PPD intradermally on the volar surface of the forearm (the bevel is visible under the skin). Position the syringe at a 10-15° to the forearm and insert just below the epidermis (about 2 mm).


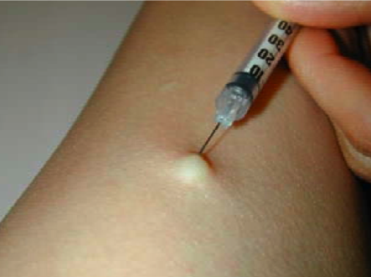


1. Remove the needle quickly. Do not massage or use dressing. A well-defined bleb of 6-10mm in diameter should be formed if injected correctly. If the bleb is <6mm, repeat the process 2.5cm from the first site.
2. Mark down the site, date and time of injection, both on the forearm and in patient’s record.
3. After 48 to 72 hours, read the test result by marking down the transverse diameter of the induration, not erythema, by Sokal’s ballpoint method.


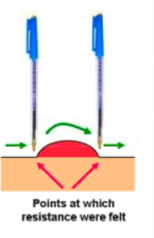


1. Measure the largest transverse diameter of induration (palpable, raised, hardened area of swelling) and note down in millimeters (mm). Do not just write test result positive or negative.

**A *positive result* is *5mm of induration in HIV positive children* or *10mm of induration in HIV negative children*.**

## ANNEX 8: FINE NEEDLE ASPIRATION (FNA)

Fine needle aspiration is a very useful way of diagnosing tuberculosis when enlarged lymph nodes are present.FNA is a rapid, safe and cost-effective diagnostic test to diagnose EPTB. It’s recommended especially in children with persistent LN not responding to DS treatment. If pus or necrotic material is aspirated, it should be sent for culture/GenXpert**.**

Material needed:

- 10ml syringe
- 22 or 23 gauge needle
- Cytology slides labeled with the patient’s name and surname
- Fixative spray
- Adequate laboratory form
- Specimen cup in case we send for culture.

Perform as follows:

Site preparation

- Wash hands and aseptically put on sterile gloves.
- Sterilize a wide area surround the puncture site with either 0.05 percentchlorhexidine or 10 percentpovidone-iodine solution.
- Allow adequate time for the skin preparation to dry.

Procedure:

1. Immobilize the mass with one hand and insert the needle into the mass
2. Pull back the plunger to create a vacuum of no more than 1 ml
3. Aspirate the mass by moving the needle in a fan-like fashion throughout the mass.
4. When there is material in the hub, release the suction and withdraw the needle
5. Remove the needle from the syringe, pull 8-10ml of air into the barrel, reattach the needle and push the plunger down while placing the tip of the needle in the slide
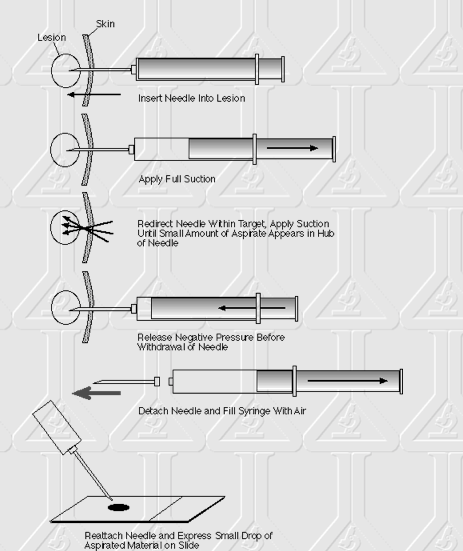
^[[8]](#footnote-8)^

## ANNEX 9: GASTRIC ASPIRATE PROCEDURE FOR CULTURE OFM. *tuberculosis^[[9]](#footnote-9)^*

It is a simple procedure to obtain gastric material in order to perform smear and culture/GenXpert for children who can’t produce sputum.

Ideally 3 gastric aspirates should be collected whenever possible for culture (to obtain the highest yield). If the child is admitted, samples can be collected on 3 consecutive days. If the child is not admitted, 1 or 2 aspirates can be done on the same day, at the clinic, with 5-6 hours of difference and repeated the following visit.

Material needed:

- N95 masks
- A sheet to wrap the child
- 8-10 french or larger feeding tube with a 20ml syringe
- Pen/marker
- Sterile water with a 20 ml syringe
- Specimen cup
- Sodium Bicarbonatum 8,5% (IV from the pharmacy) with a needle and a 10ml syringe
- Culture request form

Procedure:

1. Describe the procedure to the family beforehand. Explain the procedure is brief, uncomfortable but not painful. Advise that a negative result does not exclude TB and that the results can take up to 6-8 weeks.
2. Patient should be NPO at least 5-6 hours. If the child is admitted, the procedure should be done in the AM before he starts ambulating.
3. All HCW involved in the procedure are encouraged to wear a N95 mask and good ventilation is advisable in the room.
4. Measure the expected distance from nose to stomach. Strech the tube from the tip of the nose, around the ear and down to the stomach. This is the distance the tube should be inserted into the stomach. Mark the spot on the tube.
5. Immobilize the child with a sheet or with his own upper clothes.
6. Moisten the tube in the child’s mouth to avoid bacteriostatic lubricants.
7. Pass the tube through the child’s nose to the stomach. (If the child does not swallow the tube, take a breath, pull your mask away, puff in the child’s face and replace your mask. This maneuver frequently elicits a swallow-look to make sure that the tube is not coiled in the mouth. Children frequently vomit at this stage, so be prepared to collect any emesis in a specimen cup. It may contain the mucous you’re looking for!)
8. Once the child swallows the tube, pass the tube quickly down into the stomach. Stop when the pen mark on the tube reaches the tip of the nose. *RARELY, the tube will pass into the airway. If the child has any respiratory distress or a muffled cry, the tube is misplaced. Remove the tube immediately*
9. When the tube reaches the pen mark, aspirate the stomach contents with the syringe and place the gastric aspirate in the specimen cup.
10. If less than 5-10cc of mucous returns, re-position the tube and/or the child in order to look for the pool of mucous. While continuing to gently aspirate with the syringe, pass the tube further along several centimeters and try rolling the child up onto his/her side.
11. If still < 5-10cc of gastric contents have been aspirated, instill 20-30cc sterile water into the tube. *BEFORE instilling anything into a nasogastric tube, always check the position on the tube inserting 10cc of air quickly while listening with a stethoscope directly over the stomach. If there is doubt of the positioning, withdraw the tube.*
12. Re-aspirate the contents of the stomach. If still there is no significant yield, try advancing or withdrawing the tube and changing the child’s position in order to find mucous. Continue to aspirate as you withdraw the tube.
13. Place the material obtained in the cup.
14. Insert the same quantity of sodium bicarbonate as gastric material you have, to neutralize the sample.

## Assure sample is well labeled and all forms correctly filled before sending the material to the laboratory.ANNEX 10: INDUCED SPUTUM IN CHILDREN^[[10]](#footnote-10)^

**Sputum Induction in Children**

**Precautions:** *Only HCW who have been trained in proper, safe technique should perform sputum induction.* Patients must be observed at all times during sputum induction

Sputum induction should not be performed in children with:

- Acute (active) asthma
- Any signs of moderate to severe respiratory distress
- Wheezing
- Abnormal vital signs
- Epistaxis
- Pneumothorax
- Fractured ribs or other chest trauma
- Recent eye surgery

**Infection control:**

Sputum induction produces coughing so it is likely that infectious droplets, if present, will be expelled into the room air. *Sputum induction must be performed in a well ventilated area.* The HCW must wear an N95 respirator throughout the procedure and disposable gloves when handling the sample.

If outside, determine the wind direction before beginning the procedure. The patient must always be located downwind of the HCW. If inside, open doors and windows and use fans to direct airflow away from the HCW

Nebulizers and tubing must be sterilised after every use with (precept or glutaraldehyde) for 15-20 minutes

Material needed:

- N95 respirator for the HCW
- Nebuliser machine
- Sterile hypertonic saline (3-5%)
- Salbutamol and 0.9% NaCl solution (normal saline)
- Suction machine and catheter or Yankaeur
- Pulse oximetry machine (if available)
- Oxygen cylinder
- Goggles
- Small volume nebuliser
- The compressor
- Infra-red light
- Sputum traps (mucous extractor)
- Disinfectant (precept or glutaraldehyde)
- Disposable gloves
- Completed laboratory request form with patient details
- Sterile sputum collection container identified with the patient details and culture form adequately filled

Procedure:

**NPO requirements:** The child should be NPO for 6 hours prior to the procedure. Do not perform sputum induction if the child has eaten within 3 hours prior to the procedure. Medication with a small amount of liquid is acceptable.

**Preparations:**

- Set up area ahead of time to minimize the anxiety level of the child
- Keep syringes and needles out of sight
- Preload the equipment with salbutamol and normal saline
- Keep a suction catheter or Yankaeur nearby in case of vomiting
- Fill out laboratory request forms
- Have the caregiver hold the child during the procedure. If this is not possible, an assistant should hold the child.
- Position the child in the upright or semi-upright position
- Hold infants supine in the feeding position
- Stand or sit where you can clearly observe the child and all of the equipment

**Induction procedure:**

1. Run correct dosage of Salbutamol in normal saline (0.9% NaCl solution) for 3-5 minutes.
2. Add hypertonic saline (3-5% NaCl solution) to the solution and continue nebulisation for at least 10 minutes:
   1. If the child coughs during this time and produces a sputum, you’re done.
   2. If the child does not produce a specimen within 10 minutes, insert the suction catheter, nasopharyngeal airway (NPA), or oropharyngeal airway (OPA) to stimulate cough.
3. When there is adequate sputum in the oronasopharyngeal area, insert the catheter from the sputum trap (either alone or through an airway).
4. Apply vacuum until at least 2 ml of sputum is collected in the sputum trap. Start at 15-20kPa pressure and increase only if needed.
5. Ensure that the sputum collection container is tightly sealed and labeled.

**Stop induction if:**

- Respiratory distress including increased respiratory rate, wheezing, laboured breathing, chest wall retractions, nasal flaring or cyanosis
- Profuse sweating
- Nausea or vomiting
- Light-headedness, dizziness or loss of consciousness

**After induction:**

- Monitor the child for several minutes. If pulse oximetry is available and is below baseline or there are signs of respiratory distress give oxygen and suction excess sputum from the airway.
- Educate the caregiver that coughing may be more frequent within 24 hours of the procedure.
- Assure all samples are labeled and the forms for culture correctly filled.
- Keep samples out of direct sunlight.

In case there is no nebuliser machine available, nasopharyngeal aspirates can be done without nebulisation. In this case, clapping of the patient for 10 minutes is recommended, to facilitate the secretion of the mucous. The rest of the procedure is the same as the same as indicated above.

The committee should meet monthly.

## ANNEX 11: PERFORMINGTHORACENTESIS

INDICATION

Perform thoracentesis on all patients with pleural fluid unless;

- There is a small amount of pleural fluid

or

- Patient has clinically obvious congestive heart failure without atypical features.

Atypical features (implying additional complications to the CHF or an incorrect diagnosis) that would justify a thoracentesis include:

- A unilateral effusion, especially if it is left-sided

- Bilateral effusions that are of disparate sizes

- Pleurisy

- Fever

- Normal cardiac silhouette on chest radiograph

- An effusion that does not resolve with heart failure therapy

- Known chronic kidney failure

CONTRAINDICATION

- Active skin infection at the site of needle insertion

- Patient is actively bleeding or has received anticoagulation

- Very small amount of pleural fluid

MATERIAL

- Lidocaine 1 percent

- Sterile syringe with 25-gauge needle for lidocaine injection

- 50 mL syringe for fluid collection

- Sterile gloves

- Sterile drapes

- Povidone-iodine solution

- Sterile sponges or 4 x 4s for preparing puncture site

- 20 or 22-gauge needle 1.5 inches in length.

PROCEDURE

1. Patient positioning

- Patient should be sitting upright with their arms on a solid surface (such as a table with a pillow on it). If unable to sit, patient can assume a lateral recumbent position.

2. Site selection

The site of needle insertion must meet all of the below criteria:

- One to two interspaces below the level at which breath sounds decrease or disappear on auscultation, percussion becomes dull, and fremitus disappears

- Above the ninth rib, to avoid subdiaphragmatic puncture

- Midway between the spine and the posterior axillary line, because the ribs are easily palpated in this location.

3. Site preparation

- Wash hands and aseptically put on sterile gloves.

- Sterilize a wide area surround the puncture site with either 0.05 percentchlorhexidine or 10 percentpovidone-iodine solution.

- Allow adequate time for the skin preparation to dry.

- Using a 25-gauge needle sterilize the area with local anesthetic . Advance towards the rib, and direct towards the superior edge of the rib , intermittently pull back on the plunger of the syringe as the needle is advanced to make sure you are not hitting a vessel. Pleural fluid return indicates needle is inserted in the pleura. Inject more anesthetic into the border of the rib and the pleural space and remove the needle.

4. Needle insertion and aspiration

Add 1 mL of 1:1000 heparin to a 50 mL syringe . Attach the syringe to a 22-gauge needle. Advance through the anesthetized region until reaching pleural space, trying to passing on the superior edge of the rib. Remove 30 to 50 mL of pleural fluid. Send to the laboratory for cell count, chemistry, AFB and culture, if applicable.

• No fluid return, “dry specimen”, signifies incorrect needle placement, thick pleural fluid, or use of an inappropriately short needle. The needle can be withdrawn and reinserted in a slightly different angle if the patient tolerated the initial dry tap.

• Aspiration of air means the lung was punctured. Obtain a chest x-ray to rule out pneumothorax. Consideration insertion of chest tube if pneumothorax is severe.

• Aspiration of small amount of blood implies the needle was inserted inferior to the effusion.

5. Clean the area with betadine and apply sterile dressing.

COMPLICATIONS

Pneumothorax, infection and empyema, allergic reaction to the anesthetic, liver or spleen puncture, seeding of needle tract with tumor.

## ANNEX 12: PERFORMING LUMBAR PUNCTURE IN ADULTS

INDICATIONS

Non-Urgent diagnosis:Tuberculous meningitis

CONTRAINDICATION

- Patients suspected of having an epidural abscess in the lumbar area.

- Patients with known bleeding disorders

MATERIAL

- Lidocaine 1 percent without epinephrine

- Sterile 3 mL syringe with 25-gauge needle for lidocaine injection

- Four sterile collecting tubes

- Sterile gloves

- Sterile drapes

- Povidone-iodine solution

- Sterile sponges or 4 x 4s for preparing puncture site

- Manometer (typically used in patients older than two years of age)

- 20 or 22-gauge styleted spinal needle.

PROCEDURE

1. Patient positioning

Preferred position is the lateral recumbent. It can also be performed when patient is sitting upright. If in lateral recumbent, instruct the patient to remain in fetal position with the neck, back and limbs held in flexion. The lower lumbar spine should be flexed with the back perfectly perpendicular to the edge of a bed or examining table. The hips and legs should be parallel to each other and perpendicular to the table. Pillows placed under the head and between the knees may improve patient comfort.

2. Site selection

- Locate the highest point of iliac crest bilaterally and confirm by palpation.

- A direct line joining these points passes across 4th lumbar vertebral body.

- Locate the spinous processes of L3, L4 and L5 by palpation.

- Identify the interspaces of L3/L4 and L4/L5. Needle can be inserted in either of these two sites.

3. Site preparation

- Wash hands and aseptically put on sterile gloves.

- Clean the overlying skin using alcohol, and disinfect (e.g. povidone iodine)

- Place a sterile drape with an opening over the lumbar spine.

- Allow adequate time for the skin preparation to dry.

- Anesthetize only the skin and the underlying soft tissue, using anesthetic. Intermittently pull back on the plunger of the syringe as the needle is advanced to make sure you are not hitting a vessel.

4. Needle insertion

Prepare a 20 or 22 gauge spinal needle containing a stylet. The bevel of the needle should be facing up for the patient in the lateral decubitus position and sideways for the patient in the sitting position . Advance the needle slowly, angling slightly toward the head, as if aiming towards the umbilicus.

The stylet can be cautiously removed from time to time as the needle is advanced to look for CSF. A "pop" often is perceived as the needle penetrates the dura and enters the subarachnoid space. Flow of CSF confirms correct insertion of the needle.

5. Fluid collection

Instruct the patient to slowly straighten or extend the legs to allow free flow of CSF within the subarachnoid fluid. Opening pressure can now be measure using a manometer if available. If manometer is not available, use IV tubing attached to the back of the spinal needle and held vertically up in the air. Up to 40 mL of fluid can be removed, however, 8 to 15 mL is the amount most commonly collected in routine LP. Once CSF has been collected, the stylet should be replaced and the needle removed. Clean the area with betadine and apply sterile dressing.

COMPLICATIONS

- Post-LP headache; 10-30% of patients, 24-48 hours post procedure, exacerbated in an upright position and improved in the supine position. Can be associated with nausea, vomiting, dizziness, tinnitus, and visual changes. Treat with paracetamol, fluids and rest.

- Infection;

1. Meningitis, usually due to contaminated instruments or bad technique.

2. Osteomyelitis (rare)

- Bleeding, 1-2% can develop serious bleeding due to thrombocytopenia or clotting factor abnormalities.

- Cerebral herniation; neurologic decline either immediately or within 12 hours of LP

- Minor neurologic symptoms such as radicular pain or numbness

- Late onset of epidermoidtumors of the thecal sac

## ANNEX 13:PERFORMING LUMBAR PUNCTURE IN CHILDREN

INDICATIONS

Urgent diagnosis:Tuberculous meningitis,

CONTRAINDICATION

- Confirmed or suspected increased intracranial pressure (ICP) .

- Patients suspected of having an epidural abscess in the lumbar area.

- Patients with coagulation defects who are actively bleeding, have severe thrombocytopenia (eg, platelet counts <50,000/µL), or an INR >1.4.

MATERIAL

- Lidocaine 1 percent without epinephrine

- EMLA cream only if available

- Sterile 3 mL syringe with 25-gauge needle for lidocaine injection

- Four sterile collecting tubes

- Sterile gloves

- Sterile drapes

- Povidone-iodine solution

- Sterile sponges or 4 x 4s for preparing puncture site

- 22-gauge styleted spinal needle. The following guidelines for the appropriate length needle are based on the child's age (although a longer needle may be necessary for children who are large for their age, particularly for those closer to 12 years):

o Under two years, 1.5 inches (3.75 cm)

o Between 2 and 12 years, 2.5 inches (6.25 cm)

o Over 12 years, 3.5 inches (8.75 cm)

PROCEDURE

1. Patient positioning

The lateral recumbent or the sitting position can be used. The lateral recumbent position can be achieved by the help of an assistant placing one arm around the posterior aspect of the child's neck and the other arm under the child's knees, flexing the neck and drawing knees upward. The child's hips and shoulders should be kept perpendicular to the examining table in order to maintain spinal alignment without rotation. The gluteal crease must be aligned with the spinous processes.

Sitting position is best in children in respiratory distress, particularly infants and small children. Hold the infants in the sitting position by the help of an assistant grasping one of the infant's arms and one of the legs in each hand, while supporting the head to prevent excessive flexion at the neck. Older children should be asked to sit with their legs hanging over the edge of the examining table. They can then be flexed over a pillow with the elbows resting on the knees. An assistant should maintain alignment throughout the procedure (even in a cooperative child).

2. Site selection

- Locate the highest point of iliac crest bilaterally and confirm by palpation.

- A direct line joining these points passes across 4th lumbar vertebral body.

- Aim for the L3-L4 and L4-L5 interspaces accordingly.

3. Site Preparation

Apply EMLA cream if adequate time (30-60 min) is available for the cream to be effective. Otherwise proceed;

- Wash hands and aseptically put on sterile gloves.

- Clean the overlying skin using alcohol, and disinfect using povidone iodine. Clean a large area, including the posterior superior iliac spine.

- Allow adequate time for the skin preparation to dry.

- Place a sterile drape with an opening over the lumbar spine.

- Anesthetize the skin and the underlying soft tissue, using lidocaine. Intermittently pull back on the plunger of the syringe as the needle is advanced to make sure you are not hitting a vessel.

6. Needle insertion

Prepare a 20 or 22 gauge spinal needle containing a stylet. The bevel of the needle should be facing up for the patient in the lateral decubitus position and sideways for the patient in the sitting position . Advance the needle slowly, angling slightly toward the head, as if aiming towards the umbilicus.

The stylet can be cautiously removed from time to time as the needle is advanced to look for CSF. A "pop" often is perceived as the needle penetrates the dura and enters the subarachnoid space. Flow of CSF confirms correct insertion of the needle. Alternatively you can remove the stylet once penetrating the skin. This improves CSF fluid collection in infants.

7. Fluid collection

Slowly straighten the child's legs to increase fluid flow. 8 to 15 mL is the amount most commonly collected in routine LP. Once CSF has been collected, the stylet should be replaced and the needle removed. Clean the area with betadine and apply sterile dressing.

COMPLICATIONS

- Post-LP headache; 18-40% of children.

- Infection;

3. Meningitis, usually due to contaminated instruments or bad technique.

4. Osteomyelitis (rare)

- Bleeding, spinal hematoma

- Cerebral herniation; neurologic decline either immediately or within 12 hours of LP

- Minor neurologic symptoms such as radicular pain or numbness

- Late onset of epidermoidtumors of the thecal sac

## ANNEX 14: INFECTION CONTROL RISK ASSESSMENT TOOL

| **INFECTION CONTROL RISK ASSESSMENT TOOL** | | | | |
| --- | --- | --- | --- | --- |
| DATE:_____/______/______ | Risk for TB in the setting (0=negligible, 1=Low, 2=High, 3= Very High) | Likelihood of event occurring. 0= not likely, 1=remotely possible, 2=likely, 3= highly likely | Total  = a+b | Assessment  4-6=Very high risk; 2-3= High risk; 1= Low risk; 0=No risk |
| Exposure of HCW to a potentially infectious TB case |  |  |  |  |
| Exposure of other patients to a potentially infectious TB case |  |  |  |  |
| Inadequacy of ventilation |  |  |  |  |
| Duration of exposure to a potentially infectious case |  |  |  |  |
| Exposure of a person living with HIV (PLHIV) to a potentially infectious TB case |  |  |  |  |
| Contamination of the environment with infectious material from a TB case |  |  |  |  |
| Generation of infectious aerosol containing *M. tuberculosis* |  |  |  |  |
| Accumulation of potentially hazardous infectious waste material |  |  |  |  |

## ANNEX 15: HIV TESTING MODELS

REFERENCES

Ben J Marais, H Simon Schaaf & Peter R Donald. Pediatric TB: issues related to current and future treatment options, Future Microbiol. (2009) 4(6), 661–675

International Standards for Tuberculosis Care World Health Organization; 2006. <http://www.who.int/tb/publications/2006/istc/en/index.html>

Ministry of Health Swaziland: National Guidelines for Isoniazid (INH) Prophylaxis; 2010.

Nicol, Mark P., y Heather J Zar. «New specimens and laboratory diagnostics for childhood pulmonary TB: progress and prospects». Paediatric Respiratory Reviews 12, no. 1 (March 2011): 16–21.Francis J. Curry National Tuberculosis Centre 2007. Tuberculosis Infection Control, A practical manual for preventing TB. [www.nationaltbcenter.edu](http://www.nationaltbcenter.edu)

Paed(Stellenbosch), H. Simon Schaaf MBChB(Stellenbosch) Mmed Paed(Stellenbosch) DCM(Stellenbosch) MD, y Alimuddin Zumla BSc.MBChB.MSc.PhD.FRCP(Lond).FRCP(Edin).FRCPath(UK), eds. Tuberculosis: A Comprehensive Clinical Reference, 1e. 1.a ed. Saunders, 2009.

PIH/WHO 2010. Management of MDR-TB: A field guide. WHO/HTM/TB/2008.402a. <http://whqlibdoc.who.int/publications/2009/9789241547765_eng.pdf>

Rapid Advice. Treatment of tuberculosis in Children. WHO/HTM/TB/2010.13. <http://whqlibdoc.who.int/publications/2010/9789241500449_eng.pdf>

WHO 2006. Guidance for national tuberculosis programmes on the management of tuberculosis in children. WHO/HTM/TB/2006.371. <http://whqlibdoc.who.int/hq/2006/WHO_HTM_TB_2006.371_eng.pdf>

Stephen M. Graham, Centre for International Child Health, University of Melbourne Department of Paediatrics and Murdoch Children’s Research Institute, Royal Children’s Hospital, Melbourne, Australia. Child Lung Health Division, International Union Against Tuberculosis and Lung Disease, Paris, France Mini-symposium: Childhood TB in 2010. Treatment of paediatric TB: revised WHO guidelines

The UNION 1998: Epidemiological basis of tuberculosis control. First Edition1999: p.8.

The UNION 2009: Epidemiological basis of tuberculosis control.1999. First Edition:

WHO 2008. Guidelines for the programmatic management of drug-resistant tuberculosis. WHO/HTM/TB/2008.402. <http://whqlibdoc.who.int/publications/2008/9789241547581_eng.pdf>

WHO 2008: Implementing the WHO Stop TB strategy: A handbook for national tuberculosis control programmes. World Health Organization; 2008.WHO/HTM/TB/2008.401 <http://www.who.int/tb/publications/2008/en/index.html>

WHO 2009: WHO Policy on TB infection control in health-care facilities, congregate settings, and households. 2009. WHO/HTM/TB/2009.419 <http://www.who.int/tb/publications/2009/en/index.html>

WHO 2010. Antiretroviral drugs for treating pregnant women and preventing infection in infants. Recommendations for a public health approach. 2010 Revision. WHO/HTM/HIV/2010.

WHO 2010. Antiretroviral therapy for HIV infection in infants and Children towards universal access: Recommendations for a public health approach. 2010 Revision. WHO/HTM/HIV/2010.

WHO 2010. Guidance on ethics of tuberculosis prevention, care and control. WHO/HTM/TB/2010.16. <http://whqlibdoc.who.int/publications/2010/9789241500531_eng.pdf>

WHO 2010. Multidrug-resistant tuberculosis (MDR-TB) indicators: A minimum set of indicators for the programmatic management of MDR-TB national tuberculosis control programmes. WHO/HTM/TB/2010.11

WHO 2010. Rapid Advice on the treatment of tuberculosis in children. WHO/HTM/TB/2010.13. <http://whqlibdoc.who.int/publications/2010/9789241500449_eng.pdf>

WHO 2010: Guidelines for intensified tuberculosis case-finding and isoniazid preventive therapy for people living with HIV in resource-constrained settings. World Health Organization; 2010, pp. 14. WHO/HTM/TB 2010.

WHO 2010: Multidrug and extensively drug-resistant TB (M/XDR-TB): 2010 global report on surveillance and response. WHO/HTM/TB/2009.3. <http://whqlibdoc.who.int/publications/2010/9789241599191_eng.pdf>

WHO 2010: The Global Plan to Stop TB 2011-2015. <http://www.stoptb.org/global/plan/>

WHO 2011. Commercial serodiagnostic tests for diagnosis of tuberculosis:
policy statement. WHO/HTM/TB/2011.5

WHO 2011. Guidelines for the programmatic management of drug-resistant tuberculosis - 2011 update. WHO/HTM/TB/2011.6. <http://www.who.int/tb/challenges/mdr/programmatic_guidelines_for_mdrtb/en/index.html>

WHO 2011. Use of tuberculosis interferon-gamma release assays (IGRAs) in low- and middle-income countries: Policy Statement. WHO/HTM/TB/2011.18

WHO 2011: Global Tuberculosis Control 2011. WHO/HTM/TB/2011.16. <http://www.who.int/tb/publications/global_report/en/index.html>

WHO 2011: Rapid implementation of the Xpert MTB/Rif diagnostic test. Technical and operational ‘How to’ Practical considerations. WHO/HTM/TB/2011.2 <http://whqlibdoc.who.int/publications/2011/9789241501569_eng.pdf>

WHO 2011: Towards universal access to diagnosis and treatment of multidrug-resistant and extensively drug-resistant tuberculosis by 2015. WHO progress report 2011. WHO/HTM/TB/2011.3. <http://www.who.int/entity/tb/publications/2011/mdr_report_2011/en/index.html>

WHO 2012: Meeting Report. First meeting of the Global GLC. WHO/HTM/TB/2012.2 <http://www.who.int/entity/tb/publications/Meetingreport1stgGLCmeeting.pdf>

WHO 2012: WHO policy on collaborative TB/HIV activities: guidelines for national programmes and other stakeholders. WHO/HTM/TB/2012.1. <http://www.who.int/entity/tb/publications/2012/tb_hiv_policy_9789241503006/en/index.html>

WHO2009. Treatment of tuberculosis guidelines. Fourth edition. WHO/HTM/TB/2009.420. <http://whqlibdoc.who.int/publications/2010/9789241547833_eng.pdf>

[
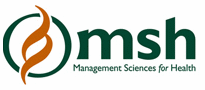
](http://www.msh.org/)


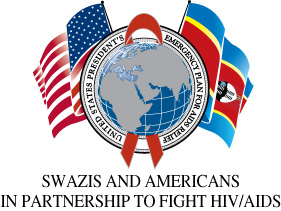


[
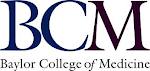
](http://www.google.com/imgres?imgurl=http://ecosystem.news-sap.com/files/2011/01/BCM_Logo_RGB_1191708351.png&imgrefurl=http://www.news-sap.com/baylor-college-of-medicine-cures-it-ailments-with-help-from-sap-communities/&usg=__9mlejhJNfy7VDR2LsGFMEzsSVAI=&h=1412&w=2992&sz=165&hl=en&start=1&zoom=1&tbnid=YrhArPXk-N4uLM:&tbnh=71&tbnw=150&ei=VVonUNHpCIS0hAfqgoDYCw&prev=/images?q=baylor+college+of+medicine+logo&hl=en&gbv=2&tbm=isch&itbs=1)
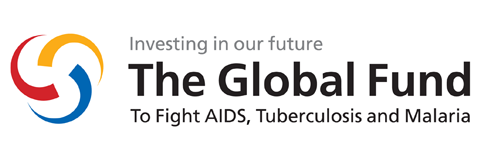


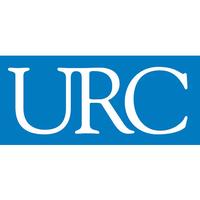


[
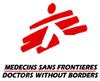
](http://www.google.co.za/imgres?imgurl=http://www.isrreports.com/images/content_images/graphic_logo_msf.gif&imgrefurl=http://www.isrreports.com/about-us/community-involvement&h=102&w=126&sz=2&tbnid=IWdrcd06xDhSFM:&tbnh=81&tbnw=100&prev=/search?q=MSF+Logo&tbm=isch&tbo=u&zoom=1&q=MSF+Logo&usg=__QvqCyKLa0bZc_MubNH3egwSKMNA=&docid=SgBi2fn67Pc81M&sa=X&ei=5f5NUPXeFIGy8QTVwICgDQ&ved=0CCsQ9QEwAw&dur=2951)
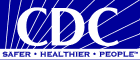

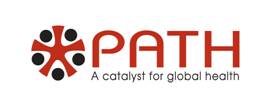


World Health Organization. 2012.WHO policy on collaborative TB/HIV activities: guidelines for national programmes and other stakeholders. Geneva, WHO

1. Health Centres in general have between 20-60 beds and should have a Public Health Unit (PHU). It is unclear whether they should have a functional operating theatre. There are five HCs in the country (see map). [↑](#footnote-ref-1)
2. Clinic Type A does not have a without maternity unit, while Clinic Type B has a maternity unit. [↑](#footnote-ref-2)
3. WHO Geneva: Laboratory services in TB Control : Culture Part III 1998 [↑](#footnote-ref-3)
4. High lactate (hyperlactatemia) is a potentially serious side effect resulting from mitochondrial toxicity in patients who have been on NRTIs (especially d4T and ddI) for > 4 months. If hyperlactatemia is not recognized early, it will progress to lactic acidosis, which carries a significant risk of mortality. A point-of-care lactate machine should ideally be available in all sites where ART is being made available. Any patient developing symptoms of hyperlactatemia (weight loss, fatigue, nausea, vomiting, abdominal pain, and/or shortness of breath) should have a lactate level checked the same day, and be immediately managed by a trained clinician. [↑](#footnote-ref-4)
5. Tuberculosis Handbook WHO. 1998 [↑](#footnote-ref-5)
6. Community Contribution to TB Care: Practice and Policy. WHO. 2003. [↑](#footnote-ref-6)
7. Carol Yu Centre for Infection; http://www.hku.hk/hkucoi [↑](#footnote-ref-7)
8. Pathology Laboratory of Arkansas [↑](#footnote-ref-8)
9. Francis J. Curry National Tuberculosis Center [↑](#footnote-ref-9)
10. Botswana-Baylor Children’s Clinical Centre of Excellence [↑](#footnote-ref-10)
